# Walnut *N*-Acetylserotonin Methyltransferase Gene Family Genome-Wide Identification and Diverse Functions Characterization During Flower Bud Development

**DOI:** 10.3389/fpls.2022.861043

**Published:** 2022-04-15

**Authors:** Kai Ma, Ruiqiang Xu, Yu Zhao, Liqun Han, Yuhui Xu, Lili Li, Juan Wang, Ning Li

**Affiliations:** ^1^Institute of Horticultural Crops, Xinjiang Academy of Agricultural Sciences, Urumqi, China; ^2^Key Laboratory of Horticulture Crop Genomics and Genetic Improvement in Xinjiang, Urumqi, China; ^3^Xinjiang Fruit Science Experiment Station, Ministry of Agriculture and Rural Affairs, Urumqi, China

**Keywords:** melatonin, ASMT (*N*-acetylserotonin methyltransferase), walnut, bioinformatics, floral bud development

## Abstract

Melatonin widely mediates multiple developmental dynamics in plants as a vital growth stimulator, stress protector, and developmental regulator. *N*-acetylserotonin methyltransferase (ASMT) is the key enzyme that catalyzes the final step of melatonin biosynthesis in plants and plays an essential role in the plant melatonin regulatory network. Studies of ASMT have contributed to understanding the mechanism of melatonin biosynthesis in plants. However, *AMST* gene is currently uncharacterized in most plants. In this study, we characterized the *JrASMT* gene family using bioinformatics in a melatonin-rich plant, walnut. Phylogenetic, gene structure, conserved motifs, promoter elements, interacting proteins and miRNA analyses were also performed. The expansion and differentiation of the ASMT family occurred before the onset of the plant terrestrialization. *ASMT* genes were more differentiated in dicotyledonous plants. Forty-six *ASMT* genes were distributed in clusters on 10 chromosomes of walnut. Four *JrASMT* genes had homologous relationships both within walnut and between species. *Cis*-regulatory elements showed that *JrASMT* was mainly induced by light and hormones, and targeted cleavage of miRNA172 and miR399 may be an important pathway to suppress *JrASMT* expression. Transcriptome data showed that 13 *JrASMT* were differentially expressed at different periods of walnut bud development. WGCNA showed that *JrASMT1/10/13/23* were coexpressed with genes regulating cell fate and epigenetic modifications during early physiological differentiation of walnut female flower buds. *JrASMT12/28/37/40* were highly expressed during morphological differentiation of flower buds, associated with altered stress capacity of walnut flower buds, and predicted to be involved in the regulatory network of abscisic acid, salicylic acid, and cytokinin in walnut. The qRT-PCR validated the results of differential expression analysis and further provided three *JrASMT* genes with different expression profiles in walnut flower bud development. Our study explored the evolutionary relationships of the plant *ASMT* gene family and the functional characteristics of walnut *JrASMT*. It provides a valuable perspective for further understanding the complex melatonin mechanisms in plant developmental regulation.

## Introduction

Melatonin (*N*-acetyl-5-methoxytryptamine), a widely known tryptophan derivative that regulates reproductive physiology and circadian rhythms, was initially identified in bovine pineal tissue and subsequently demonstrated to be common in various plant tissues (Dubbels et al., 1995; Hattori et al., 1995; Lerner et al., 2002). Melatonin participates in the growth and development of plants in different tissues and periods (Wang et al., 2016; Zhang et al., 2017), especially as an antioxidant regulating the resistance of plants to various adverse environments (Zhang et al., 2014; Wang et al., 2015), and is considered a potential phytohormone (Arnao and Hernández-Ruiz, 2019). Similar to that in animals, melatonin synthesis in higher plants requires four enzymatic reactions catalyzed by at least six enzymes, including tryptophan decarboxylase (TDC), tryptophan hydroxylase (TPH), tryptamine 5-hydroxylase (T5H), serotonin *N*-acetyltransferase (SNAT), and *N-*acetylserotonin methyltransferase (ASMT; Back et al., 2016). ASMT is involved in the *O*-methylation of *N-*acetylserotonin in the last melatonin biosynthesis step and is regarded as the vital terminal enzyme for melatonin production (Tan and Reiter, 2020).

*ASMT* genes have been identified in more than 10 species, including Arabidopsis (*Arabidopsis thaliana*), rice (*Oryza sativa*), tomato (*Solanum lycopersicum*), pepper (*Capsicum annuum*), apple (*Malus domestica*), and wheat (*Triticum aestivum*; Liu et al., 2017; Pan et al., 2019; Zhan et al., 2019; Bhowal et al., 2021; Wang et al., 2022). Functions of *ASMT* in plant biotic and abiotic stress responses have been extensively characterized. Rice *OsASMT1* was the first *ASMT* gene analyzed and cloned from recombinant *Escherichia coli*, and its expression level was induced by the aging process and highly correlated with melatonin content (Kang et al., 2011). Further studies demonstrated the presence of multiple *ASMT* genes in rice, and independent overexpression of *OsASMT1*, *OsASMT2*, and *OsASMT3* increased rice ASMT enzyme activity and drought stress tolerance (Park et al., 2013a). Increased *AtASMT* expression and enzyme sulfhydryl modification promoted stomatal closure and osmotic stress tolerance in Arabidopsis under exogenous H_2_S treatment (Wang et al., 2021). Cadmium infiltration enhanced *ATASMT1* transcript expression levels and melatonin biosynthesis in mature Arabidopsis leaves (Byeon et al., 2016). Four *SlASMTs in tomato* were thought to respond to multiple pathogens (Liu et al., 2017). The expression of *SlASMTs* was induced by selenium and can alleviate cadmium stress (Li et al., 2016). The apple genome contains at least 37 *MdASMT* genes. Elevated expression of *MdASMT1* increased apple stomatal performance and water content under drought stress. *MdASMT11* and *MdASMT14* are assumed to play essential roles in the response of apple rootstocks to abiotic stresses (Wang et al., 2022). *ASMT* also participated in multiple growth and developmental dynamics in plants. The melatonin-deficient *ASMT* rice exhibited accelerated senescence in detached flag leaves and significantly reduced yield (Byeon and Back, 2016). Overexpression of *AtASMT* caused massive melatonin accumulation and synergized with the phytohormone abscisic acid (ABA) to inhibit seed germination in Arabidopsis (Lv et al., 2021). At least 16 *CaASMT* genes were identified in pepper. They were differentially expressed during pepper pericarp formation and development (Pan et al., 2019). Exogenous melatonin induced the strawberry *ASMT* expression and accelerated the ripening of strawberry fruits through the ABA pathway (Mansouri et al., 2021). Overexpression of *SlASMT* genes increased the heat shock protein (HSP) profile and expression of autophagy-related genes in tomatoes (Xu et al., 2016). *MeASMT2* and *MeASMT3* in cassava simultaneously promote melatonin bioactivation and similarly interact with autophagy-related genes to positively stimulate dynamic changes in cassava autophagic activity (Wei et al., 2020). Although decisive molecular genetic data are not yet available, the possible role of *AMST* in plant flower development has been implied by several researches. Melatonin levels and *OsASMT* expression were induced in parallel during rice flower development (Park et al., 2013b). Three *SlASMT* genes were tissue-specific expressed in flowers and buds in tomatoes (Liu et al., 2017). Seasonal light signal repressed *MdASMT9* expression in apple, thereby promoting flowering (Zhang et al., 2019a).QTL analysis of cowpea [*Vigna unguiculata* (L.) Walp.] RIL population showed that five Arabidopsis *ASMT* orthologs were highly correlated with floral scent (Lo et al., 2020).

Walnuts (*Juglans regia*) are an important nut crop that nutritionists and consumers generally favor due to the high nutritional value of their seeds and their effectiveness in preventing many diseases (Ros et al., 2018). Compared to other nuts, walnuts are richer in melatonin (Verde et al., 2022). The available studies have mainly focused on pathological experiments or nutritional value evaluation of melatonin in walnuts (Bonomini et al., 2018; Cheng et al., 2021; Kamoun et al., 2021; Steffen et al., 2021). However, systematic reports on the mechanism of melatonin synthesis and inheritance in walnut are still lacking. The publication of the whole walnut genome has made it possible to investigate this important fruit crop using bioinformatics (Marrano et al., 2020). Genome-wide isolation and identification of the walnut ASMT gene family have not been reported. In this study, 46 walnut *ASMT* gene family members were identified, and their physicochemical properties, gene characteristics, phylogeny, evolutionary status, promoter *cis*-regulatory elements (CRE), and potential interaction relationships were analyzed by bioinformatics methods. Transcriptome and qRT–PCR data were also used to screen the expression dynamics and differential coexpression network of *JrASMTs* during the development of walnut flower buds. This research provides a global reference for the functional study of the *ASMT* gene family in walnut and a theoretical basis for exploring the function and molecular mechanism of melatonin during walnut flower bud development.

## Materials and Methods

### Plant Materials

Conventionally cultivated walnut (*J. regia*, “Xinxin 2”) was used as research material in this study. Walnuts were grown under natural conditions at Xinjiang Fruit Science Experiment Station of Ministry of Agriculture and Rural Affairs (Yecheng, China). Female flower buds from the terminal buds’ lower 2–3 positions were collected on April 15 (qP1), May 15 (qP2), May 30 (qS1), June 15 (qS2), and July 15 (qS3) in 2021, respectively. All floral bud samples were collected after removing the outer skin and fixed by flavonoid acetic acid (FAA) for subsequent studies.

### *ASMT* Gene Family Identification

Complete genomic data for all species used in this study were downloaded from the EnsemblPlants^1^ and Phytozome^2^ public databases; Markov model files of the dimerization domain (PF16864) and *O-*methyltransferase domain (PF00891) were downloaded from the Pfam^3^ public database (El-Gebali et al., 2019). The amino acid sequences of OsASMT1/2/3 downloaded from the Rice Genome Annotation Project^4^ and AtASMT1 downloaded from The Arabidopsis Information Resource^5^ were used as query sequences for local BLAST (*E*-value: 1e^−5^); HMMER software was simultaneously used to search for walnut protein sequences (*E*-value: 1e^−5^; Wheeler and Eddy, 2013). Amino acid sequences of all subsequent genes were verified by SMART,^6^ Pfam,^7^ and CDD^8^ databases for complete conserved structural domains (Schultz et al., 2000; Marchler-Bauer et al., 2007). *ASMT* genes of Arabidopsis (*A. thaliana*), tomato (*S. lycopersicum*), rice (*O. sativa*), sorghum (*Sorghum bicolor*) and Apple (*M. domestica*) were collected from published studies (Zhan et al., 2019; Bhowal et al., 2021; Wang et al., 2022). Confirmed walnut ASMT genes were submitted to the ExPASy^9^ and WoLF PSORT^10^ online sites to calculate physicochemical properties and predict subcellular localization.

### Gene Structure, Conserved Motifs, and Phylogenetic Analysis

Annotation information of ASMT genes was extracted from walnut whole genome files using Perl scripts. MEME software was used to analyze the conserved motifs of JrASMTs (motif number: 20, min-width: 6; Bailey et al., 2009). TBtools was used to visualize the gene structure and conserved motifs (Chen et al., 2020). Multiplex sequence alignment was performed using Clustal X 2.1 (Larkin et al., 2007). Phylogenetic trees were constructed by IQ-TREE software (automatic calculation of the optimal model, bootstrap ≥1,000; Minh et al., 2020). The ASMT amino acid sequences of all species were used to construct species phylogenetic trees based on OrthoFinder (STAG algorithm; Emms and Kelly, 2019). The evolutionary tree was embellished using the iTOL^11^ online website.

### Homologous Genes, *cis*-Regulatory Elements, and Interaction Network Analysis

Inter- and intraspecies synteny and collinearity analysis was performed using MCScanX software (Wang et al., 2012). Synonymous and nonsynonymous substitution rates between paralogous homologous pairs were calculated by KaKs Calculator 2.0 (Wang et al., 2010). The CREs in the *ASMT* promoter sequences (2,000 bp sequence upstream of each gene) were predicted with PlantCARE^12^ (Lescot et al., 2002). Visualization of homologous relationships and CREs was also performed using TBtools. All JrASMT proteins were submitted to STRING^13^ to predict interactions (Szklarczyk et al., 2019). The miRNAs targeting *ASMT* were predicted through the PmiREN^14^ online website (Expectation: 5, Gaps: 0, Identity: 12; Guo et al., 2020). The interaction network was visualized by Cytoscape software (Su et al., 2014).

### Analysis of *JrASMT* Gene Expression Profiles in Flower Bud Development

RNA-seq data for early physiological differentiation (P1), late physiological differentiation (P2), critical morphological differentiation (PS), early morphological differentiation (S1), and late morphological differentiation (S2) of female walnut buds were obtained from published studies (PRJNA673588).

All transcriptome data were mapped to the walnut reference genome by HISAT2 (Kim et al., 2019). Gene expression was normalized by fragments per kilobase of exon model per million mapped reads (FPKM). Differentially expressed genes were filtered using DESeq2 (Love et al., 2014), with differential levels set to absolute values of the FoldChange greater than 2 and FDR values less than 0.01. The differentially expressed genes were plotted and hierarchically clustered based on TBtools.

### Weighted Gene Coexpression Network Analysis

Weighted gene coexpression network analysis (WGCNA) of all differentially expressed genes was performed based on the publicly available R package (“WGCNA”) in the R language (v 4.1.2) environment (Langfelder and Horvath, 2008). Genes with a mean FPKM of less than 0.1 and those with more than 50% deletions in a set of replicates were removed. Soft thresholds were calculated by the scale-free topological fit index and mean connectivity. Hierarchical clustering of genes was performed based on the topological overlap measure (TOM). The minimum number of genes in the module was 60, and high similarity modules were merged based on a threshold of 0.2.

### Gene Function Annotation and Core Network Extraction

*De novo* annotation of transcriptome data was completed *via* eggNOG-Mapper (v 5.0; Huerta-Cepas et al., 2017). Enrichment analysis (*p*-value < 0.01) and visualization of annotation results from Gene Ontology (GO) and Kyoto Encyclopedia of Genes and Genomes (KEGG) were performed using R. The cytoHubba plugin in Cytoscape was used to extract specific modules with *JrASMTs* with high connectivity (Chin et al., 2014). Cytoscape was used to visualize core coexpression networks.

### Paraffin Sectioning and Real-Time Fluorescence Quantification

Walnut female bud samples fixed in FAA for 24 h and softened by glycerol immersion were treated with a continuous gradient of ethanol (70, 85, 95, and 100%) and a mixture of anhydrous ethanol and xylene (*v*:*v* = 1:1) for dehydration, and then were immersed in 60°C liquid paraffin. Slicing was done using a Leica Microtome (Leica, Weztlar, Germany) with a thickness of 8 μM, sections were stained using Fast Green FCF (Sangon Biotech, Shanghai, China), Nikon Eclipse Ts2 microscope (Nikon, Shanghai, China) was used for observation of the sections, and images were processed using NIS-Elements.

Total RNA was extracted from different tissues by RNA extraction kits (Tiangen, DP441, Beijing, China). First-strand cDNA was synthesized using Vazyme HiScript II 1st Strand cDNA Synthesis Kit (Vazyme Biotech Co., Ltd., China). Specific primers were designed based on 18 selected coding sequences of *JrASMTs* (Supplementary Table 1), and Sangon Biotech Co., Ltd. (Shanghai, China) synthesized the primers. The Actin gene was used as an internal reference and real-time fluorescence quantification by Maxima SYBR Green/ROX qPCR Master Mix. Three biological replicates were included in this experiment, and gene expression was calculated by 2^−ΔΔCt^ (Livak and Schmittgen, 2001). qP1 period samples were set as the control.

## Results

### Genome-Wide Identification of the Walnut *ASMT* Family

The whole-genome sequence of walnut was used to identify ASMT proteins. Candidate genes were initially screened by a local BLAST search of query sequences and an hmmsearch of Markov model files. A total of 46 genes were finally identified as members of the walnut ASMT family by validation of their conserved structural domains and were named in order of their position on the walnut genome (*JrASMT1-46*). The physical and chemical properties showed that most of the walnut ASMT proteins (71.74%) had amino acid lengths ranging from 335 aa to 385 aa (Supplementary Table 2). Forty-one (89.13%) JrASMTs were acidic proteins, and the isoelectric points (pI) of all JrASMT proteins ranged from 4.91 to 8.91, with an aliphatic index of 71.7 to 105.02. Nineteen (41.30%) JrASMTs were hydrophobic proteins, with GRAVY values ranging from 0 to 0.175. Twenty-six (56.52%) JrASMTs were hydrophilic proteins, with GRAVY values ranging from −0.017 to −0.247. The prediction of subcellular localization indicated that most of the JrASMT proteins were distributed in the cytoplasm (29), and the remaining JrASMT proteins were distributed in the cytoskeleton (12), nucleus (2), chloroplast (2), and peroxisome (1).

### Phylogeny, Gene Structure, and Conserved Motif Analysis of *JrASMTs*

**Figure 1:**
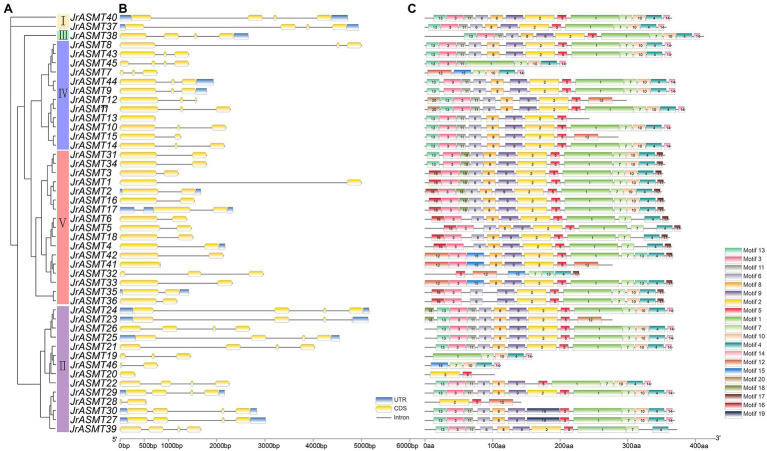
Phylogenetic, gene structure, and motif analysis of walnut *JrASMT* genes. **(A)** The phylogenetic tree was constructed by IQ-TREE. Forty-six *ASMT* genes were divided into five subgroups (I–V). **(B)** TBtools were used to show the gene structure, with UTRs in blue, exons in yellow, and introns in black solid lines. **(C)** The motifs of JrASMT. Rectangles with different colors and numbers represented different motifs.

The phylogenetic tree was constructed with the amino acid sequences of all JrASMTs to explore the relationships among walnut ASMTs. Forty-six JrASMTs were distributed in five evolutionary distinct groups (Figure 1A). Group I contained only two members, JrASMT40 and JrASMT37. Group II contained 14 JrASMTs. Group III, Group IV, and Group V were located in a common internal node. Among them, Group III contained only one JrASMT38, which was considered to be an outgroup. Group IV contained 12 JrASMTs. Group V contained the largest number of JrASMTs at 17. Gene structure analysis showed that *JrASMT* genes contained 1–4 exons and 1–3 introns, and only 11 *JrASMT* genes contained complete 3′ and 5’ UTRs. *JrASMT13* and *JrASMT41* contained only one exon and one intron (Figure 1B). Conserved motif analysis showed that some JrASMT genes have more conserved motif sequences than others (Figure 1C), four JrASMT (JrASMT28, JrASMT20, JrASMT46, JrASMT19) genes had only partial motifs due to their short protein length. The different groups of JrASMT proteins contained different motifs, mainly at the 5′ terminus (motif13, motif26) or 3′ terminus (motif14, motif17).

### Evolutionary Relationship and Phylogenetic Analysis of *JrASMTs* in Multiple Plants

**Figure 2:**
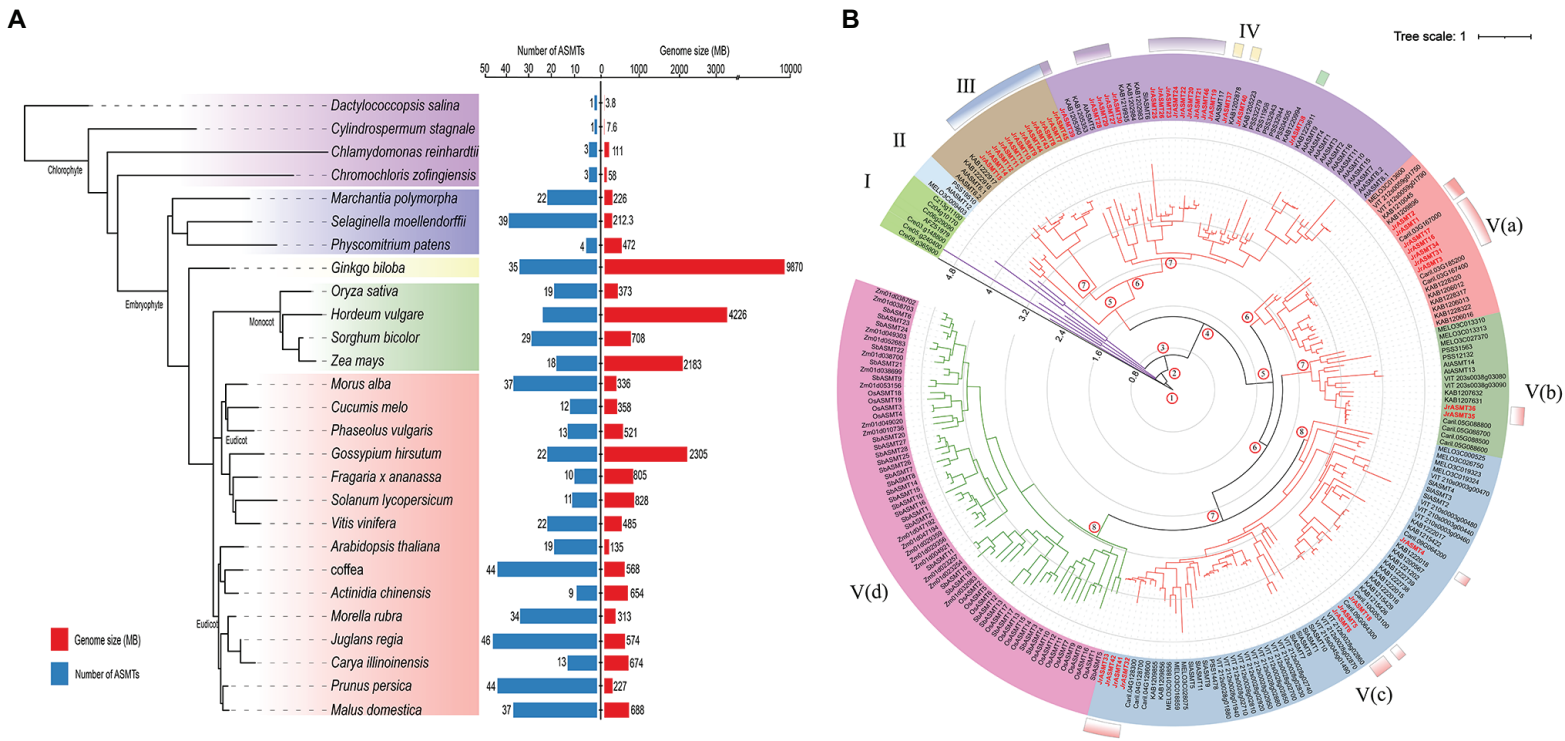
Numerical statistics and taxonomic status analysis of the ASMT family in multiple plants. **(A)** The species tree of the ASMT family was constructed based on OrthoFinder. All selected plants were divided into four subgroups, distinguished by different colored branches. The statistics of the respective genome size and number of ASMT family members for each species were shown on the right. **(B)** The phylogenetic tree was constructed with IQ-TREE. The *ASMT* of algae was used as the midpoint root. Different colored branches represented different taxonomic levels, purple for algae, red for dicotyledons and green for monocotyledons. The length of the branches represented the evolutionary distance. Two hundred and thirty-seven *ASMT* genes were divided into five subgroups (I–V) and displayed in different background colors. The black axes inside the evolutionary tree displayed the tree scale. The numbers at the internal nodes represented the level of clades. The classification results of JrASMT in Figure 1 were displayed in the outer circle of the evolutionary tree as identical gradient color rectangles.

To assess the evolutionary status of *JrASMT* in major plant species, 584 ASMT proteins were identified from 27 plant species by multiple methods (Supplementary File 1). These plants included four algae, two mosses, one pteridophyte, one gymnosperm, four monocotyledons, and 15 dicotyledons. All protein sequences were manually validated for structural domains by the CDD, Pfam, and SMART databases to ensure accurate identification. The species tree constructed base on OrthoFinder showed that *ASMT* genes were present in all selected species (Figure 2A). Only a single copy of the *ASMT* gene was present in the cyanobacteria *D. salina* and *C. stagnale*. Two green algae, *C. reinhardtii* and *C. zofingiensis*, contained 3 *ASMT* genes each. All selected embryophytes contained at least nine *ASMT* genes, with *A. thaliana* containing 19 *ASMT* genes and *J. regia* containing the most *ASMT* genes at 46. A clear differentiation among *ASMT* genes occurred at different taxonomic levels. Dicotyledonous and monocotyledonous plants were strictly assigned to different branches on the species tree. The number of *ASMT* genes was not strongly correlated with species genome size. The larger genomes of *H. vulgare*, *Z. mays*, and *G. hirsutum* contained only 24, 18, and 22 *ASMT* genes. Woody plants seemed to possess more *ASMT* genes. In addition to walnuts, *P. persica*, *Morella rubra*, *M. alba*, and *M. domestica* contained 44, 34, 37, and 37 *ASMT* genes, respectively. However, *A. chinensis* contained only nine *ASMTs*.

The 237 protein sequences from 14 species, which contained one cyanobacteria, two green algae, three monocotyledons, and eight dicotyledons, were used to construct a phylogenetic tree with the *D. salina* ASMT protein as the midpoint root (Figure 2B). All ASMT proteins were distributed within five main different subgroups (I–V), the subgroup V were further elaborated into Va, Vb, Vc, and Vd. With subgroup I containing only algae distributed in four clades. Subgroup V(d) contained only monocotyledonous plants, and the *ASMT* genes in this subgroup shared similar branch lengths and close evolutionary distances. Interestingly, a general divergence of the ASMT family in eudicot occurred, as they were distributed in subgroups II-V. Subgroups IV and V(c) contained 46 and 63 *ASMT* genes, respectively, which are relatively high numbers. Subgroup II contained the fewest genes, with only three *ASMTs* from *A. thaliana*, *Cucumis melo*, and *A. chinensis*. Subgroup V(a) contained genes from five species: *C. melo*, *Vitis vinifera*, *M. rubra*, *J. regia* and *C. illinoinensis*. Specifically, Arabidopsis *ASMTs* were absent from this group. Walnut *ASMT* genes were distributed in five of the four subgroups of Dictyostelium, but each subgroup member did not match the grouping results of the previous phylogenetic tree (Figure 1A). The introduction of many outgroups allowed for a more precise delineation of the *JrASMT* divergence relationships.

### Analysis of Paralogous and Direct Homologous Genes

**Figure 3:**
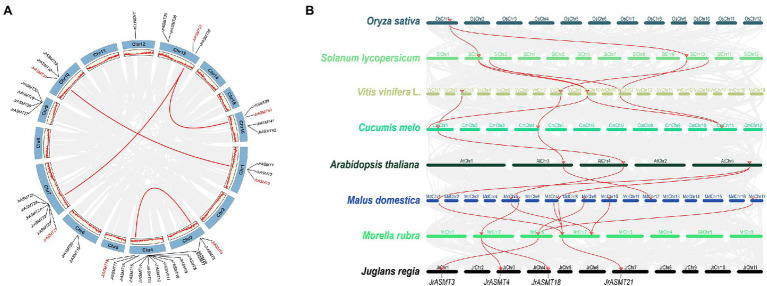
Synteny and collinearity analysis of the *ASMT* family. **(A)** Distribution of *JrASMTs* on walnut chromosomes and analyzed of paralogous genes. The outer circle indicated the 16 chromosomes of walnut, and the inner circle showed the density of genes on the chromosomes. The gray lines represented genes and blocks with paralogous relationship, the red lines represented paralogous *JrASMT* genes, and the corresponding *JrASMTs* name were marked in red. **(B)** Collinearity analysis of *ASMT* in multiple species. Latin names of species were labeled on the left. The ranking order was according to the proximity of the relatives in the species tree. Chromosomes of different species were shown in different colors. The gray lines represented genes or blocks with collinearity, and the red lines were *ASMT* genes with collinearity. Four *JrASMTs* name were labeled below.

Gene doubling and duplication are important pathways for the adaptive evolution of organisms and the acquisition of diverse phenotypes. The 46 putative *JrASMT* genes were unevenly distributed on the 10 chromosomes of walnut (Figure 3A). Twelve (26.09%) of these *JrASMT* genes were concentrated on walnut chromosome 4 (Chr4), which had the highest number. In contrast, only 1 (2.17%) *JrASMT* was found on chromosome 12 (Chr12). The results of the walnut ASMT collinearity gene analysis showed that only 7 (15.22%) *JrASMTs* were found to have four pairs of paralogous homologs (*JrASMT3*/*JrASMT31*, *JrASMT4*/*JrASMT18*, *JrASMT21*/*JrASMT37* and *JrASMT37*/*JrASMT40*). *JrASMT3*/*JrASMT31* was classified in subgroup V(a) of the phylogenetic tree (Figure 2B). *JrASMT4*/*JrASMT18* was classified in subgroup V(c). *JrASMT21*/*JrASMT37* and *JrASMT37*/*JrASMT40* were both classified in subgroup IV. All paralogous homologous genes were derived from segmental duplication, and no tandem duplication events occurred. Synonymous and nonsynonymous mutation rates were calculated for all paralogous homologous genes (Supplementary Table 3). The Ka/Ks values for both homologous pairs *JrASMT37*/*JrASMT21* and *JrASMT4*/*JrASMT18* were close to 1, and these genes were almost free from environmental selection and showed neutral evolution, while gene pairs *JrASMT3*/*JrASMT31* and *JrASMT37*/*JrASMT40* had Ka/Ks values of 0.296 and 0.092, respectively, suggesting that these two gene pairs were subject to strong purifying selection and were functionally stable.

The *ASMT* genes from eight plant species were chosen for collinearity analysis according to their distant relationships on the species phylogenetic tree (Figure 2A), including one monocotyledon (*O. sativa*) and seven dicotyledons (*S. lycopersicum*, *V. vinifera* L., *C. melo*, *A. thaliana*, *M. domestica*, *M. rubra*, *J. regia*; Figure 3B). Rice contained only one gene that has orthologous relationship with two genes in the dicotyledonous tomato plant, while the number of orthologous genes increased significantly inside the dicotyledonous clade. There were five pairs of orthologous relationships formed by four *ASMT* genes, with two each between tomato and grape, grape and melon, although the origin of these orthologous relationships was not the same. Only two ASMT gene pairs had four orthologous relationship between melon and Arabidopsis. In direct contrast, there were eight direct orthologous gene pairs between the nine apple *ASMT* genes and the four poppy *ASMT* genes. Three poplar *ASMT* genes were orthology to *JrASMT1*, *JrASMT4*, *JrASMT18*, and *JrASMT21*, and these same *JrASMTs* were located in subgroups IV, V(a), and V(c). *JrASMT4* and *JrASMT18* shared the same orthologous *ASMT* gene with poplar. Collectively, there are four conserved direct homologous relationships within the ASMT gene family among dicotyledonous plants.

### Analysis of *cis*-Regulatory Elements in *JrASMTs*

**Figure 4:**
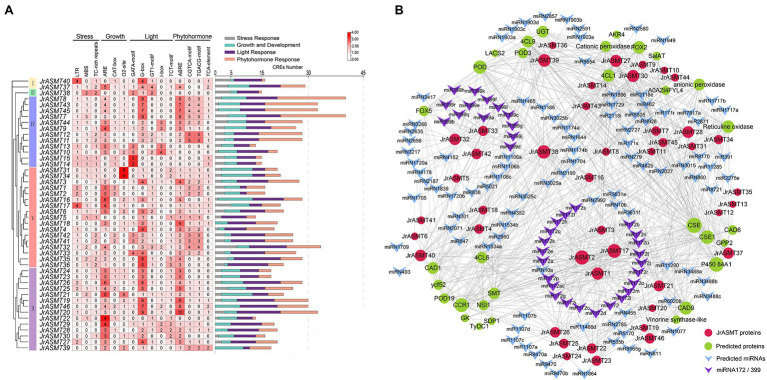
CRE analysis of the promoter region of *JrASMT*, and interacting proteins and miRNAs prediction. **(A)** The CRE statistics of *JrASMT* promoter regions. CREs were classified into four categories: stress-response, growth and development, light-response and phytohormone-response. *JrASMT* was arranged in the order of phylogenetic tree. The names of CREs were marked on the top. The heat map showed the number of 15 main CREs corresponding to each *JrASMT*. The bar chart on the right side counted all the number of four types of CREs in each *JrASMT*, and different types of CREs were indicated by different colors. **(B)** Network diagram of proteins and miRNAs interacted with JrASMT. Gray connecting lines indicated the presence of interactions. JrASMT was represented by red circles, and interacting proteins were represented by light green circles, with the size of the circles represented the number of interaction relationships. miRNAs were represented by V-shaped rectangles, where miRNA172 and miRNA399 were shown in purple.

Transcription factors regulate gene expression by binding to CRE sites in promoter regions. To explore the possible biological functions and expression patterns of walnut *ASMT* genes, the 2,000 bp upstream sequences of all *JrASMT* genes were subjected to CRE analysis (Figure 4A). Except for core response elements and functionally unknown elements, a total of 1,076 CREs were characterized in all *JrASMT* promoter regions. These CREs could be broadly classified into four categories: light-response elements (34.94%), growth- and development-related elements (21.84%), stress-response elements (10.41%) and hormone-response elements (32.81%). Among them, there were 21 types of light-response elements, mainly G-boxes (13.38%), GATA motifs (3.07%), GT1 motifs (3.25%), *JrASMT* expression was widely regulated by multiple modes of light-mediated. There were 11 types of growth- and development-related elements, including hyphal tissue-specific expression element CAT-box (2.23%) and circadian rhythm element circadian (1.49%). There were six types of stress-response elements, mainly the LTR (3.25%) and MBS (2.70%). It suggested that *JrASMT* was sensitive to low temperature and drought environment. *JrASMT* was extensively involved in walnut hormonal regulatory networks, 11 hormone-response elements were observed, including the ABA response element ABRE (12.17%); growth hormone response elements AuxRR-core (1.30%); jasmonic acid (JA) response elements CGTCA-motif (6.13%) and TGACG-motif (6.13%); gibberellin response elements P-box (1.30%); and salicylic acid (SA) response elements TCA-element (2.23%). More than 30 CREs were found in the promoter regions of *JrASMT8*, *JrASMT43*, *JrASMT45*, *JrASMT7*, *JrASMT32*, and *JrASMT20*, and multiple pathways may widely regulate these genes in walnut. *JrASMT13*, *JrASMT5*, and *JrASMT22* each had fewer than 15 CREs in the promoter region, implying that these genes expression may be mediated in fewer ways. The promoter regions shared a similar distribution of CREs among the more closely related *JrASMT* genes. The *JrASMT8*, *JrASMT43*, *JrASMT45*, and *JrASMT7* genes in subgroup IV, which were distributed in the same clade, all contained a large number of G-box elements and hormone-related elements. The promoter regions of the *JrASMT31* and *JrASMT34* genes in subgroup V(a) contained only O2-site elements. *JrASMT23*, *JrASMT24*, *JrASMT25*, and *JrASMT26* in subgroup II also showed similar transcription factor binding sites, and they all contained high amounts of AREs, G-boxes, and ABREs. Overall, these results provided evidence for the expression patterns of walnut *ASMT* genes.

### Network Analysis of *JrASMT* Proteins and miRNAs

Plants possess complex and indirect gene expression networks. Molecular interactions are an important component of plant complicated expression patterns. The interrelationship between miRNA and mRNA or between proteins is essential for plants to display different expression patterns. Based on online databases, potential miRNAs and proteins that target or interact with JrASMT were predicted (Supplementary Table 4). Only six JrASMT proteins (JrASMT27/28/30 and JrASMT37/39/40) were predicted to exhibit nine pairs of direct interactions with each other (Supplementary Figure 1). A total of 27 JrASMT proteins were predicted to directly or indirectly interact with 31 proteins (Figure 4B), the main ones being CAFFEOYL SHIKIMATE ESTERASE (CSE), cytochrome P450 84A1, cinnamoyl-CoA reductase (CCR), peroxidase (POD) and cinnamyl alcohol dehydrogenase (CAD), which interacted with 40, 37, 34, 31, and 30 proteins, respectively. Forty-two *JrASMT* genes were predicted to have potential targeting relationships with 132 miRNAs from 75 families (Supplementary Table 3). miR2118, miR482, miRN2785, miRN1729, and miR5505 interacted with the most genes, targeting 9, 9, 7, 6, and 5 *JrASMTs*, respectively. The miR172 and miR399 families were the most abundant, with 23 types miR172 and 13 types miR399 targeting the *JrASMT1/2/3/17* and *JrASMT32/33/42* genes, respectively. JrASMTs distributed in the same clade on the phylogenetic tree had similar protein interaction relationships. The *JrASMT27/28/30/37/38/39/40* genes classified in subgroup IV had an almost identical number of interactions (mean degree = 87), and the other six genes (*JrASMT21/22/23/25/26/46*) in this subgroup had a degree of 59. Five genes (*JrASMT2/3/16/17/34*) in subgroup V(a) all had a degree equal to 18, while the degrees for two genes (*JrASMT35/36*) in subgroup V(b) and the seven genes (*JrASMT4/5/6/18/32/41/42*) in subgroup V(c) were all equal to 2. There may be complex synergistic expression or functional redundancy of these genes.

### Expression Dynamics of *JrASMTs* During Walnut Bud Development

**Figure 5:**
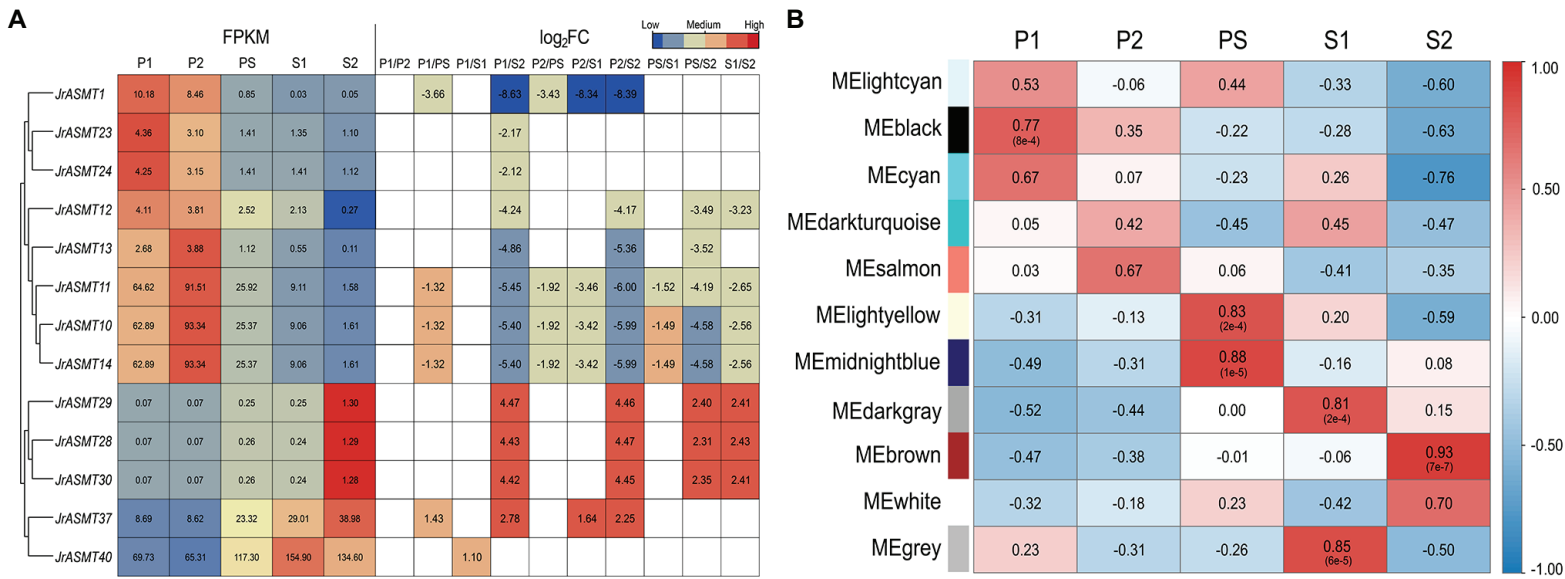
The expression analysis of *JrASMTs* in walnut flower bud development. **(A)** The DEG heat map of *JrASMT*. Genes were hierarchically clustered based on expressions. The normalized expressions were shown on the left, and the fold change under each difference grouping were shown on the right, in log_2_FC. The colors of the heat map represent the magnitude of expressions and fold change. **(B)** Correlation of each module with the developmental period. The module names were on the left and the different periods were on top. The color of the heat map represented the magnitude and properties of the correlation. The numbers in the heat map were the correlation values and the numbers in parentheses were the *p*-values.

We retrieved the expression patterns of all *JrASMT* family genes during physiological differentiation (P1, P2), the physiological differentiation and morphological differentiation critical period (PS), and morphological differentiation (S1, S2) of walnut buds from published transcriptome data. Significant differential expression occurred in 13 of 46 *JrASMT* genes during walnut flower bud development. The heatmap shows that differentially expressed *ASMT* genes were hierarchically clustered into two groups based on their expression during the P and S periods (Figure 5A). *JrASMT1*, *JrASMT10-14*, *JrASMT23*, and *JrASMT24* showed similar expression patterns; all eight genes had the highest expression in the P1 and P2 periods and decreased expression in the PS-S2 period. The expression of *JrASMT28/29/30*, *JrASMT37*, and *JrASMT40* increased during later floral bud development: *JrASMT28/29/30* were highly expressed during S2, and both *JrASMT37* and *JrASMT40* were highly expressed during PS-S2. Among all the differentially expressed *JrASMTs*, only *JrASMT40* was relatively highly expressed in all periods, reaching a significant difference only under the P1 vs. S1 comparison, indicating that this gene may play an essential role in walnut flower bud development.

To further determine the underlying functions of *JrASMT* genes in walnut bud development, all differentially expressed genes (DEGs) were used to conduct weighted gene coexpression network analysis after data filtering. The soft threshold for constructing the adjacency matrix was 27 (correlation over 78%). Except for those at PS and S1, walnut buds at different developmental stages showed significant differences in their expression patterns (Supplementary Figure 2). Total of 12,664 DEGs were assigned in 11 merged modules (Supplementary Figure 3). The connectivity of eigengenes was analyzed for differences between modules and the magnitude of association between module genes and traits, and the results showed significant differences between modules (Figure 5B, Supplementary Figure 4). The black module containing 4,316 genes was highly correlated with the P1 period (*p* = 8e^−4^, *R* = 0.77). The light-yellow module containing 263 genes (*p* = 2e^−4^, *R* = 0.83) and the midnight-blue module containing 334 genes (*p* = 1e^−5^, *R* = 0.88) were highly correlated with the PS period. The dark-gray module containing 220 genes (*p* = 2e^−4^, *R* = 0.81) and the gray module containing 1,181 genes (*p* = 6e^−5^, *R* = 0.85) were highly correlated with the S1 period, and the brown module containing 4,528 genes was highly correlated with the S2 period (*p* = 7e^−7^, *R* = 0.93).

### Association Module and Functional Prediction of *JrASMTs*

**Figure 6:**
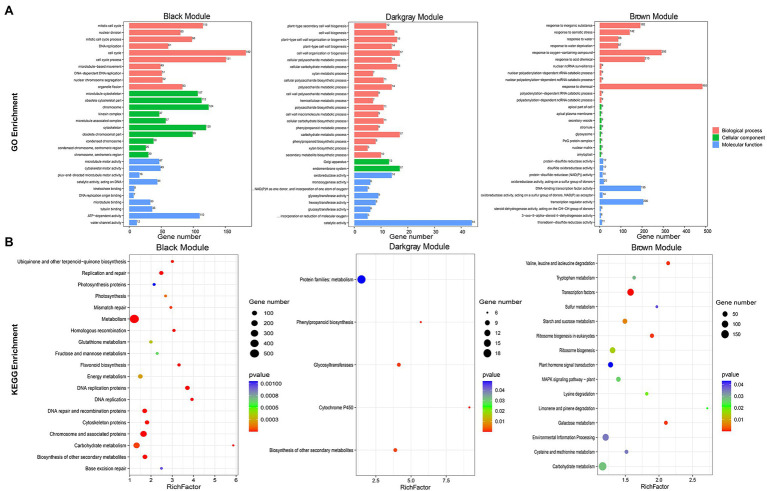
Functional annotation of black, dark-gray, and brown modules genes. **(A)** Bar chart of GO enrichment for the three modular genes. GO enrichment terms were shown on the left. Module names were labeled above the bar graph. **(B)** Bubble plots of KEGG enrichment for the three modular genes. KEGG enrichment pathway were shown on the left. The RichFactor represented the level of enrichment. The size of dots represented the number of genes. The color of the dots represented the q-value of the enrichment. The module names were labeled above the bubble diagram.

*JrASMT1/10/13/23* were specifically classified in the black module, *JrASMT40* was specifically classified in the dark-gray module, and *JrASMT12/28/37* were specifically classified in the brown module. GO, and KEGG annotation and enrichment analysis were performed for genes in these three modules (Figure 6). In the black module, 182 (8.90%) genes were enriched in the cell cycle term, 115 (5.62%) genes were enriched in the mitotic cell cycle term, 206 (12.96%) genes were enriched in the chromosome and associated protein pathway, and 187 (11.76%) genes were enriched in the carbohydrate metabolism pathway. These results suggested that genes in the black module played roles in cell cycle regulation, DNA replication and repair, and nuclear chromosome segregation. In the dark-gray module, 44 (12.61%) genes were enriched in the catalytic activity term, 17 (4.87%) genes in the endomembrane system term, 17 (4.87%) genes in the cell wall organization or biogenesis term, and 18 (1.48%) genes in protein families: metabolism pathway. A large number of DEGs were also enriched in functions related to cell wall synthesis and metabolism, glycolic acid, lignin, cellulose, and other metabolic processes in plants. This result indicated that genes in the dark-gray module were involved in cell wall formation during morphological differentiation of walnut flower buds and mediated various secondary metabolic pathways. In the brown module, 485 (23.00%) genes were enriched in response to chemical terms, 292 (13.85%) genes in response to oxygen-containing compound terms, 206 (9.77%) genes in transcription regulator activity terms, 92 (13.53%) genes in environmental information processing pathways, and 51 (7.50%) genes in plant hormone signals. The genes of the brown module were significantly associated with the response of walnut buds to various environmental factors and extensively involved in multiple signaling and hormone regulatory networks in the late stage of walnut bud morphological differentiation.

### The Coexpression Network of *JrASMTs* in Walnut Flower Bud Development

**Figure 7:**
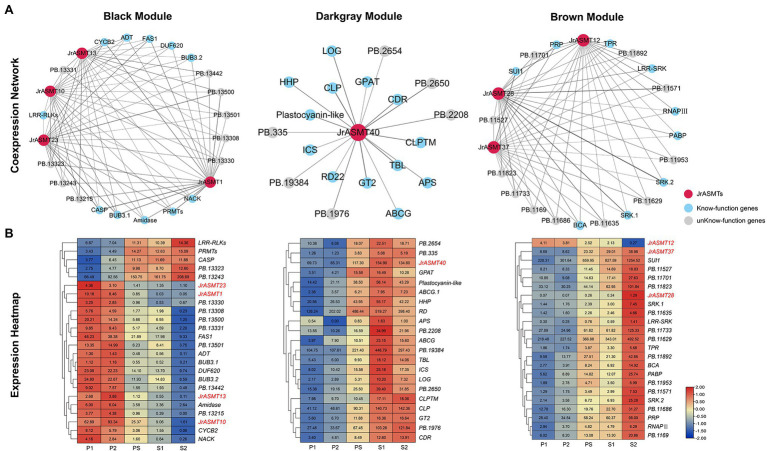
Construction and expression analysis of core coexpression networks. **(A)** The core coexpression networks with *JrASMT* in the three modules. *JrASMT* was represented by red circles, known-function genes were represented by light blue circles, and unknown-function genes were represented by gray circles. Gray connecting lines indicated the presence of coexpression relationships, the thickness of the line indicated the confidence level of coexpression. The names of the modules were labeled above the network diagram. **(B)** Heat map of gene expression in the three coexpression networks. The horizontal axis was samples from different periods. The vertical axis was the genes name. The different colors represent the high or low expression levels. All genes were clustered hierarchically based on expression patterns. *JrASMT* was labeled in red.

Genes coexpressed with *JrASMTs* in the three modules were screened. The black, dark-gray and brown modules contained 500 (11.58%), 91 (41.36%) and 582 (12.85%) genes coexpressed with *JrASMT* genes, respectively. The core subnetworks of the three modules were extracted by cytoHubba. The interactions and expressions were visualized (Figure 7). Except for genes with unknown functions, in the black module, *JrASMT1/10/13/23* were coexpressed with several cell process-related genes, such as the mitotic checkpoint protein coding gene Budding uninhibited by benzimidazole (*BUB*), which is involved in the cell cycle; Protein arginine methyltransferase (*PRMT*); Cyclin B2 (*CYCB2*), which belongs to the cyclin family; and NPK1-activating kinesin (*NACK*), which belongs to the TRAFAC class of myosins. In addition, the *JrASMT* genes in the black module were also associated with the Fasciclin I family protein coding gene (*FAS1*), glutamyl-tRNA (Gln) amidotransferase subunit coding gene (*Amidase*), Casparian strip membrane domain protein (*CASP*), and other coexpressed genes. Almost all functionally known coexpressed genes were highly expressed during P1 and P2, except for LRR receptor-like serine threonine-protein kinase (*LRR-RLKs*), *PRMT*, and *CASP*, which had elevated expression during morphological differentiation. In contrast, the genes coexpressed with *JrASMT40* in the dark-gray module were more functionally diverse, containing Lonely guy (*LOG*), which activates the cytokinin-activating enzyme Constitutive disease resistance (*CDR*) and Responsive to desiccation 22 (*RD22*). Two transporter protein family members ATP-binding cassette (*ABCG*) and Hypothetical protein (*HHP*) involved in the AMPK signaling pathway; Adenosine-5′-phosphosulfate (*APS*) involved in the purine metabolism pathway; and Glycerol-3-phosphate acyltransferase (*GPAT*) involved in the glycerolipid metabolism pathway, were found in the coexpression network that also included Chitinase-like protein (*CTL*), Trichome birefringence-like (*TBL*), and Glycosyltransferase 2 (*GT2*) involved in cellulose synthase, encoding Isochorismatase family protein (*ICS*). All genes expression levels in the dark-gray module increased during the PS period; most reached maximum expression at the S1 period and remained unchanged or decreased at the S2 period. In the brown module, *JrASMT12/28/37* was coexpressed with the gene encoding DNA-directed RNA polymerase III subunit (*RNAPIII*), protein translation factor SUI1 homolog coding gene (*SUI1*), Poly(A) binding protein (*PABP*), and other genes. These genes are involved in transcriptional regulation or translational modifications. The network likewise contained multiple Receptor-like serine–threonine protein kinases (*SRKs*) and *LRR-RLKs*. *JrASMT12/28/37* was also coexpressed with Beta carbonic anhydrase (*BCA*), Proline/serine-rich protein (*PRP*), and a Tetratricopeptide repeat protein coding gene (*TPR*). All coexpressed genes were most highly expressed during the S2 period. However, *JrASMT12* was more highly expressed during the P1 and P2 periods.

### Tissue Sectioning and Real-Time Fluorescence Quantification

**Figure 8:**
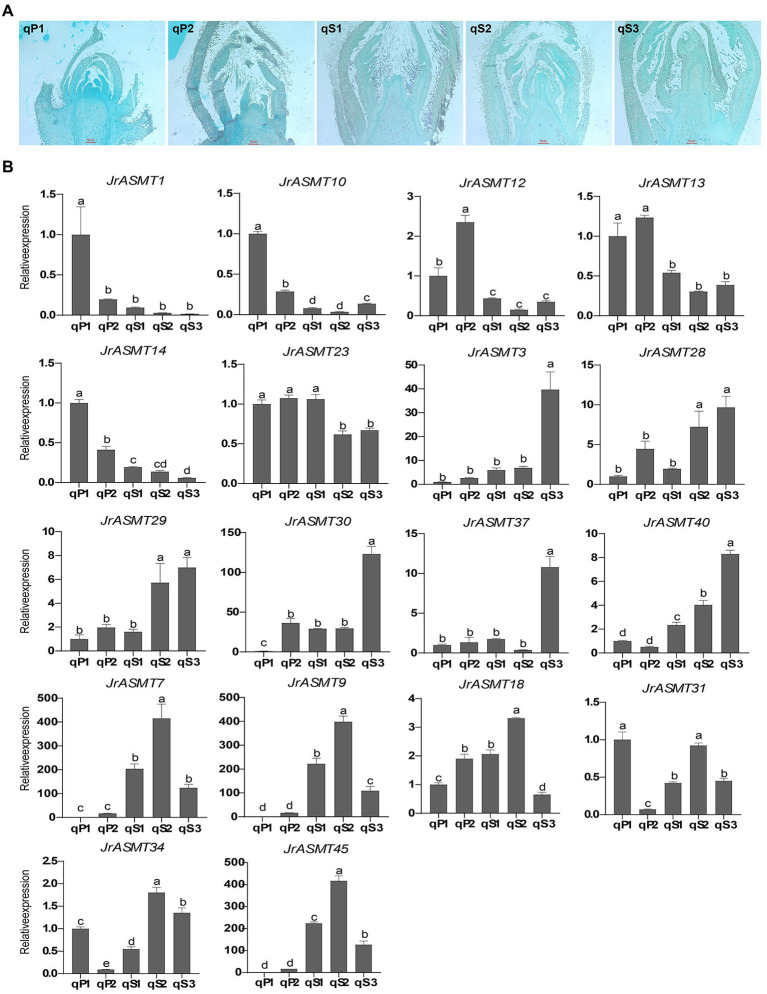
Paraffin sectioning and Real-time quantitative PCR. **(A)** Results of paraffin sections of female flower buds of walnut at different periods, corresponding sample numbers were marked in the upper left corner. **(B)** Bar chart of the 18 *JrASMTs* Real-time quantitative PCR. The horizontal axis showed the names of samples from different periods. The vertical axis showed the real-time quantitative result. The corresponding *JrASMTs* name was labeled above the bar chart. The standard deviations were shown with error bars. Letters indicated the significance of differences between expressions. The expression at qP1 was used for normalization.

To verify the expression pattern of *JrASMT* during the development of walnut buds, flower bud tissues from walnuts at different periods were collected. The Paraffin section results showed that the walnut buds in the qP1 and qP2 periods were undergoing physiological differentiation (Figure 8A), and those in qS1, qS2, and qS3 were undergoing bud morphological differentiation. Eighteen *JrASMTs*, including 11 DEGs in the transcriptome and seven non-DEGs, were used for real-time fluorescence quantification in these samples (Figure 8B). Similar to several previous results, multiple *JrASMTs* were significantly differentially expressed and clustered at different periods of walnut bud development. *JrASMT1/10/12/13/14* were significantly higher in qP1 and qP2 but barely expressed during morphological differentiation. *JrASMT23* had no significant differential expression in the qP1, qP2, and qS1 periods but was slightly decreased during the qS2 and qS3 periods. *JrASMT3/28/29/30/37/40* were commonly expressed at different stages. However, they were only highly and specifically expressed at the later stages of flower bud development. The expression profiles of *JrASMT7/9/18/31/34/45* were also relatively similar, with all of these genes being significantly highly expressed at qS2 and weakly expressed at qS1 and qS3. Most of the differentially expressed *JrASMT* genes except for *JrASMT23* showed similar expression trends with RNA-seq data. *JrASMT7*, *JrASMT9*, and *JrASMT45* were highly expressed at qS2; these genes, which were not considered DEGs in the transcriptome, apparently played important roles during the morphological differentiation of the walnut bud.

## Discussion

### Identification of *ASMT* Gene Family Members in Walnut

As an indole-like molecular substance, melatonin is most well known for its ubiquitous regulation of circadian rhythms, immune systems, and antioxidant activities in living organisms (Zhao et al., 2019). With the study of melatonin synthesis, metabolism, and catabolism in higher plants, melatonin is considered a potentially pleiotropic phytohormone that regulates plant growth and development, life processes, redox dynamics, biotic and abiotic stresses, and fruit preservation (Back, 2021). Several studies have demonstrated that many melatonin synthases in plants interact directly with various proteins, including transcription factors (Wei et al., 2018). The formation of these protein complexes implies that melatonin synthases play an essential role in plant melatonin-mediated physiological and biochemical processes. ASMT is considered to be the key enzyme for melatonin synthesis.

This study identified 46 possible *ASMT* genes from walnuts based on published genomic data. Even though the selection criteria of hmmsearch and local BLAST were stringent and all genes were similarly validated for structural domains, the numbers of *ASMTs* in walnuts were still obviously higher than those in all currently identified species, including rice (19), Arabidopsis (19), tomato (13), pepper (16), wild mulberry (20), and apple (37). Some of the *JrASMTs* may have lost their functions. *SlASMT13*, which encodes only 103 amino acids in tomatoes, was determined to be a pseudogene (Liu et al., 2017). We observed the same presence of *JrASMT20* (103 aa) and *JrASMT46* (112 aa) genes encoding short peptides in walnuts. Despite our retention of these two members, whether they were pseudogenes similar to *SlASMT13* needs further confirmation. Another possible reason for such a large number of *JrASMTs* is the high degree of redundancy within the family; for example, *JrASMT31* on chromosome 10 and *JrASMT34* on chromosome 12 encode proteins with identical sequences, and *JrASMT8* and *JrASMT43* likewise share identical amino acid sequences. These functionally redundant genes increased the number of ASMT family members identified.

*JrASMT* genes contained relatively few exons; the simple gene structure confers a faster expression response of *ASMT* to ensure the realization of dynamic changes in melatonin content in plants. Most JrASMTs shared similar conserved motifs, except for members limited by the encoded protein length. The ASMT enzymes were more conserved among plants and localized in the cytoplasm, cytoskeleton, and chloroplast. Overall, these results were consistent with previous reports (Wang et al., 2022). Interestingly, an X-ray crystal structure analysis showed that the *Homo sapiens* ASMT enzyme only had a helical domain at the N-terminal end that interacted with multiple monomers (Botros et al., 2013). However, in terms of conserved motifs only, both the N-terminal and C-terminal motifs of JrASMT were significantly different, which provided a possibility for differences in the three-dimensional structure and substrate affinity between plant and animal ASMT enzymes. Although the crystal structure of the rice melatonin synthase OsTDC has been studied (Zhou et al., 2020), data from high-resolution X-ray or Cryo-transmission electron microscopy of plant ASMT enzymes are still lacking. CREs is an important regulator of gene expression, identification of CREs in the promoter region of *JrASMT* revealed that many elements are associated with light-response and hormone-response, consistent with the manner in which melatonin is predominantly induced in plants (Arnao and Hernández-Ruiz, 2021; Li et al., 2021; Tiwari et al., 2021; Yao et al., 2021). The promoter of *JrASMT20* was distributed with many ABA response elements ABRE, melatonin and ABA are both essential components of the phytohormone-mediated network (Zhang et al., 2014, 2017; Hu et al., 2021), *JrASMT20* was probably involved in the synergistic response of melatonin and ABA in walnuts. JA response elements were enriched in the promoter region of *JrASMT11* and *JrASMT12*. Melatonin and JA were considered to regulate together important plant physiological changes in biotic and abiotic stresses (Imran et al., 2021; Tiwari et al., 2021), these two genes may mediate the interaction between melatonin and JA. Two groups of *JrASMTs* were predicted to be separately targeted by a large number of miR172 and miR399 family members. miR172 regulation of meristem size, trichome initiation, stem elongation, branch meristem, and flowering capacity has been extensively characterized (Lian et al., 2021). miR399 plays an important role in plant resistance to various abiotic stresses (Li et al., 2020; Pegler et al., 2021). These results implied that miRNA172 and miR399 may antagonize the melatonin biosynthesis in walnuts under certain developmental stages or growth environments. This regulation is not mentioned in other studies (Liu et al., 2017; Wang et al., 2022).

### Expansion and Differentiation of *ASMT* Genes During the Evolution of Land Plants

Evolution is the source of all biological functions. Exploring the evolutionary trajectories of species at the molecular (DNA, RNA, protein) scale is a common approach in modern evolutionary biology. Previous research on the evolution of 62 *ASMT* genes in 13 species during plant terrestrialization showed that *ASMT* genes first appeared in primitive bacteria and expanded to embryophytes (Zhao et al., 2021). Our study increased the number of species and ASMT proteins identified. *ASMT* genes were found in both cyanobacteria and green algae at early evolutionary stages, with the difference that only one single-copy *ASMT* gene was found in cyanobacteria, whereas this number was apparently increased in green algae. Considering the more complex life system and significantly greater photosynthetic capacity of green algae (Khan et al., 2020), we infer that the ASMT gene family had already expanded before plants began terrestrialization to better regulate melatonin biosynthesis and better accommodate new environmental and biological functional requirements.

The number of *ASMT* gene copies in higher plants does not appear to be strongly correlated with evolutionary status or genome size, and the evolution of *ASMT* genes may have been influenced by a variety of complex factors, thereby perpetuating events such as gene duplication, loss, or mutation.

Significant divergence of *ASMT* genes has occurred within higher plants. A phylogenetic tree of 237 ASMT proteins provides direct evidence. Monocotyledonous and dicotyledonous clades are distributed on completely different evolutionary branches. All monocotyledonous phyla are in the same clade V(d), and dicotyledonous phyla are distributed in II–V(c) six different clades. This coincides with previous results for rice, Arabidopsis, tomato, and sorghum (Bhowal et al., 2021). The difference is that the introduction of a large number of ASMT proteins in our study classified dicotyledonous plant ASMTs into four subgroups more reliably, which was not suggested by other phylogenetic analyses of ASMT families. Considering that the functions of most ASMT proteins, including those in Arabidopsis, have not been characterized in detail, these ASMT proteins were crudely classified into subgroups II-V(c) based on their distant relationship with algal ASMTs. There are obvious evolutionary distance differences between different subgroup clades. Subgroups II–IV share the same internal node, and they maintain a very close relationship with algal ASMTs. Subgroup V(a) contains only perennial woody plant ASMT members. Taken together, these results undoubtedly show a high degree of sequence and functional differentiation of *ASMT* genes in Dictyostelium. Three algal ASMT proteins are in separate clades and at different evolutionary distances, further demonstrating that the differentiation of *ASMT* genes occurred early in biological evolution.

Plant evolution is usually accompanied by mutations and the loss of large segments of genes. Molecular evolution encounters immense background noises; identifying the evolutionary trajectory of genes among species is difficult (Williams et al., 2021). The orthology of ASMTs between different species was searched according to the distances between ASMTs. Although more or fewer orthologous genes existed between some species depending on the degree of species differentiation, in general, there were roughly four pairs orthologous *ASMTs* among dicots. The results of walnut collinearity analysis showed that these genes were also paralogous homologs, suggesting that these genes have maintained a high degree of collinearity among and within species in the evolution of the ASMT family. In contrast, only one *ASMT* gene is orthologous to these other genes in rice. Considering that the number of *ASMT* genes in both primitive bacteria and cyanobacteria was one, we believed that there was a single copy of *ASMT* genes in the ancient ancestor, which began to replicate and diverge in late algal evolution. Multiple whole-genome duplication events in higher plants caused chromosome duplication, rearrangements, and losses that expanded the number of *ASMT* genes, and the ancestral *ASMT* gene finally reached four copies in dicotyledons. These genes may represent the most conserved and typical functions of ASMTs. Both evolutionary trees and homology relationships suggest that monocot *ASMT* genes had fewer copies and were more conserved. This may be related to the evolution of the ASMT toward caffeic acid *O-*methyltransferase (*COMT*) during plant terrestrialization (Zhao et al., 2021). The reasons for this evolutionary drive playing a prominent role in monocotyledonous plants still need further investigation.

### Diversified Functions of *JrASMTs* in Walnut Flower Bud Development

Flowering in plants requires a complex series of physiological and biochemical processes, and several flowering pathways, such as photoperiod, vernalization, autonomous, and gibberellin, have been identified in Arabidopsis. The floral regulator gene Flowering locus T (*FT*) and the repressor of *FT*, Flowering locus C (*FLC*), are vital genes (Izawa, 2021). It has been demonstrated that melatonin participates in the flowering process in plants. High melatonin concentrations can inhibit flower opening in *Chenopodium rubrum* through the Photoinductive cycle (Kolář et al., 2003). The differential expression of *TDC* in herbaceous peony (*Paeonia lactiflora*) flowers during different developmental periods caused significant changes in melatonin accumulation (Zhao et al., 2018). *TDC*, *T5H*, and *COMT* were highly expressed in rice before and after flowering, and the melatonin content was significantly higher than that in other tissues (Park et al., 2013b). Exogenous melatonin administration (500 μM) delayed flowering time and increased the stability of two flowering repressors of the DELLA protein in Arabidopsis (Shi et al., 2016). Nevertheless, the mechanisms involved in melatonin regulation of floral developmental processes and flowering time are still poorly understood. On the one hand, melatonin plays multiple roles in plant floral organs, often responding to stress and pressure (Sharif et al., 2018). On the other hand, melatonin exhibits clear dose-dependent and interspecies differences during flower development. Different amounts and concentrations of exogenous hormones may lead to conflicting effects (Arnao and Hernández-Ruiz, 2020).

Similar to the results of other melatonin synthases in plant flowers (Zhao et al., 2018; Lee et al., 2019; Pan et al., 2021; Tsunoda et al., 2021), *JrASMT* genes were also participated in walnut flower development in this study. Coexpression analysis divided these genes into three modules. Four *ASMT* genes in the black module participated in early physiological differentiation. Among their coexpressed genes, *BUB3* is a core protein forming the spindle assembly checkpoint (SAC), which is associated with chromosome segregation in mitosis and meiosis (Komaki and Schnittger, 2017). Some components of the SAC promote flowering in Arabidopsis and have indirect interactions with *FLC* (Bao et al., 2014). *CYCB2* is highly expressed during the floral transition of the shoot apical meristem (SAM) in Arabidopsis (Klepikova et al., 2015). *NACK* regulates and controls cytoplasmic division within the mitogen-activated protein kinase (MAPK) pathway (Liang and Yang, 2019). These genes are evidently involved in floral developmental processes and cell fate, which coincides with melatonin-regulated rhythms. In addition, epistatic modifications may also be important for the involvement of *ASMT* in flower bud differentiation in walnut. *ADT* acts as a possible methylation target induced by vernalization to promote beet priming and flowering (Hébrard et al., 2013). *PRMT* promotes growth and flowering in cauliflower through asymmetric arginine methylation (Niu et al., 2007). Recent studies have revealed that Arabidopsis *PRTM* promotes *FT* by positively regulating flowering nuclear factor Ycs *via* physical interactions to promote *FT* transcript levels (Zhang et al., 2021).

The four *ASMT* genes in the dark-gray and brown modules are mainly engaged in the morphological differentiation of walnut flower buds. Among the coexpressed genes, *RD22* is a molecular marker of ABA signaling that is mediated by drought, salt, and other abiotic stresses (Harshavardhan et al., 2014; Matus et al., 2014). *HHP1* acts as a negative regulator of ABA in the crosstalk between cold stress and osmoregulation (Chen et al., 2010). *CDR* plays a role in mediating the expression of plant defense genes and enhancing disease resistance through salicylic acid (Ying et al., 2020). *LOG* converts nucleotide precursors of cytokinins into biologically active forms through the direct activation pathway. The content and location of cytokinins (CK) significantly impact plant flower development and sex differentiation (Kuroha et al., 2009; Ming et al., 2020). These results indicate that melatonin synergizes multiple hormonal regulatory networks during walnut flower development to influence and alter late developmental processes and resistance to multiple biotic-abiotic stresses in walnut flowers. The coexpression of several Receptor-like kinases (*RLKs*), generally considered receptor proteins for phytohormones and related to multiple stresses (Cui et al., 2022), provides further evidence for this. Some research have suggested interaction between melatonin and NO, GA, and SL (Mukherjee, 2019; Zhang et al., 2019b; Jahan et al., 2021). However, there is no similar direct evidence in our study. *TBL* is a component of plant cell wall polysaccharide acetylation (Gao et al., 2017). A recent genetic map identified a *JrTBL13* gene on chromosome 4 associated with the flowering date in male walnut flowers (Bernard et al., 2020). Interestingly, *TBL33* was coexpressed with *JrASMT40* in our study. Most of the genes coexpressed with *JrASMTs* still lack precise functional annotation, and no reliable homologs exist in Arabidopsis.

qRT–PCR results verified the expression of the core network of *JrASMT* genes during the development of walnut flower buds. Moreover, some *JrASMT* genes, which were found to not be differentially expressed, exhibited significantly high expression at specific floral developmental stages, *JrASMT18* and *JrASMT33* were, respectively, homologous with *SlASMT2* and *SlASMT12*, which were specifically expressed in tomato buds and flowers. In our results, *JrASMT18* was significantly highly expressed in qS2 period. In addition, *Vigun11g097000* was significantly associated with secondary metabolism changes during the *V. unguiculata* flowering period, and this gene was orthologs with *JrASMT42*. All these results certainly implying once again the complexity of the mechanisms involved in the regulation of plant flowering by melatonin. Overall, our study on the changes in melatonin synthase expression during germination and differentiation in a long-day flowering plant, walnut, provides a new perspective on the involvement of melatonin in plant flowering.

## Conclusion

In this study, 46 members of the ASMT family were identified in walnut. The genes were distributed heterogeneously and in clusters on 10 chromosomes of walnut and were named sequentially. The gene structure, conserved motifs, and phylogeny of all JrASMT proteins were analyzed. The evolution of the ASMT family was examined among different lineages of plants, and ASMT divergence began before the terrestrialization of plants. The presence of four homologous pairs of genes derived from a single copy of the *ASMT* gene in a distant ancestor is conserved within and among plants of the Dictyostelium phylum. A large number of light- and hormone-responsive elements are present in the promoter region of the *JrASMT* genes. Members with similar taxonomic status share a similar composition of CREs. RNA-seq data were also used in this study to explore the role of *JrASMT* in female flower bud development in walnut, and 13 *JrASMT* showed differential expression. WGCNA further suggested that *JrASMT1/10/13/23* were expressed in concert with cell cycle and epistatic modification genes at the stage of physiological differentiation of female flower buds. Similarly, *JrASMT12/28/37/40* were involved in phytohormone regulatory networks such as ABA, SA, and CK during flower bud morphological differentiation, regulating flower development and providing stress tolerance in walnut. Paraffin sections and qRT-PCR verified the expression of core network genes and provided other *JrASMT* members that may have potential functions. In conclusion, this analysis of the ASMT family, a key enzyme for melatonin synthesis, and its study in walnut flowering provides a theoretical basis for further understanding the mechanism of melatonin synthesis and action in higher plants.

## Data Availability Statement

The original contributions presented in the study are included in the article/Supplementary Material, further inquiries can be directed to the corresponding authors.

## Author Contributions

RX, LH, and LL performed the experiment and data analysis. RX, YZ, and KM drafted the manuscript. NL and JW designed the research and revised the manuscript. YX participated in the writing and revision of the manuscript. All authors contributed to the article and approved the submitted version.

## Funding

This study was supported by National Natural Science Foundation of China (31960584), Xinjiang Uygur Autonomous Region “Tianshan Youth” talent program (2020Q031), and The central government guided local scientific and technological development special funds in 2022.

## Conflict of Interest

The authors declare that the research was conducted in the absence of any commercial or financial relationships that could be construed as a potential conflict of interest.

## Publisher’s Note

All claims expressed in this article are solely those of the authors and do not necessarily represent those of their affiliated organizations, or those of the publisher, the editors and the reviewers. Any product that may be evaluated in this article, or claim that may be made by its manufacturer, is not guaranteed or endorsed by the publisher.

## Footnotes

^1^ https://plants.ensembl.org/

^2^ https://phytozome-next.jgi.doe.gov/

^3^ http://pfam.xfam.org/

^4^ http://rice.uga.edu/

^5^ https://www.arabidopsis.org/

^6^ http://smart.embl.de/

^7^ http://pfam.xfam.org/search

^8^ https://www.ncbi.nlm.nih.gov/cdd

^9^ https://web.expasy.org/protparam/

^10^ https://wolfpsort.hgc.jp/

^11^ https://itol.embl.de/

^12^ http://bioinformatics.psb.ugent.be/webtools/plantcare/html/

^13^ https://string-db.org/

^14^ https://www.pmiren.com/

## Supplementary Material

Supplementary File 1 Amino acid sequence of ASMT gene family in 25 plants.

Supplementary Figure 1 Sample clustering tree and trait correlation heat map.

Supplementary Figure 2 Direct interaction network diagram of JrASMT.

Supplementary Figure 3 Merged dynamic gene modules and genes tree.

Supplementary Figure 4 Scatter plot of correlation between module membership and gene significance in black, brown and dark grey.
