# Supplementary material for: Walnut N-Acetylserotonin Methyltransferase Gene Family Genome-Wide Identification and Diverse Functions Characterization During Flower Bud Development
Source: Front Plant Sci. 2022 Apr 15;13:861043. doi: 10.3389/fpls.2022.861043 (PMC9051526; doi:10.3389/fpls.2022.861043)
Supplement: Supplementary File 1 — Amino acid sequence of ASMT gene family in 25 plants. [file Data_Sheet_1.DOCX]

>AFZ51979

MEAKNKIFSIIQGFWGSQCLYIATCLGIPNLLEEYGAQSVESLAEKTETHTETLYVVLRGLAHLEILEEKPDRVFAPTEASSLLVTNAGPSIGHFALHITEPCQWDGWKELYDAVHTGEVAFERANGKGVYEFTRDNPWSGDVFINAMSFLTDHATEALMAVYDFGQYETVMDVGGGQGGLIAEIVKTYNCKGMLFDVPYVIETAPSFLESRGVPKDAIALHTGDVFEKVPTGADAIVMKYFLSAWNDEDAGKILARCREALPDHGKIVLLQSIVPDVGEPTVCPDGIMPGLFAVQIRSAVPGGVWRTLKQYQEIFANSGFKLDRVVHTSTNLSAMEFSLA

>maker-Fvb5-3-augustus-gene-182.54|maker-Fvb5-3-augustus-gene-182.54-mRNA-1|Fx_ananassa_v1.0.a1

MLGFADSMALKSAVELRIPDIINSHGQALSLSEIVSNIDSKSPSPDITCLSRIMRLLVHRNIFAAHHDDGDSGETLYGLTPSSRWLLQDSELTLAPMVLLQTDPILMAPMQYFSQCVKQGGTHAFSKAHGRDIWQFFSENLEANQLFNDAMACSANIIMKVILARCKGAFDDVTTLVDVGGGTGRAAAEIVKAYPSIKAINFDLPYVVATAPAYHGVSHVGGDMFDEGNIPNADAIFMKWIMHDWSNGDCIKILKNCRKAIPERSGKVIIVDAVLEPNGDGLFDDTGLVFDLLMITHSSGGKERSESEWKQMLEQAGFPRYNIINIPAVVSIIEAYPV*

>maker-Fvb6-2-augustus-gene-322.43|maker-Fvb6-2-augustus-gene-322.43-mRNA-1|Fx_ananassa_v1.0.a1

MEAAAELDEASLRGQADVWKYMLGFADSMALRSAVELRIPDIIHSHGRALTLSEIASSFDSASPSPDISCLARIMRLLVRRNIFTAHHDGEDSEKTGLYGLTHSSRWLLHDSELTLTPMVLMETNPTLLAPWHYFSQCVKQRGPYAFERAHGSDIWQFFSENLKFNRLFNDGMACTAKITMKAILAGYKGGFEDVATLVDVGGGTGSAVAEIVKAYPSVKGINFDLPHVVATAPLYHGVSHVGGDMFDEGSIPKADAIFMKWVMHDWSDSDCVKIFKNCRNVIPERTGKVIIVDVILEPNGVGMFDDTGLVFDLLMIAHTSGGKERTESEWKKMLEQAGFPRLKIIKIPALMLIIEAYRV*

>maker-Fvb6-3-augustus-gene-174.48|maker-Fvb6-3-augustus-gene-174.48-mRNA-1|Fx_ananassa_v1.0.a1

MERLNSFRHVNQKWSNGDNSNELLHAQAHIWNHIFNFINSMSLKSAIQLGIPDIINKHGRPMTLSELTSALPINPTKSHSIYRLMRILIHSGFFAKKKLSKSDEEGYVLTDASQLLLKDHPLSMTPFLNAMLDPVLTKPWHYLSTWFQNDDPTPFDTAHGMTFWDYGYHQPSIAHFFNDAMASDARLVTSVIVDECRGVFEGLDSLVDVGGGTGTVAKAIADAFPHIECTVLDLPHVVADLQGSKNLKYTGGDMFEAVPPADAVLLKWILHDWNDEECVKILERSKEAITSKDKKGKVIIIDMMMENQKGDEESMETQLFFDMLMMALLTGKERNEKEWSKLFTDAGFGDYKITPILGLRSLIEVYPK*

>maker-Fvb6-3-augustus-gene-47.29|maker-Fvb6-3-augustus-gene-47.29-mRNA-1|Fx_ananassa_v1.0.a1

MLGFADSMALRSAVELRIPDIIHSHGRALTLSEIASSFDSASPSPDISCLARVMRLLVRRNIFTAHHDGEDSEKTFLYGLTHSSRWLLHDSELTLAPMVLMETNPTLMAPWHCFSQCVKQRGPCTFKRAHGLDIWQFFYENLKFNRLFNDGMACTAKITIKAILAGYKGGFEDVATLVDVGGGTGSAVAEIVKAYPSIKGINFDLPHVVATAPVYHGVSHVGGDMFDEGSIPNADAIFMKWVMHDWSDSDCVKILKNCRNAIPERTGKVIIVDVILETNGVGMLDDTGLVFDLLMIARTSGGKERTESEWKKMLEQAGFPRLKIIKIPALMLIIEAYPV*

>maker-Fvb6-4-augustus-gene-323.29|maker-Fvb6-4-augustus-gene-323.29-mRNA-1|Fx_ananassa_v1.0.a1

MEAAAELEEASLRGQADVWKYMLGFADSMALRSAVELRIPDIIHSHGRALTLSEIASSFDSASPSPDISCLARIMRLLVRRNIFTAHHDGEDSEKTVLYGLTHSSRWILHDSELTLAPMVLMETNPTLMAPWHCFSQCVKQRGPCAFKRTHGHDIWQFFSENLKFNRLFNDGMACTAKITMKAILAGYKGGFEDVATLVDVGGGTGSAVAEIVKAYPSIKGINFDLPHVVATAPVYHGVSHVGGDMFDEGSIPNADAIFMKWVMHDWSDSDCVKILKNCRNVIPERTGKVIIVDVILEPNGVGMFDDTGLVFDLLMIAHTSGGKERTESEWKKMLEQAGFPRLKIIKIPALMLIIEAYPV*

>maker-Fvb6-1-augustus-gene-179.34|maker-Fvb6-1-augustus-gene-179.34-mRNA-1|Fx_ananassa_v1.0.a1

MFSQVYKGIKDSDKPSLIKQALMSSLANNGVASATASSTDELIVQSQLHVRNHILQLMNSMVLNCAIQLGIPDIIHNHAQPITLSDLTSALNVHPSKSRFLYRLMCILVQQGFFTKHNDVQHGIVYSLTPSSKLLLKDGLMTTFLLLLLDPLLTSPWHLLGTWFQNCASTPFEMAHGMSFFDLVAHEPVFGNMFNEAMVADSKLVSRAVVKECQGVFEGLKSLVDVGGGKGTMASAIADAFPQIKCTVLDLPHVIENLKGSNNLDFIGGNMFEQIPPANAILLKWILHDWNDEESVEILKRCRDAIPSKSDGGKVIIIEMVVTVDDDKKMNNKSTETQLFWDMLMMVCLTGRERTEKDWEKLFLAAGFSHYKITHTLGIRSFIEVYP*

>maker-Fvb6-1-augustus-gene-179.41|maker-Fvb6-1-augustus-gene-179.41-mRNA-1|Fx_ananassa_v1.0.a1

MERLNSFRHLNQKWSNGEHSNELLHAQAHIWNHIFSFINSMSLKSAIQLGIPDIINKHGRPMTLSELTSALPINPTKSHSIYRLMRILIHSGFFAKKKLSKSDEEGYVLTDSSQLLLKDHPLSITPFLNAMLDPVLTKPWHYFSTWFQNDDPTPFDTAHGMTFWDYGNHQPSIAHFFNDAMASDARLVTSVIVDECRGVFEGIDSLVDVGGGTGTVAKAIADAFPHIKCTVLDLPHVVADLQGSKNLKYTGGDMFEAVPPADAVLLKWILHDWNDEECVKILERSKEAITGKDKKGKVIIIDMMMENQKGDEESMETQLFFDMLMMALVTGKERNEKEWSKLFTDAGFSDYKITPILGLRSLIEVYP*

>maker-Fvb2-2-augustus-gene-106.22|maker-Fvb2-2-augustus-gene-106.22-mRNA-1|Fx_ananassa_v1.0.a1

MEHTQKELILEQKEEELAKVEMWKYVLGFSKIAVVKCAIELGIADAIESHGSAMTLSEIAATLKCDPSSLYRIMRFLVHHQIFKEVQPKIQLGPRSYAQTPLSRCLLKSGKTSMAALILLEASPVMLEPWHGLSAGVQGDGMSTPAFEAVHGEDVWSFAAANPGHSELINEAMACDARLAVPAVIESCIEVFHGIESIVDVGGGDGTTLSLLVKACPWITRGINFDLPHVVSVAKESDRVENVGGDMFDCIPKADAAIIKSVLHDWGDEECICILRNCREAIPKDKGKVIILEAIIEEDELEEDELTHVRLMLDMVMMAHTNTGKERTLKEWGYVLQEAGFSRHTVTPISAVQSVIQAFL*

>maker-Fvb2-2-augustus-gene-105.52|maker-Fvb2-2-augustus-gene-105.52-mRNA-1|Fx_ananassa_v1.0.a1

MEHTQRELILEQKEEELAKVEVWKYVFGFSKIAVVKCAIELGIADAIESHGSAMTLSELAATLKCDPSSLYRIMRFLVHHQIFKEVQPKIQLGPRSYAQTPLSRCLLKSGKNSMAAFILLETSPVMLEPWHGLSARVQGNGMSTPAFEAVHGEDVWSFAAANPGHSELINEAMACDARLAVPAVIESCIEVFHGIESIVDVGGGDGTTLSLLVKACPWITRGINFDLPHVVSVAKESDRVENVGGDMFDCIPKADAAIIKWVLHDWGDEECICILRNCREAIPKDKGKVIILEAIIEEDELEEDELTHVRLMLDMVMMAHTNTGKERTLKEWGYVLQEAGFSRHTVTPISAVQSVIQAFL*

>maker-Fvb5-2-augustus-gene-98.40|maker-Fvb5-2-augustus-gene-98.40-mRNA-1|Fx_ananassa_v1.0.a1

MEAVTELDEVTLRGQADVWKYMLGFADSMALKSAVELRIPDIINSHGQALSLSEIVSNIDSKSPSPDITCLSRIMRLLVRRNIFAAHHDDGDSGETLYGLTPSSRWLLQDSELTLAPMVLLQTDPILMAPMQYFSQCVKQGGTHAFSKAHGRDIWQFFSENLEANQLFNDAMACSTNIIMKVILARCKGAFDDVTTLVDVGGGTGRAAAEIVKAYPSIKAINFDLPYVVATAPAYHGVSHVGGDMFDEGNIPNADAIFMKWIMHDWSDGDCIKILKNCRKAIPERSGKVIIVDAVLEPNGDGLFDDTGLVFDLLMITHSSGGKERSESEWKQMLEQAGFPRYNIINIPAVVSIIEAYPV*

>Gohir.A03G158200|Gohir.A03G158200.2|Ghirsutum_v2.1

MSSQESGREKEIIELDEARLQGQAEIWRYMFSFADSMALKSAVELRIADIIHSHGVAITLSQIASCINGCLTSPDITTLARIMRLLVRRKIFTVHHPLDGGDPLYDLTHSSRWLLHDFEQTLAPMVLMENHPWLIAPWHCFSQCVKEGGIAFKKAHGREIWDLTSGNPEFNKLFNDGLASTSKVVTSAILSGYKQGLSSIESLVDVGGGIGGLISEIVKAYPHIKGVNFDLPHVILAAPAYNEISHVGGDMFHVIPNANAVIMKWVLHDWGDEDCIKILKNCRKAIPRENGKVIIVEVVVKAEGSGVFDDMGFIFDLLMIAHSSGGKERTEVEWKKILEEGGFSRYKIIDIPALPSIIEAYPDDQ*

>Gohir.A03G158100|Gohir.A03G158100.1|Ghirsutum_v2.1

MERTELDEARLQGQAEIWRYMFSFADSMALKSAVELRIADIIYSYGGAATLSQIASCINDGLTSPDITTLARIMRLLVRRKIFTIHHPSDGRDSLYNLTHSSRWLLHDSEQSLAPMVLMENHPWQMAPWRYFSQCVKEGGDAFKKAHGCEIWDLASRNPDFNKLFNDGLACTSKVVTSAILSGYKQGFNSIGSLVDVGGGTGGLISEIIKVYPHIKCVNFDLPHVVSTAPAYNGVSHIGGDMFHAIPNTDAIIMKWILHDWDDEDCIKILKNCKKAIPKENGKVIIIEIILKEDGSGVFDEIGFILDLAMIAHTNGKERTEVEWKKILEGGGFFHYKIINIPSLLSIIEAYPDDK*

>Gohir.A03G158000|Gohir.A03G158000.1|Ghirsutum_v2.1

MERIELDEAMLQGQAEIWRYMYSFADSMALKSAVELRIADIIHSNGGPVTLSQIASCINGGLASPDITTLARIMRLLIRRKIFTVHPSDGGDLLYDLTHSSRWLLHDSEQTLAPMVLMNTHPWQMTPWHYFSQCVKEGGIAFKKAYGCELWDLASRDPDLNKLFNNGLACSSKVITSVILSSYKQGLSSIGSLVDVGGGIGGLISEIVKAYPHIKGVNFDLPHVVSTAPTYNAVSHIGGDMFHDAIPNADAVIMKWILHDWTDEECIKILRNCKKAIPRENGKVILVEIILKEDGSGVFDDIGFVMDLVMIAHTNGKERTEAEWKKILGGGGFSHYKIINIPALFSIIEAYPDAQ*

>Gohir.A11G293300|Gohir.A11G293300.1|Ghirsutum_v2.1

MERIELDEAMLQGQAEIWRYLYSFADSMALKCAVELRIADIIHSNGGPLTLSQIASCINGGLTSPDITTLARIMRLLIRRKIFTVHHPSDGGDLLYDLTHSSRWLLHDSEQTLVPMVLMENHPLQMAPWHYFSQCVKEGGFAFKKAHGCEIWDLASRNPDFNKLFNDGLACTSKFLTSVILSSYKQGLSSIGSLVDVGGGTGGLISEIVKKHPHIKGINFDFPHVVSTAPTYDGVSHIGGDLFHAIPNADAVILKWILHCWGDEDCIKILRNCRKAIPRENGKVIIVEIILKEDGSGVFDEIGFLLDLVMIAHANGKERTEAEWKKILEGGGFSHYKIINIPALASIIEAYPDA*

>Gohir.A04G132406|Gohir.A04G132406.1|Ghirsutum_v2.1

MEVSGIEEEEARPEVDIWNYVFGYAKIAVVKCAIELGIADVIENYGSPMPLSELATALRCDPSRLHRIMRFMVHYRIFKREPINQHTVGFSSTPLSRCLIKGGEKSMAAFILFMSSPPWLASWHCLNSRVLETSNNISPFEAANGKDLWSYTETNPYHNEVFNEAMACVARLTVQAIIKGCPEVFDGVKSLVDVGGGINFDLPHVVAVAPKFGSIENVGGDMFMSIPNVDAAFLMWILHDWDDEECIKILKKCREASLEDKGKVIIVEAVLEKDKEVDELGYLGLMLDMAMMAHTDKGKERTLKEWSYVLRQSGFTRFNVKPIRAVQFIIEAYP*

>Gohir.A04G132300|Gohir.A04G132300.1|Ghirsutum_v2.1

MGDMEVTIKEEARAEIKIWNYVFGYAKIAAVKCAIELGIADVIENYGSPMPLSELASALRCEPSRLHRIMRLMVHDRIFKQEPINQHTVGFSSTPLSRCLIKGGEKSMAAFILLMSGPTCLAPWHSLSARVLETDHNISPFEVANGKDIWSYAEANPDFSELFNNAMGCKARLTVQATIEGCPEVFDGVENLVDIGGGNATALSLLVKAFPWIRGINFDLPHVVAVAPKSDSIENVGGDMFMSIPNADAAFLMSVLHDWDDEECIKILKKCREAIPEDKGKVIIVEAVLEEDKEVDEVGVVGLMLDTAMMAITNKGKERTLKEWSYVLRQSGFTRFNVKPIRAVQSVIKAYP*

>Gohir.A04G132350|Gohir.A04G132350.1|Ghirsutum_v2.1

MEVTIKEEEARAEVEIWNYVLGYVKIAVVKCAVELGISDVIENYGSPMPLSELATALQCEPSRLHRIMRIMVHYRIFKQEPINQYTVGFSSTPLSRRLIKGGEKSMAAFILLMYSPTCLASWHCLSARVLETGNNISPFEVANGKDIWSYAEANHDFSELFNNAMGCDARLTVQATTEGCPEVFDGVESLVDVGGGNRTALSLLVNAFPWIRGINFDLPHVVTVAPKSDSIENVGGDMFMSIPNVDAAFLMWVLHDWDDEECIKILKKCREAIPEDKGKVIIVEAVLEEDKEGNELGVVGLMLDTTMMAITNKGK*

>Gohir.A04G132400|Gohir.A04G132400.1|Ghirsutum_v2.1

MGDKEMGDKEVTIKEEARAEIKIWNYVFGYAKIAAVKCTIELGIADVIENYGSPMPLSELATALRCEPSRLHRIMRLMVHDRIFKQEPINQHTVGFSSTPLSRRLIKGGEKSMAAFILLMSGPTCLAPWHSLSARVLETGNNISPFEVANGKDLWSYTEANPDFRELLNNSMGCKARLTVQATIEGCPEVFDGVESLVDVGGGNGTALSLFVKAFPWIRGINFDLPHVVAVAEKSDGIENVGGDMFMSIPKADAAFLMWVLHDWDDEECIKILKKCREAIPEDKGKVIIVEAVLEEDKEGDELGVARLMLDTAMMVITNKGRERTLKEWSYVLRQSGFKRFNVKPIRAVETVIETVIEAYP*

>Gohir.A04G132200|Gohir.A04G132200.1|Ghirsutum_v2.1

MGDMEVTRKEEARAEIKIWNYMLGYVKIAVVKCAIELGIADVIENYGSPMPLSELATALRCEPSHLHRIMRFMVHYRIFKQEHINHHTVGFSSTPLSSLLIKGGEKSMVAFILFVSSPTWLAPWHSLSARVLETGNDISPFEVANGKDLWSYAEANPNFSELFNNAMGCDARLTVQATIEGCPEVFDGVKSLVDVGGGNGTALSLLVKAFPWIRGINFDLPHVVAVAPKSDSIENVGGDMFMSIPNADAAFLMWVLHDWDDEECIKILKKCREAIPEDKGKVIIVEAVLEEDKEGDELGAVGLMLDMVMMAITNKRKERTLKEWSYVLQQSGFTRFNVKPIRAVKFVIEAYP*

>Gohir.A12G035600|Gohir.A12G035600.1|Ghirsutum_v2.1

MGDIKVRGKKEDEDEKAEVDIWNYVFGYVKIEVVKCAIELGIADAIDKHGSPMTLSQLTTTLKCEPPRLYRIMRFLVHYQIFKEEPVTQDSFGFALTPLSRRLIRHGERSMAAFILLESSPVMLAPWHSLSARVLDSGNSPFETAHGKDVWSYAEENPRHSKLIDEAMACDARVAVRALIEGCPQVFDGIKSLVDVGGGNGTALSMLVKEFPWMHGINFDLPHVVAVAPKVDGIEYVGGDMFECVPKADAAFFLWVLHDWDDEECIQILKKCREAIPQDKGKVIIVEAVLEEDKNDKLEFVGLMLDMVMMAHTKKGKERTLKEWKYVLGEAGFTRINVKPIHAVQFIIEAYI*

>Gohir.A12G035700|Gohir.A12G035700.1|Ghirsutum_v2.1

MGDVKVRGKKEDEDEKAEVDIWNYVFGYVKIAVVKCAIELGIADAIDKHGSPMTLSQLTTTLKCQPSRLYRIMRFLVHYQIFKEEPITKDSIGFALTPLSRRLSRHGERSMAAMILLQSSPVTLATWHSLSARVLDSGNSPFETAHGKDVWSYAEENPRHSKLIDEAMACDARVAVRAIIEGCPQVFDGIKSLVDVGGGNGTALSMLVKAFPWIHGIDFDLPRVVAVAPKVDGIKYVGGDMFECVPKADAAFFMWVLHDWGDEECIQILKKCREAIPQDKGKVIIVESVLEEDENDKLEFVGLMLDMVMMTHTNKGKERTLKEWKYVLGEAGFTRIDVKPIHAVQSVIEAYI*

>Gohir.D04G169000|Gohir.D04G169000.1|Ghirsutum_v2.1

MEVSGIEEEEARAEVDIWNYVFGYAKIAVVKCAIELGIANVIENYGSPMSLSELATALRCEPSRLYRIMRFMVHDRIFKQEPINQHTVGFSSTPLSRCLLKGGEKSMAAFILLMSSPTCLAPWHSLSARVLETGNNISPFEVANGKDLWSYTEANPDFRELFNNAMGCDARLTVQATIEGCPEVFNGVESLVDVGGGNGTALSLLVKAFPWIRGINFDLPHVVAVAAKSDSIENVGGDMFMSIPNADAVFLMWVLHDWDDEECIEILKKCREAIPEDKGKVIIVEAVLEEDKEGDELGGVGLMLDTALMAITNKGKERTLKEWSYVLRQSGFTRFNVKPTRAVQFVIEAYP*

>Gohir.D04G168800|Gohir.D04G168800.1|Ghirsutum_v2.1

MGDMEVTVKEEARAEIKIWNYVLGYAKIAVVKCAIELGIADTIENYGSPMPLSELATALRCEPSRLHRIMRFMVHDRIFKQEHINHHTVGFSSTPLSRLLIKRGEKSMAALILFVSSPTWLAPWHSLSARVLETGNNISPFEVANGKDLWSYAEANPDFRELFNNAMGCDARLTVQATIEGCPEVFDGVESLVDVGGGNGTALSLLVKAFPWIRGINFDLPHVVAVSAKSDSIENVGGDMFMSIPNADAAFFMWVLHDWDDEECIKILKKCREAIPEDKGKVIIVEAVLEEDKEGDELGAVGLMLDMTMMAITNKGKERTLKEWSYVLRQSGFTRFNVKPIRAVQSVIEAYP*

>Gohir.D04G169300|Gohir.D04G169300.1|Ghirsutum_v2.1

MGDMEVTIKEEARAEIKIWNYVLGYVKITVVKCAIELGIADVIENYGSPMPLSELATALRCEPSRLHRIMRFMVHDRIFKQEPINQHTIGFSSTPLSRLLIKGGEKSMAAFILLMSGPACLATWHSLSARVLETGNNISPFEVANGKDLWSYAEANPDFRELFNNAMGCHARLTVHATIEGCPEVFNGVESLVDVGGGNGTALSLLVKAFPWIRGINFDLPHVVAVAATSDSIENVGGDMFMSIPNADAAFLMCVLHDWDDEECIKILKKCREAIPEDKGKVVIVEAVLEEDKEDDEIGGVGLMLDTALMAITNKGKERTLKEWSYVLRQSGFTRFNVKPICAVRSVIEAYP*

>Gohir.D04G169100|Gohir.D04G169100.1|Ghirsutum_v2.1

MEVTIKEEVRAEIKIWNYVLGYVKIAVVKCAIELGIADVIENYGSPMPLSELATALRCEPSRLHRIMRFMVHDRIFKQEPINQHTVGFSSTPLSRLLMKGGQKSMAALILLMSSPTCLAPWHSLSARVLETGNNISPFEVANGKDLWSYAEANPDFRELFNNAMGCDARLTVQATIEGCPEVFDGVESLVDVGGGNGTALSLLVKAFPWIRGINFDLPHVVAVAAKSDSIENVGGDMFMSIPNADAAFLVWVLHDWDDEECIKILKKCREAIPEDKGKVIIVEAVLEEDKEGDELGGVGC*

>Gohir.D04G168900|Gohir.D04G168900.1|Ghirsutum_v2.1

MGDMEVTVKEEARAEIKIWNYVLGYAKIAVVKCAIELGIADTIENYGSPMPLSELATALRCEPSRLHRIMSFMVHDRIFKQEPINQHTVGFSSTPLSRLLIKRGEKSMAAFILLMSSPTCLAPWHSLSARVLETGNNISPFEVANGKDIWSYAEANPDFRELFSNAMGCDARLTVQATIEGCPEVFDGVESLVDVGGGNGTALSLLVKAFPWIRGINFDLPHVVAVAAKSDSIENVGGDMFMSIPNADAVFLMWVLHDWDDEECIKILKKCREAIPEDKGKVIIVEAVLEEDKEGDELGGVGLMLDTALMAITNKGKERTLKEWSYVLRQSGFTRFNVKPTRAVQFVIEAYP*

>Gohir.D12G037100|Gohir.D12G037100.1|Ghirsutum_v2.1

MGDIKVRGKKEDEDEKAEVEIWNYVFGHVKIAVVKCAIELGIADAIDKHGSPMTLSQLTTTLKCEPPRLYRILRFLVHYQILKEEPVTQDSIGFALTPLSRRLIRHGERSMAALILLESSPVMLAPWHSLSARVLDSGNSPFETAHGKDVWSYAEENPGHSKLIDEAMACDARVAVRALIEGCPRVFDGIKSLVDVGGGNGTALSMLVKAFPWMHGINFDLPHVVAVAPKVDGIEYVGGDMFECVPKADAAFFMWVLHDWDDEECIQILKKCREAIPQDKGKVIIVESVLEEDENDKLEFVGLMLDMVMMAHTNKGKERTLKEWKYVLGEAGFTRINVKPIHAVQSIIEAYI*

>Gohir.D12G036900|Gohir.D12G036900.1|Ghirsutum_v2.1

MGDIKVRGKKEDEDEKAEVEIWNYVFGHVKIAVVKCAIELGIADAIDKHGSPMTLSQLTTTLKCQPSRLYRIMRFLVHYQIFKEEPITKDSIGFALTPLSRRLSRHGERSMAAMILLQSSPVTLAPWHSLSARVLDSGNSPFETAHGKDVWSYAEENSGHSKLIDEAMACDARVAVRAIIEGCPQVFDGIKSLVDVGGGNGTALSMLVKAFPWIHGIDFDLPRVVAVAPKVDGIEYVGGDMFECVPKADAAFFMWVLHDWGDEECIQILKKCREAIPQDKGKVIIVESVLEEDENDKLEFVGLMLDMVMMTHTNKGKERTLKEWTYVLGQAGFTRIDVKPIHAVQSVIEAYI*

>Gohir.D02G181200|Gohir.D02G181200.1|Ghirsutum_v2.1

MERIELDEAMLQGQAEIWRYLYSFADFMALTSAVELRIADIIHSNGGAATLSQIASCISDGLTSPDITALARIMRLLVRRKIFTIHPPLDGGDPLYNLTHSSRWLLHDSEQTLAPMVLMENHPWQMAPWHYFSQCVKEGGTAFKKAHGCEIWDLTSRDPNFNKLFNDGLACTSKFITSAILSGYKQGFNSIGSLVDVGGGTGGLISEIVKVYPHIKGVNFDLPHVVSTAPAYNGVSHIGGDMFNAIPNTDAIIIKWVLHNWNDEECIKILRNCKKAIPRENGKVIIVEIILKEDGSGVFDDIGFVMDLVMFAHNSGKERTEAEWKKILEGGGFSHYKIINIPAMVSIIEAYPDAQ*

>Gohir.D02G181400|Gohir.D02G181400.1|Ghirsutum_v2.1

MSSQESGREKETIELDEARLQGQAEIWRYMFSFADSMALKSAVELRIADIIHSHGVAITLSKIASCINGSLTSPDTTTLARIMRLLVRRKIFTVHHPSDGGDPLYDLTHSSRWLLHDSEQTLAPMVLMENHPWLIAPWHCFSQCVKERGIAFKKAHGREIWDLASGNPEFNKLFNDGLACTSKVVTSAILSGYKQGLSSIESLVDVGGGIGGLISEIVKAYPHIKGVNFDLPHVVLAAPAYNGIFHVGGDMFHAIPNANAVIMKWVLHDWGDEDCIKILRNCRKAIPRENGKVIIVEVVVKAEGSGVFDDMGFIFDLLMIAHSSGGKERTEVEWKKILEEGGFSRYKIIDIPALPSIIEAYPDDQ*

>Gohir.D02G181300|Gohir.D02G181300.1|Ghirsutum_v2.1

MERIELDEAMLQGQAEIWRYLYSFADSMALKSAVELRIADIIHSNGGAATLSQIASCINDSLTSPNITTLARIMRLLVRRKIFTIHPPSDGGDPLYDLTHSSRWLLYDSEQTLVPMVLMENHPWQMAPWHYFSQCVKEGGVAFKKAHGCETWDLASRNPDFNKLFNDGLACTSKFITSAILSGYKQGFNSIGSLVDVGGGTGGLISEIVKVYPHIKGVNFDLPHVVSTAPAYNGVSHIGGDMFHAIPNTDAIIMKWILHDWTDEECIKILRNCKKAIPRENGKVIIVEIILKEDGSGVFDDIGFVMDLVMFAHTNGKERTEAEWKKILEGGGFSHYKIINIPALVSIIEAYPDAQ*

>Gohir.D10G206300|Gohir.D10G206300.1|Ghirsutum_v2.1

MDMVNANGEHVNELLQAQAHVWNHIFNFINSMSLKCAIDLGIPDIIQNHGKPMTITELVAALPMLNPTKACNIYRLMRILVHSGFFAQRNLCNDAQEDGYVLTNASRLLLKDNPLSVTPFLKAMLDPILTEPWHCLGTWFKNNDHTPFDTTHGKTFWDYAGHDQKLNSLFNEGMASDARLVNSILIDKCKKVFEGLNSLVDVGGGTGTLSKAIVDAFPHLECIVLDLPHVVANLQDSGNLKYVAGDMFKEIPASDAILLKWILHDWNDDECLKILKRCKEAISRQNKKGGKVMIIDMVLMKNEKMNGEAFNSTETQLFFDMLMMVLVTGKERQEEEWAKLFFAAGFSNYKITPILGLRSLIQVFP*

>HORVU2Hr1G004710|HORVU2Hr1G004710.1|Hvulgare_r1

MTLKLLAEVSPQELLEALAGLQNHLLGYIKSMSLRCVVDLGIPDAIHCRGGTATLADIVTDTKVHPGKVADLQRVMKLLTTSGIFTATPNAGDDDDTMVYGLTTACRILVGWCNLSPIVPFLVNPLVVSSFFSMPAWFRTEPEAAGAGSLFELAHGCSQWEMVSKDAGFKNVLNNSMAADSQVFLEVIIVDKGRIFRGLRSLVDVGGGNGAGTQVIAKAFPRIKCTVMDLPHVVVSGQAAAGDDILSFVAGDMFQSIPSADAVLLKNILHDWGHDDCVKILKHCKEAIPARNAGGKVIIIDMVRGSANGDRKINEMEAIQNLFMMYITGVERNEIEWKMIFSDAGFSDDYKILPILGPYSVIEIYP*

>HORVU2Hr1G007720|HORVU2Hr1G007720.1|Hvulgare_r1

LSPRRGKTELALAMAAEAPTVEVSEEDGELLRAQADLWRHSFGYVTSMGLRCAVKLGVPTAIHHLGGVTSLPDLTTALSLPPSKMPYLRRLMRLLVSTGVFAKAESSSEEGPELFRLNPLSKILVDGDGDEHHGHTSLVLAATSSQYLEASLGLAEWFKKDFGEAPVPSPFEDVHGVPLLDERTASLDKEFDELVNDAVAAHDNLGIDMIVRECHYLFKGLESVTDCCGGDGTTARAIIDAHPHLKCNVLDLPKVIEKAPAHDIINYVAGDMFQAIPPAQAVMLKFVLHNWNDEDCVNILDQCKKATPSREEGGKVIIIDILVNPSLEPIIYESQMLFDVGMMTLTKGRHRDENDWGTLFMKAGFSEYKIVKKLGDRVVFEVYP*

>HORVU3Hr1G007570|HORVU3Hr1G007570.4|Hvulgare_r1

MTTPTDAELVQAQADLWHHCLSYLTPMALRCAVQLGIPTAIHRLGGAASLPDLVTALSLPTSKVPFVGRLLRLLAATGILASNEAGMYSLVPLSYLLVDGVRIDGDASQTAFVLTVTSRHFMEAAIGLADWFKNDIPPPVPSPFEDVHGAALFEESMAALDPECDKLFNESLAAHDHMGIGTILRECHGIFNGLQSLTDCCGGDGTTARAIVKAYPHIKCNIFPCPLVLHFWSDEDCINILAQCKKAIPTREAGGKVIIIDIVVDSSSGKMFETQLLMDMAMMVYTRGRQRDEQDWNAIFVKAGFSDYKIVKKLGARGVIEVYP*

>HORVU3Hr1G029150|HORVU3Hr1G029150.1|Hvulgare_r1

MATQAQQLAVPTEAELLQGQADLWRHSLYYMTSMAFQCAVKLGIPTTIHSLGGAASLPDLVAALSLPPAKLPYLRRIMRLLATSGVFAADSAAADVVATYRLTPLSWLLLDGVAVDGHPSQTSLVLAATSRHCLEAAMGLSDWFKKDVAASPFQDLHGVTLFDGTMAEQDPEVDAVFNDALASHDNSGFLAVLRECGGTLFQGLESLTDCCGGDGTTARAIVEAFPQIKCTVLDLPRVIDNVPADGVVNYVAGDMFNSVPFAQAVLIKLVLHHWSDEDCVRILAQCKKAIPPREEGGKVIVIDIVVDSSSGPTHEAELLMDVAMMVMTNGRQRDETDWSEIFIKAGFSDYKVVKKLGARGVFEVYP*

>HORVU3Hr1G026150|HORVU3Hr1G026150.1|Hvulgare_r1

ACFAVGSSSLLIHVNVLGSLYIRACLITLGCTYTPCDTVQLKTTMATFSNEELLQAHAELWNLTFGYLKSMALDCAIKLGLPNAIHRCSGAATLPDLLDAISVPESKKAHLPRLMRFLAAFGIFTVNLPAAGECGDGEYGLTPMSRLLVDDAGANGSCGSLSPLVLSQTTKYHVKAALHLPEWFMSDDGAAAAETPFRMAHGTDLWGVMDRDPTMNQVFNASMGSDIRFAMDFVVSNYGDVFEGVTSLVDVGGGTGSAARAIAKAFPHVKCSVLDLPNVVNSIPSDGVVEYISGDMMSSIPTTDAVFLKYVLHDWNDEDCVKILTQCKKAIPKSGGKVIIIDIVVGSPLKAMLEAQASFDLLMMVIAAGKERDEHEWRKIFMDAGFSRYKTRPVLGFMSITELYA*

>HORVU3Hr1G110170|HORVU3Hr1G110170.3|Hvulgare_r1

QNIMAENHQAVKMPTSSNKELLQAHAELWNLTFSYLKSMALECAIKLGIPTAIHRCGGTASLTDLLAALSIPESKKSYLPRLMRFLAASGIFTIDFLATGECANAGATVTYRLTPLSHLLVDGDDTYAHQCTSLSPFVLAQTNKYHVTAAMHFSDWFTSDEGSASAEMPYKMAHGTDHWEIMARDPKLNQVFNAGMAADTQFAMNFIINNCGEVFEGITSIVDVAGGTGTAAREIAKAFPHIKCSVLDLPNVINSISSDGLVNYIVGDMMSSIPTADAVFLKDVLHDWNDEDCVKILTQCKKAITKPGGKVIIVDMVVGSPSTSMFEGQVLFDMLMMVMTPGKEREEHEWGKIFMDAGFNHYKTRPIMGCMAITELYP*

>HORVU5Hr1G066150|HORVU5Hr1G066150.2|Hvulgare_r1

AMATQAQQLAVPTEAELLQGQADLWRHSLYYMTSMAFQCAVKLGIPTTIHSLGGAASLPDLVAALSLPPAKLPYLRRIMRLLATSGVFAADSAAADVVATYRLTPLSWLLLDGVAVDGHPSQTSLVLAATSRHCLEAAMGLSDWFKKDVAASPFQDLHGVTLFDGTMAEQDPEVDAVFNDALASHDNSGFLAVLRECGGTLFQGLESLTDCCGGDGTTARAIVEAFPQIKCTVLDLPRVIDNVPADGVVNYVAGDMFNSVPFAQAVLIKLVLHHWSDEDCVRILAQCKKAIPPREEGGKVIVIDIVVDSSSGPTHEAELLMDVAMMVMTNGRQRDETDWSEIFIKAGFSDYKVVKKLGARGVFEVYP*

>HORVU1Hr1G001570|HORVU1Hr1G001570.1|Hvulgare_r1

MASHIQEDELTMSSEELLQAQLDLYHHCFVYVKSMALGAATDLRIPDAIHLRGGAATLSDLAADTGIYPTKLSHLRRLMRVLTTSGVFSADVNASGDTTYKLTRVSRLLVGAGGESSRRHDLSPMVGLFVNPVPITALFSIREWFTDEKSTASSSLFEVAHGCTRWEMIAKDAGDDRLFNASMAADSCLTMGILLKECNNVFGSIGSSLVDVGGAHGTATAIIAKAFPHVKCTVLDLPRVVAGAPAHDNVIFVPGDMFEYIPPADTVLLKWILHDWPDDDCIKVLRQCKKAIPARDAGGKVIIMDMVVGSAGPQQETVSKEAEVLFDVFMMYIDGIQREEHEWRKIFLEAGFSDYKITPVTGIRSIIEVYP*

>HORVU1Hr1G089520|HORVU1Hr1G089520.1|Hvulgare_r1

MDMVTSMPLEANSNGQILQAEAELFCHSFGYLKSMALQSVVKLRIPDVLHRYGGAASLPELLSTVPIHPNKLPYLPRLMKMLAAAGIFTAEDVPATVGDGEPTTLYHLNAVSRLLVDDASVNGGASMSPCVLLGTVPLFLGASLKLHEWLQSEEQATTETPFMLAHGGTLYGIGGRDSEFNTVFNKAMGASSEFVAALAVRECRDVFAGIKSLVDVAGGNGTTARTIAEAFPYVKCSVLDLPQVIQGISSHGTVEFVAGDMMEFVPPAEAVLLKYVLHNWSDQDCVKILTRCREAISHGEKAGKVIIIDTVVGSPSQQILESQVTMDLSMMMLFNGKVREEQNWHKIFLEAGFSHYKIHNVLGMRSLIEVQP*

>HORVU1Hr1G076660|HORVU1Hr1G076660.1|Hvulgare_r1

MVVHILSAKCQRKLKQHKHTASIMALPGQSTDDQAMLGAEHELWRTTFSYIKSMALKSALDLRLADAIHHHGGAATLPQIVARVMVHPSKIPCLRRLMRTLTVSGVFSVQQQDVVPADYSVSNGNGAVVDPLYTLTPVSRLLVGSQNSASIMAFVLSPVLVTPFLGIGAWFQHALPDPCFFEEAHGEALWEMSEHDVALDTLINSAMVSDSRFIVDIAVRESGDVFRGISSLVDVGGGLGAAAQVISEAFPHVECSVLELEHVVSKAPTGTGVKYVAGDMFQSVPPADAVFLKVRTYMLLLLGPWSRNIHIRLIKHLVNVQRYISIMHVCCLQSVLHDWDDEKCVKILKTCRKAIPPREAGGKVIIVDIVVGADKKHGEVHALLDLYIMFIDGIERDEQEWSKIFLEAGFSDYKIIPVLGFRSIIEVYP*

>HORVU1Hr1G076670|HORVU1Hr1G076670.2|Hvulgare_r1

MALTGDYKLISTEDMLQGHAELCIHAYGFVKSMALKCAIELGIPGAIHGYGGGATLDELATIIALPPSRLPRLRRLMRVLTVSGVFSVQHKQPDDSVCDGAVVYGLTSASRLLVSDGETSSGLSRLVSLMVDPNLTAPFSGMSAWFMDDEQPRSFFEMHHGEDLWDMAARDAALSRTIGDGMTDDSRFVVEVLLRDSRARDVFRGMRSMVDVGGGTGAIAKAIATAFPQVECSVLDLPHVVAEAPADGEVRFIAGDMFDYIPPADAVLLKSVMHDWRDDECVKILRRCKEAIPSKEAGGKVIIINMVVGSGMSKGKGTEKEEAQALYDLFLMVFEGGEREEVEWEKIFLDAGFSGYNIIPVLGIRSIIEVYP*

>HORVU6Hr1G090510|HORVU6Hr1G090510.2|Hvulgare_r1

MAGQAAEKVFVPTDAELLQAQSDLWRHSLCYLTPMSLRCAVDLGVPTAIHRLGGAASPSELVAALSLPASKLPFLARLLRQLATAGVFTSTDAGTYRLNPLSYLLVDGVRIDGDASQTAIVRAAASRYYVEAAMGLADWFRKDFDGAVPSPFEDVHGAAIFEESMALLDPEMDQLIHDAVAAHDHMGIGPVLRQCRELFEGLESLTDCGGGDGTTARSIVEAYPHINCTVLDLPKVMDKALPPAQQGVVKYVSGDLFHVVPPAQAVLLKLVLHFWSDEDCIKILAQCKKAVPPRDAGGKVIVIDIVLGSVSGPMLETQHLMDMLMLVMTRGRQREEKDWSEIFTKAGFSGYKIVKKLGARAVIEVYP*

>HORVU6Hr1G003160|HORVU6Hr1G003160.2|Hvulgare_r1

MTLKLLMEVSPQELLEALAELQNHVLGYVKATSLKCAVDLGVPAAIHKRGGTATLADIATDAKVHPAKVADLRRMMALLTASGIFTTTAEDGDGAADDTAAVQYGLTTTGRFLVGWRNLSPVAPFFVNPLIVSSFFDLPGWLRSEPAPAGAGSLFEQAHGCSRQEMARKDAGFGTVLRDAMTADSELFLEVIIVDKGRIFRGVTSLVDVGAGNGAGTRVVAKAFPRIKCTVLDRPQSLGQAADAGDDNLRFVAGDMFESIPPADAVLLKNVLHDWGHDDCVKILQRCKEAIPARSAGGKVIIIDMVRGSLLGDKKIAEMEAMQDMFAMCTNGVERDESEWKRIFSDAGFGQDYKIMPVLGPLSVIEIYP*

>HORVU7Hr1G018870|HORVU7Hr1G018870.2|Hvulgare_r1

SFEMENFTAGLCRCVAYLSTDAITYRNHTLITDRNGERRGRAMAAQADTIEVPTDAELLQAQADLWRHSLYYLSSMGLRCAVQLQIPTTIHRLGGVALLADLMAALSLPSVKMPFLHRLMRVLVTSGVFAADKDSESGGELYRLTPLSRILVDGVTDEHHSQKYFVLAVTAPHCAEAALGLADWFKKDLEPPVPSPFEDMHGAPVFDERTPLMDEEFDAVANEGLAAHDNLGIATILRECGDIFKGLESLTDCCGGDGTTARALVKAYPHIKCTVLDLPKVIDKAPKDGVVNYVAGDLFHTVPSSQAVMLKLVLHFWSDEDCVKILTQCKKAIPPHDEGGKIIIIDIVIGPSLGPIMFEAQLLMDMLMMVTTRGVQRSENDWRKLFMEAGFKDYKIVKKLGARCVIEVYP*

>HORVU7Hr1G008260|HORVU7Hr1G008260.2|Hvulgare_r1

MAVPSDAELLQAQADLWRHTLCYLTPMALRCAVELGIPTAIHRLGGAASPSDLIAALSLPPSKLSFLGRLLSQLATAGVFDSTDDMGTATYRLKPISYLLVDGVHIEGDACQTAIVRAASSRYYVEAALGLSEWFKKDFEGPVPSPFEDLHGATIFDDSMALLDPEMDKLVHEAKASHNHMGIGLVLRQCRELFQELESLTDLCGGDGTTARAIVRAYPHIKCTVMDLPKVIDKAPAEGVIKYVAGDIFHAVPPAQAVLLKSVLHLWSDEDCIKILAQCKKAIPPRGAGGKVIVIDIVLSSASGLMLETHHLVDMLKLVMTRGRQRDEKDWSDIFMKAGFSEYKVFKKVGARTVIEVYP*

>HORVU7Hr1G117710|HORVU7Hr1G117710.10|Hvulgare_r1

MIETERAMAAQVPAIEVPTDAELLQAQADLWRHTLYYLTSMGLRCAVKLGIPTAMHNLGGVTSLPDLAAVLSIPASKQPFLGRLMRALVTSGVFAAGGTNNSGAELFRLNPLSRVLVDGVDAEEHHSQTSFVLAGTSPHYMEAALGMAEWFKKDVTGPVPSVFEDVHSASLFDESTAALDPELDALVTDGLEAHDNLGIGTIMRECHDLFKGLESLTDCCGGDGKTARAITKAHPHVKCTVLDLPKVIEKTPSDCVVNYVAGDLFHTVPKAQAVMLKLVLHHWSDDDCVKILTQCKNAIPSREEGGKVIVIDIVVEPSLGPVMFEAQTLMDMLMLVFTRGRQRSENDWRDLFMKAGFTDYKIIKKMGARGVIEVYK*

>HORVU7Hr1G117850|HORVU7Hr1G117850.1|Hvulgare_r1

MAAQAPEMEVPKGAELLQAQADLWRHTLYYLTSMGLRCAVKLGIPTAIHNLGGVSSLADLAAALSIPASKQPFLGRLMRALVTSGVFANGGKNDSGAELFGLNPLSRILVDGVVADEHHSQTSFVLAGTSRHYMEAALGMADWFKKDATGPVPSVFEDVHSASLFDESTAALDPELDALVTDGLAAHDNLGIGTIIRECHDLFKGLESLTDCCGGDGTTARAITKAHPHIKCTVLDLPKVIDKTSADGIVNYVGGDLFHTVPKAQAVMLKLVLHHWSDDDCVKILTQCKNAIPSREEGGKVIVIDIVVEPSLGPVMFEAQTLMDILMLVFTRGRQRSESDWHVLFTKAGFSDYKIVKRLGARGVIEVYK*

>HORVU7Hr1G018880|HORVU7Hr1G018880.1|Hvulgare_r1

MLRKIPPRNSQSKTFNHIIKPRKGERAMATQAETIEVPADAELLQAQADLWRHSLYYLSSMALRCAVELEIPTAIHRLGGVASLPDLMAALSLPSVKMSFLGRVMRVLVNSGVFAADNSSEYGVELYRLTPLSRVLVHGVLADEHHSQKYFVLGVTSPHYTEAALGLADWFKKDTELPVLSPFEEKFGVPLFDEKTALLDEELDSVCNQGLAAHDNLGIATILRECGDIFKGLDSLTDCCGGDGTTARALVKAYPHIKCTVLDLPKVVEKASADGVINYVAGDLFHTVPPSQVVMLKLVLHFWSDEDCVKILAQCRKAIPPREEGGKVIIIEIVVGPSLGPVMFEAQLLMDMLMMVNTRGGQRDENHWCELFKKAGFTDYKIVKKLGARSVIEVYP*

>HORVU7Hr1G011340|HORVU7Hr1G011340.2|Hvulgare_r1

MVLTREQRTDQGLLDAQLELWHISFGYVKSMALKSCLDLGIADAIHHHGGAATLSQIGAVATLHPSKICCLRRLMRVLTVSGVFSVQQPSPRDDVQVELVYTLTAVSHLLVSSASVNIVPVINLQLQPNIVSSFSELGAWFQHKLPEPDLFKLKHGKTFWEIAHHNAAFNTIVNDAMASDSRFLMDIAIRECGSVFEGIGSLVDVAGGHGGAAQAISKSFPHIKCSVMDLGHVIAGAPSGTDVQYIAGDMFESIPQADVVFLKWIMHDWSDDDCIKILKNCNKAIAPKDAGGKVIIVDMVVGGGPQDLKHKETQVLFDLYIMLLNGIERDEQEWKKIIMAAGFSDFKITPILGVRSIIELYP*

>HORVU7Hr1G117890|HORVU7Hr1G117890.1|Hvulgare_r1

MALNVQKSSSALADAELELWTNTFSYIKSMALKSALDLRLADAINHHGGVATLSQIVTRVTLHHSKIPCLRRVMRVLTLTGVFSVQQDQVLSTDDEPTYALTPASRLLVGSHNLASMMAMLLNPIMLTPFLGIGDWFKYGQPDPSLFEQTHGEGLWKMAHRDATFDALINDGMVSDSRFIMDIAVKECGEVFQGITSLVDVGGGLGAASQAISKAFPHLECTVMDLGHVIANAPTGTDVKFVAGDMFESVPAANAVFLKWVLHDWSDDECVRILKNCKEAILAKEGGKVIIMDMVIGAGPSDVKHREMQAMFDLYMIIINGIERDEKQWKQIFMEAGFSRHKVMPVQGFRSIIEVYP*

>HORVU4Hr1G076000|HORVU4Hr1G076000.2|Hvulgare_r1

NKHTTLQLEIYNAMAAQAPTMAVPTDAQLIQAQADLWRHSLCHLRAIALRCAVQLGIPTAIHRLGGTTSVSDLVTALSLPQSKMPYLGRVLRLLAASGALASPKEGTYSLVPLSYLLVDGVFIDGEASQKAIVLTTTSRHYIEAALGLADWFKKDIAPPPSPFEDVHGATLFEESMALLDPESDKMFHEALAAHDHLGIGTILRECHDLFKGVQSLTDCCGGDGTTARAIVKAFPHIKCNVLDLPKVIEKVPSDGIVNYVAGDLFHAIPPAQVLMLKLVLHFWSDEDCINILSQCKKAIPSREMGGKVIVIDIVVGSSSKEILETELLMDMLMLVCTKGRQRDENDWSTIFTKAGFSDYKIVKKLGARGIIEVYP*

>HORVU4Hr1G076050|HORVU4Hr1G076050.1|Hvulgare_r1

MVCVCTYIGASAQAVNKHTTLQSEIYNAMATQSPTMAVPTDAQLIQAQADLWRHSLCHLTAMALRCAVQLGIPTAIHRLGGTTSVSDLVTALSLPQSKMPYLGRVLRLLATSGALASPKEGTYSLVPLSYLLVDGVFIDGEASQKALLLSTTSRYNIEAALGLADWFKKDITPPPSPFEDVHGATLFEESMALLDPESDELFHEALAAHDHLGIGTILRECHDLFKGVQSLTDCCGGDGTTARAIVKAFPHIKCNVLDLPKVIEKVPSDGIVNYVAGDLFHAIPPAQVLMLKLVLHFWSDEDCINILAQCKKAIPSREMGGKVIVIDIVVGSSSKEMLETQLLVDMLMLVCTRGRQRDENDWSTIFTKAGFSGYKIVKKLGPRGVIEVYP*

>HORVU4Hr1G012540|HORVU4Hr1G012540.1|Hvulgare_r1

QEPECIVERRRAMAGGEAATMEVPTDAELLQAQADLWRHSLYYLTSMGLRCAIKLGIPTAIHRLGGVTSLPDLMAALSLPASKQPFLGRLMRVLVTSGVFAAGGNGSESGSEAGELFSLNPLSRILVDGVVGHDEHHSQTSFVLAATSPFYTEAALGLADWFRKDVTGPAVPSPFEDVHGASLFDESTALLDKELDELVTEGLAAHDNLGIGTIMRECHDLFKGLESLTDCCGGDGTTARAITKAHPHVKCTVLDLPKVIDKAPSDGIVNCVAGDLFHSVPKSQAVMLKLVLHHWSDEDCVKILSQCRDAIPSHEEGGKVIIIDIVVGPSLGPVMFEAQLLMDMLMLVNTRGRQRTENDWHELFMKAGFSDYKIVKKLGARGVFEVYK*

>HORVU4Hr1G085680|HORVU4Hr1G085680.2|Hvulgare_r1

HPIQSPEHQESDREIMAAQSQTMTVPTDAQLIKAQADLQRHSLTYLTSMALRCAIELGIPTAIQRLGGTASLPDLMAALSLPPPKAPFLSRVLRLLAKSDALACTEAGIYSLTPLSYILVDGVLIDGEARQVTFPLAVTSRYHMESGLGLADWFKNDNLLPVPSPFEHVHAAAPFDESMTLLDPETDKLFYEALAAHDHMGIGTVLRECRGLFDGLQSLTDCCGGDGTTARAIVKAFPHIKCNVLDLPKVIDKVPSDGVVNYVAGDLFHTVPPAQAVMLKLVLHFWSDEDCINILAQCKKAIPSREMGGKVIIIDIVLGSSLATITETELLMDMLMFICTRGRQRNEKEWSMLFMKAGFSDYKIIKKLGHRGVIEVYP*

>cds.Jr16_05020_p1

MGSTGETQMTPTQVSDEEANLFAMQLASASVLPMVLKSAIELDLLDIIAKAGPGAYLSPSEVASQLPTTNPDAPVMLDRILRLLASYSVLTYSLRTLPDGKVERLYGVGPVSKFLTKNEDGVSIAALCLMNQDKVLMESWYYLKDAVLEGGIPFNKAHGMTSFEYHGKDLRFNKVFNKGMSDHSTITMKKILETYKGFEGLTSLVDVGGGTGAVLSTIVSKYPSIRGINFDLPHVIEDAPSYPGVDHVGGDMFVSVPKGDAIFMKWICHDWSDEHCLKFLKNCYEALPDNGKVIVAECILPVAPDTSLAAKGVIHIDVIMLAHNPGGKERTEGEFEALAKGAGFQGFRVMCCAFNTYIMEFIKKL

>cds.Jr13_24380_p1

MTPTQVSDEEANLFAMQLASASVLPMVLKSAIELDLLEIMAKAGRGAYLSPSEIASQLPTTNPDAPVMLDRILRLLASYSVLTYSLRTLPDGRVERLYGLGPVSKFLTKNEDGVSIAPLCLMNQDKVLMESWYYLKDAVLEGGIPFNRAHGVTSFEYHGKDLRFNKVFNKGMSDHSTITMKKILETYKGFEGLTSVVDVGGGTGAVLSMIVSKYPSIKGFNFDLPHVIEDAPSYPGVDHVGGDMFVSVPKGDAIFMKWICHDWSDEHCLKFLKNCYKALPDNGKVIVAECILPVAPDTSLAAKGVIHIDVIMLAHNPGGKERTEKEFEALAKGAGFQGFRVMGCAFNTYIMEFIKKL

>cds.Jr13_26200_p1

ILTFLRCRKHDTPKFQTLSKGTYPSAFLILGFTLTSFAQVMSLSENNNAIRIAQQQELNEEEEAGKLAVRLANGVILPMVLKSALELNLIDIISDAGIGVFLSPSEIAARLPTKNPDTPVLLDRMLRLLASYSILKYSLRTREDGEIERLYGVAPICKFLVKNPHGGSVAPLFLLHHDKVFMESWYHMNDAILDGGIPFNRAYGMTAFEYPGTDERFNRVFNQAMSNHTNLIMKKILDVYKGFEGLKVLVDVGGGVGVTLNRITSKYPQIKGINFDLPHVLTDAPSYPGVDHVGGDMFDSVPCGDAIFMKWILHDWSDEHCLRLLKNCWKALPKSGKVIIVESILPVAPESKVSSNIVFEQDLFMLAQNPGGKERTQKEFEALALKSGFSGCEVICCAYNSWVMEFHKRADP

>cds.Jr07_09880_p1

MASLTESHSLNNLDGHDRKEEDESFLYAGQLVNSLVLPMALQTAIELGVFEILAKAGPEAKLSSSQIVAKMPTTNPEAPQMLDRILRMLASHSVVRCFAGADDAGAFQRLYSLSPVAKQFVPDEDGISFGHMMALLQDKVFVDSWFQLKGAILEGGIPFDRVHGTNAFEYPGLDPKFNQVFNKAMLNHTTMVIKKILEYYKGFETLKQLVDVGGGLGVALNLITSKYPHIKGINFDLPHVVQHAPSYPGVEHVGGDMFKSVPKGDAIFLKWILHDWSDEHCVKLLKNCYAAIPNDGKVIVVEAVLPKMPEVSTSMRCTSQLDVLMLTQNPGGKERTEEEFMALATKAGFKGIRYECFVYSFWLMEFFK

>cds.Jr07_09890_p1

MASQTESNSLNNLDGHGQKEEDESFLYAGQLVNSLVLPMALQTTIELGVFEILAKAGPGAKLSSTQIVAKMPTTNPEAPQMLDRILRMLASHSVVRCSAGADDAGAFQRLYALSPVSKQFVPDEDGISFGPMMALHQDKVFVDSWFQLKAAILEGGIPFDRVHGINAFEYTGLDPKFNQVFNKAMLNPTTMVIKNILECYKGFETLKQLVDVGGGLGVSLNFITSKYPHIKGINFDLPHVVQHAPSYPGVEHVGGDMFKNVPKADAIFMKWILHNWSDEHCVKLLKNCYAAIPNDGKVIVVEAVLPKMPEVSTSMRCTSQLDVLMLTQIPGGKERTEEEFMALATKAGFKGIRYQECFVNTFWLMEFFK

>cds.Jr07_09710_p1

MASHTAFRPSNLDGDQTKEDESFVYASQLANSLVLPMALQTAIELGVFEILAKAGPGAKLSPSQISTEMPTTNPEAPKMLDRILRMLTSHSVLSCSAGADDAGSFQRLYSLSPVSKQFVLDQDGVSLCPLMALVQDKVFLASWSQLKDAILEGGIPFDRVHGTSAFEYLGMDPRFNKVFNTAMLNHTTMLFKKILESYRGFEPLKQLVDVGGGLGGALHSITSKYPHIKGINFDLPHVVQQAPSYPGVEHVGGDMFKSVPKGDAIFMKGILHDWGDEQCLKLLKNCSTAIPNDGKVIVVEAFLPMMPEVSTCMKRNSQLDVLMMAQHPGGKERTQQEFEALATKAGFKGIRYECVVYSTQVMEFFK

>cds.Jr07_09860_p1

MMASLTESHSLNNLDGDQKEEDESFLYAGQLVNSLVLPMALHTAIELGVFEILAKAGHGAKLSSSQIVAEMPTTNPEAPQMLDRILRMLASHSVVRCSAGADDAGIFQRLYALSPVSKQFIPDEDGISFGHLMALFQDKVFVDSWFQLKGAILEGGIPFNRVHGINAFEYPGLDPKFNQVFNKAMLYPTTMVIKKILECYKGFETLKQLVDVGGGLGVSLNLITSKYTHIKGINFDLPHVIQHAPSYPGVEHVGGDMFKSVPKANVIFMKWILHDWSDEHCVKLLKNCYAAIPNDGNVVVVEAVLPKMPEVSTSMRVTSQLDVLMLTQNPGGKERTEEEFMALATKAGFKGIRYECFVYGFWLMEFFK

>cds.Jr10_14980_p1

MERNEEIMPILEKGQAAIWQLLFAFADSMALKCAVELRIADIIHSHGVPVTLNQIASEIDSPTSPDIPYLARIMRSLVHKKIFTEHHLPDGSDTLYGSTETSRWLLHDAELSLVPMVIMENNPWQLSPWHCLSQCVKEGGIAFQKAHGCEMWDFAAKNPEFNKIFNDAMTCTTKIMMGVVLTEYKDGFNSIGSLVDVGGGTGEMIAKIIKSHQHIKAINFDLPHVVAAAPLRQGVSHVGGNMFEAIPSADAIFIKCVLHDWSDEHCIKILKNCKKAIPPKSGKVIIVDIVLEKENDDLYQDTRMVFDLVMMAHTTGGKERTEVEWKQLLMDAGFPRYKIIKIPSIPSIIEAYPVM

>cds.Jr12_07430_p1

MERNEEIMPILEKGQAAIWQLLFAFADSMALKCAVELRIADIIHSHGVPVTLNQIASEIDSPTSPDIPYLARIMRSLVHKKIFTEHHLPDGSDTLYGSTETSRWLLHDAELSLVPMVIMENNPWQLSPWHCLSQCVKEGGIAFQKAHGCEMWDFAAKNPEFNKIFNDAMTCTTKIMMGVVLTEYKDGFNSIGSLVDVGGGTGEMIAKIIKSHQHIKAINFDLPHVVAAAPLRQGVSHVGGNMFEAIPSADAIFIKCVLHDWSDEHCIKILKNCKKAIPPKSGKVIIVDIVLEKENDDLYQDTRMVFDLVMMAHTTGGKERTEVEWKQLLMDAGFPRYKIIKIPSIPSIIEAYPVM

>cds.Jr04_16510_p1

MDLVNGQGASDELSRLQSHLYKNILSFTDSMSLYCAIQLGIPEIIHNHGQPITLKQLASKLRIHPQKTRCMHRLMRLLVHSGFITKTAPVVHEDQEEEEAYALTASSRVVLVDKVTGLSPLVQAILDPAVVNSWYSLGDWFRGTELTPFVKAHGMGIFDYCNQNPEYGVAFDEGMASDSRLMSKVIKDYKTIFDGLDSLVDVGGGSGTVSRIISEAFPHIKCTVFDLPHVVANLPDDKNLKYVGGDMFQSIPAADAILMKWILHDWSDEECVEILKRCKEAIRSKGKDGKIIIIDVVINEEEEQHDITKAKLFLDALMMILVTGKERTKKEWEKLFLDAGFSHYKIVAPYGLKSLIEVYPNS

>cds.Jr01_20680_p1

MERKEEVALLKGQAEIWQHLFAFADSMALKCAVELRLADIIHSHSVPITLSQIASAIDSPSPDIPYLSRIMRSLVYKKIFTEHHPSDGGETVLYGPTHTSRWLLHDAELTLAPLVLMENNQWQLAPWHFLSQCVKEGGIAFKKAHGFEMWDFAARNPEFNKIFNDAMACTTKIMMGVLLAEYKDGFGSIGSLVDVGGGTGEMIAEIIKQHPHIKGINFDLPHVVATAPVHEGVSHVGGNMFEAIPNANAILLKCVLHDWSDEHCIKILRNCKKTVPQKTGKVIIVDIVIEKDNDDLFQETRMVYDLLMMAHTTGGKERSELEWKQLLKEGGFPRYKIIKLPSITSIIEAYPE

>cds.Jr07_09790_p1

MASHTAPHPSNLDGDQKEEDESFVYAGQLANSLVLPMALQTAIELGVFKILAKSGPGAKLSPSQISTEMPTTNPEAPKMLDRILRMLTSHSVLSCSAGADDAGSFQRLYSLSPVSKHFVPDQDGVSFRPLMALLQDKVFLASWFNKVFNTAMLNHTTMLFKKILESYKGFEPLKQLVDVGGGLGGALHSITSKYPHIKGINFDLPHVVQQAPSYPGVEHVGGDMFKSVPKGDAIFMKGILHDWGDEQCLKLLKNCYTAIPNDGKVIVVEIFLPMMPEVSTYMKRNSQLDVLMMAQHPGGKERTRQEFEALATKAGFKGIKYKCLVYSFQVMEFFK

>cds.Jr16_03670_p1

MASPANQISYDNKPFIDDEDGGVEYAGALAGSYIFQMVFNACIELDVLEIINKAGPGAKLSSHAIAAQLPIRNNNPDHAPAVLLDRMLYLLASYNILSCSVESLEDGGGGGVQRRYGLTSAGSCFVRSEERGSMAPFSLLTRHQAIMDMRFHLKDAVLEGGFPFEKAHGMSIYKYNDKDPIFGEAFNFAMSQYSILVVKQILKKYMGFEGLSTFVDVGGGIGGTLNLIISKYPSIKGINFDVSEVLRNAPPLKGIEHVGGDMFKEVPKGDAILLKLILHNWNDEQCLKILKNCYKALPETGKVIIIDPVLPVAPEATNFCKFATQSDNIVFNKLGAGKERTETEFRSLATAAGFKDFRVACCVCAYLVMELHK

>cds.Jr10_15890_p1

MSSFRQHSFKLVPGEHATELLNAQAHIWNHIFNFINSMSLKCAIQLGIPDIIHNHAKPITLSELLVALPIHPTKARNIPRLMRILIHSGFFVAEEVIGNDEEERYALTDASRLLLKENPLSVTPFLLAVLDPVLTKPWHFLTAWFQNEDLTPFDTAHGKMFWDYAGHEPKLNNIFSDAMASDARLVMSVVIDKCSAVFGGLESLVDVGGGTGTMAKAIADAFPSMECTVLDLPHVVAGLEGSKNLKYIGGDMFEAVPPADAILLKWILHDWNDEECVKILRRCKEAITSNDKKGKVMIIEMIVLQNQKEDDKESIETQLFFDMLMMVLVTGKERNEKQWAKLFFDAGFSDYKITHIFGLRSLIEVYP

>cds.Jr16_08680_p1

MGSFRQHSFKLVPGEHATELLNAQAHIWNHIFNFINSMSLKCAIQLGIPDIIHNHAKPMTLSELLVALPIHPTKARNIPRLMRILIHSGFFVAEEMIGNDEEERYALTDASRLLLKENPLSVTPFLLAMLDPVLTKPWHFLTAWFQNEDLTPFDTAHGKMFWDYGGHEPKLNNIFSDAMASDARLVMSVVIDKCSAVFGGLESLVDVGGGTGTMAKAIADAFPSMECTVLDLPHVVAGLEGSKNLKYIGGDMFEAVPPADAILLKWILHDWNDEECVKILRRCKEAITSNDKKGKVMIIEMIVLQNQKEDDKESIETQLFFDMLMMVLVTGKERNEKQWAKLFFDAGFSDYKITHIFGLRSLIEVYP

>cds.Jr04_07880_p1

MHIAYANIGKERYQKIEQVQNMEVQEDGDHFISSQLGGLASTQMALRAAIELKVFSIIADAGPDAHLSAAEIISKIPTKDPSSAEWTLERVLRVLGANSILSISRKPLGNNGEHGRHEWTYGLTKKSRCLVSSSSTTDELATFTTSLILFATEREMLESQYMIKDAVLDPGSSPFYKAYGVNFYDYMGEKPRLRQLFDEFMEVSSKLQFEDVFKLYGGFKDLKELMDVGGGIGTSLAKITSTYPHVRGLNFDLPHVIDAAPKLPGVKHVAGDMFKSIPNAQTILLKWILHNWDDEHCKKLLRNCWEALPRDGKVIIVEMVISEEVGNNLEAKDVIMEDFFMMLLMTGGKERTLAEFKDLGKAVGFTKMEIFPMPHWSVHVIEFHK

>cds.Jr03_07860_p1

MDLVNCGQEASELFRVQSHLYKHLFSFIDSMSLGCAIQLGIPDIIHSHGQPMTLPQLVSKLHIDPKKTICVHRLMRLLSHSGFFTKTTIVHEDRKEEEAYALTPSSRLVLKDDRTSLSPFVVAMLDPALVNPWYSLGDWFRGTELTPFAKANGMGLWDYCNQNPKYGDTFNEAMASDSRFMRLVVKEYKPIFEGLGSLVDVGGGTGTMARIISEAFPHIKCTVYDLPHVVANLPETSNLNYVGGDMFQSIPPTDAIMFKWIMHDWNDEDCVNILKRCKEAITSKGYKEGNKVIIIDVVINEEKDDEDTTKAKLLFDTLMMVLLPGKERTKKEWEKLFMEAGFSRYKIVASYGMKSLIEVYP

>cds.Jr03_07840_p1

KIMDLTHAQGEGATEQFQAQSHLYKHVFNYIGSMSLKCAVQLGIPDIIHNHGQPITLPELVSKLQIHPSKAGFVPRLMRLLVHSGLFATTVRVKNQEEEEAYDLTPSSRLLIKDQIISLSPFVLAMLNPALVTPCHDLGNWYQGDKKSTPFESVHGMSFWDYNDQNPEFNSLFNEAMASDSGMMNLIVKDCKAVFEGLDTLVDVGGGTGTCARIISEEFPHLKCTVFDIPHVVANLAADSLKLNYVAGDMFESIPSADALLLKLVLHGWSDEHCVKILKKCREAISKNGSKGEGKVIIIDVVINEKKDEHEMTEAKLVFDMLMMIVASGRERDEKDFKKLFLEAGFNHYKITPIYGLRSIIEVYP

>cds.Jr04_07960_p1

MEVQEDGDHFITSQLGGLASTQMALRAAIELKVFSIIADAGPDAHLSAAEIISKIPTKDPSSAAWTLERVLRVLGANSILSIISRKPLGNNGEHGRHEWTYGLTKKSRCLVSSCTTDELATFTTSIILFTTEREVLESQYMIKDAVLDPGSSPFYKANGVNFYDYMGEKPRLRQVFDEFMEVSSKLPFEDVFKLYGGFKDLKELMDVGGGIGTILAKITSTYPHVRGLNFDLPHVIDAAPKLPGVKHVAGDMFKSIPNAQTILLKWILHNWDDEHCKKLLRNCWEALPRDGKVIIVEMVISEELGNNLEAKDVIMEDFFMMLLMTGGKERTLAEFKDLGKAVGFTKMEIFPMPHWSVHVIEFHK

>cds.Jr04_07870_p1

MEVQEDGDHFISSQLGGLASTQMALRAAIELKVFSIIADAGPDAHISAAEIISKIPTKDPSSAACTLERVLRVLGANSILSISRKPLGNNGEHGRQEWTYGLTKKSRCLVIRSGTTDELANFTTSFILFATEREMLESQYMIKDAVLDPGSSPFYKSYGLNFYDYMGEKPRLRQVFDEFMEVCAKLQFKDVFKLYGGFKDLKELMDVGGGTGTILAKITSTYPHVRGLNFDLPHVIDAAPKLPGVKHVAGDMFKSIPNAQTILLKCILHNWDDEHCKKLLRNCWEALPRHGKVIIVEMVISEELGNNLEAKDVIMEDFFMMLLMTGGKERTLAEFKDLGKAVGFTKMEIFPMPHWSVHVIEFHK

>cds.Jr04_07730_p1

MEVQEAGDQLSSSQLGGLVSIPMVLRAAIELNVFNIIADAGSDAYLSAAEITSKIPTTDPNSAAYTLERILRFLGANSILSISQKPLGNNGENAVRHEWTYGLTKMSRCLVSISTGGSTATSPASVMIRFSCQRELLESLLRIKDAVLEPGSAPFKKAYGVNFYEYLEKKPIFRQLFDELMEVGEKLVFDDVFKVYGGFEDVRELIDVGGGIGTTSAKIISLNPHVRGLNFDLPQVIADAPTLPGVKHAAGDMFESIPNAQTILLKRILHNWDDERCKRLLRNCWEALPADGKVIVVEMAIPQELENNLETMNVLKEDLYMMLLMAGGKERTLAEFDHLAKAVGFTKMKVFPMPHGSVHVIELYK

>cds.Jr_Scaffold_205_00020_p1

MEVQEAGDQLSSSQLGGLVSIPMVLRAAIELNVFNIIADAGSDAYLSAAEITSKIPTTDPNSAAYTLERILRFLGANSILSISQKPLGNNGENAVRHEWTYGLTKMSRCLVSISTGGSTATSPASVMIRFSCQRELLESLLRIKDAVLEPGSAPFKKAYGVNFYEYLEKKPIFRQLFDELMEVGEKLVFDDVFKVYGGFEDVRELIDVGGGIGTTSAKIISLNPHVRGLNFDLPQVIADAPTLPGVKHAAGDMFESIPNAQTILLKRILHNWDDERCKRLLRNCWEALPADGKVIVVEMAIPQELENNLETMNVLKEDLYMMLLMAGGKERTLAEFDHLAKAVGFTKMKVFPMPHGSVHVIELYK

>cds.Jr09_08980_p1

MSSKESQMGISSTEYQESVSEYAMQLTSASVLPSVLKAAIDLGVLVIIGKAGPGALLSASQIASELSTHSNANLVSSLLLDCMLRILASHSIFTCSITHQNDGHVLRLYGLAPVSKYFDRGSQDGGPVVGMLDLMQDKDIMSIWDHLKDAVLEGVLPCYKSHGMNMSEFLGKDARFHETFKGLMKSLSPTLMEKILETYKGFEGLKSLVDVGGHDGTILNIIISKYPSIKGVNFDMASVIEKSPPYPGIEHVAGDMFVSIPKGDAIFMKWIIHHYNDEDSLKVLKNCYEALPEHGKVILVDMVVPEVPETSAAHKSLFQFYLFIMNANPQGKERTERELESLAKAAGFSGIQVVGFAYGYSLVEFYKNM

>cds.Jr03_07850_p1

MYVHARSSELRDLHQIKKMDLVNCGQEGSELFQVQSHLYKHLFSFIDSMSLGCAIQLGIPDIIHSHGQPMTLPQLVSKLHIDPNKTICVHRLMRLLSHSGFFTKTTTVHEDGEEEEAYALTPSSRLVLNDDRTSLSPFVVAMLDPALVNPWYSLGDWFRGTELTPFAKVNGMGLWDYCNQNPEYGDTFNEAMASDSRFMRLVVTEFKPIFEGLGSLVDVGGGTGTMARIISELFPHIKCTVYDLPHVVANLPETSNLNYVGGDMFQSIPPTDAIMFKCVMHNWNDEDCVNILKRCKEAITSKGYKKGNKVIIIDAVINEEKDDEDTTKAKLLFDTLMMVLLPGKERTKKEWEKLFMEAGFSRYKIMASYGMKSLIEVYP

>cds.Jr01_20610_p1

MELTTMEAMIQSQAKLWGHIFTFTDSMALRCALELGIVDIIHSHGGPITLSQIAAGIDSPSLNIDNLARVMRLLVRKDIFTTHHQPSDSGEITLYGLTAHTSRWLIQGSEFSLVPFILMQTHPGLMTSWFSLSQCVEEGGTAFSKVHGCEVIDFAHNNPEFNKMFNDAMACTAKLVAEPILWGYRDGFGSINGSLVDVGGGTGEMVAKIVKAHPHIKAINFDLPHVITTAPMHKGVSHVEGNMFEAIPNADAILLKRVLHGFSDEDCIKILKNCVKAISKKTGKIIIVEHVLDPNGNGPFDETGLAFDLIMMTVAPSGKERTELDWKKLLEEGGFPHHKIIKIPAFLSIIEAHPA

>cds.Jr_Scaffold_58_00010_p1

DMQVQNMEVQEAGDQLSSCSQLGGLVSIQMVLRAAIELNVFNIIADAGPDAYLSAADITSKIPTTDPNSAAYTLERILRFLGAYSILSISRKPLGNNGENASHEWTYGLTKMSQCLVSSGTGGSTATSPASIMIRISCERELLESLLRIKDAVLEPGSAPFKKAHGVNFYEYLEKKPIYRQLFDEFMEVGEKLVFDDVFKVYGGFEDVRELIDVGGGIGTTSAKIISLNPHVRGLNFDLPQVIADAPALPGVKHAAGDMFESIPNAQTILLKRILHNWDDERCKRLLRNCWEALPADGKVIVVEMAIPQELENNLETMNVLKEDLYMMLLMAGGKERTLAEFDHLAKAVGFTKMKVFPMPHGSVHVIELYK

>cds.Jr04_07760_p1

DMQVQNMEVQEAGDQLSSCSQLGGLVSIQMVLRAAIELNVFNIIADAGPDAYLSAADITSKIPTTDPNSAAYTLERILRFLGAYSILSISRKPLGNNGENASHEWTYGLTKMSQCLVSSSTGGSTATSPASIMIRISCERELLESLLRIKDAVLEPGSAPFKKAHGVNFYEYLEKKPIYRQLFDEFMEVGEKLVFDDVFKVYGGFEDVRELIDVGGGIGTTSAKIISLNPHVRGLNFDLPQVIADAPALPGVKHAAGDMFESIPNAQTILLKRILHNWDDERCKRLLRNCWEALPADGKVIVVEMAIPQELENNLETMNVLKEDLYMMLLMAGGKERTLAEFDHLAKAVGFTKMKVFPMPHGSVHVIELYK

>cds.Jr04_12680_p1

MELTTMEAMIQGEAKLWGNIFAFMDSMALRCALELGIVDIIHSHGGPITLSQIAAGIDSPSLNIDILERVMRLLVRKDIFTTHHQPSDSGEITLYGLTAHTSRWLMQGSEFSLVPFILMQSHPGLMASWLCLSQCVKEGGTVFSKVHGCEVIDFAHKNPEFNKMFNDAMACTAKLVAEPILWGYRDGFGSINGSLVDVGGGTGEMVAKIVKAHPHIKAINFDLPHVITTAPMHQGVTHVEGNMFEAIPNADAILLKRVLHCYSDEDCIKILKNCVKAIPKKTGKIIIVEHVLEPNGNGPFDETGSVFDLIMMTVNPSGKERAQLDWKKLLEEGGFPHHKILKIPAFPSIIEAHPA

>cds.Jr04_12690_p1

MELTTMEAMIQGEAKLWGNIFAFMDSMALRCALELGIVDIIHSHGGPITLSQIAAGIDSPSLNIDILERVMRLLVRKDIFTTHHQPSDSGEITLYGLTAHASRWLMQGSEFSLVPFILMQSHPGLMTQWLCLSQCVKEGGTAFSKVHGCEVIDFAHKNPEFNKMFNDAMACTAKLVAEPILWGYRDGFGSINGSLVDVGGGTGEMVAKIVKAHPHIKAINFDLPHVITTAPMHQGVSHVEGNMFEAIPNADAILLKRVLHCYSDEDCIKILKNCVKAIPKKTGKIIIVEHVLEPNGNGPFDETGLVFDLIMMTVTPSGKERTQLDWKILLEEGGFPHHKIIKIPAFPSIIEAHPA

>cds.Jr01_20620_p1

MEAMIQSQAKLWGHIFTFTDSMALRCALELGIVDIIHSHGGPITLSQIAAGIDSPSLNIDNLARVMRLLVRKDIFTTHHQPSDSGEITLYGLTAHTSRWLIQGSEFSLVPFILMQTHPGLMTSWFSLSQCVEEGGTAFSKVHGCEVIDFAHNNPEFNKMFNDAMACTAKLVAEPILWGYRDGFGSINGSLVDVGGGTGEMVAKIVKAHPHIKAINFDLPHVITTAPMHKGVSHVEGNMFEAIPNADAILLKRVLHGFSDEDCIKILKNSVKAIPKKTGKIIIVEHVLDPNGNGPFDETGLAFDLIMMTVAPSGKERTALDWKKLLEEGGFPHHKIIKIPAFLSIIEAHPA

>cds.Jr09_09000_p1

MSSKESQMGTSSTEDQESISEYAMQLTSASVLPSVLKAAIDLGVLEIIGKAGPGAPLSASQIASELSTHSNDNLVSSLLLDCMLRVLASHSILTCSITHQNDGHVLRLYGLAPVSKYFVRNQDGGTLAPVLDFMQDKVIMSVWDHLKDAVLEGVLPFYRAHGVDMSNEFKGKEGRLRETSMGYMKGFNQVFMEKILETYKGFEGLKSLVDVGGGDGTILNSIISKYPSIKGVNFDLASVIEKSPPYPGIEHVAGDMFVNIPKGDAIFMKWIIHQHVDKDSLKVLKNCYEALPERGKVILVDMVVPEASETSAAHKSLFQLYLFLLNSNSHGKERTEREFESLGKAAGFSSIQVAGFAYGFSVVEFYKTM

>cds.Jr09_08970_p1

MSSKESQMGTSSTEDQESISEYAMQLTSASVLPSVLKAAIDLGVLEIIGKAGPGALLSASQIASELSTHSNDNLVSSLLLDCMLRVLACHSILTCSITHQNDGHVLRLYGLAPVSKYFVPNQDGGTLAPVLDFMQDKDIMSIWDHLKDAVLEGVLPFYRAHGVDMSNEFKGKEGRLRETSMGRMKSFNQVFMEKILETYKGFEGLKSLVDVGGGDGTILNSIISKYPSIKGVNFDLASVIEKSPPYPGIEHIVGDMFVNIPKGDAIFMKWIIHQHVDKDSLKVLKNCYEALPERGKVILVDMVVPEVSETSAAHKSLFQLYLFLLNSNSHGKERTEREFESLGKVAGFSSIQVAGFAYGFSVVEFYKTM

>cds.Jr13_09640_p1

MENTQRSEWDEEEKQAEVSIWKYIFGFTEMALVKCAIELGIADTIESHGGGPMTLSELSSTLGCDPSPLYRVMRFLTHRGIFKEMPTTLGSPGYAQTRLSSRLLRNGEHSVAALILFESSPVMLAPWHSLSARVLAHGTAPFDVVHGEDIWRYAAENPGYSRLLNEAMACEARLAVPAILQGCPEVFDGLSSLVDVGGGNGTTLQLLVKSCQWIRGINFDLPHVVSDAAEFPGIEHVGGDMFASVPKADAAFLMRVLHDWGDNECIQILKKCREAIPEDKGKVIIVEAVIDQEAKISDKLRDARLALDMIMMAHTTMGKERTLEEWGLVLGKAGFSRYRAKPIRAVQSVIEAFP

>cds.Jr13_09690_p1

MENTQRSEWDEEEKQAEVGIWKYIFGFTEMALVKCAIELGIADTIESHGGGPMTLSELSSTLGCDPSPLYRVMRFLTHRGIFKEMPTTQGSPGYAQTRLSSRLLRNGEHSVAALILFESSPVMLAPWHSLSARVLAYETAPFDVVHGEDIWRYAAENPGHSRLLNEAMACDARLVVPAILQGCPEVFDGLSSLVDVGGGNGMTLQLLVKSCQWIRGINFDLPHVVSDAAEFPGIEHVGGDMFASVPKADAAFLMWVLHDWGDNECIQILKKCREAISEDKGKVIIVEAVIDQEAKISDKLRDARLALDMIMMAHTTTGKERTLEEWGLVLGKAGFSRYRAKPIRAVQSVIEAFP

>cds.Jr10_15840_p1

MGRWNSRWRGIRGCEKQTEWEEIRAVTVGVKKHFETKRVVIDKCSAVFGGLESLVDVGGGTGTVAKAIADAFPSMECTVLDLPHVVAGSEGSKNLKYIGGDMFEVVPPADAILLKVNASLIKLAYTNQWILHDWNDEECVKILRRCKEAITSNDKKGKVMIIEMIVQNQKEDDKESIETQLFFDILIMVLVTGKERNEKQWAKLFFDAGFSDYKITHILGLSSLIEVYP

>cds.Jr_Scaffold_205_00010_p1

MEVQEAGDQLSSSQLGGLVSIPMVLRAAIELNVFNIIADAGSDAYLSAAEITSKIPTTDPNSIISLNPHVRGLNFDLPQVIADAPTLPGVKHAAGDMFESIPNAQTILLKRILHNWDDERCKRLLRNCWEALPADGKVIVVEMAIPQELENNLETMNVLKEDLYMMLLMAGGKERTLAEFDHLAKAVGFTKMKVFPMPHGSVHVIELYK

>cds.Jr04_07740_p1

IISLNPHVRGLNFDLPQVIADAPTLPGVKHAAGDMFESIPNAQTILLKRILHNWDDERCKRLLRNCWEALPADGKVIVVEMAIPQELENNLETMNVLKEDLYMMLLMAGGKERTLAEFDHLAKAVGFTKMKVFPMPHGSVHVIELYK

>cds.Jr06_10640_p1

MDPRFNKVFNTAMHNHTTMLFKKILESYKGFEPLKQLDQGVEHVGGDMFKSVPKGDAIFMKGILHDWGDEQCLKLLKNCSTSIPNDGKVIIVEAFLPMMPEVCTCMKRNSQLDVLMMAQHPGGKERTQQEFEALATKAGFKGIKYECLVYSFQVMEFFK

>cds.Jr07_09870_p1

MMASLTESHSLNNLDGDQKEEDESFLYAGQLVNSLVLPMALHTAIELGVFEILAKAGHGAKLSSSQIVAEMPTTNPEAPQMLDRILRMLASHSVVRCSAGADDAGIFQRLYALSPVSKQFIPDEDGISFGHLMALFQDKVFVDSWFQLKGAILEGGIPFNRVHGINAFEYPGLDPKFNQVFNKAMLYPTTMVIKKILECYKGFETLKQLVDVGGGLGVSLNLITSKYTHIKGINFDLPHVIQHAPSYPGVEHVGGDMFKSVPKANVIFMKQFQMMGM

>cds.Jr04_07890_p1

MHIAYANIGKERYQKIEQVQNMEVQEDGDHFISSQLGGLASTQMALRAAIELKVFSIIADAGPDAHLSAAEIISKIPTKDPSSAEWTLERVLRVLGANSILSISRKPLGNNGEHGRHEWTYGLTKKSRCLVSSSSTTDELATFTTSLILFATEREMLESQYMIKDAVLDPGSSPFYKAYGVNFYDYMGEKPRLRQLFDEFMEVSSKLQFEDVFKLYGGFKDLKELMDVGGGIGTSLAKITSTYPHVRGLNFDLPHVIDAAPKLPGVKHVAGDMFKSIPNAQTILLKVSFYSWILGAIW

>cds.Jr06_10690_p1

SQLKDAILEGGIPFDRVHGTSAFEYLGVDPRFNKVFNTAMLNHTTMLFKKILESYKGFEPLKQLVDVGGGLGGALHSITSKYPHIKGINFDLPHVVQQAPSYP

>cds.Jr16_08670_p1

MGSFRQHSFKLVPGEHATELLNAQAHIWNHIFNFINSMSLKCAIQLGIPDIIHNHAKPMTLSELLVALPIHPTKARNIPRLMRILIHSGFFVAEEMIGNDEEERYALTDASRLLLKENPLSVTPFLLAMLDPVLTKPWHFLTAWFQNEDLTPFDTAHGKMFWDYGGHEPKLNNIFSDAMASDARLVMSVVIDKCSAVFGGLESLVDVGGGTGTMAKAIADAFPSMECTVLDLPHVVAGLEGSKNLKYIGGDMFEAVPPADAILLKVNASLIELAIQT

>cds.Jr04_07970_p1

MEVQEDGDHFITSQLGGLASTQMALRAAIELKVFSIIADAGPDAHLSAAEIISKIPTKDPSSAAWTLERVLRVLGANSILSIISRKPLGNNGEHGRHEWTYGLTKKSRCLVSSCTTDELATFTTSIILFTTEREVLESQYMIKDAVLDPGSSPFYKANGVNFYDYMGEKPRLRQVFDEFMEVSSKLPFEDVFKLYGGFKDLKELMDVGGGIGTILAKITSTYPHVRGLNFDLPHVIDAAPKLPGVKHVAGDMFKSIPNAQTILLKVFFLRNATKKPYTTRSHLINM

>cds.Jr_Scaffold_661_00050_p1

MFKSVPKGDAIFMKGILHDWGDEQCLKLLKNCSTSIPNDGKVIIVEAFLPMMPEVSTCMKRNSQLDVLMMAQHPGGKERTQQEFEALATKAGFKGIKYECLVYSFQVMEFFK

>cds.Jr04_07950_p1

MEVQEDGDHFISSQLGGLASTQMALRAAIELKVFSIIADAGPDAHISAAEIISKIPTKDPSSAAWTLERVLRVLGANSILSISRKPLGNNGEHGRQEWTYGLTKKSRCLVIRSGTTDELANFTTSFILFATEREMLESQYMIKDAVLDPGSSPFYKAYGVNFYDYMGEKPRLRQLFDEFMEVSSKLQFEDVFKLYGGFKDLKELMDVGGGIGTSLAKITSTYPHVRGLNFDLPHVIDAAPKLP

>cds.Jr09_08990_p1

MLDLMQDKDIMSIWDHLKDAVLEGVLPCYKSHGMNMSEFLGKDARFHETFKGLMKSLSPTLMEKILETYKGFEGLKSLVDVGGHDGTILNIIISKYPSIKGVNFDMASVIEKSPPYPGMDFNIWVSSTSYKSIETITFHFSS

>PSS19510 pep chromosome:Red5_PS1_1.69.0:LG10:13610913:13614144:1 gene:CEY00_Acc11603 transcript:PSS19510 gene_biotype:protein_coding transcript_biotype:protein_coding description:Caffeic acid 3-O-methyltransferase

MAEETRERRNSARLAILELANMISVPMSLNAVVRLNVADAIWQGGSNTPLSRPPLRRPGQPPAHPPHALQYSLTDIGKTLVTDDEGLSYGAYVLQHHQDALMRAWPLVHEAVADPSTEPFVKANGEPAYSYYGKKLEMNGLMKKAMSGVSVPFMKAILDGYDGFGGVDRLVDVGGSAGDCLRMILQKHPNVKEGVNYDLPEVVAKAPIIPGVTHVGGDMFKSIPTGDAIFMKWVLTTWTDEECKLIMKNSYKALPAGGKLIACEPVLPKLTDDSHRTRALLEGDIFVMTIYRTKGKHRTEEEYRQLGLLAGFPHFRALYIDYFYTLLEFQK

>PSS14478 pep chromosome:Red5_PS1_1.69.0:LG13:15860726:15862145:1 gene:CEY00_Acc15046 transcript:PSS14478 gene_biotype:protein_coding transcript_biotype:protein_coding description:Trans-resveratrol di-O-methyltransferase

MLDPTLTKPWHFVSEWFKNHDPTPFDTAHGRPLWDYAGHEPRMNHFFNEAMASDSRLVTSVLVKDCKAIFEGLNSLVDVGGGTGTVAKAIAEAFPNLECTVLDLPHVVADLQGSCKNLTYVGGDMLEVIPSADAVLLKWILHDWSDEDSVKILKRCKEAIPSRGKVIIIDMKVENQKGDDHDQSIETQLFFDMLMMTLTPGRERTEKEWAKLFSDAGFSDYKIIPILGLRSLIEVYP

>PSS12132 pep chromosome:Red5_PS1_1.69.0:LG14:17477518:17478984:-1 gene:CEY00_Acc16343 transcript:PSS12132 gene_biotype:protein_coding transcript_biotype:protein_coding description:(R,S)-reticuline 7-O-methyltransferase

MEGETNREEEEIQAQVDIWKYVFGFVDMAVVKCAIELGIAEVVESHEGPVTISELSSALGCSQSSLYRIMRFLVHRKFFKEDHNTNQGAIGYVQTPMSRLLLRNGEKSMAAFVLLESSPVMLAPWHGLSARVLAKGDLAFESTHGKDIWSYAATNPAHSKLINDAMACDARVAVAAIIDGCPDLFNGMDSVVDVGGGDGTTLRLLVKACPWIRGINFDLPHVVSEAPACDGIEHIGGDMFVSVPKADATLLMWVLHDWGDDECINVLRKCKEAIPKDKGKVIIIEGVVEEEEEDKLKYVRLMLDMVMMAHTNTGKERTSKEWVHLLIGAGFSRYTIKRIKAVQSIIEAYP

>PSS11908 pep chromosome:Red5_PS1_1.69.0:LG14:13951197:13953369:-1 gene:CEY00_Acc01300 transcript:PSS11908 gene_biotype:protein_coding transcript_biotype:protein_coding description:Caffeic acid 3-O-methyltransferase

MTTFEYHGTNPRSNKLFNQGMSNLSTITMKKVLDSYQGFEGLTTIVDVGGGTGASLNMILSKYPTIKGINFDLPRDFCICGVSNSGIGFGAGIPGPRSGRGSGLQQNPRRGSGRGSGLKKISGIGDGDTNLRPRPAPLPSLVGGDMFVSVPNGDAIFMKWICHDWSNEHSSKFLKNCYNALPNNGKVIVAEYILPVALDTSLAMKSVVDMDIIMLAYSQGGKERTEKEFEALAKGAGFEGFRVMCCAFNTYVMEFLKKI

>PSS04505 pep chromosome:Red5_PS1_1.69.0:LG18:13044644:13047850:-1 gene:CEY00_Acc20359 transcript:PSS04505 gene_biotype:protein_coding transcript_biotype:protein_coding description:Caffeic acid 3-O-methyltransferase

MGSITTNQTVAIRSEEEEACLFALQLANASVLPMVLKSAIELDLLEIMAKAGPGAYVSPSELAAQLSTTNPDAPAMIDRILRLLTSYSVLKCDLRDLPDGGVERLYGLEPVCKFLTRNADGVSLAPLALMTQDKVLMQSWYHLKDAVVDGGIPFNKAYGMSAFEYHGTDPRFNKVFNQGMSNHSTFTMKKILETYHGIESLTTLVDVGGGIGATLNMIVSKYPSLKGINFDLPHVIEDAPTYPGVEHVGGDMFISVPKGDAIFMKWICHNWSDEHCLKFLKNCYDSLPDDGKVIISECILPVSPNTELATKTVFHLDIIMLSHYPGGKERTEKEFEALAKGAGFEGFRVACYVFNTYIIEFLKKI

>PSS32943 pep chromosome:Red5_PS1_1.69.0:LG3:7186730:7187815:1 gene:CEY00_Acc03239 transcript:PSS32943 gene_biotype:protein_coding transcript_biotype:protein_coding description:Caffeic acid 3-O-methyltransferase

MFVSVPKGDAIFMKWICHDWSDEHCLKFLKNCYDSLPDDGKVIISECILPVAPDTGLATKTVFHIDIIMLAHNPGGKERTEKEFEALAKGAGFEGFRVACCAFGTYIMEFLKKI

>PSS32944 pep chromosome:Red5_PS1_1.69.0:LG3:7191631:7193152:1 gene:CEY00_Acc03240 transcript:PSS32944 gene_biotype:protein_coding transcript_biotype:protein_coding description:Flavone 3'-O-methyltransferase

MIRYHLKDAVVDGGIPFNKAYGMSAFEYHGTDPRFNKVFNQGMYHLKDAVVDGGIPFNKAYGMSAFEYHGTDPRFNKVFNQGMSNHSTFTMKKILETYHGFEGLTTLVDVGGGIGATLNMIVSKYPTVRGINFDLPHVIEDAPT

>PSS32279 pep chromosome:Red5_PS1_1.69.0:LG3:306049:309984:1 gene:CEY00_Acc02578 transcript:PSS32279 gene_biotype:protein_coding transcript_biotype:protein_coding description:Caffeic acid 3-O-methyltransferase

MGSTSEAQITPTDQEEAFLFAMQLASASVLPMVLKSAIELDLLEAIARAGPAAYVSPSDLAAQLSTTNPHAAVMLDRILRLLTSYSVLHCTLRTLPNGGVERLYGLAPVCKFLIKNEDGVSMAPLLLMNQDKVLMESWYHLKDAVLDGGIPFNKAYGMTAFEYHGTDTRFNKVFNQGMSNHSTITMKKIMDSYQGFEGLNTIVDVGGGTGATLNMILSKYPAIKGINFDLPHVIEDAPSYPGVEHVGGDMFVSVPNGDAIFMKWICHDWSDEHCSKFLKNCYDALPNNGKVIVAECILPIAPDTSLATKNVVHIDVIMLAHNPGGKERTEKEFEALAKGAGFEGFRVMCCAFNTHVMEFLKKI

>PSS31563 pep chromosome:Red5_PS1_1.69.0:LG4:5292021:5295031:-1 gene:CEY00_Acc07226 transcript:PSS31563 gene_biotype:protein_coding transcript_biotype:protein_coding description:(RS)-norcoclaurine 6-O-methyltransferase

MASFLLLQSSPALLATWHGLSARVLTNGPSAFETTHGKDIWSYVETNPALSKLFNESMACDARRVVPEIINGCPGLLDGLDSLVDVGGGNGTTMRLLVKACDWIRDINFDLPHVVSDAPACVRVEHVGGDMFDSVPNADAAFLMWVLHDWGDDECIRILKNCREAISKDKRKVIIVEAVVEEKEDKLGHVRLMLDMVMMAFTNNGKQRTSKEWAHLLREAGFSRYTVTQIKSVKSVIEAYP

>AT1G21100.1 (AtASMT1)

MGYLFQETLSSNPKTPIVVDDDNELGLMAVRLANAAAFPMVLKAALELGVFDTLYAAASRTDSFLSPYEIASKLPTTPRNPEAPVLLDRMLRLLASYSMVKCGKALSGKGERVYRAEPICRFFLKDNIQDIGSLASQVIVNFDSVFLNTWAQLKDVVLEGGDAFGRAHGGMKLFDYMGTDERFSKLFNQTGFTIAVVKKALEVYEGFKGVKVLVDVGGGVGNTLGVVTSKYPNIKGINFDLTCALAQAPSYPGVEHVAGDMFVDVPTGDAMILKRILHDWTDEDCVKILKNCWKSLPENGKVVVIELVTPDEAENGDINANIAFDMDMLMFTQCSGGKERSRAEFEALAAASGFTHCKFVCQAYHCWIIEFCK

>AT1G21110.1 (AtASMT2)

MGYLFEETLSSNPKTPIVVDDDNELGLMAVRLANAAAFPMVLKASLELGVFDTLYAEASRTDSFLSPSEIASKLPTTPRNPGAPVLLDRMLRLLASYSMVKCEKVSVGKEQRVYRAEPICRFFLKNNIQDIGSLASQVIVNFDSVFLNTWAQLKDVVLEGGDAFGRAHGGMKLFDYMGTDERFSKLFNQTGFTIAVVKKALEVYQGFKGVNVLVDVGGGVGNTLGVVTSKYPNIKGINFDLTCALAQAPTYPGVEHVAGDMFVDVPTGNAMILKRILHDWTDEDCVKILKNCWKSLPQNGKVVVIELVTPDEAENGDINANIAFDMDMLMFTQCSGGKERSRAEFEALAAASGFSHCQFVCQAYHCWIIEFCK

>AT1G21120.1 (AtASMT3)

MGYLFEETLSSNPKTPIVVDDDNELGLMAVRLANAAAFPMVLKASLELGVFDTLYAEASRTDSFLSPSEIASKLPTTPRNPGAPVLLDRMLRLLASYSMVKCEKVSVGKGERVYRAEPICRFFLKNNIQDIGSLASQVIVNFDSVFLNTWAQLKDVVLEGGDAFGRAHGGMKLFDYMGTDERFSKLFNQTGFTIAVVKKALEVYQGFKGVNVLVDVGGGVGNTLGVVTSKYPNIKGINFDLTCALAQAPSYPGVEHVAGDMFVDVPTGDAMILKRILHDWTDEDCVKILKNCWKSLPENGKVVVIELVTPDEAENGDINANIAFDMDMLMFTQCSGGKERSRAEFEALAAASCFTHCKFVCQAYHCWIIEFCK

>AT1G21130.1 (AtASMT4)

MGYLLEETLSSNSKTPIVIDDDNELGLMAVRLANAAAFPMVLKAALELGVFDTLYAEASRSDSFLSPSEIASKLPTTPRNPEAPVLLDRMLRLLASYSVVKCGKVSEGKGERVYRAEPICRFFLKDNIQDIGSLASQVIVNFDSVFLNTWAQLKDVVLEGGDAFGRAHGGMKLFDYMGTDERFSKLFNQTGFTIAVVKKALEVYQGFKGVNVLVDVGGGVGNTLGVVASKYPNIKGINFDLTCALAQAPSYPGVEHVAGDMFVDVPTGDAMILKRILHDWTDEDCVKILKNCWKSLPESGKVVVIELVTPDEAENGDINANIAFDMDMLMFTQCSGGKERSRAEFEALAAASGFTHCKFVCQAYHCWIIEFCK

>AT1G33030.1 (AtASMT5)

MEEQNLSSYAMILSSSSVLPMVLKTAIDLGLFDILAESGPSSASQIFSLLSNETKKHHDSSLVNRILRFLASYSILTCSVSTEHGEPFAIYGLAPVAKYFTKNQNGGGSLAPMVNLFQDKVVTDMWYNLKDSVLEGGLPFNNTHGSSAVELVGSDSRFREVFQSSMKGFNEVFIEEFLKNYNGFDGVKSLVDVGGGDGSLLSRIISKHTHIIKAINFDLPTVINTSLPSPGIEHVAGDMFTNTPKGEAIFMKWMLHSWDDDHCVKILSNCYQSLPSNGKVIVVDMVIPEFPGDTLLDRSLFQFELFMMNMNPSGKERTKKEFEILARLAGFSNVQVPFTSLCFSVLEFHKNK

>AT1G51990.1 (AtASMT6.1)

MISLQTSGGSSEEEDMLLAIQLGGLNFVPYIVKTARELDLFEIMAKARPLGSYLSPVDLASMAAPKNPHAPMMIDRLLRFLVAYSVCTCKLVKDEEGRESRAYGLGKVGKKLIKDEDGFSIAPYVLAGCTKAKGGVWSYLTEAIQEGGASAWERANEALIFEYMKKNENLKKIFNESMTNHTSIVMKKILENYIGFEGVSDFVDVGGSLGSNLAQILSKYPHIKGINFDLPHIVKEAPQIHGVEHIGGDMFDEIPRGEVILMKWILHDWNDEKCVEILKNCKKALPETGRIIVIEMIVPREVSETDLATKNSLSADLTMMSLTSGGKERTKKEFEDLAKEAGFKLPKIIYGAYSYWIIELYPN

>AT1G51990.2 (AtASMT6.2)

MISLQTSGGSSEEEDMLLAIQLGGLNFVPYIVKTARELDLFEIMAKARPLGSYLSPVDLASMAAPKNPHAPMMIDRLLRFLVAYSVCTCKLVKDEEGRESRAYGLGKVGKKLIKDEDGFSIAPYVLAGCTKAKGGVWYNVQHAIQEGGASAWERANEALIFEYMKKNENLKKIFNESMTNHTSIVMKKILENYIGFEGVSDFVDVGGSLGSNLAQILSKYPHIKGINFDLPHIVKEAPQIHGVEHIGGDMFDEIPRGEVILMKWILHDWNDEKCVEILKNCKKALPETGRIIVIEMIVPREVSETDLATKNSLSADLTMMSLTSGGKERTKKEFEDLAKEAGFKLPKIIYGAYSYWIIELYPN

>AT1G62900.1 (AtASMT7)

MRVFELIGSNEQFAEMFNRTMSEASTLIMKKVLEVYKGFEDVNTLVDVGGGIGTIIGQVTSKYPHIKGINFDLASVLAHAPFNKGVEHVSGDMFKEIPKGDAIFMKWILHDWTDEDCVKILKNYWKSLPEKGKVIIVEVVTPEEPKINDISSNIVFGMDMLMLAVSSGGKERSLSQFETLASDSGFLRCEIICHAFSYSVIELHK

>AT1G63140.1 (AtASMT8.1)

MENHLQHSLTIIPKPDLIKEEQRYHEDTVSLQAERILHAMTFPMVLKTALELGVIDMITSVDDGVWLSPSEIALGLPTKPTNPEAPVLLDRMLVLLASHSILKYRTVETGDNIGSRKTERVYAAEPVCTFFLNRGDGLGSLATLFMVLQGEVCMKPWEHLKDMILEGKDAFTSAHGMRFFELIGSNEQFAEMFNRAMSEASTLIMKKVLEVYKGFEDVNTLVDVGGGIGTIIGQVTSKYPHIKGINFDLASVLAHAPFNKGVEHVSGDMFKEIPKGDAIFMKVSRK

>AT1G63140.2 (AtASMT8.2)

MENHLQHSLTIIPKPDLIKEEQRYHEDTVSLQAERILHAMTFPMVLKTALELGVIDMITSVDDGVWLSPSEIALGLPTKPTNPEAPVLLDRMLVLLASHSILKYRTVETGDNIGSRKTERVYAAEPVCTFFLNRGDGLGSLATLFMVLQGEVCMKPWEHLKDMILEGKDAFTSAHGMRFFELIGSNEQFAEMFNRAMSEASTLIMKKVLEVYKGFEDVNTLVDVGGGIGTIIGQVTSKYPHIKGINFDLASVLAHAPFNKGVEHVSGDMFKEIPKGDAIFMKWILHDWTDEDCVKILKNYWKSLPEKGKVIIVEVVTPEEPKINDISSNIVFGMDMLMLAVSSGGKERSLSQFETLASDSGFLRCEIICHAFSYSVIELHK

>AT1G76790.1 (AtASMT9)

MGHLIPQTGDEETELGLAAVRLANCAAFPMVFKAAIELGVIDTLYLAARDDVTGSSSFLTPSEIAIRLPTKPSNPEAPALLDRILRLLASYSMVKCQIIDGNRVYKAEPICRYFLKDNVDEELGTLASQLIVTLDTVFLNTWGELKNVVLEGGVAFGRANGGLKLFDYISKDERLSKLFNRTGFSVAVLKKILQVYSGFEGVNVLVDVGGGVGDTLGFVTSKYPNIKGINFDLTCALTQAPSYPNVEHVAGDMFVDVPKGDAILLKRILHDWTDEDCEKILKNCWKALPENGKVIVMEVVTPDEADNRDVISNIAFDMDLLMLTQLSGGKERSRAEYVAMAANSGFPRCNFVCSAYHLWVIELTKQA

>AT1G77520.1 (AtASMT10)

MTNHLQDPLPTYPKPVLTKEEQEVDEKMVSLQAESIVNTVAFPMVLKAAFELGVIDTIAAAGNDTWLSPCEIACSLPTKPTNPEAPVLLDRMLSLLVSHSILKCRMIETGENGRTGKIERVYAAEPVCKYFLRDSDGTGSLVPLFMLLHTQVFFKTWTNLKDVILEGRDAFNSAHGMKIFEYINSDQPFAELFNRAMSEPSTMIMKKVLDVYRGFEDVNTLVDVGGGNGTVLGLVTSKYPHIKGVNFDLAQVLTQAPFYPGVEHVSGDMFVEVPKGDAVFMKWILHDWGDEDCIKILKNCWKSLPEKGKIIIVEFVTPKEPKGGDLSSNTVFAMDLLMLTQCSGGKERSLSQFENLAFASGFLRCEIICLAYSYSVIEFHK

>AT1G77530.1 (AtASMT11)

MSNHLQDPLTTYPKPGLTKEEQEIDEKMVSLQAESIVNAVAFPMVLKAALELGVIDTIAAASNGTWLSPSEIAVSLPNKPTNPEAPVLLDRMLRLLVSHSILKCCMVESRENGQTGKIERVYAAEPICKYFLKDSDGSGSLSSLLLLLHSQVILKTWTNLKDVILEGKDAFSSAHDMRLFEYISSDDQFSKLFHRAMSESSTMVMKKVLEEYRGFEDVNTLVDVGGGIGTILGLITSKYPHIKGVNFDLAQVLTQAPFYPGVKHVSGDMFIEVPKGDAIFMKWILHDWGDEDCIKILKNCWKSLPEKGKVIIVEMITPMEPKPNDFSCNTVLGMDLLMLTQCSGGKERSLSQFENLAFASGFLLCEIICLSYSYSVIEFHK

>AT3G53140.1 (AtASMT12)

MENESSESRNRARLAIMELANMISVPMSLNAAVRLGIADAIWNGGANSPLSAAEILPRLHLPSHTTIGGDPENLQRILRMLTSYGVFSEHLVGSIERKYSLTDVGKTLVTDSGGLSYAAYVLQHHQEALMRAWPLVHTAVVEPETEPYVKANGEAAYAQYGKSEEMNGLMQKAMSGVSVPFMKAILDGYDGFKSVDILVDVGGSAGDCLRMILQQFPNVREGINFDLPEVVAKAPNIPGVTHVGGDMFQSVPSADAIFMKWVLTTWTDEECKQIMKNCYNALPVGGKLIACEPVLPKETDESHRTRALLEGDIFVMTIYRTKGKHRTEEEFIELGLSAGFPTFRPFYIDYFYTILEFQK

>AT4G35150.1 (AtASMT13)

MEESKRNLLDEEAKASLDIWRYVFGFADIAAAKCAIDLKIPEAIENHPSSQPVTLSELSSAVSASPSHLRRIMRFLVHQGLFKEVPTKDGLATGYTNTPLSRRMMITKLHGKDLWAFAQDNLCHSQLINEAMACDARRVVPRVAGACQGLFDGVATVVDVGGGTGETMGILVKEFPWIKGFNFDLPHVIEVAQVLDGVENVEGDMFDSIPASDAVIIKWVLHDWGDKDCIKILKNCKEAVLPNIGKVLIVECVIGEKKNTMIAEERDDKLEHVRLQLDMVMMVHTSTGKERTLKEWDFVLTEAGFARYEVRDFDDVQSLIIAYRS

>AT4G35160.1 (AtASMT14)

MSSDQLSKFLDRNKMEDNKRKVLDEEAKASLDIWKYVFGFADIAAAKCAIDLKIPEAIENHPSSQPVTLAELSSAVSASPSHLRRIMRFLVHQGIFKEIPTKDGLATGYVNTPLSRRLMITRRDGKSLAPFVLFETTPEMLAPWLRLSSVVSSPVNGSTPPPFDAVHGKDVWSFAQDNPFLSDMINEAMACDARRVVPRVAGACHGLFDGVTTMVDVGGGTGETMGMLVKEFPWIKGFNFDLPHVIEVAEVLDGVENVEGDMFDSIPACDAIFIKWVLHDWGDKDCIKILKNCKEAVPPNIGKVLIVESVIGENKKTMIVDERDEKLEHVRLMLDMVMMAHTSTGKERTLKEWDFVLKEAGFARYEVRDIDDVQSLIIAYRS

>AT5G37170.1 (AtASMT15)

MTNHHQESLTTYPKPGPTREQEQVDEEMMSMQMQALRITNSLAFPMGVWLSPSEIAFGLPTKPTNPEAPMLIDRMLRLLVSHSILKCRLVETGENNRTESTQRVYAAEPDTSEGCDTRRKRCIQFCPWHGTLRIRCTDEQFAAIFNQAMSDSSTMIMTKILEVYKGLKDVNTLVDIGGGLGTILNLVISSKYPQIKGINFDLAAVLATAPSYPGVEHVPGDMFIDVPKGDAIFMRRILRDWNDKDCVKILTNCWKSLPEKGKVIIVDMVAPSEPKSDDIFSKVVFGTDMLMLTQCSCGKVRSFAQFEALASASGFHKCEVSGLAYTYSVIEFHK

>AT5G53810.1 (AtASMT16)

MANHLQVPLTKPDRVKEEQEVEEEARLLARRLANAAASPMVLKAALELGVIDTITTVGGGDLWLSPSEIALRLPTKPCNLEAPALLDRMLRFLVSHSVLKCRTVIEENGQTGKVERVYAAEPVCKYLLNKSDDVSGSFASLFMLDLSDVFIKTWTHLEDVILEGRDAFSSAHGMKLFEYIQADERFGKVFNRAMLESSTMVTEKVLKFYEGFKDVKTLVDVGGGLGNTLGLITSKYPHLIGINFDLAPVLANAHSYPGVNHVAGDMFIKIPKGDAIFMKWILHDWTDEQCVAILKNCWKSLEENGKLIIVEMVTPVEAKSGDICSNIVFGMDMTMLTQCSGGKERDLYEFENLAYASGFSRCAIVCAVYPFSVIEIYK

>AT5G54160.1 (AtASMT17)

MGSTAETQLTPVQVTDDEAALFAMQLASASVLPMALKSALELDLLEIMAKNGSPMSPTEIASKLPTKNPEAPVMLDRILRLLTSYSVLTCSNRKLSGDGVERIYGLGPVCKYLTKNEDGVSIAALCLMNQDKVLMESWYHLKDAILDGGIPFNKAYGMSAFEYHGTDPRFNKVFNNGMSNHSTITMKKILETYKGFEGLTSLVDVGGGIGATLKMIVSKYPNLKGINFDLPHVIEDAPSHPGIEHVGGDMFVSVPKGDAIFMKWICHDWSDEHCVKFLKNCYESLPEDGKVILAECILPETPDSSLSTKQVVHVDCIMLAHNPGGKERTEKEFEALAKASGFKGIKVVCDAFGVNLIELLKKL

>Caril.10G053100

MDLVNGQGASDEQSRLQSHLYKNMLGFTDSMSLYCAIQLGIPDIIHNHGQPITLKQLASKLRIHPQKTSCMHRLMRLLVYSGFITKTAAVVHEDQEEEEAYALTASSRVVLKDKVTGLSPLVQAILDPAVVNSWYSLGDWFRGTELTPFVKAHGMGFFDYCNQNQEYGITFDEGMASDSRLMSKVIKDYKTIFEGLDSLVDVGGGSGTVSRIISEAFPHIKCTVFDLPQVVANLPDDKNLKYVGGDMFQSIPAADAILMKWILHDWSDEECINILKRCKEAIRSKGKDGKIIIIDVVINEEEEEHDITKAKLFLDALMMILLTGKERTKKEWEKLFLDAGFSHYKIVASYGLKSLIEVYPN*

>Caril.05G088800

MESKREKEMGSTQRSEWDEEEEQAEVDIWKYMFGFTEMAVVKCAVELGIADTIESHGGGPMTLSELSSTLGCDPSPLYRVMRFLTHRGIFKEMPTTQGSPGYGQTRLSSRLLRNGEHSMAALILLHNSPVMLAPWHSLSAHVLAYETAPFDVVHGEDIWRYAAENPGHSRLFNEAMACDARLVVPAMLQGCPEVFDGLSSLVDVGGGNGTTLQLLVKSCQWIRGINFDLPHVVSDAAEFPGIEHVGGDMFATVPKADAAFVKWVLHIWGDKECIQILKKCRESIPEGKGKVIIVEAVLDQEAKISDKLTEARLALDMIVMAHTTTGKERTLEEWGLVLEKAGFSKYTVKPIRAVQSVIEAFP*

>Caril.05G088700

MENTKRTEWDEEEEQAEVSIWKYIFRFTEMAVVKCAIELGIADTIESHGGGPMTLSELSSTLGCDPSPLSRVLRFLTHRGIFKEMPTTKGSPRYAQTRLSSRLLRKGEHSMAALILFESRPVMLAPWHRLSARVLAYETAPFEVVHGEDIWRYAAENPGHSRLINEAMACDARLVVPAMLQGCPEVFDGLSSLVDVGGGNGTTLQLLVKSCQWIRGINFDLPHVVSDAAEFPGIERVGGDMFASVPKADAAFLKNVLHDWGDNECIQILKKCRESIPEGKGKVIIVEAVIDQEAKISDKLTDVRLALDMIMMAHTTTGKERTLEEWGLVLGKAGFSKYTVKPIRAVQSVIEAFP*

>Caril.05G088600

MENTQRTEWDEEEEQAEVGIWKYIFGFTEMAVVKCAIELGIADTIESHGGGPMTLSELSSTLGCDPSPLSRVMRFLTHRGIFKEMPTTKGSPGYAQTRLSSRLLRNGEHSMAALILLESSPVMLAPWHSLSAHVLAYETAPFDVVHGEDIWRYAAENPGHSRLINEAMACNARLVVPAILQVCPEVFDGLSSLVDVGGGNGTTLQLLVKSCQWIRGINFDLPHVVSDAAEFPGIEHVGGDMFASVPKADAAFLKQWVLHDWGDNECIQILKKCRESIPEGKGKVIIVEAVIDQEAKISDKLTAVRLALDMMMMAHTTTGKERTLEEWGLVLGKAGFSKYTVKPIRAVQSVIEAFP*

>Caril.05G088500

MENTHRTEWDEEEEQAEVGIWKYIFGFTEMAVVKCAIELGIADTIESHGGGPMTLSELSSTLGCDPSPLSRVMRFLTHRGIFKEMPTTKGSPGYAQTRLSSRLLRNGEHSMAALILLESSPVMLAPWHSLSARVLAYETAPFDVVHGEDIWRYAAENPGHSRLINEAMACNARLVVPAILQGCPEVFDGLSSLVDVGGGNGTTLQLLVTSCQWIRGINFDLPHVVSDAAEFPGIDYVGGDMFASVPKADAAFINNVLHDWGDNECIQILKKCRESIPEGKGKVIIVEAVIDQEAKISDKLTDVRLALDMIMMAHTTTGKERTLEEWGLVLGKAGFSKYTVKPIRAVQSVIEAFP*

>Caril.09G064200

MDLIHAQGEGEREQFQAQSHLYKHVFNYIGSMSLKCAVQLGIPDIIYNHGQPITLPELVSKLQIHPSKAGFVPRLMRLLVHSGLFAATVRVKNQEEEEAYDLTPSSRVLIKDQVSNLSPFVLAMINPALVTPCHDLGNWLQGEKVTPFESVHGMSFWDYNDQNPEFNSLFNEAMASDSGMMNFIIKDCKAVFEGLDTLVDVGGGTGTCARIISEEFPHLKCTVFDLPHVVANLAADSLKLNYVAGDMFESIPSADALLLKLVLHGWSDEHCVKILKKCREAISKNGKGEGKVIIIDVVINEKKEEHEMTEAKLLFDMLMMVVTSGKERDEKDFKKLFLEAGFNRYKITPIYGLRYLNLPHTTVMGFENNDKVAWVERIMQIDRRDIGTALSVISSNISAATFLASISLTLCSLIGAWMANSSNYFMQGLVYGDTRPSTMSIKYISLLICFLLAFSCFVQSARNFVHANYLLSTPDSNIPVRNVELVILRGGDFWSLGLRALYFALDILLWFFGPIPMFVSSIVMVIIFHYLDTNTTLLHKHGSPQKQMVETVAV*

>Caril.09G064300

MDLVNCGQEASELFRVQSHLYKHLFSFIDSMSLGCAIQLGIPDIIHSHGQPMTLPQLVSKLHIDPKKTICVHRLMRLLSHSGFFTKTTTVHEDREEEEAYALTPSSRLVLKDDRTSLSPFVVAMLDPALVNPWYSLGDWFRGTELTPFAKAHGMGLWDYCNENPEYGDTFNEAMASDSQFMRLVVKDYKPIFEGLGSLVDVGGGTGTMARIISEAFPHIKCTVYDLPHVVANLPESSNFNYVGGDMFQSIPPADAIMFKWIMHDWNDEDCVNILKRCKEAITSKGYKEGNKVIIIDVVINEEKDDEDITKAKLLFDTLMMVLLPGKERTKKEWEKLFLEAGFSRYKIAASYGMKSLIEVYP*

>Caril.04G128700

MSGLRQPSFELVPEEHLAKLLNAQALIWNRIFNFINSMSLKCAIQLGIPDIIHNHGKPMTLSELLVALPIHPTKACNVSRLMRILIHSGFFVAEKIIGNDQEERYALTDASRLLLKDNPLSVTPFLIAMLDPVLTKPWHFLTAWFQNEDLTPFDTAHGKKFWDYGGHDPKLNHLFNDAMASDARLVMSVVIDKCSAVFSGLESLVDVGGGTGTVAKAIADAVPSMECTVLDLPHVVAGLEGSKKLKYIGGDMFEAVPPADAILLKWILHDWNDEECIKILGRCKEAITSNDKKGKVIIIDMIVQNQKEDDEESIETQLFFDMLMMVMATGKERNEKEWAKLFFDAGFSDYKITHILGLRSLIEVYP*

>Caril.04G128600

MSSFRMNSFKLIPGEHATELLNAQAHIWNHIFNFINSMSLKCAIQLGIPDIIHSHGKPMTLSELLVALPIHPTKACNVPRLMRILIHSGFFVAEKMFENDQEERYALTDASRLLLKDNPLSVTPFLVAMLDPVLTKPWHFLTAWFQNEDLTPFDTAHGKKFWDYGEHEAKLNHFFSDAMASDARFVVSVLIDKCSAVFDGLESLVDVGGVTGTVAKAIADAFPSMECTVLDLPHVVAGLEGSERLKYIGGDMFEAVPPADAILLKWILHDWNDEECIKILGRCKEAITSTDKKGKVIIIDMIVQNQKEDDEDRESIETQLFFDMLMMVLVTGKERNEKEWAKLFFDAGFSDYKTTHKLGLRSLIEVYP*

>Caril.04G128300

MSSFRQHSFKLVPGEHATELLNAQAHIWNHIFNFINSMSLKCAIQLSIPDIIHNHAKPMTLSELLVALPIHPTKARHIPRIMRILIHSGFFVAEETIGNDEEERYALTDASRLLLKENPLSVTPFLLAVLDPVLTKPWQFLTAWFQNEDLTPFDTAHGQMFWDYGCHEPRLNNFFNDAMASDARLVMSVVIDKCSAVFGRLESLVDVGGGTGTVAKAIADAFPSMECTVLDLPHVVACSEGSKNLKYIGGDMFEAVPPADAILLKWILHDWNDEECVKILRRCKEAIMSNGKKGKVIIIEMIVQNQKEDDKESIETQLFFDMLMMVLVTGKERNEKEWAKVFFDAGFSEYKITHILGLRSLIEVYP*

>Caril.03G167400

MERKEEVALLKGQAEIWQHLFAFADSMALKCAVELRIADIMHSHSVPITLSQIASAIDSPSPDIPYLSRIMRSLVYKKIFTEHHPSDGGEKLYGPTHTSRWLLHDAELTLAPMVLMENSQRQLAPWHFLSQCVKEGGIAFKKAYGVEMWDFAARNPEYNKIFNDAMACTTKIMMGVLLAEYKDGFGSIGSLVDVGGGTGEMIAEIIKQHPNINGFNFDLPHVVATAPVHEGVSHVGGNIFEAIPNANAILLKCVLHDWSDEHCIKILRNCKKTVPQKTGKVIIVDIVIEKDNDDLFQETRMVYDLLMMAHTTGGKERSELEWKQLLEEGGFPRYKIIKLPAITSIIEAYPE*

>Caril.03G167000

MELTTMEAMIQGEAKLWGNIFAFTDSMALRCVLELGIVDIIHSHGGPITLSQIAAGIDSPSLNIDNLARLMRLLVRKDIFTTHHQPSDCGEITLYGLTPHTSRWLMRGSEFSLVPFILMQSHPELMTSWLCLSQCVKEGGTAFSKVHGCEVRDFAHKSPKFNKMFNDAMACTAKLVAERILWGYRDGFSSINGSLVDVGGGTGEMVAKIVKAHPHIKAINFDLPHVITTAPMQLGVSHIEGNMFEAIPNADAILLKRVLHGFSDEDCIKILKNCAKAIPKKTGKIIIVEHVLEPNGNGPFDETGLVFDLIMMLLAPSGKERTELDWKKLLKEGGFPHHKIIKIPAFPSIIEAHPA*

>Caril.03G185200

MERKEEVALLKGQAEIWQLLFAFADAMALKCAVELRIADIIHSHSVPITLSQIASAIDSPSPDIPYLSRIMRSLVYKKIFTEHHPSDGGEKLYGPTHTSRWLLHDAELTLAPMVLMENSQRQLAPWHFLSQCVKEGGIAFKKAHGVEMWDFAARNPKFNKIFNDAMACTTKIMMGVLLAEYKDGFSSIGSLVDVGGGTGEMIAEIIKLHPHIKGFNFDFPHVVATAPVHEGVSHVGGNMFESIPNANAILLKCVLHDWSDEQCIKILRNCKKTVPQQTGKVIIVDIVIEKDNDDLFRETRMVYDLLMMAHTTGGKERSELEWQQLLEEGGFPRYKIIKLPAITSIIEAYPE*

>Cre03.g148800

MQPDVKPVLDLLNGFRASQTLLTAVQLGLFECLEHTNELSVDGLASALAHRLPGSPAPSLDGLDRLCRACVALGLLSSPANGQFALTDAARAYLLASAPQSLAGYCVHSSQVVWPLFGGLPAAVTTGSNVWQQQFGAPGSDVFARVYDTPGAVRRFMSGMHSFATLSAPAVVRAFDLSFATRLLDLGGATGALAAAACAAYPSLEEAVVVDLPHVLELAQRHFAPTAAPREPAAPAEVQGAGGGGGGGFGGGGDGGGSGGGGSGDCGCNGRLRWLAADFFTETDKLPQQVDLVVLSRILHDWDGPRCAQLLARVHGLLRPGGAVLVAEMLLQPDRLGPPAALLQDLNMLCQTHGRERSLAEYEQLLQAAGFVDVRGHVTGTYLDAVLARKPEQHL*

>Cre05.g240400

MMTWGGILCLNADRSFPMFTHLPHVLRHGELPPESKQSIPDILTTFGSDVAAAEFFAEGMTGASLGNFNLLARSFPFARYTSLGDLGGSSGCLACCVAAAHPHLTATTYDLAPVHAAAERHVRAQGLEGRVQVVDYDFFSPAAVPGRHDVIALGMVLHDWGLPRKMQLLRKAYAALPPGGALIAIDHLVDCRRAGSPLQLGMSLTMLLEFGAAESAFDYSYEEFCGWVREVGFSSTQLIDLVGTAKAAVAYK*

>Cre08.g365800

MEPPQQQPQQQQLARAPSRSPPSSAQDGNAAADSVSRAGSSSSSCGACCGLQTALSSPVLKAVRTVPADKAAALENLIRGSWAPNAIYCLCRLGVPDALGAAPAAALSPAELASRLHCSAPCLRRLLRLCAAYGLLVECASRGEGGGGWGGGEGGGGSGEGGKGGEGGEGGKQTRGDVGGVGGGGLTDGSQAEAGDVRAGAGKGEREEGEGRGVEEEGEQVERTASGGSGTSSSCTGASSSGGSSSSAGTAAATAATRSSSGSSADCGSSTATAAATAAATAIAAGASPAAATAGAAATCLEARTFFYLTDIGTMLQSSHPSCMHWLALMLGLPGHYVSRGHLYDNVKQGRMGFETAFGCDWYTYVSHHSFERRAFDAAMTATSTAAAQAVAAGYDFSRHGTVMDVGGGQGLLMAAILHTHSGVRAGYVMEVPGVVAAARRLGQRGMERLRYVEGDFFQPFPRPADGSPLECVVMRLVLHDWPDAEAAQILRHARKALLAPPTHHAAAAASEEEAAAADEVEAEEEEEPASGAAPTTAATAAAPTAAAAATAAEALLLLRRLLVVEAVLPELVTPPAVAADPAAPAAAVAAADGGCSIIGDGGDGGAAATADLVQRLEFDMGMMLMTQGRERSLSEWRQLLRAGGFELQQVVMTGGDGSSNGSGRSSSSGGSSGSSTAGSSGGSGGGSGGSGSGSSSGGECGSSSAARMPRLPVLVARPLLGRELGEEGE*

>Cz13g11100

METVAVPYALFVVGVAYIAITAVIFTAAFWFISPMFTINRRRSQYFDDWNVALHVPWVFWHIGAWWFRIQELLGNYVLPAPQRVMDLATQYMKSQVIIVIAELGIPDLLRKRPMNGSELAVALDLHQGYMERVLRVAERLQLLKITEIPNKSDPLGAPTRVYELTQLSAVLCEDHPNSVKYMVQLMGDHFQPAGCLGEGVRTGKTPYMLWAHGQTHWQHMTAEPELYERFNRAMTNMNNLSPIQAIFMEYSFKQFDRCIDVGGGLGSFVAGCMRMYPHMQGGVFDLPHVIEHSKKVWESQHKQLSNRITFYPGSFFEPNSIPMSQAGKQTVYMLREILHDWDDNDSIKILRSVRNAIGSNVSNCKLLIVEACIANSMKATHTPRMTGDVHMMMQYGDAKERDEREFKEVLDVSGFRLTRVIPTKGLFFILEAVPV*

>Cz04g10170

MWFRLQETLGNYVIPAPQRVLEWATRYMTSQVVIVLAELGIPDLLKNAPMTGQELASGIKAHQEYLESLLRLAVRMGLVNVTETSPSSKLDSMLDSATAPQKTYHLTDISAALCEDHPNSVKYMVQMTGDNFGAGAYLAQGIKQGKAPYTLYSGGVTYWQHMTKVPELHARFNRRSTWWCGCVAGSDADNAAAAAADTTLLLLLLILLCHHLFLFLSSSFLFILLLLQLETDMNNMSPVEAVFQAYSFKGVACCVDVGGGLGSFLAACMTQYPRMLGCVFDLPHVIEHSKQVRDTTHADLLDRVTFQSGSFFASDNVPCAAAGSTTIDLLRELLHNWSSSECVKILQTLRRSMTSAPRSKLLILEPCIADSMQSMHTPRLVHDLTMLVQFGDAKERDEREFEQLLGAAGFRLERVIPTKGLFFIVEAVPGGSR*

>Cz06g29090

MFAVTNLEVPERLVSGPKTSAQLACECGVNPEWMDRVCKAAAAMGLLGTEEATAIAGIDGQQDTKVVKPGTAEHPSIGTDSSVDAECTRDQMHKNHNITADIIAGTGQTLHSQKHPAASTAEQLRVQPSTTRLYKNTATSSVLCKGHPSSVKAFVKFFEHQFGAFGHLSQGLKQGVTPYELYSGGVTFWEHCCTDEALGTVFDEAMQAQKWLGSVAVVTDYHWGQYRAVIDIAGGIGGFLSELLQQYPQLQGVLLDLEQPVQRGKQWWSTQHPDKLNRIRFHAGDMFEPSSIPSPPSPSDNLQLDEGQTRQDTVYTLRNILHDWPDADCVKILRAIRSRITDQQVQSGAVRLAVVEMTSMEDVMPALLRFRTQFDMNMLMSFGHGKERDRAGFNALFEASGFKLLRVVPTRSVYLVLEAAPV*

>CDP20220 pep supercontig:AUK_PRJEB4211_v1:scaffold_1012:2402:3567:1 gene:GSCOC_T00005623001 transcript:CDP20220 gene_biotype:protein_coding transcript_biotype:protein_coding

MDLARNIGDHTGELFQAQAHIWSHIFNFINSMSLKCAIQLGIPDVIHKHGQPMTLDQLIDALPIKNAKATFIYRLMQNLIHSGFFIEAKIPGNENDNQKGYLLTPASELLLKSNPFSVTPFLLAMLDPTLTDPWHHLSQWFQNSDETPFYTCHGRSLYDFASHEPRINQFFNKAMASDARLVSSVVTKDCKHVFEGLNSLVDVGGGTGTLAKAIADAFPSLKCTVLDLPHVVDGLESSKNLAYVGGNMFEAIPPANAVLMKWILIDWSDD

>CDP22155 pep supercontig:AUK_PRJEB4211_v1:scaffold_11254:2:510:1 gene:GSCOC_T00006900001 transcript:CDP22155 gene_biotype:protein_coding transcript_biotype:protein_coding

AIPPADDVTMKLILHDWNDEECVQMLRKCKEAIPSKENGRKVIIIDMVLNDQQKGADDHEAIESQLFFDMLLMVLVTGKQRNEREWAKLFSEAGFNDYKTTLVLGRIH

>CDP20595 pep supercontig:AUK_PRJEB4211_v1:scaffold_1411:19161:19642:-1 gene:GSCOC_T00013329001 transcript:CDP20595 gene_biotype:protein_coding transcript_biotype:protein_coding

MKWILHDWNDEECVQMLRKCKEAIPSKENGRKVIIIDMVLNDQQKGADDHEAIESQLFFDMLMMVLVTGKQRNEREWAKLFSEAGFNDYKTTLVLGRIH

>CDP20594 pep supercontig:AUK_PRJEB4211_v1:scaffold_1411:1343:2459:-1 gene:GSCOC_T00013328001 transcript:CDP20594 gene_biotype:protein_coding transcript_biotype:protein_coding

MEKAENLVELIEAQTHVWNAMFHFKKSACLGIPDVISNHGKPITLSDLISVIIIDSVMGSQIQDETSFETEFSLDMQMLVMLGPAKERTGKEWSKLFSDAGFSSYKVYPVLGMRCLIEVYP

>CDP20700 pep supercontig:AUK_PRJEB4211_v1:scaffold_1587:23117:26022:1 gene:GSCOC_T00008849001 transcript:CDP20700 gene_biotype:protein_coding transcript_biotype:protein_coding

MDSLAETTKNHGVALKEEEEEEEHFSYAMQLVTSAAQPMVLLAAIRLDVFEIIARAGPGAQLSPSEIAANVSSENPNAAAMLDRMLRLLASYSVLTCSVATDVDGDHDIQTPTRVYGLAPVAKFFVQNKTKGGGSLGSVLGLLQDKVFIDSWYQLEDAVRKGGDPFHRAHGTHAFEFLGSDPRFNEVFNKAMIHHTAIVINRMLERYKGFEHLKTLVDVGGGLGMNLNIITTKYPSLKGINFDLPHVIQHAPAYPGVEHVGGDMFESVPQGDAIFMKWILHDWDDGHCLKLLKNCYKALPDNGKVIAVDAILPVVPDDSARDKATCQADLVVVTQYRGGIERYETEFLALATAAGFKGISVKCFVCNLWVMEFYK

>CDP18606 pep supercontig:AUK_PRJEB4211_v1:scaffold_257:223381:227546:1 gene:GSCOC_T00000316001 transcript:CDP18606 gene_biotype:protein_coding transcript_biotype:protein_coding

MECTGKITMQAILSVFKGTYWDGVETLVDVGGGTGATIAEIVKVYPHIKGINFDLPHVVATAPKYDGVSHVGGDMFDAIPSAQAVFMKWIMHDWCDEDCVKILKNCRRAIPEKTGKVFIVEVVLKPDGDGLFDSIGMILDLLMIAHSSGGKERTEPEWKKLLDKGGFPRYKITEIPACFSIIEAYPE

>CDP21212 pep supercontig:AUK_PRJEB4211_v1:scaffold_2810:4514:7277:1 gene:GSCOC_T00000226001 transcript:CDP21212 gene_biotype:protein_coding transcript_biotype:protein_coding

MDLARNIGDHTGELFQAQAHIWNHMFNFINSMSLKCAIQLGIPDVIHKHGQPMTLDQLIDALPIKNAKAPFVYLLMQILIHSGFFIEAKIPGNENDNQKGYLLTSAAELLLKSNPFSMTPLLLFTLDPTLTDPWHHLSQWFQNSDETPFYTCHGRSLYDLASHEPRLNQFFNEAMASDTRLVSSVVTKDCKHVFEGLNSLVDVGGNMFEAIPPADAVLMKWILIDWSDDECVQILKKCKEAIPSKEKRGKVIIVEMFCKSQQKGDDDHEAIETQLFFDMAVMVLVKGRQRNEKDWAKLFTEAGFSDYKITAVLGLRSIIEVYHN

>CDP21213 pep supercontig:AUK_PRJEB4211_v1:scaffold_2810:13992:14553:1 gene:GSCOC_T00000228001 transcript:CDP21213 gene_biotype:protein_coding transcript_biotype:protein_coding

MACDSQLVGSILIRDCKDVFSGLNSLVDVGGGTGTLAKEIADAFLDLNCIVTDLPHVVDGLVANNKSLAFVGGDMFVAIPPADDVIMKWILHDWNDEECVQMLRKCKEAIPSKENGGKLNFSVIR

>CDP21231 pep supercontig:AUK_PRJEB4211_v1:scaffold_2893:3934:4143:-1 gene:GSCOC_T00001556001 transcript:CDP21231 gene_biotype:protein_coding transcript_biotype:protein_coding

MFCKSLQKGDDDHEAIETQLLFHMLMMVLFKGRQRNEKDWAKLFTETGFSDYKITAVLGLRSIIEVYYN

>CDP21551 pep supercontig:AUK_PRJEB4211_v1:scaffold_4795:1107:3100:1 gene:GSCOC_T00011418001 transcript:CDP21551 gene_biotype:protein_coding transcript_biotype:protein_coding

MSLKCAIQLGIPDIIHMHGQPMALAQLIDALPINNAKAPFVYRLMRILIHSGFFIKAKIPDNEGQEGYALTPASKLLLANDPFSLTHTMVIKNCKDVFMGLNSLIDVGGGTGTVAKAIADAFPHLECSVLDLPHVVDGWESSKNLAYVGGDMFEAIPPADAVLLKWILHDWSDEECVQILRKCKEAIPSKEKGGKVIIIDMLLKSQQNGDDDAEAIETQLFFDILMMVHAKGRERNEKDWEKLFLEAGFNGYKITPVLGLRSIIEVYYY

>CDP21582 pep supercontig:AUK_PRJEB4211_v1:scaffold_5086:336:1778:-1 gene:GSCOC_T00010868001 transcript:CDP21582 gene_biotype:protein_coding transcript_biotype:protein_coding

MPTCYSQEGKQNRQLIKKMDLSRNGDANELLQAQAHIWNRIFNFINSMSLKCAIQLGIPDIIHKHCKPMTLDELTNALPIGNAKAPFVYRLMRILIHSGFFIEAKISQHDEEEGYMLTSSSKLLLKDEPLSLTAFLLSMLDPTLMDPWYHLSQWFQNYSDVNPFKTCHGKVAWELAGQDQKLNNFFNEGMASDSRLVGSILIRDCKDVFSGLNSLVDVGANNKNLAFVGGDMFVAIPPADAVIMKWILHDWNDEECIQILKKCKEVIPSKQNGGRAIIIDMVLNDQQKGADDDEAIETQLFFNMLMMVLVTGKQRNEKERAKLFSEVGFNDYKITSVLGLRSLIKVYY

>CDP21583 pep supercontig:AUK_PRJEB4211_v1:scaffold_5086:2236:3679:-1 gene:GSCOC_T00010869001 transcript:CDP21583 gene_biotype:protein_coding transcript_biotype:protein_coding

MDLARNLGDHGGEFFQAQAHIWNHIFNFINSMSLKCAIQLGIPDIIHKHGQPMTLDQLIDALPIKNAKAPFIYRLMRILIHSGFFNSDESPFYTCHGRPLWELAGHEPRLNQFFNEAMASDARLVSSLVIKDYKHVFEGLNSLVDVGGGTGTFAEAIADAFPRLKCTVLDLPHVVNGLESKNLAYVGGDMFEAIPPADTVLLKWILHDWSDEECVQILRKCKEAIPSKEKGGKVIIIDILLKSQQKGDDDHEAIETQLLFDMLMMVLLKGRERNEKDWAKLFFGAGFNDYKITAALGLRSIIEVYYY

>CDP21661 pep supercontig:AUK_PRJEB4211_v1:scaffold_5489:1323:2770:-1 gene:GSCOC_T00006934001 transcript:CDP21661 gene_biotype:protein_coding transcript_biotype:protein_coding

MDLARNIGDHTDELFQAQAHIWNHIFNFINSMSLKCAIQLDIPDVIHKHGRPMTLDQLIDALPIKNEKAPFVYRLMQILIHSGFFIEAKIPGNENDNQKGYLLTSASELLLKSNPFSVTPFLLAMLDPALTDPWHHLSQWFQNSYESPFYTCHGRSLYDFASHESQLNQFFNEAMASDARMVSSVVTKDCKHVFESLNSLVDVGGGTGTFAKAIADAFPRLKCTVLDLPHVVDGLESTKNLGYVGGNMFEAIPPADAVLMKWILIDWSDDECVQILKKCKEAIPSKEKGGKVIIVDTFCKSLQKGDDDHEAIETQLFYDMGAMVLVKGRQRNEKDWAKLFSEAGFCDYKITAVLGLRSIIEVYY

>CDP19711 pep supercontig:AUK_PRJEB4211_v1:scaffold_627:37926:39322:-1 gene:GSCOC_T00003634001 transcript:CDP19711 gene_biotype:protein_coding transcript_biotype:protein_coding

MEKVENLGELIEAQTHVWNAMFHFKKSACLKCAVELGIPDVIRNHGKPITLSDLISVLPIHPSKSAHIFRLMRFLANSGFFVENPQGYALTSAGRLLLKDEPFNVRAHIFLSCDPAMLKPWNFLTKWFQNDDPSPFDTAYGNNFWHYNAQEVRFGKMFNEAMASDNQLSVEVLMTKCKFVFEGLTTLADVGGGTGKVDRAIAQNFPNIKCTVYDLPHVVANQEGAENLEFLAGDMFQSVPRANAILLKRILHDWSDEDCLKILKNCKKAIPEKDNGGKVIIIDGVMGSQIQDKTSFETEFSMDMQMLVMLGPAKERTEKEWSKLFSDAGFSSYKVYPVLGMRCLIEVYP

>CDP19709 pep supercontig:AUK_PRJEB4211_v1:scaffold_627:22812:24128:1 gene:GSCOC_T00003631001 transcript:CDP19709 gene_biotype:protein_coding transcript_biotype:protein_coding

MERVENLTELLAAQNHVGNQMPNFRKSASLKCAIELGIPDAINQHGKPITLSELVSALPINPSKANHIYRLMRFLSNAGFFVLQDQGYALTAAGRLLLKEEPFNLRAFIFYTSDPVLVKPWNSLTEWFRNDDPSPFHTAHGKNFWAYAAEEPNFANLFNEAMANDSTLTVQVMMTQCKFVFDGLTSLVDVGGGTGAVARAIAQNFPNLECVVCDLPHVIAGQEGTENLDFVAGDMLEKVPAADAILLKWILHDWSDDDCVKILKNCKEAIPGRDKGGKVIIIDMILESHMKDDESVETQFVVDMQMLTCYGAKERTEKEWAKVFQDAGFSDYKVLPVLGVRCLIEVYP

>CDP19708 pep supercontig:AUK_PRJEB4211_v1:scaffold_627:20269:21897:-1 gene:GSCOC_T00003630001 transcript:CDP19708 gene_biotype:protein_coding transcript_biotype:protein_coding

MFNEAMASDNQLSVEVLMTKCMSVFEGLTTLADVGGGTGKVARAIAQNFPNIKCTVYDLPHVVANQEGAENLEFLAGDMFQSVPRANAILLKVTKAILKSSITIFPANAWILHDWNDEECVQILRKCKEAIPSKENGGKVIIIDMVLSDQQKGADDHEAIETQLFSDMLMMVLLRGKQRNEREWAKLFSEAGFNDYKITPVLGLRSLVEVYY

>CDP19714 pep supercontig:AUK_PRJEB4211_v1:scaffold_627:60045:62926:-1 gene:GSCOC_T00003638001 transcript:CDP19714 gene_biotype:protein_coding transcript_biotype:protein_coding

MEKVENLGELIEAQTHLWNAMFHFKKSACLKCAVELGIPDVIRNHGKPITLSDLISGIAQNFPNIKCTVYDLPHVVANQGAENLEFLAGDMFQSVPRANAVLLKRILHDWSDEDRDHYRQCNGEDYAFGSCAFRFHPETGNAFQHRPASCLWTANSLFQIKRQLPRKIIVHLSKTKMSKLADQN

>CDP19712 pep supercontig:AUK_PRJEB4211_v1:scaffold_627:49877:50652:-1 gene:GSCOC_T00003636001 transcript:CDP19712 gene_biotype:protein_coding transcript_biotype:protein_coding

MEKADNLGDLLEAQAHVWNAMFHFKKSACVKCAVELGIPDLISNHGKPITLSDLISVLPIHPSKSAHIFRLMRFLANSGFFVENPQGYALTSAGRLLLKDEPFNVRAHIFLSCDPALLKPWNFLTDSIWKMFNENMASDNHLFVEVLMTKCKSVFEGLTTLADVGGGTGKVARAIAQNFPNIKCTVYDLPHVVANQGGAENLEFVAGDMFQSVPRANAILLKVTKAILSLNF

>CDP19713 pep supercontig:AUK_PRJEB4211_v1:scaffold_627:53255:55089:1 gene:GSCOC_T00003637001 transcript:CDP19713 gene_biotype:protein_coding transcript_biotype:protein_coding

MERAENLTELLAAQNHVRNQMLNFRKSASLKCAIELGIPEAINQHGKPITLSELVSALPVNPSKASHIYRLMRFLSKAGFFVLQDQCYALTAAGRLLLKDDPFNLRALIFFMSDPVLVKPWNSLTEWFRNDDPAPFDTAHGKNFWAYAAEEPNFANRFNEAMANDSTFIVQVMMTRCKFVFDGLTSLVDVGGGTGTVARAIAQNFPNLECVVCDLPHVIACQEGTENLDFVAGDMLEKVPTADAILLKKPDSIHDWSDEDCVKILKNCKEAIPGRNKGGRVIIIDMILESQIKDDESVETQVGMDMQMLMCHAAKERTEKEWAKLFRDAGFSYYKILPVFGVRCLIEVYH

>CDP19754 pep supercontig:AUK_PRJEB4211_v1:scaffold_661:39199:42033:1 gene:GSCOC_T00012515001 transcript:CDP19754 gene_biotype:protein_coding transcript_biotype:protein_coding

MLQLLASYSVLTCSVAEADASISSQRRVYGLAPVAKYFVQNKTYAAGGGGVSLGPLLALLQDKVFIDSWYQLEDAVREGGVPFDRVHGVHAFEYPARDPRFNEVFNKAMINPTTIAINRIVQRYKGFEHLKTLVDVGGGLGVTLGVITAKYPSLKGINFDLPHVIQHALVYPGVEHVGGDMFESVPQGDAIFLRSILHDWDDGRCLKLLKNCFKALPKDGKVIVVDAIVPVVPDTSACIKAICQSDLFMMAQNPGGKERSEAEFLDLATAAGFRGIRVECFVCNAWVMEFYK

>CDP19753 pep supercontig:AUK_PRJEB4211_v1:scaffold_661:28856:31077:1 gene:GSCOC_T00012514001 transcript:CDP19753 gene_biotype:protein_coding transcript_biotype:protein_coding

MDSLSKATNNVVVEAGLDEQEEQHFSYAMQLVTSVSLPMVLLAVIRLDVLEVIAEAGPGAQLSPWDIAAQVCPKNPDAAAMLDRMLRLLASFSVLTCSVAEADARISSQRRVYGLTPVAKYFVQNKTYGAGGGVSLGPLLALVQDKVFIDSWYQLEDAVREGGVPFDRVHGMHAFEYPARDPRFNEVFNKAMVNRTTIAINRMVERYKGFEHLKTLVDVGGGLGVTLSVITAKYPSLKGINFDLPHVIQHAPVYPGVEHVGGDMFDSVPQGDAIFLRLILHDWDDGRCLKLLMNCFKALPKDGKVIVVDAIVPVVPGTSACIKAICQSDLIMMAQNPGGKERSEAEFLDLATAAGFRGIRVEFFVCNAWVMEFYK

>CDP19755 pep supercontig:AUK_PRJEB4211_v1:scaffold_661:43935:46614:1 gene:GSCOC_T00012516001 transcript:CDP19755 gene_biotype:protein_coding transcript_biotype:protein_coding

MDSSSRATDNVVVEAGLDEQEEQHFSYAMQLVTSVSLPMVLLAAIRLDVLEVIAQAGPGAQLSPWDIAAQVGPKNPDAAAMLDRMLQLLASYSVLTCSVAEADASISSQRRVYGLAPVAKYFVQNKTYAAGGGGVSLGPLLALFQDKVFIDSWYQLEDAVREGGVPFDRAYGVRAFEYPGRDPRFNEVFNKAMINHATIAINRIVERYKGFEHLKTLVDVGGGLGVTLSVITTKYPSLKGINFDLPHVIQHAPVYPGVEHVGGDMFESVPQGDAIFMKWILHFWDDGRCLKLLKNCFKALPDHGKVIVVDPILPVVPDTSAGIKATCQSDLITMTQNPGGKERSEAEFLDLATAAGFRGIMVVCFVCNVWVMEFYK

>CDP21787 pep supercontig:AUK_PRJEB4211_v1:scaffold_6650:661:2111:1 gene:GSCOC_T00004245001 transcript:CDP21787 gene_biotype:protein_coding transcript_biotype:protein_coding

MDLARNLGDHTGELFQAQAHIWNHIFNFINSMSVKCATQLGIPDVIHKHGQPMTLDQLIDALPIKNAKAPFVYRLMQILIHSGFFIEAKIPGNENDNQKGYLLTSASELLLKSNPFSMTPFLLAMLDPTLTDPWQNLSQWFQNSDETPFYTCHGRSIYAFASHEPWLNQFFNEAMASDTRLVSSVVTKDCKHVFESLNSLVDVGGGTGTFAKAIADAFPRLKCTVLDLPHVVDGLESSKNLAYVGGNMFEAIPPADAVLMKWILIDWSDDECVQILKKCKEAFPSKEKGGKVIIVDMFCKSLQKGDDDHETIETQLFFDMEVMVLLKGRQRNEKDWAKLFTEAGFSDYKITAVLGLRSIIEVYYY

>CDP21836 pep supercontig:AUK_PRJEB4211_v1:scaffold_7029:61:1617:-1 gene:GSCOC_T00010736001 transcript:CDP21836 gene_biotype:protein_coding transcript_biotype:protein_coding

MDLARNIGDHTGELFQAQAHIWNHIFNFINSMSVKCAIQLGIPDVIHKHGQPITLDQLIDALPIKNAKAPFVYRLMQILIHSGFFIEAKIPGNENDNQKGYLLTSASELLLKSNPFSMTPFLLAMLDPTLTDPWHHLSQWFQNSDETPFYTCHGRSIYAFASHEPWLNQFFNEAMASDTRLVSSVVTKDCKHVFESLNSLVDVGGGTGTFAKAIADAFPRLKCTVLDLPHVVDGLESSKNLGYVGGNMFEAIPPADAVLMKWILIDWSDDECVQILKKCKEAIPSKEKGGKVIIVDMFCKSLQKGDDDHEAIETQLFFDMVVMVLLKRRQRNEKDWAKLFTEAGFSDYKITAVLGLRSIIEVYYY

>CDP19840 pep supercontig:AUK_PRJEB4211_v1:scaffold_721:54453:56457:1 gene:GSCOC_T00005422001 transcript:CDP19840 gene_biotype:protein_coding transcript_biotype:protein_coding

MDLARNIGDHTGELFQAQAHIWNHIFNFINSMSVKCAIQLGIPDVIHKHGQPMTLDQLIDALPIKNAKAPFVYRLMQILIHSGFFIEAKIPGNENDNQKGYLLTSASELLLKSNPFSVTPFLLAMLDPALTDPWHHLSQWFQNSDETPFYTCHGRSIYAFASHEPWLNQFFNEAMASDARMVSSVVTKDCKHVFEGLNSLVDIGGGTGTFAKAIADAFPRLKCTVLDLPHVVDGLESSKNLAYVGGNMFEAIPPADAVLMKWILIDWSDDECVQILKKCKEAIPSKEKGGKVIIVDTFCKSQQKGDDDHEAIETQLFYDMGAMVLVKGRQRNETDWAKLFSEAGFSDYKITAVLGLRSIIEVY

>CDP19839 pep supercontig:AUK_PRJEB4211_v1:scaffold_721:16718:18545:1 gene:GSCOC_T00005419001 transcript:CDP19839 gene_biotype:protein_coding transcript_biotype:protein_coding

MSVKCAIQLGIPDVIHKHGQPMTLDQLIDALPIKNAKAPFVYRLMQILIHSGFFIEAKIPGNENDNQKGYLLTSASELLLKSNPFSMTPFLLAILDPTLTDPWHHLSQWFQNSDETPFYTCHGRSIYAFASHEPWLNQFFNEAMASDTRLVSRVVTKDCKHVFEGLNSLVDVGGGTGTFAKAIADAFPRLKCTVLDLPHVVDGLESSKNLAYVGGNMFEAIPPADAVLMKWILIDWSDDECVQILKKCKEAIPSKEKGGKVIIVEMFCKSQQKGDDDHEAIETQLCYDMEVMALGKGRQINEKDWAKLFSEAGFCDYKITAVLGLRSIIEVYYY

>CDP21928 pep supercontig:AUK_PRJEB4211_v1:scaffold_7886:1776:2943:1 gene:GSCOC_T00007655001 transcript:CDP21928 gene_biotype:protein_coding transcript_biotype:protein_coding

MDLGRNGHANELLQAQAHIWNHIFNFINSMSLKCAIQLGIPDIIHKQGKPMTLDELQCSSNWQCKSPTLSLNNFFNEGMASDSRLVGSILIRDWKDVFSRLNSLVDVGGGTGTLAKEIADAFLDLHCIVPDLPHVVDGLVANNKSLAFVGGNMFVAIPPADDVIMKWILHDWYDEECVQILRKCKEAIPSNGNGGKLNFSLIR

>CDP22007 pep supercontig:AUK_PRJEB4211_v1:scaffold_8978:1360:2229:-1 gene:GSCOC_T00004618001 transcript:CDP22007 gene_biotype:protein_coding transcript_biotype:protein_coding

MDTNDEGSKLHHGQAKIWKHLFGFVDSMALKSALELQIADIIHFHGRPLSLSEISSNITNSSSPNIPYLARIMRLLVRNKIFTSSEVRPGHGGDTPSTILYDLTPASNWLLNNNDPLSLAPFILMENHPWLLSPWHQLSACVREGGIAFQKSHGKEIWDFASQNPEFNKIFNDGMECTGMITVQAVLSGLKSANWDGVESLVDVGGGIGATIAEIVKAYPHIKGINFDLPHVVATAPKYDGVSHVGGDMFDAIPSAQAIFMKVLIFAKITAKSCCFLISPLYFLLWGDT

>CDP15753 pep chromosome:AUK_PRJEB4211_v1:1:15543419:15548682:1 gene:GSCOC_T00015810001 transcript:CDP15753 gene_biotype:protein_coding transcript_biotype:protein_coding

MESSVKTQLKSSSSSGENGKEGEKQNHFSYAMQLVSSASLTMVLYNAVKLNLFEIIAKAGPGAKLSPSEIASQLPVTNNPDAASMLDRMLRLLSSYSLFTCDVVEVAVDGGGGGETNVGYERVYGLSPVAEYFVPDEEGNSVAPLVELLQDKVLIDSWYELGNAVLEGGIPFNRVHGVHAFDFPSRDPKYNELFNKGMVGPTAIMMKELLQQYKGFEHLQTLVDVGGGLGITLHKIISKYPSIRGINFDLPHVIENAPSYPGVEHIDGDMFESVPGGDAIFMKMILHDWSDDHCLKLLKNCFKALPGHGKVIVVDLILPVKPDTSAFVKGIFQADALMMTQNPGGKERSESDVRALAIRAGFKDVKLQCLVGNVGVLELYK

>CDP15755 pep chromosome:AUK_PRJEB4211_v1:1:15650414:15653379:1 gene:GSCOC_T00015815001 transcript:CDP15755 gene_biotype:protein_coding transcript_biotype:protein_coding

MTKTLLDFHPMYELGNSLLEGGIPFNRVHGMHAFDYPSRDPRYNELFNKGMVGPTAITMKKLLQQYKGFEHLQTLVDVGGGLGITLHKIISKYPSIRGINFDLPHVIENAPSYLGVEHIGGDMFESVPGGDAIFMKMILHDWSDDHCLKLLKNCFKALPDHGKVIVVDLVLPVKPDTSAFVKGIFQTDALMMTQNPGGKERSESDVRALAIRAGFKDIKLECCVGSLGVLELYK

>CDO96828 pep chromosome:AUK_PRJEB4211_v1:2:13590430:13591236:1 gene:GSCOC_T00013976001 transcript:CDO96828 gene_biotype:protein_coding transcript_biotype:protein_coding

MEKVENLVELREAQDYAGTQIFNFRKCASLKCAIELGIPDVIAQHGKPIMLSGLISTLPINPSKSIHIHRLMRFLSNAGFFVQQNEGYSLSTAGRLLLKNEPFNMRAFIYYVSDPIALKPWNFLTEWFKNDDPSPFDTAHGKNFWSYAAAEPQFGKIFNEAMAGDSSLIVEVVVTQCKSVFEDLTSLVDVGGGTGEFAKAIVQNFPNLECLVCDLPHVVSNQQRTENLDFVAGNMLEMVPPGNAILLKVIRDIHPPCFNHLNFLRNKV

>CDP04982 pep chromosome:AUK_PRJEB4211_v1:2:569234:572303:1 gene:GSCOC_T00019870001 transcript:CDP04982 gene_biotype:protein_coding transcript_biotype:protein_coding description:Flavone 3'-O-methyltransferase 1 [Source:Projected from Arabidopsis thaliana (AT5G54160) UniProtKB/Swiss-Prot;Acc:Q9FK25]

MAEEEACLFAMSLASASVLPMVLKSAIELDLLELIAKAGPGAYVSPSELAAQLPTHNPEAPIMLDRILRLLATYSVLDCKLNNLADGGVERLYGLAPVCKFLTKNADGVSMAPLLLMNQDKVLMESWYHLKDAVLDGGIPFNKAYGMTAFEYHGTDPRFNKVFNQGMSNHSTITMKKILEVYRGFEGLKTVVDVGGGTGATLNMIISKYPTIKGINFELPHVVEDAPSHSGVEHVGGDMFVSVPKGDAIFMKWICHDWSDDHCRKLLKNCYQALPDNGKVILAECVLPEAPDTSLATQNVVHVDVVMLAHNPGGKERTEKEFEALAKGAGFKEFRKVCSAVNTWIMELCK

>CDO96819 pep chromosome:AUK_PRJEB4211_v1:2:13393220:13394026:-1 gene:GSCOC_T00013964001 transcript:CDO96819 gene_biotype:protein_coding transcript_biotype:protein_coding

MEKVENLVELREAQDYAGSQIFNFRKCASLKCAIELGIPDVIAQHGKPITLSGLISALPINPSKSIHIHRLMRFLSNAGFFVRQNEGYSLSTTGRLLLKNEPFNMRAFLYYVSDPIALKPWNFLTEWFKNDDPSPFDTAHGKNFWSYAAAEPQFGKIFNEAMAGDSSLIVEVVMTQCKSVFEDLTSLVDVGGGTGEFVKAIAQNFPNLECLVCDLPHVVSNQQRTENLDFVAGNMLEMVPPDNAILLKVIRDIHPPCFNHLNFLRNKV

>CDP18090 pep chromosome:AUK_PRJEB4211_v1:4:14231758:14239353:1 gene:GSCOC_T00008263001 transcript:CDP18090 gene_biotype:protein_coding transcript_biotype:protein_coding

MEKIKSILNFDHCYVVDDMNRYGGMALLWNEKTKVKDIKYSAFTIEVLIEDAEVKQEWWLVGIYASCDNQVRKNQWEVISRRKSLWGDNQIIMGDFNDVCSNEEKWGGRMREEWSFHDFRRFIQENQLIDVGFEGNPWTWSNQWQTGEIKQRLDRGLSSGGWHNLFEHTRCTHIESLGSDHSMLILDTMPGARTKRKKFFFDKRWIQREGIKEVVKKTWEEDVRGSRMFRVVNKIKRCRVALLKWRNGFIENSKKKRISDLKQRLMVEKRSGNEEMERKATILLLESSPVMLSPWLGLGRRVLANSPPPFDTYHGHDIWRYAQNNPAHSKLINDAMACDARVAVSAMIYRCPQVFEGISSLVDVGRGDGTALRTLLKACPWIHGINFDLPHVVSIAPRSDGVEHVGGDMFHSFPNADTAFIMSVLHDWGDDNCISILMNCKEAIPQDTGKVIIVEAVIDHEEGDDKLKDVGLTLDMVMMAHTTTGKERTSEEWAHILNQVGFSRHTMTHIQAVQSVIEAYL

>CDP18321 pep chromosome:AUK_PRJEB4211_v1:4:21609248:21611196:1 gene:GSCOC_T00004357001 transcript:CDP18321 gene_biotype:protein_coding transcript_biotype:protein_coding

MAGDSSLIDLTCLVMTQCMSVFEDLTSLVDVEGGTSEFTKAIAQNFPNLECLVCDLPHVVANQHRTENLDFVAGNMLEMVPPGDAILLKLIWQELALTKMAISILHDWSDDGCVKILKNCNNAIPERSKGGKVNMDMAMLVLHGAKETTEKEWAKLFQDAGFSNYKVFPVLGLRCLVEVYPD

>CDP15884 pep chromosome:AUK_PRJEB4211_v1:5:22549886:22551244:-1 gene:GSCOC_T00016789001 transcript:CDP15884 gene_biotype:protein_coding transcript_biotype:protein_coding

MERVENLTELLAAQHHVGNQMLNLRKSASLKCAIELGIPDAINQHGEPITLSELVSALPINPSKANHIYRLMRFLSNAGFFVLQDQGYALTAAGPMANDSTLIVQVMMTQCKFVFDGLTSLADVGGGTGAVARAIAQNFPNLKCVVCDLPHVIAGQEGTENLDFVAGDMLEKVPAADAILLKWILHDWSDEDCVKILKNCKEAIPGREKGGKVIIIDMILESQMKDDESVETQVGVNMQMLMGYGAKERSEKEWAKLFQDAGFSDYKALPLLGVCCLIEVYP

>CDP11862 pep chromosome:AUK_PRJEB4211_v1:5:20200513:20201617:-1 gene:GSCOC_T00035133001 transcript:CDP11862 gene_biotype:protein_coding transcript_biotype:protein_coding

MMMLLHLKLHFWGYAAGEPEFGKIFNEAMASDSNLTMEVLMTQSRLVFEGLESLVDVGGGTGKDGRAIVQNFPNIETYQIEDNEAIETQMSFDMQMLVLYGAKERTEKEWATLFSDADFSSYKIFPVLGIWCLIEVYP

>CDP16374 pep chromosome:AUK_PRJEB4211_v1:5:3036652:3039777:1 gene:GSCOC_T00018212001 transcript:CDP16374 gene_biotype:protein_coding transcript_biotype:protein_coding

MDSLAETTKNHGVVLKEEDEEEEHFSYAMQLVTSAAQPMVLLAAIRLDVFEIIARAGPGAQLSPSEIAANVSSENPNAAAMLDRMLRLLASYSVLTCSVATDVDGDHDIQTPTRVYGLAPVAKFFVQNKTKGGGSLGSVLGLLQDKVFIDSWYELEDAVRKGGDPFHRAHGTHAFEFLGSDPRFNEVFNKAMVHHTAIVINRMLERYKGFEHLKTLVDVGGGLGMNLNIITTKYPSLKGNNFDLPHVIQHAPAYPGVEHVGGDMFESVPQGDAIFMKWILHDWDDGHCLKLLKNCYKALPDNGKVIAVDAILPVVPDDSARDKATCLADLVVVTQYRGGIERYETELLALATAAGYMIELGVIGYC

>CDP15888 pep chromosome:AUK_PRJEB4211_v1:5:22639859:22641778:-1 gene:GSCOC_T00016793001 transcript:CDP15888 gene_biotype:protein_coding transcript_biotype:protein_coding

MDLARNGDHTGELFQAQAHIWNHIFNFINSMSLKCAIQLGIPDIIHKHGQPMALAQLIDALPINNAKAPFVYRLMRILIHSGFFIKAKIPDNEGQEGYALTSASKLLLANDPFSVTPFLLAMLDPILTDPWHHFSQWFQNSEETPFHTCHGTSLWELAGRQPQLNQFFNEGMASDARLVSTMVIENCKDVFMGLNSLIDVGGGTGTVAKAIADAFPHLQCSVLDLPHVVDGWESSKNLAYVGGDMFEAIPPADAVLLKWILHDWSDEECVQILRKCKEAIPSMEKGGKVIIIDMLLKSQQNGDDDAEAIETQLFFDMLMMVHVKGRERNEKDWEKLFLEAGFNGYKITPVLGLRSIIEVYYY

>CDP15886 pep chromosome:AUK_PRJEB4211_v1:5:22558654:22560311:1 gene:GSCOC_T00016791001 transcript:CDP15886 gene_biotype:protein_coding transcript_biotype:protein_coding

MERVENLTELLAAQNHVGNQMLNFRKSASLKCAIELGIPDAINQHGKPITLSELVSALPINPSKANHIYRLMRFLSNAGFFVLQDQGYALTAAGRLLLKEEPFNLRAFIFYMSDPVLVKPWNSLTEWFRNDDPSPFHTAHGKNFWAYAAEEPNFANLFNEAMANDSTLIVQVMMTQCKFVFDGLTSLADVGGGTGAVARAIAQNFPNLKCVVCDLPHVIAGQEGTENLDFVAGDMLEKVPAADAILLKWILHDWSDEDCVKILKNCKEAIPGRDKGGKVIIIDMILESQVKDDDSVETQVGVDMQMLMCYGAKERTEKEWAKLFQDAGFSDYKILPALGVCCLIAVYP

>CDP17644 pep chromosome:AUK_PRJEB4211_v1:6:31162623:31164330:1 gene:GSCOC_T00001552001 transcript:CDP17644 gene_biotype:protein_coding transcript_biotype:protein_coding

MDFARNGDHTGELFQAQAHIWNHLFNFINSMSLKCAIQLGIPDIIHKHGQPMALAQLIDALPINNAKAHFVYRLMRILIHSGFFIKAKMPDNEGHEGVTPFLLAMLDPILTDPWHHFSQWFQDNEETPFHSCHGTSMWELAGRQPRLNQFFNEGMASDARLVCTMVIKNCKDVFMGLNSLIDVGGGTGTVAKAIADAFPHLKCSVLDLPHVVDGSESSKNLAYVGGDMFEAIPPSDAVLLKWILHDWSDEECVQILRKCKEAIPSKEKGGKVIIIDMLLKSRQNGDDDDNAEAIETQLFFDMLMMVLVKGRERNEKDWAKLFFEAGFNGYKITPVLGLRSIIEVYYY

>CDP07899 pep chromosome:AUK_PRJEB4211_v1:8:20388651:20391118:1 gene:GSCOC_T00025391001 transcript:CDP07899 gene_biotype:protein_coding transcript_biotype:protein_coding

MECTGMITVQAVLSGLKSANWDGVESLVDVGGGIGATIAEIVKAYPHIKGINFDLPHVVATAPKYDGVSHVGGDMFDAIPSAQAIFMKWIMHDWDDDDCVKILKNCRRAIPEQTGKIFIVDVVLKPDGDGLFDSVRMKLDLVMIAHASGGKERTEPEWKILLQKGGFPRYNISAIPACLSVIEAYPE

>CDP18417 pep chromosome:AUK_PRJEB4211_v1:8:22333969:22337256:1 gene:GSCOC_T00007228001 transcript:CDP18417 gene_biotype:protein_coding transcript_biotype:protein_coding

MVLNAAVKLDVLEIIAKAGPGGKLSPSEIVSQMPTKNPDAPDFLDRMLRLLAGYSVLTCSVVDGGAGAHHERRYGLAPVAKYFIKHQYGATLRQLSVYLQNKLLMDSWYQLEGSVLEGGNAFKRTHGCELYTYMAKDPTYNEAFNKAMSCHTKVVLEKALECYKGFENLKTLVDVGGALGQAIHMITSKYPDIKGINFDLPHVIELAPPYPGIEHKGGDMFESVPEADAIFMKWILHNWDDEHCVKLLRNCYKALPNDGKVIVVDAIVPVNPENSDAAAKSNMQIDLFMMAVCSPGAKGRSELEFRALATEAGFRGIRVDCRLFDLWVLEFYK

>CDP00481 pep chromosome:AUK_PRJEB4211_v1:11:28813798:28817537:1 gene:GSCOC_T00032433001 transcript:CDP00481 gene_biotype:protein_coding transcript_biotype:protein_coding

MSSTNPSSPLPLLFTSMLLESLKLHKSTISSTMPADSSSSSSEKGHPMNSAIHLMWKSGSSLSSSYVKNKTFPGFPANTAAHCSKMVQPDSLLPCRTCRKPLPVKGFPFQAFSFCLRSVKGIFKHPWTSCRCRSACTLSPPRILAMTVRHVSRYDPRRRRGIRIRYLFIKFIIVLLHRHQFVGILRYALTPAGRLLLKDEPFNVRAYVFMAADPVVLKPWFFLTEWYQNDDLSPLYTAHGNNFWHYAAREVPFGQMFNNEVMANDSEFCIEVLMTKCKFVFEGLATLADVGGGTGKFARAIAKNFPSIKCTVYDLPHAVANEGGDKNVDFVAGDMFESVPYANAILLKLILHDWSDEDCTKILKNCRKAIAEKHDGGKVIIIDIVMGSRIQDKASLQTQINTDMQMLVALGPAKERTEKEWAKLFMEAGFTSYKVYPILGTVRSLIEVYP

>maker-chr01-exonerate_est2genome-gene-33.22-mRNA-1:cds pep supercontig:Melonv4:contig2:3385694:3386853:1 gene:MELO3C018856.2 transcript:MELO3C018856.2.1 gene_biotype:protein_coding transcript_biotype:protein_coding

MLESAFVEPSHFLSAWFRTDDQTPFETAHGMSFWEFVGNKQKDGDIFNAGMASDARLVMSVLIGKHKSVFEGVESLVDVGGGTGTMTKAIAKAFQQIECTVLDLPQVVAELKSDIPNFKYVEGDMFDAIPPADALLLKWILHDWSDEECVKILKKCKEAITSNGNKGKVMVIDLVLFNKKNDQDSIETQLFHDMLMMVVTGGKEREEKEWAKLIKEAGFSAYKIFPILGLRSLIEIYP

>maker-chr04-exonerate_est2genome-gene-249.17-mRNA-1:cds pep supercontig:Melonv4:contig5:24916622:24918606:-1 gene:MELO3C026750.2 transcript:MELO3C026750.2.1 gene_biotype:protein_coding transcript_biotype:protein_coding

MEENIELVEAQAHIWNHTFKYINSMSLKCVVELGIPDIIHNHGQPMSLSQLLESLHIHPSKAQCLSRLMRLLVHSGFFAQPQPDFFSLTPPSRLLLRENHKTAFDTTPFLLLILSPLMMSPWQTMSKWLCSQDDDQDQDHYSTAFELANGKPIWDYVKEEEESCGFGKLFHQTLECDSRLIGKVVSSECGEMFEGLRSLVDVGGGAGAMAKAIVEAFPHINCFVLDLPQVVANQKPKQHIQNLHFIEGDMFQKIPPANAVLLKSILHDWNDEESIKILKKCKESIPSRGEGGKVIIIEMVLEKELEQMKKSSVETQLC

>maker-chr01-exonerate_est2genome-gene-34.55-mRNA-1:cds pep supercontig:Melonv4:contig2:3425986:3428094:-1 gene:MELO3C018859.2 transcript:MELO3C018859.2.1 gene_biotype:protein_coding transcript_biotype:protein_coding

MYEWKKSRSQVLDTSPKCLDNYFSLVVLNMNMEAGGKKLAMGGDELLEAQSHIWNHIFNFINSMSLKCAIQLGIPDAIHSHGPNPMPLSLLVSSLQLHPNKTQFIYRLMRLLTHSGFFVQQEEGYILTNSSRLLLKDNHFAVSPFLLSMLQPALTDPWQFLSIWLQTDDRTPFETAHGMPFWEYMGNKAKDGEVFNEGMASDARLVMSVILEKHKSVFEGVESLVDVGGGTGTMAKAISQAFPQMECTVFDLPQVVAHLKEDQPNFKYVEGDMFKLIPPADVLLLKWILHDWSDEECVEILKNCKAAITSNGDRGKVMVIDIVLFGNKKDSMETQLLFDMLMMTLAGGKEREEEKWAELIKEAGFRSYKIFPIMGVRSLIEIYP

>maker-chr01-exonerate_est2genome-gene-153.9-mRNA-1:cds pep supercontig:Melonv4:contig2:15329520:15330614:1 gene:MELO3C027370.2 transcript:MELO3C027370.2.1 gene_biotype:protein_coding transcript_biotype:protein_coding

MKGINFDLPNVVSTSEKYDGVEHVGGNMLDFVPKADAAFFMWILHAWDDEDCIKILRNCKEAIGENKAGGKVIIIDSVIDENEENKMVTDIRLTLDIMMMTRSRKGRERSADEWTQLLINKAGFSRCTITPIPAAVPSIIQAFIS

>maker-chr04-exonerate_est2genome-gene-249.25-mRNA-1:cds pep supercontig:Melonv4:contig5:24930131:24930448:-1 gene:MELO3C000525.2 transcript:MELO3C000525.2.1 gene_biotype:protein_coding transcript_biotype:protein_coding

MNNSSGEGRGKVIIIEAVLEKENQMEDKESIETQLCGDVLMMATFNTAKRNEKEWKTLFLAAGFSHYSITSFLGLRSLIELYP

>maker-chr01-exonerate_est2genome-gene-34.5-mRNA-1:cds pep supercontig:Melonv4:contig2:3400267:3400546:1 gene:MELO3C028075.2 transcript:MELO3C028075.2.1 gene_biotype:protein_coding transcript_biotype:protein_coding

MGSNKSSEGAAVFDASLASDAKFVVSVLMEKCKGVFDGVGSLVDVGGGTGNVTKCIAQAFPQMECTVFDLPQVVADLKAEGNLKFVGGDMFQS

>maker-chr01-exonerate_est2genome-gene-152.35-mRNA-1:cds pep supercontig:Melonv4:contig2:15280987:15283334:1 gene:MELO3C013313.2 transcript:MELO3C013313.2.1 gene_biotype:protein_coding transcript_biotype:protein_coding

MNFAPQVSISKKEEEEARVQIWKYIFGFVEMAIVKCAIELRIGDTIESHGSPMTLSQLSTALNCSSSLLYRILRFLVRRGIFKQEITEENVISYDHTPLSRLLASSNNNSMAPLLLLESSPVMLAPWHRLSARIKGNGETPFEAAHGKDVWSFAAADPIHNIVINDAMSCTARVHTVPAILEDCPQIFEGIGSLVDVGGGNGTCLSMIVKAFPWIKGINFDLPHVISSSQQYIGVEHVGGNMLDSIPKADAAFIMWVLHDWDDETCIKILKNCKEAISEKRGKVIIVEAIIEERSEEENNNNNLGDVGLMLDMVMMAHTENGKERTIKEWGNVLRQAGFTRYTITPIRAVHSVIQGFL

>maker-chr11-exonerate_est2genome-gene-114.8-mRNA-1:cds pep supercontig:Melonv4:contig12:11484721:11487090:1 gene:MELO3C019324.2 transcript:MELO3C019324.2.1 gene_biotype:protein_coding transcript_biotype:protein_coding

MGENLNELLQSQAHVWNHALKFINSMSLKCVLELEIPDIIHNHGQPMSLSSLVAALHIEPTKAECLSRLMNLLVHSGFFTTAQTQAHDRAEDVKYSLTPSSKLLLHNKQATPFLFLALDKSTIASFQSLSSWFCSSNNNGQNYSNAFEMANGKLLWEYAAQEQTFANLFQQTMVCDSEMIGKIVKECSEVFEGLKSLVDVGGGTGVMGKAIVEAFPHITCTVFDLPQVISNQPLQNAKNLRFVEGDMFEEIIPLANAVLLKWLLTNFQKLMRDQSHQSRKKWILHDWNDEQSIKILKKCKDAIPSREKGGKLIIIDIVMEDKKEEKESTETQLLFDVLMMVNLGGKERNENEWKNLFMEAGFSGYKIISKLGLRSVIEVYPA

>maker-chr04-exonerate_est2genome-gene-312.0-mRNA-1:cds pep supercontig:Melonv4:contig5:31193767:31196529:1 gene:MELO3C009403.2 transcript:MELO3C009403.2.1 gene_biotype:protein_coding transcript_biotype:protein_coding

MVVGKETAPMEQHNASTTADSEIQNKARLAILELANMISVPMSLNAIVRLNVADAIWQNGSNSPLSASEILARVVPSGGGDAHNLERILRMLTSYGVFEEHLSPNSSNHRYSLTDVGKTLVTDTDGLSYAPYVLQHHQDALMRAWPRVHEAAIDSTTEPFVRANGEPAYSYYGKKTEMNELMQRAMAGVSVPFMKAVLDGYDGFKGVEKLVDVGGSAGDCLRMILQKYPSIKEGINFDLPEVVARAPPIPGVSHVGGDMFKSIPTADAMFMKWVLSTWTDDECKIILENCCKSLPVGGKLIACEPTLPEKTDESHRTRALLASDVFIMTIYKAKSKQRTEEQFRQLGLSAGFSALRPFHIDYFYCLLEFTK

>maker-chr01-exonerate_est2genome-gene-152.41-mRNA-1:cds pep supercontig:Melonv4:contig2:15231414:15233627:-1 gene:MELO3C013310.2 transcript:MELO3C013310.2.1 gene_biotype:protein_coding transcript_biotype:protein_coding

MEVKHKESSSEKEEDGQAIIQMWRYIFRFTEMAAIKCAIDLKIADIIESYGSPVTLSQLSSTLNCSSSLLYRILRFLIHRGIFKRETIDENQIGYSQTPMSRLLAINVENSMAPLLLLETSPVMLAPWQHLSAHLKNSDTSPFEIAHGKDLWNYAEANHEHNLLFNEAMACSAKVIVSAIIEGCGDVFDGVGCLVDVGGGNGSTLNILVKVCPWMKGINFDLPHVVCASPQYENVEHVAGNMFDFVPHADVAFLKWILHDWEDEECIKILKKCKEAIPKSGGKVIIIEAIIIEAEKGEKMKKKLSDVGLMFDLVMMAHTNKGRERTAEEWAFLIHQAGFTRHTITPLQAIQSLIQCFP

>maker-chr11-exonerate_est2genome-gene-114.7-mRNA-1:cds pep supercontig:Melonv4:contig12:11475396:11476646:1 gene:MELO3C019323.2 transcript:MELO3C019323.2.1 gene_biotype:protein_coding transcript_biotype:protein_coding

MFGIMLLKFINSMSLKCVLELGIPDIIHNHGQPMSLSSLVAALHIEPTKAECLSRLMNLLVHSGFFATAQEQDHDKAEDVKYSLTPSYKLLLHHSQATPFLFVSLDKAIIASFQSLSSWFRSSNNNGQNYSNAFEMANGKLLWEYAPQEQTFASLFQQTMVCDSEMIGKIVKQECSEVFEGLKSLVDVGGGTGVMGKAIVEAFPHITCTVFDLPQVISNQPLQNAKNLRFVEGDMFEEIIPPANAILLKWILHDWNDEQNIRILKKCKDAIRSREKCGKLIIIDIVMEDKKEEKESTETQLLFDVLMMVNLGGKEMNENEWKNLFMEAGFSGYKIISKLGLRSVIEVYPA

>maker-chr11-exonerate_est2genome-gene-178.7-mRNA-1:cds pep supercontig:Melonv4:contig12:17829136:17830624:1 gene:MELO3C013600.2 transcript:MELO3C013600.2.1 gene_biotype:protein_coding transcript_biotype:protein_coding

MEAKETNEAEALLQGQAEIWKYMLCFADSMALKCAVELHLADIINSHGSPISLSQISSSIAASNPSSSPQISYLNRIMRLLVRRNIFAAHHPSDGGDTLYGLTHSSKWLLRDSPLTLAPMAFSELHQWMVSPWLCFTEAVKEGGSPFKIAHGLDIWDFASKNPQFNHFFNDAMASTSKVVMNAILSVYQDGFNSLDSLADVGGGIGGSISEIVKAFPHIKGINYDLPHVISTAPVYEGVTHIGGDMFEDIPKVDAIFMKWILHDWNDKECVKILENCKKAIPEKRGKVIIVEVVLNEEGKGAFDDTKFYFDLLMLAHTNGKERTEKEWKTILEEAGFSRYNLIPLPALVSIIEAYPS

>AFZ23489

MVASEDRPTSLPVKLASLDMLNLITSYRVTQTIHVAAKLGIADLLKDGPKRSQELADDTATDASALYRLLRALASIGVFQEVEHDLFELTALGESLRSDVPGSMRAWAIMVGGEHHWQPWGHLLHSVQTGKPAFDHVFGMGPFEYYKQKPAAGQIFQEALGGLTQIVNSQILASYDFSSIQKLVDIGGGHGSLLSGILQANPEMLGVLFDQKSVIDQAAALLEDKGVYSRCELVAGDFFASVSKGGDAYILKHIIHDWDDERSVKILKNCYEAMSGDSKLLVVEMVIPSGNTPFYGKFLDIEMLVGYSGKERTADEYQNLFAQAGFKLTQIFGTQALVSVIEGVRA

>KAB1200567.1 Trans-resveratrol di-O-methyltransferase [Morella rubra]

MDPVQAQAGASHELLEAQTHLYQHVFNYISSMSIKCVVQLGIPDIIHNHGKPITLPELASALEIHDPTKAGAIHRLMRLLVHNGFFAQTEVHGNQQEEEELYDLTPSSRILLKDNVMSLSKSVLASLHPAIMDPWHVLESWVRGDKVTPFENAHGMDFWNYAVQNPAFGTLYDEALASDSGMVNLVLRDCKSVFEGLDTLIDVGGDSSNLKFVGGDMFELIPSADAILLKLVLNNWGDEDCLKVLKKCREAISNNGNRGKVVIIDIVLNEKKDKHELTEAKLYFDLLMMIVVSGRQRYEKEWEKLFLEAGFSHYKIIPLFGLRSLVEVYP

>KAB1202878.1 Caffeic acid 3-O-methyltransferase 1 [Morella rubra]

MTAFEYQGKDLRLNNIFNKGMSDHSTFTMKKILETYKGFEGLTSVVDVGGGTGVVLNMTVSKYPSIKGINFDLPHVIADAPSYPGVVAAGVDHVGGDMFASVPRGDAIFMKWICHDWSDEHCMKLLKNCYDALPDNGKVIVVECILPVAPDTSLASKGICHVDIFMLVQTPGGRERTEEEFEAFAKGAGFQVFRVMGCAFNTSIMEFIKKSSA

>KAB1202983.1 Caffeic acid 3-O-methyltransferase [Morella rubra]

MPTTNPEAPRMLASHSVFRCSVVADGSESFQRLYSLSPVSKHFVRDEVGSHWDLLWRCFKTRSQLKDAILEGGIPFNRVHGTHPFEYPGLDPMFNQVCNKAMFNHTTMVIMKILQSYKGFEQLEQLVDVGGGGDMFSSVPEGDAIFMKWILHDWSDEHCLKLLKNCHGAIPNHGKVIVLEAVLPVMSEISTSVKSTSQLDVLMMTQTPGGKERTREEFLTLVTEPGFKGIRYGCFVRNFWVWSF

>KAB1202984.1 Caffeic acid 3-O-methyltransferase [Morella rubra]

MASQTNSSPCPSLLNDHPKEEEDSFSHAMQLANSLVLPMALQSAIELGVFDILAKAGPEAELSSSQIVAQMPNTNPEAPKMLDRILRMLASHSVFRCSVVADDSESFQRLYSLSPVSKHFVRDEDGISLGPFMALLQDKVFVDSWVYGTHAFMYPGLDPRFNQVFNRAMFNHTTMVIKKILESYKGFEQLEQLVDVGGGLGVTLNLVTSRYPHIKGINFDLPHVIQDAPPFPGVEHVGGDMFSSVPKGDAIFMKWILHDWSDEHCLKLLRNCYGAIRNDGKVIVLEAVLPVMPEISTSVKSTSQLDVLMMTQNPGGKERTREEFLTLATEAGFKGIRYECFVCNFWVMEFLK

>KAB1205223.1 Caffeic acid 3-O-methyltransferase [Morella rubra]

MGSTGETQMTPTQVSDEEANLFAMQLASASVLPMVLKSAIELELLEVISKAGPGAYLSSSEIASQLPTTNPDAPVMLDRILRLLASYSVLTYSLRTLPDGRVERLYGQGPVCKFLTKNEDGVSIAALCLMNQDKVLMESWYYLKDAVLEGGIPFNKAHGMTSFEYHGKDMRFNKVFNKGMADHSTITMKKILDTYKGFEGLASVVDVGGGTGAVLSMIVSKYPSIKGINFDLPHVIEDAPSYPGIERVAAGVEHVGGDMFVSVPKGDAIFMKWICHDWSDEHCLKFLENCYKALPDNGKVIVAECILPVAPDTSLATKGVIHIDVIMLAHNPGGKERTEKEFEALAKGAGFQGFRVMGCAFNTYIMEFIKKL

>KAB1205353.1 Caffeic acid 3-O-methyltransferase 1 [Morella rubra]

MDSMGKQTTDHMASTLNEVEEAIIHDFVVSGSFLVPMVLNACVELNVLEIIHKAGPNMQLSSHAITSHLPTSNPDAPHLLDRMLYLLCSYSLLTCSVETLEDDRVQRRYGLAPAGKFYLKNTETGSLSTFALLTSHRAIMDMRFHFKDAVLEGGYPFEKANGVSIFKHREKDPRFGEAYNSGMSEHSTFFMKQILNTYHGFEGLSSLVDVGGGNGAMLHLIMSKYPSIKGGVNLDLADVIRHAQPYKGIEHVDGNMFEDVPKGRDAILVKQVFHNWSDADCLRILRNCHKALPSNGKVIIIDLIMSTAPDTTIMGKFICHYDNLMFGLFGARERAENEFKALAIGAGFSKFRLVCCVCGFGVMELHK

>KAB1205360.1 Caffeic acid 3-O-methyltransferase 1 [Morella rubra]

MDSMGKQTTDHMASTLNEVEEAIIHDFVVSGSFLVPMVLNACVELNVLEIIHKAGPNMQLSSHAITSHLPTSNPDAPHLLDRMLYLLCSYSLLTCSVETLEDDRVQRRYGLAPAGKFYLKNTETGSLSTFALLTSHRAIMDMRFHFKDAVLEGGYPFEKANGVSIFKHREKDPRFGEAYNSGMSEHSTFFMKQILNTYHGFEGLSSLVDVGGGNGAMLHLIMSKYPSIKGGVNLDLADVIRHAQPYKGIEHVDGNMFEDVPKGRDAILVKQVFHNWSDADCLRILRNCHKALPSNGKVIIIDLIMSTAPDTTIMGKFICHYDNLMFGLFGARERAENEFKALAIGAGFSKFRLVCCVCGFGVMELHK

>KAB1206012.1 (R,S)-reticuline 7-O-methyltransferase [Morella rubra]

MALKCAVELRLADIIHSHNGPIILHQIASGIDSPSPDILYLSRIMRSLVRKKIFSEHCPTDGGETIYELTHVSRWLLHDVELSLAPMVLMQNHPWVVAPWYYLSQCVKEGGIAFKKAHACDVWEFASQNPEFNKLVNDSMACTTKIVMMAILAEYKAGFDCIGSLVDVGGGTGGMISEIVRSHPQIRGIDFDLPHVIATAPLHEGVSHVGGNMFEAIPCADAIFMKFVLHDWDHEDCIKILRNCRKAILEKTGKLIIVDIVLEKDSHDLFDDTRMVFDLMMMAHSSGGKELTELEWKEFLKGGFPGYKITKIPAIPFIIEAYPM

>KAB1206013.1 (R,S)-reticuline 7-O-methyltransferase [Morella rubra]

MGVVMAKYKDGFDGVGSLVDVGGGTGGMIAEIVKSHPHIRGINFDLPHVVATAPWVLHDWGDEDCIKILWNCRKAIPEKRKKGRLIIVDIVLEKDSHDLFDETRMVFDLSMMAHTASGKEKTELEWKEILKEGGFPRYKINKIPTIPSIIEAYPI

>KAB1206016.1 (R,S)-reticuline 7-O-methyltransferase [Morella rubra]

MVLAEYKDGFDCVGSLVDVGSGGTGGMIAEIVKSHPHIRGINFDLPHVVATAPVRERVSHVGGDMFEAGAIPDADAIFMKGKLIIVDIVLEKDSHDLFDKTRMVFDLLMMAHTASGKERTELEWKELLTEGGFRCYKIIKIPTIPSIIEAYPIQSLVQRFSN

>KAB1207631.1 (R,S)-reticuline 7-O-methyltransferase [Morella rubra]

MGETQIREAKWQSEEEQAEVGIWRCIFGFTEMAVVKCAIDLGIADAIESHASPMTLSELSTALGCAPSSLYRIMRFLMHRGIFKEKINSQGSLVYTQTCLSRRLTRHGEQSMAPLILLESSPVMLAPWHCLSARVRATETPPFDVAHGGDIWRYSATNPGHSQLINEAMACDARLVVPSMIRGCPEVFDGLSTLVDVGGGTGTTLQVLVKAFPWLRGINFDLPHVVSVAAEFTGVEHVGGDMFEGVPEADAVFLKWVLHDWGDDECIQILKKCREAVPDDKGKVIIVEAVIEEADQGDKLTEVRLALDMVMMAHTTTGKERTSKEWGFVLGKAGFSRYTVKPLIRTVQSVIEGFP

>KAB1207632.1 (R,S)-reticuline 7-O-methyltransferase [Morella rubra]

MEKTPRRAPWHEEEEQGEVGIWKYAFGFSEMAVLKCAIELGIAEAIESHGSCPMTLSELSSALGCAPSALFRIMRFLVHRGMFKEELTAQGSLGYAETPLTRRLMKHGEYSMAALILLESSPVMLAPWHSLSASCVLANESSPFDVAHGEDLWRFAAANPGHSQLIDEAMACIARSTVRLMVQGCPEVFDGLSSLVDVGGGNGTTLQKLVKACPGIRGINFDLPHVVSVAEEFTGVEHVGGNLFKSVPKADAALLMSVLHDWGDDDCIDILKKCREAIPVDKGKVIIVEAVIEEGDQTDKLTDLRLGLDMIMMAHTTAGKERTLKEWGFVLGKAGFSRYTVKPLPAMPSVIEAFP

>KAB1209855.1 Trans-resveratrol di-O-methyltransferase [Morella rubra]

MSGFRQLSFKLVPGRQGTELLHAQAHTWNHILSFAKSMSLKCAVQLGIPDIIHNHGKPMTFSELIAALPIHPSKAGNVYRLMRFLVHSGVFAEEKSTEIDQEEGYLLTGVSRLLLKDDPLSLTPLLLFVLDPDMTKPWHHLTSWLQNDDPMPFDTAHGMTMWDYEGCDPKMAHLFQDAMASDTRLVISVVIDKCSDVLEGLESLVDVGGSSGTMTKAITDAFPNIECTVLDLPHVVAGLQDNANLKYVGGDMFEAVPPADAVLLKWILHDWNDEECVKILQKCKEAITRNGKKGKVIIIEMMVGNQKGDKDSTETQLFFDMLMMALVKGKERNEKEWAKLFYDAGFTDYKIKPILGLRSLIEVYP

>KAB1209856.1 Trans-resveratrol di-O-methyltransferase [Morella rubra]

MKHEGRRSGAERLLLKDDPLSLTPLLLFVLDPDMTKPWHHLTGWLQSDDPMPFDTAHGMTVWDYEGRDPKIAHLFQDAMASDTRLVISVVIDKCNEVFEGLGSLVDVGGSSGAMAQAINDAFPNIDCTVLDLPHVVAGLQGRKKLKYVGGDMFEAVPPADAVLLKWILHHWNDEECVKIQKCKEAITRNGKKGKVIIIEIMTVENQKGDKDSTETQLFFDKLMMALAKGKERNEKEWAKLFYDAGFSYYKINPILGLRSLIEVYP

>KAB1209896.1 (R,S)-reticuline 7-O-methyltransferase [Morella rubra]

MALTEAKVLQDGQTKLWKHTFSFAVSMALKCAVDLRVADIINSHGVPISLSQIAAKIDSPSPNIPNLERIMRLLVRKEIFSAHQRPNSEETLYGLTASSRWFLHDSELSLAPFISLQNSEWLMAPWYRLAQFVKESVPGGSTGFSKCYGSEIFDFASKNHEFNKLFNAGMESTATVMVDAILTGYKDGFSSIGSLVDVGGGNGHMISKIVKAHPHIKGINFDLPHVIAAAPKHKGVANVGGNIFEAIPKADAIFMQRVLASFNDEDSIKVLKNCLKAIPKETGKVVIIDPLLDLENEGPFDEMIAALDLSMMMLCKGGQERTKLEWQKILEASGFLLNRVIKIPAVLTIVEAYPA

>KAB1210045.1 (R,S)-reticuline 7-O-methyltransferase [Morella rubra]

MESTEACKALMQKGQAQLWQHIFAFAESMALKCVVELRVADIIQSHGVATSLSQIAAGIDSPSPNIPYLSRIMRLLGRKGIFSAHQSSTSGETLYGLTDVSRCLLSESELDQTPLVLMQNHEWVMATWHRLAECVKEGGNAFVKVHGCELFDLAAKNPEFNKLFNDAMTCSAKGFARSLVVGYKDGLDSINGSLMDVGGGSGELIANIIKEYPHIKGVNCDLPHVIATAPAHKGVSHVEGDMFGALPKADAIIMQRILHGFDDEDCIKILRNCLKAIPKKGGKLIVVDHVRKADGNGAYDAMGVTIDLLMMTLGTGQGGKQRTAPEWKKLIEKGGFSVQRIIELHNLPSVIEAFPA

>KAB1215422.1 Isoflavone-7-O-methyltransferase 9 [Morella rubra]

MASDSGMMNLVVRDCKPVFKGLDTLIDVGGGTETCARIISKAFPHLKCRVFDLPLVVKLFFSSSLNLDYVAGEMFQSIPPADAILLKQC

>KAB1215426.1 Trans-resveratrol di-O-methyltransferase [Morella rubra]

MRLLVNSGFFTRSIKGQENQEAEEEEEEEAYALTPSSKLVLKENVTTLSPFVLAMLDPALVNSWQFLGDWFRAGSELTPFGEAHGMGFWDYCDQNAEYGNMFNEGMASDSRLMSLVLMDYAPIFEGLGSLVNVGGGTGTMARIISEAFPHMSCTVFDLPHVVANLPDSSNLIYVGGDMFQSIPSADAILIKASNRFSLLKDLQYFILKWILHDWNDDECVSILKRCKEAITSNGKKGKVIVIDVVIDEEKDEQGIIKTKLLFDALMMVLLTGKERNKKEWEKLFLDAGFSRYKIAASFGMKSVIEIYP

>KAB1215429.1 Isoflavone-7-O-methyltransferase 6 [Morella rubra]

MLDPALVNSWQFLGDWFRAGSELTPFGEAHGMGFWDYCDQNAEYGNMFNEGMASDSRLMSLVLMDYAPIFEGLGSLVNVGGGTGTMARIISEAFPHMSCTVFDLPHVVANLPDSSNLIYVGGDMFQSIPSADAILIKASNRFSLLKDLQYFILKWILHDWNDDECVSILKRCKEAITSNGKKGKVIVIDVVIDEEKDEQGIIKTKLLFDALMMVLLTGKERNKKEWEKLFLDAGFSRYKIAASFGMKSVIEIYP

>KAB1219935.1 Caffeic acid 3-O-methyltransferase [Morella rubra]

MAKNRRGKGRKRQKRMASFDTDTQIPPLQTGEDLAFSKAMELVYGSALLMAMHAAIDLGVFDILASAGPEAKLSAADIAAKMPTENPEAPAMLDRICTLLVHDCVLDYAISIPKCAFADTNWLYCLNPVSEYFVRNQDGVSLAPILTLIQDNVDMDTWSRLKDAVLEGGVPFHRVQGMDAYYKYLGEDARLSQIFNTAMSNHTTIIVKTFLENYDGFEKVKQVVDVGGGIGAALSLITSEYPQIKGINFDLPHVIDCAPRYPRVKHVAGDMFESVPKGDVIILKWILRDWGDEQCLKLLKNCYDALPKDGRVIVMDQFLPIKPGAAGKSSFLLDTLLMTQNLGGKQRKPDEFSRLAREAGFTYSLSDQHFVLDIWVIEFRKISNHEYRSH

>KAB1220994.1 Caffeic acid 3-O-methyltransferase 1 [Morella rubra]

MNDRSTSRVLLEGVDGGDKLDSGSFDGLGGEEDGEDPSTETGEGDLRMREPKKKWRSSTEIEGKNGNKKKRKSVESPTTKRTTEKERRDHLKQLCVESQRILRGLAQDGCRMDASAVLYTSVKSAHASVAMLHQKKIRGGDGLGTSAGWGGLILQLAIDMVQIDVDNMSPDVVKFLRFNRAPLGKNEQLQTHLPEPDFSTARARRRRRGGQIGRPLANAVALPMVLKSALELNLIDIIAESGTGALLPVSEIAARLRTKNPDTSVMLDRMLGLLASYDILKCSLRTRKDGEVERLFGVGPICKFLVRNSDGASVGPLFLLHHDKVFMESWFHMNEAILEGGIPFKRAYGISIFEKLGTDERFNRVFNQAMSHHSTLIMKKILDVYKGFEGLKVLVDVGGGIGATLNSIISKYPQIKGINFDLPHVLVDAPPFPGVEHVGGNMFDSVPSGDAIFMKWMLHGCSDEQCLQALKNCWKVLPNSGKVIVVESIRPVAPENNVSSQIVYEQDLMMLTQSPGGKERTQNEYEALALKSGFSGSEVICCAYNTWVMEFRK

>KAB1221202.1 Trans-resveratrol di-O-methyltransferase [Morella rubra]

MDPVQAQAGASNELLEAQSHLYQHVFNYISSMSIKCVVQLGIPDIIHNHGKPITLPELASALEIHDPTKAGAIHRLMRLLVHNGFFAQTEVHGNQQQEEELYDLTPSSRILLKDNVMSLSKSVLASLHPAIMDPWHVLESWVRGDKVTPFENAHGMDFWNYAVQNPAFGTLYDEALASDSGMVNLVLRDCKSVFEGLDTLIDVGGGTGTCAKIISEAFPNLKCTVLDLPRVVSDLRDSSNLKFVGGDMFELIPSADAILLKSVLNNWGDEDCLKVLKKCREAISNNGNWGKVVIIDIVLNDKKDKHELTEAKLYFDLLMMIVVSGRQRYEKEWEKLFLEAGFSHFKIIPLFGLRSLVEVYP

>KAB1222015.1 Trans-resveratrol di-O-methyltransferase [Morella rubra]

MANQLLFHSWSLSFTFLPQKTSSLHRLMRLLVHFGFFTKTKVRENQEEEEAYGLAPSSRLVLRDNVASFSWLVLLISEPFLVNPCHFWADWFRGSDEFTPFENAHGMGFWDYCDQHPDLGNMFNKAMASDSQLMSLVVKDYKPIFEGLGSLVDVGGGTGTAARIISEAFPQIKCTVFDLPRVVANLPDSTNLKYVGGDMLQSIPPVDDILMKWVLHDWCDEECIKILQRCKEAITSEERNKTEWEKLFLEAGFSHYKMVASFGVRSVIEVYP

>KAB1222016.1 Trans-resveratrol di-O-methyltransferase [Morella rubra]

MRLLVNSGFFTRSIKGQENQQEEEEEAYALTPSSKLVLKENVTNSSPFILAMLDPALVNSWQYLGDWFRGSELTPFGEAHGMGFWDYCDRNAEYGNMFNEGMASDSRLMSLVVKDYKSIFEGLGSLVDVGGGTGIMARIISEAFPHMNCTVFDLPHVVANLPDSSNLIYVGGDMFQSIPSADAILIKVSNRFSLLKDLQYFISKVHGILFSYISNYILQWILHDWNDDECVSILKRCKEAITSNGKKGKVIVIDLVIDEEKDEQDIIKTKLLFDALMMVLVTGKERNKKNGKSSSWMLALAASKLQHHLA

>KAB1222017.1 Trans-resveratrol di-O-methyltransferase [Morella rubra]

MDLTRDQGASELFHAQSHLYRHIFSFISSMFLKCAVQLGISDIIHNHGQPITLPELVLELQINPTKAGFLHRLMRLLVHCGLFATTRVHKNQEDEEEAYDLTPSSRILLKDNITSLSPFVVAMLDPALVTPWQVLGNWFRCDQVTPFEGAHGVGFWDFGDQNPEFNKLFNEAMASDSGMMNLLSETASQFLRVWIH

>KAB1222018.1 Trans-resveratrol di-O-methyltransferase [Morella rubra]

MDPVQSQGASHELLEAQTHLYQHVFNYISSMSIKCVVQLGIPDIIHNHGKPITLPELASALEIHDPTKAGAMHRLMRLLVHNGFFAQTEVHGNQQQKQEEAYDLTPSSRILLKDNVMSLSKSVLASLHPAVMDPWHVLESWVRGDKVTPFENAHGMDFWNYANQNPAFGTLYDEALASDSGMVNLVLRDCKSVFEGLDTLIDVGGGTGTSAKIISEAFPNLKCTVLDLPRVVSDLPDSSNLKFVGGDMFELIPSADAILLKSVLNNWGDEDCLKVLKKCREVISNNGNRGKVIIIDIVLNDKKDKHELTEAKLYFDLLMMIVVSGRQRYEKEWEKLFLEAGFSHYKIIPLFGLRSLVEVYP

>KAB1222738.1 Trans-resveratrol di-O-methyltransferase [Morella rubra]

MDPVQAQAGASNELLEAQSHLYQHLGIPDIIHNHGKPITLPELSSALEIHDPTKAGAIHRLMRLLVHNGFFAQTEVHGNQQQEEELYDLTPSSRILLKDNVMSLSKSVLASLHPAIMDPWHVLESWVRGDKVTPFENAHGMDFWNYAVQNPAFGTLYDEALASDSGMVNLVLRDCKSVFEGLDTLIDVGGGTGTCAKIISEAFPNLKFTVLDLPRVVSDLRDSSNLKFVGGDMFELIPSADAILLKSVLTIGRRGLLEKMPRSYFKQRKLGKSVIIDIRYEKEWEKLFLEAGFSHYKIIPLFGLRSLVEVYP

>KAB1222739.1 Trans-resveratrol di-O-methyltransferase [Morella rubra]

MDPVQAQAGASNELLEAQSHLYQHVFNYISSMSIKCVVQLGIPDIIHNHGKPITLPELSSALEIHDPTKAGAIHRLMRLLVHNGFFAQTEVHGNQQQEEELYDLTPSSRILLKDNVMSLSKSVLASLHPAIMDPWHVLESWVRGDKVTPFENAHGMDFWNYAVQNPAFGTLYDEALASDSGMVNLVLRDCKSVFEGLDTLIDVGGGTGTCAKIISEAFPNLKFTVLDLPRVVSDLRDSSNLKFVGGDMFELIPSADAILLKSVLTIGRRGLLEKMPRSYFKQRKLGKSVIIDIRYEKEWEKLFLEAGFSHYKIIPLFGLRSLVEVYP

>KAB1222917.1 (S)-scoulerine 9-O-methyltransferase [Morella rubra]

MLLFFSGKEIVESMNMLKDSVLDPGRSAFYRTHGVHPYEYMEKKPTLKRSFNGFIESTSRAILDEVLKVYGGFEDVKELLDVGGCAGASLGKILSVHPHIRGLNLDLAHVIADAPTFPGMEHIAGDLFRSLPHTQTILLQGILHNWDDDHCKKLLRNCWEALPDDGKVIVVESVIPQVLANDIETRYAVTSDLCMMLLLVGGKERTISEIDILAKAVGFVETKDFPVAKSIHDIELRKKVMHHP

>KAB1222918.1 (S)-scoulerine 9-O-methyltransferase [Morella rubra]

MAARAAIELNVFSIIAEAGPGAHLSAAEITSKMETTNPKSASTNLDRLLRFLGANSLLTMAQRPFKNGEDIHHEWTYGLTKPTCSPETSSEAGISSTHGVHFYEYMEKKPTLKRLFNGFLESTSQAILDEVLKVYGGFEEVKEMLDVGGGVGASLVKILSVYPHIRGMNLDLAHVIADAATFPGVEHIAGDMFRSLPHTQTILLQRILHNWDDDHCKKLLRNCWEALPDDGKVIVVESVVPQVLANDPETRYAVAYDLGMMLLLAGGKERTISEFDNLAKAVGFVETKAFPIATSIHVIELRKKVMRHP

>KAB1223611.1 Caffeic acid 3-O-methyltransferase 1 [Morella rubra]

MVLKSALELNLIDIISEAGTGAFLSPSEIAARLPTRNPDSPVLLDRMLRLLASYSILKCSIATREDGEIERLYGVGPICKFLVRNSGGGSVSPLFLLHRDKVFMESWYHLNDAILDGGIPFNRAYGMTAFEYPGTDERFNQVFNQAMSNHTTLIMKKILEVYKRFDGLKVLVDVGGGLGVTLKSITSKYPQIKGINFDLPHVLADAPSYPGTDFVLSLAQEFYDMFKIDNSYPMTQYVANSHHGPLLFTSRPAWILHDWSDEHCLKLLKNCWKALPNSGKVIIVESILPVVPDSNVSSNIVFEQDLFMLAQNPGGKERTQKEFEALAEKCGFSGYEVVCSAYNSWVMEFHKRADP

>KAB1228317.1 (R,S)-reticuline 7-O-methyltransferase [Morella rubra]

MGVVMAKYKDGFDGVGSLVDVGGGTGGMIAEIVKSHPHIRGINFDLPHVVATAPWVLHDWGDEDCIKILWNCRKAIPEKRKKGRLIIVDIVLEKDSYDLFDETRMVFDLSMMAHTASGKEKTELEWKEILKEGGFPRYKINKIPTIPSIIEAYPI

>KAB1228320.1 (R,S)-reticuline 7-O-methyltransferase [Morella rubra]

MFEAIPCADAIFMKFVLHDWDDEDCIKILRNCQKAIPEKGGKLIIVDIVLEKDSHDLFDETRMVFDLMMMAHSSGGKERTELEWKELLKKGGFPCYKITKIPAIPFIIEAYPM

>KAB1228322.1 (R,S)-reticuline 7-O-methyltransferase [Morella rubra]

MACTAKIMMAVVLAEYKDGFDCVGSLVPDVGSGGTGGMIAEIVKSHPYIRGINFDLPHVVAAAPVRERVSHVGGGMFEAGAIPDADAIFMKGKLIFVDIVLEKDSHDLFDKTCMVFDLLMMAHTAGGKERTELEWKELLTEGGFRCYKIIKIPTIPSIIEAYLIRSLVQRFSN

>EXB31255.1 8-hydroxyquercetin 8-O-methyltransferase [Morus notabilis]

MEKRLRSFRNLDTDLQINEEKATELLGAQAHIWNNIFNFINSMSLKCAVQLGIPDTIHKHGKPMTLSKLIGALPVHQNKAPYVYRLMRILVHSGFFSLQTIGESDHQEEEGYVLTDASKLLVGDNPLSVAPFLLAMLDPVLTKPWDFLSTWFQNDDPTAFDTVNGLPFWKYGGQEPKMANFFNDAMASDARLVISVVIENCKWVFDELESFVDVGGGTGTVAKAIAATFPKMECTVFDLPHVVGDLQGKDNLKFVGGDMFQQVPPADAILLKWILHDWSDDECVKILKKCKEAITSKGSKKGKVIIIDIMIENKDEDDKSYETQLFFDMMMMVLATGQERNEKEWAKLFQDSGFSDYKIIPILGLRSLIEVYP

>EXB37024.1 (RS)-norcoclaurine 6-O-methyltransferase [Morus notabilis]

MDSADDENLRGQAEIWKYMFRFVDSMALRCVVELRIPDIINSHGGPITLAQIVSSIPEASSPDISCLARIMRFLVRRNIFTAHHPSNGSEDITLYGLTHLSRWLLTCEQTLAPMLLWQTDPTLMTPMHYFSGCVKGGGVPFEKAHGRGMFAFMSENEEFGKACNDAMECTARIILKEILSDSQCGDHVFGCLGSLIDVGGGTGGALSEIVKSYPHIKGINFDLPHVVSKAPVYAGVSHVAGDMFESIPSADAWIMHDWMDEDCVKILKNCRKAVPEKSGKVIIVDVVLDLKGNGLFDDMGLILDLLMAAHSLGGKERTEKEWKKILKEGGFSCYKIIRISTFLSIIEAYPE

>EXB38591.1 Caffeic acid 3-O-methyltransferase [Morus notabilis]

MDYGGDTGVEHVGGDMFVSVPKADAVFMKLNMKGFYIDVESCQTIVMQWICHDWSDEHCLKFLKNCYDALPENGKVIVAECILPDAPDSSLATKGVVHIDVIMLAHNPGGKERTEKEFEALAKGSGFQGFRVLCDAFNTYVMEFLKKPF

>EXB54962.1 Isoflavone-7-O-methyltransferase 8 [Morus notabilis]

MSKSDDNEMMSSSELLQAFSLLRSCSISHIKSMSLKCAIELGIPYIIHNHGQRAITFSKLIESLPVHPSKTHCIYRIMRLLVHSGFFATQKPDQEEEEEAYTLTNASRLLLTDTPVNMESYFLLPLLPQMIGPWQSLSTWLQSNDHTLFKTVYGGTIWDQLADDPELNYRFNEAMASDSRLISKVILDSEYIRVFEGLKCLVDVGGGSGTLAKAIANTFPHMKCTVFDLPHVVANVQGTTDNLSFIGGNMFSDPIPPADAILLKMIMHNWNDEDGSRILKRCREAILSNGNGGKVIVIDTVIGNEKTQDLTETKLCNDVIMMTLTGKERSLMQWEKLILTAQFSHYKITPIFGSVNSIIEVYP

>EXB54963.1 Myricetin O-methyltransferase [Morus notabilis]

MSSSDELIMSSNELVEAHSLLWHSGLDYMKSMSLKCAIELDIANVIHKHGQPIPFSKLVDSLKIPPSKANFLYRIMRILVHHGFFTAQKVNGGKTQEEEEAYSLTNASRLLLTDEPALIMNMKSLSLFFLLPAVVNSWQSWSTWLKNSDDMTLFETAEGKTWWEYASQEPGLNHLFNDAMANDSKLTAKIILEECKEVLEGLKNLVDVGGGTGTMAKAIVNAFPHIKCTVLDLPHVVANVQETSTDNLNFIGGDMFSEQIPPADAILLKNVLHDWTDEACVTILKRCREAVLSNGNKGKIILIEIVVETQKTTNKETTELQLLNDVFFMGLSAKERTLLEWEKLFSAAGISHYKIIHTFGIRSVIEVYP

>EXB54964.1 Tabersonine 16-O-methyltransferase [Morus notabilis]

MEVFSEEVMSSSELLQAHSLLWSNSVHIVRNLSLKCVIELGIPDIIHKHDQPISLSKLISALPIHPSKAHCIYRIMRILVHSGFFSTRKLNGEEEESYSLTNVSRLLLSDGPAFIKMKSFFLFHLLPEVAAPWHSLSTWLQHSDGTTFEHGNGKNFWDCLADEPKHIHLFNEAMANDSQLIAKVILTEYKNVFEGLETLVDVGGGTGTMAKAIASTFPHIKCTVFDLPHVVANLEATDNLNFIGGNMFTDTIPPTDGILLKWILHDWNDEDSVSILKRCREAILRNGKGGKVIVIEMVIENQNTNEITGIQLSYDVMMMGSFFGKERNLVEWEKLFFEAGFSHYKINAKMGARSLIELYP

>EXB54965.1 Tabersonine 16-O-methyltransferase [Morus notabilis]

MDVGDEVSANELLQAHGLIWNCSLNFIKPMSLKCAVELGIPDIINNHGQPITLSKLISSLPIHSSKAHCIYRLMRILVHSGFFATQKVDNKIDEGEEEEEEEAYSLTIASRLLLKDGPWRATPFFLVQLEPHLMTPWHFVSTWLQNSDPTPFEMVHGKTFWDGVGDEPRLKYLFTEAMATDSQLMVKVIVEECKQVFEGLTSLVDVGGGTGIVAKAIAKTFPNIMCTVLDLPYVVANSQGAKNLNFIAGDMFKEIPPANAILLKWILHDWSDEEAVMILKRCREAIWSKEKGEKVIIIDMVIENPKMDKKSAETQLYFDMLMMVNLTGRERNQKEWESLFVAAGFSHYKITPIVGLRSLMEVYP

>EXB55622.1 3'-hydroxy-N-methyl-(S)-coclaurine 4'-O-methyltransferase [Morus notabilis]

MKLDEASLRGQAEVWKYMFSFADSMAVKCAVELRIPDIIKSNGGPMSLAEIVAAFPRASSPDISCLSRIMRLLVRLKIFTARQSSDGSGDSATLYGLTQSSRWLLRDTSDDQLTLAPMLLLENHPLLMAPWHCFSQCVKDGGLPLEKAHGPEIWDFVTGNGEFNKMFNRGMECASRIVMRAVLSEYYKDGFGCLGSLLDVGGGTGGDVAQIVKSCPHIIKSINFDLPDVVAKAPEHPGVSHVGGDMFKSWIMHEWSDEDCVKILKNCRKAIPEKSGKVIIVDIVLEPEGTGVFDETGLVFDLVMVAHTSGGKERTENEWKNILKEGGFPRYKVTKIPALTSIIEAYPQ

>EXB55624.1 (RS)-norcoclaurine 6-O-methyltransferase [Morus notabilis]

MQTHPWLMAPWHCFSRCVKEGGSAFQKAHGREIWDFASENPEFNKLFNDGMECTARIILKAILSEYRDGFSSLGSLIDVGGGTGRALSEIVKSHPHINGINFDLPHVVAKAPVYPGVSHVGGDMFESIPNADAVFMKASLATDLNQLSTWIMHDWGDEDCVKILKNCRKAIPEKSGKVIIVDLVLNPEGNTLFDDMSLIWDLVMVAYASGGKERTENQWKKILIEGGFPRYKIIKIPSLTSIIEAYPE

>EXB55626.1 (RS)-norcoclaurine 6-O-methyltransferase [Morus notabilis]

MESNEASLRDREEIWKYMLNHADSMAIKCAVELRIPDIINSHGGPMSLAQIAAAIPDTSSPDISCLARIMRLLVRRNIFTAHQSSDGSGDSTTLYGLTHSSRWLLSDTSDDQLTLAPFFLMETHPWLMAPWHCFSRCVKEGGSAFQKAHGREIWDFASENPEFNKLFNDGMECTARIILKAILSEYRDGFGSLGSLVDVGGGTGRALSEIVKSHPHINGINFDLPHVVGTAPVYPGVSHVGGDMFESIPNADAVFMKWIMHDWGDEDCVKILKNCRKAIPEKSGKVVIVDLVLNPEGNTLFDDMSLIWDLVMVADASGGKERTENQWKKILKEGGFPRYKIIKIPSLTSIIEAYPE

>EXB55629.1 (RS)-norcoclaurine 6-O-methyltransferase [Morus notabilis]

MESDEASLRGQAEIWKYMFNFADSLALRCAVELKIPDIINSHGGPITLAQIASSIPNTSSDPDISCLARIMRLLVRRKIFTAHQPSDGSGNSTILYGLTHSSKWLLRDTLGDDQHTLAPIFLMETHPWLVSPWHCFSRCIKEGGIAFEKSHGREIWDFASENPEFNKLFNDGMECTAKIILKAILSEYKDGFDYLGSLVDVGGGTGGALSEIVKSHPHIKGTNFDLPHVVATAPAYPGVSHVGGDMFESIPNADAWIMHDWGDEDCVKILKNCRKAIPEKSGKVIIIDVVLNPEGNGLFDDTGLIFDLVMVAHASGGKERTENEWKKILKEGGFSRYKIVKIPALTSIIEAYPQ

>EXB57403.1 Caffeic acid 3-O-methyltransferase [Morus notabilis]

MDSKETQIQSSLSQEDEACLKAMLLCSSHVLHLVLNAAIELDLFGIMAGAGPGPEAKMSPSDIASLLPIETPNGPSMLDRMLRLFASYSLLTCSTSTSQDGKVERLYGLTPTAKFFIRGRDKNELASLYRLHCHPTTLEVWFHMKDVILEGGSTFEKAHGMSIFKYMDSNESFRNIFHSAMVARSTIIMEKVLEIYNGFQGLTSLVDVGGGTGHCLHMIISKYPSILATNLELPHAIQNAPPYPGIKHVAGDMFESIPNSDAIMIKDVLHNWEDVDCIKLLRNCHEALPQENGKVIIIDIIMPETPDSSMASKYASELDNIMLTQPGGRERSRRELQALCEESGFSRFRIACFALSVWAVVEFYK

>EXB57805.1 Tabersonine 16-O-methyltransferase [Morus notabilis]

MDYFSEDVMSSSELLQAHSLLWSNSVHIVRNLSLKCVIELGIPDIIHKHGQPITLSKLISALPIHPSKAHCIYRIMRILVHSGFFSALKVNGEEEESLSLTNVSRLLLSDGPASIKMTSFFLFHLLPEVAAPWYSLSTWLQHSDGTTFEHGNGKNFWDCLADEPKHIHLFNEAMANDSQLIAKWILHDWNDEDSVSILKRCREAILRNGKGGKVIVIEMVIENQNTNEITGIQLSYDVMMMGSFFGKERNLVEWEKLFFEAGFSHYKINAKMGARSLIELYP

>EXB57807.1 Myricetin O-methyltransferase [Morus notabilis]

MAKAIASSFPHIKCTVFDLPHVVANLEGTDNLNFIGGNMFTDTIPPTDGILLNLKGGSASDYPRPSFDLHLPFKTCALPFPSMVKAGEEISRSIRSVRRIPKDQASEASKEEALLSEYDPSVRAYHKQARKKRRQPGEAAKLQRQGGSLSAYERKSVCRLALRRETLVEVGSCSVILSLGISALMLDIIDGLLFLSELPDPRASWRGSTNGTSYDDMVYIPVVRTEERVTPVTPPPAAPTMAVLSPLPSSFPAPVEITHATNATTSAASSIAGDSITIPAIAVETAPDGTEFNKSFNPSLNPSYQKRENLRYMPSVG

>EXB58750.1 Tabersonine 16-O-methyltransferase [Morus notabilis]

MDYFSEDVMSSSELLQAHSLLWSNSVHIVRNLSLKCVIELGIPDIIHKHGQPITLSKLISALPIHPSKAHCIYRIMRILVHSGFFSALKVNGEEEESLSLTNVSRLLLSDGPASIKMKSFFLFHLLPEVVAPLHSLSTWLQQSDGKTFEHGNGKNFWDFLADEPKHIHLFNEAMANDSQLIAKVILTEYKNVFEGLETLVDVGGGTGTMAKAITSSFPHIKCTVFDLPHVVANLEGTDNLNFIGGNMFTDTIPPTDGILLKVRVYSTII

>EXB67902.1 8-hydroxyquercetin 8-O-methyltransferase [Morus notabilis]

MSLKCAIQLGIPDAIYSHGKPMTLSQLISAFPIHQNKKPDSHLIYRLMRILVNSGFFALQEVVVGKTDLLEEGYVLTNASKLLLRDNPLSVTPFLLLGNLFNEAMASDARLVTSVVIDKCKSVFEGLESLVDLAGGTGTVAKAIADAFPHVACTVFDLPHVIADLQGSKNLKFVGGDMFDKIPPADAIFMKWILHDWSDEDCVKLLERCKVAITCKGNEKGKVILVDIVIVEDQNNKDIGYDNHAHDHDHQSYETQLFFDMLMMVLVRGRERKETEWAKLFLDAGFSTYKITPILGVKSLIEVFP

>EXB67905.1 8-hydroxyquercetin 8-O-methyltransferase [Morus notabilis]

MEKVVRNSGHDLEIHSTELVKAQAHIWNYIFNFINSMSLKCAVQLGIPDAIHSHGKPMTLSQLISALPIHQNKKSDSRLIYRLMRILVNSGFFVLQEVVVGETDLLEEGYVLTTASKLLLRDNPLSVTPLLLSMLDPIMTKPWHSLSTWLQSDDPTPFAATHGMGIWDYNEYDPRLGNLFNEAMASDARLVTSVLIDKCKSVLEGLESLVDLAGGTGTVAKAIAYAFPHLACTVFDLPHVIADLQGSKNLKFVGGDMFDEIPPADAIFMKWILHDWSDEDCVKLLERCKVAITCKGNKNGKVILVDMVIVEEQNNKDIGHDHHAHDHDHQSYETQLFFDMLMMVLYPGRERKETEWAKLFSDAGFNTYKITPILGVRSLIEVFP

>EXB68019.1 8-hydroxyquercetin 8-O-methyltransferase [Morus notabilis]

MPDWSDEDCVKLLEGCKKAITCKGNFKKRNKVIIVDMIVMENQKSKDIIDHVHDDRSYETLLFFDMIEMIFLPGTRDRTEKEWAKLFSNAGFTTYKITPLSGVRSLIEVFP

>EXB75147.1 (RS)-norcoclaurine 6-O-methyltransferase [Morus notabilis]

MEGIDHHVDEMQRGQADIWQVMYSHMDSLVLKCAVELRIADIIHSHGCPVTLSQIASSISGSTSPNIHYLERILRLLVCKKIFTAHRDDHDSSSGDHPHHHQTRYGLTEKSRWILWESEPSLAPVIMMQNRRLVIDAFDHLSDSVRHGVVPYKAAFGHDFVGESTVDPKSKKWFKDGMINLANMVMDPVLSALKNEFSCMGSLIDVGGFTGETIYEIVKAHPHIKGFNFDLPDIIAMAPSYDGVTHVAGDMFEAIPCADAIFMKKVLNNWTDEQCIKILRNCRQSIPEKTGKVIIVDIVLESLESNDVVFLYDEMRIRFDLGLLAFRGVGRERTELEWKKLLEEAGFPRNKIIRIPAMPSIIEAYPV

>EXB75933.1 Caffeic acid 3-O-methyltransferase [Morus notabilis]

MEDESSEKRNQARLKVLELANMISVPMSLNAVVRLGVPDAIWQSGSNSPLSASQILTRVLPSSSSSAASADPDNLQRLLRTLTSYGVFSEHISGEIRKYSLTDVGKTLVTDSDGLSYAPYVLQHHQDALMRAWSLLHEAVVDPTTEPFAKANGEPAYKYYGKMPEMNGLMQRAMSGVSVPFMKAILNGYDGFEGVRRLVDVGGSAGDCLRMILQKYPNVTEAINFDLPEVVARAPHVPGVTHVGGDMFKLIPEADAIFMKWILTTWTDDECQAIMESCCKAVPGGGKLIACEPVLPKETDDSHRTRALLSGDIFVMTIYRAKGKHRTEDEYRKLGLAAGFPHFQAFYIDYFHTVLEFTK

>EXB83827.1 Anthranilate N-methyltransferase [Morus notabilis]

MENNPNVLNDVYRREEEENYSFAMQLATAPALSMSLHAAIELGVFDIIAKAGEGAKLSPAEIVEQLAANNPEAPIMLDRILRMLASHSILSCSVVGDGSSEPKFRRLYGLRPVSKYFVTNEYDGSFGPMLALVHDKILMDSWFQLKDAILEGGVPFKRVHGTHSFEYLGLDQRLNQLFNKAMDNASTIIVKKLLKYYKGFNHLQKLVDVGGGLGVTLNLITSKFPHVKGINFDLPHVVEHAPSYPGVEHVGGDMFDKVPIGDAILLKSVLHDWNDEHCLKLLKNCSEAIPTNGKVIVVDVIFPVKPETSSHAKSNSQLDVLVMAEHQLGAKERSREEFLALATGAGFREIKFEYFMGDYWIMEFFK

>EXB93812.1 3'-hydroxy-N-methyl-(S)-coclaurine 4'-O-methyltransferase [Morus notabilis]

MEEIQRDELIKWGIPEEEIEEEERAKVEIWKYIFGFVEMAVVKCAIELGIADVIATHGRPMTLLELSSALDCDPQNLHRIMRFLVNRAIFKEILIRNDAVPTAYYAQTPISRRLMRTGENSMAAFILLESSPPMLAPWHGLSARVMARGTSAFEAAHGEDIWKYAEANPGHARLIDEAMACDARVAVPAIIDGCSDVFDGVRTLVDVGGGNGTALRMLVKGCPWISKGINFDLPHVASVAEKCEGIEHVGGLEVFGRKCTASRKDFVVHFEDEWCSLDNAIKEIQHGGNNVKEEVFECISPIGVSNSLELSELFIVWWVLHDWADDECIHILKKCRAAIPKDIGKVIIAEAVIGNKEQQKEDNKLKDVGLVLDMVMMAHTTKGKERTLDEWAYVIREAGFSRHTVRSIAAVQSVIEAFPA

>EXB94570.1 3'-hydroxy-N-methyl-(S)-coclaurine 4'-O-methyltransferase [Morus notabilis]

MGEAQRNHDHLTCGLSKEEKEEEEERARVDISKYIFGFVEMDVVKRSIELGIADAIANHGRPMTLLGLSSSLDCDPQNLQRIMRFSVNRGIFKEMTRNDAVPAAYYSQTAISRRLRRTGEDSMAALILLHSSPPMLAPWQGLRACVMAKGSSAFEAAHGGDVWRYAEVNPAHGRLINGTMACGARATVPEIINGCSDVFDRVETLVDVGGGNGTTLRMLVKACPWISKGITFDLHHVVSIAEELEWSRQVSSPSPIDAVSLVDSSASQFQFIFSIKYCTRIRRRDDRGANFNLNIVHVYGDATIVE

>EXB94571.1 3'-hydroxy-N-methyl-(S)-coclaurine 4'-O-methyltransferase [Morus notabilis]

MEETQRDHLTWGLLEEEREEEKQKKFDMSKYYFGGDVEMAVVKCAIDLGMADAIANHGRPMTLLELSSALDCDLQNLHRVMRFLVNHGIFKEIIIRNEDTVPTAYYAQTHSSRRLMRTGENSMAAFILMQSSPPAKAAWHCLSACVKGKATSAFEVVHGEDVWKHAEANPAQSQLINEAMACLARVIVSAIIDGCLDMFEGVETLVDVGGGTGTIMRRLVKACPWISKGITFDLPHVVSLAEQCEGIEYVGGDMFEFIPKADAVLLVAVLHDWGDDECIRILKKCRKAIPKDKGKVIIVEVVIGNKEQQKEDNKLNDVGLVLDMAMMALTHKGKERTLDEWAYVLREAGFSRHTVRSIAAIQSVIEAFPA

>EXB95356.1 3'-hydroxy-N-methyl-(S)-coclaurine 4'-O-methyltransferase [Morus notabilis]

MKETQRDHLTWEEREEEKHEKVDMSKYYFGGVVEMAVVKCAIELGMADAIANHGRPMTLLELSSALDCDLQNLHRVMRFLVNHGIFKEIIIRNEDTVPTAYYAQTPSSRRLMRTGENSMAALILMQSSPPAMATWHCLSACVKGKATSAFEVVHGEDVWKYAEANPAHGQLINEAMACKARVMASAIIDGCLDVFEGVETLVDVGGGTGTIMRRLVKACPWISKGITFDLPHVVSLAEQCEGIEYVGGDMFEFIPKADAVLLVTVLHDWGDDECIRILKKCREAIPKDKGKVIIVEAVIGNKEQQKEDNKLNDVGLVLDMVMMAHTYKGKERTLDEWAYVLREAGFSRHTVRSIAAIQSVIEAFPA

>EXC02140.1 Caffeic acid 3-O-methyltransferase [Morus notabilis]

MAPLMFLMQDKVYMDIWHHVKDAVMEGGHPYERAHGMNMVEYVRKDDRFGELFKCSMKEFNPILMKRILEIYQCFEGIEHCAGDMFVAIPKGDAIFMKWMLHAWDDENSLVILQNC

>EXC13656.1 Caffeic acid 3-O-methyltransferase [Morus notabilis]

MASSLKLANGGPKVLCHDRKEEEEAFSQAMCLVMSSGMPVCLKTAFELRVFDIIAKAGEGAKLSSAEIVAQMPTNNPDAATMLDRILRLLASHSVLSCSVVSDDNGCNVQRLYGLSPVSKYFVTNEDGVSLGHFIAMVQDKVFVDTWPQLKASILEGGNAFNITYGMHAFEYIGTDLRFNKVFSKAMHSHSTVVMKSILEHYKGFENIKQLVDVGGGLGVTLNQITSKYPHIKGINFDLPHVVENAPSYPGVEHVGGDMFQNVPCGDAIFMKWILHDWTDEHCLKLLKNCYKAISNDAKVIVVDSILPIMPETNFAVKSVEHVGGDMFQNVPCGDAIFMKWILHDWTDEHCLKLLKNCYKAISNDAKVIVVDSILPIMPETNLRASWHLIRACWLHIQGERSGANKSS

>EXC13658.1 Caffeic acid 3-O-methyltransferase [Morus notabilis]

MASSLKLANGGPKVLCHDRKEEEEAFSQAMCLVMSSGMPVCLKTAFELRVFDIIAKAGEGAKLSSAEIVAQMPTNNPDAATMLDRILRLLASHSVLSCSVVSDDNGSNVQRLYGLSPVSKYFVTNEDGVSLGHFIAMVQDKVFVDTWPQLKASILEGGNAFNITYGMHAFEYIGTDLRFNKVFSKAMHSHSTVVMKSILEHYKGFENIKQLVDVGGGLGVTLNQITSKYPHIKGINFDLPHVVQNAPSYPGVEHVGGDMFQNVPCGDAIFMKWILHDWTDEHCLKLLKNCYKAISNDAKVIVVDSILPIMPETNFAVERLGI

>EXC13660.1 Caffeic acid 3-O-methyltransferase [Morus notabilis]

MASPLELGNTRKVVDDQRKEEEQEEHFFSYAMQLATSTVLSMSLQSAFELGVFDIIAKAGEGAKLSSVEIAAHLPTTNPEAPIMLDRILRLLASHSVLNCSLDGDNGDIGSNFQWLYSLSPVSKYFVTNEDGVSLGPFVALVQDKVSFESWPQLKNAVLEGGIPFNKVHGMHAFEYTGVDSRFNSVFNAAMYNHTTIVIKSILNLYKGFEHLKQLVDVGGSLGVTLNQIISKYPHIKGINFDLPHVIGQAPSYPGVEHVGGDMFESVPCGDAIFMKWILHDWTDEHCLKLLKNCYEAIPDDGKVIVVEAILPVSGTSSAAKTAFQFDVGMMTQNPGGKERSQQEFVALAVGAGFSGIRFECFVTNYWVMEFFK

>EXC22153.1 Tabersonine 16-O-methyltransferase [Morus notabilis]

MAFSEEVMSSSELLQAHSLLWSTSVNYAKYVTVKCAIELGIPDIIHKHGKPITLSKLINALPIHPSKTHCICRIMRILVHSGFFSTGRQVEEEEEEEEESYSLTNASRLLLSEGSDFTKMKSFVLFHLLPEAAAPWHSMSTWLQSSDGSTFEHQKGKTFWDCLADEPSQNRLFNEAMANDSQLIAKLTLTECKDVFEGLTTLVDVGGGTGTMAKAIASTFPHIKCTVFDLPQVVANLEGNDNLNFIGGNMFSDTIPTADAILLKWILHDWNDEESLAILKKCREAILKNEKGGKVIVIETVIDSKNTDEITGIQLCNDMIMMVSFSGKERNLREWEKLFFSAGFSHYKINRKLGVRSLIELYP

>EXC27579.1 8-hydroxyquercetin 8-O-methyltransferase [Morus notabilis]

MEKVRSSGHDLQVGATEQLKAQAHIWNQIFNFINSMSLKCAIQLGIPDAIHSHGKPMTLSQLISALPIHQNKKLDSHLIYRLMRILVNSSFFSLQEVVGETDQLEDGYVLTNSSKLLVRDNPFSLTPILLTVLDPIMTKPWHSISTWLQSDDPTPFATTHGMEFWDYTVRDPRFGNVFNEAMASDARLVVSVLFDKRISVFEGLESLVDLAGGTGTVGKAIADAFPHVECTVFDLPHVIADLQGSTNLKFVGGDMFDEIPPADAIFMKWILHDWSDEDCVKLLKRCKVAITCKGNKNGKVILVDMVIVEKQNNKDIGHDHDHQLYETQILFDMLMMVSFPGRERKETEWAKLFSDAGFSTYKITPILGVRSLIEVFP

>EXC27580.1 8-hydroxyquercetin 8-O-methyltransferase [Morus notabilis]

MEKVVRSSGHDLEINATELVKSQAHIWNQIFNFINSMSLKCAIQLGIPDAIYSHGKPMILSQLISSLPIHQNKRPDSRLIYRLMRILVNSGFFALEEVVVGETNLLEEGYVLTNASKLLLRDNPLSVTPFLLSMLDPIMTKPWHSISTWLQSDDPTPFATTHGKEFWDYNEYDPRLGNLFNEAMASDARLVTSVVIEKCKSMFEGLESLVDLAGGTGTVAKAIAYAFPHLACTVFDLPHVIADLQGSKNLKFVGGDMFDEIPPADAIFVKWILHDWSDEDCVKLLERCKVAITCKGNKNGKVILVDMVIVEEQNNKDIGHDHHAHDHDHQSYETQLFFDMLMMVLLSGRERKETEWAKLFFDAGFSTYKITPILGVRSLIEVFP

>EXC30549.1 Tabersonine 16-O-methyltransferase [Morus notabilis]

MLSFISSMSIKSAVQLSIPDIINNDGQPITLSELASALDIHPTKAGFIHRLMCLLVHHGFFNSAKIGHDEEAYDLTPSSRLLKDKIPSLSPFVMSLLDPALITPWHLLGNWFQKEEKMPPFESAHGMSFWAYVNKNSDFNNHFNQAMAIDSGMMNLVVKDCKPVFEGLSSLVDVGGGTGTISKIIIETFPHIKCTVLDLPHVVANLPATENLKFIGGDMFQGIPSADAILLKLMLHCWSDEECLKVLKICRVAIPSNGGKVIITDIVINKDKDDPELTEGKLYFDLLMMILVTGRERSEKDWEKLFLEAGFSHYKITPLFGLRSLIEVFPRKQLSSNESVHLRTSLISMVARNSMQLFVFLPYVYELLK

>EXC30550.1 6a-hydroxymaackiain methyltransferase 1 [Morus notabilis]

MDLLQGQGSNELFQAQSHLYSHIFRFISSMSLKCAVQLGIPDIISNHGQPITLSELVSALDIHPTKAGFIYRLMRLLVHDGFFTSTKIGENEEEEEEEASYDLTPSSRLLLKDKIPSLSPFLVAMLEPAITTPWHSLGNWFQREEDMTPFESAHGISFWAYGNKNSEFGNLFNQAMASDSGMMNLVVKDCKLLFEGLSSLVDVGGGTGTVSKIITEAFPHLICTVLDLPHVVANLPDTENLKFVGGDMFQDIPSADAIFLKWLVHAWSDEEGLKVLKKCREAIPSNGRLHAWSDEECLKVLKKCREAIPSNGRGKVIIIDIVINKEKDERELTQGKLYFDLLMMVAVSGRERSKKEWEKLFLEAGFSHFKITPLFGLRVKNWTEIGDEVEISSGPSRPDTGVIVGMGLM

>EXC30551.1 Chavicol O-methyltransferase [Morus notabilis]

MLDPILFAPCHVLTKRLQDDTAVTSFDSFHGLNISDYIKKSFEFEDLIDSAMDSDSSKMNLAIKDLKKVFDGVTSLVDARGKRSVFCSIIIDAFLYMQCTVLDLPHVVANLEPIQGNHGKKVIIIDIVMSENDGTELMETKLYYDIMAMMLMNGKERSERDWQKLIAKAGFSLCEITVKVRIRSLPRFFLDGEGAWLDWQRTVCRVGPTRCLKSTLSMCFWLYVVYCVPCIPVCGDWIGSGICFMNLEL

>EXC32921.1 Caffeic acid 3-O-methyltransferase [Morus notabilis]

MASPLELGNDRKFLDSEGRNKEEEDKENFSYAMQLVFSTVLSMSLQTAIELGVFDIIAKAGEGAKLSPAEITAQMTTDNPEAPIMLDRILRVLASHSILRCSVVGDVNSESDFQRLYSLGPVAKYFATNEDGVSLGPLMALIQDKIFLDSWPQLKEAVLKGGIAFNRVYGTHAFEYPGLDPRFNHVFNKAMYNQTTIVIKNILKFYKGFKDLAKLVDVGGGLGVTLNLITSKYPHIKGINFDLPHVIEHAPSYPGVEHVGGDMFEKVPTGDAIFMKWILHDWSDEHCLKLLKNCYKATPENGKVIVVESVLPVEAETNTAVKSTSQLDVLMMTQNPGGKERSTQQFLALATDAGFRGIKFEYFICNFWVMEFFK

>EXC61661.1 (RS)-norcoclaurine 6-O-methyltransferase [Morus notabilis]

MQKVLNNWTDEQCIKILRNCRQSIPEKTGKVIIVDIVLESLESNDVVFLYDEMRIRFDLGLLAFRGVGRERTELEWKKLLEEAGFPRNKIIRIPAMPSIIEAYPV

>MD00G1100700

MKNAIIEGGIPFEMAHGSHAFKYVGYDQRFNQAFNIGMNSITTMLVKKILEVYMGFENLKKVVDVGGGLGVAISLITTTYPHIKGINYDLPHVVKDAPKYPGVEHVGGDMFASILDADVVFMKGILHDWFDEDSIKLLKICYKALPVDGKVIVVDAVLPSIPDSKSITKSNYLLDVHMMVQTPGGKQRSAEEFMDLATAADFSAIKLACFAAHLGVVEFYK

>MD01G1048300

MPFWELSAKKQTFGDLFNEAMEANSKLIARVAVEECGGVFEGSKSLVDVEGGTGTMAKAIAYGFPNINCTVFDQPHVVADLEGTAHNLGFVGGDMFDKIAPANAILLKIKYLHLILDYWNDEESMKILNKCRESKLLSKNEGRKKIIIIDIVVGYNKKSIETQLMFDMLMMSIVTSKERSKS

>MD01G1048400

MGLNNGEVGAISHELLGAQAHVWNHMFQFINSMSLKCAVQLGIPDVIHNHGQPISLSELVARLNIHPSKAHFMSRLMRILVHSDFFAQHDHVHHDRDDAEEEVAVVLYSLTPASGLFLKDGPLNTTPFLLMILDQVITDPFHSMGNWLQMNCGDDLVPCTPFEVANGMPFWELSAKKPRFGDLFNEAMEADSKLIAGVVVEECGGVFEGLKSLVDVGGGTGTMAKAIANAFPNINCTVFDQPHVVADLEGMTHNLGFVGGDMFEEIPPANAILLKWILHDWNDEESVKILKKCREAIFLNKNEGRKKKIIIIDIVVGYADNKTKVDKKSIETQLMFDMLMMSTITGKERSESEWEKIFLAAGFSHYNITHMLGLRSLIEVYP

>MD01G1051800

MGLSNGEVGATSHELLGAQAQIWNHMFQFINSMALKCAVQLGIPDVIHNHGQPISLSELIAALNVHPSRAHFVSRLMRILVHSDFFAQHHHVHRGCDDMEEEEAVVLYSLTPTSRLLLKDGPSNTTPLILMILDPVLTTPFHLMGAWLQMNGGDDPATICTPFKMENGMPFWDLAAQEPRFGNLFDEAMEADSKLLGREVVEECGGVFEGLKSLVDVGGGTGTMAKAIANAFPSINCIVFDQPHVVANLLGSTHNLGFVGGDMFVEIPPANAILLKWILHDWSDEESVKILKKCREAILLSKNDEGNKKIIIIDIVVGHVDKKEKMVDKKSIETQLMFDMLMMSTVTGKERSESEWKKIFLAAGFTHYNITHTFGFRSLIELYP

>MD01G1051900

MSLSNEVGATSHELLGAQAQLWNHIFQFINSMSLKCAVQLGIADVIHNHGQPISLSELMAGLKVHPSKAHFVSRLMRILVHSNFFAQHHHVHHDRADVEEEETVVLYSLTPASRLLLKDGSLNTTPFLLMILDPVVTTPFHLMGAWLKINGGDDPAATCTPFEMENGMPFWELGAREPRFGNLFNEAMEADSKLIGRVVVEECGGVFEGLKSLMDVGGGSGTMAKAIANAFPNINCTVFDQPHVVAGLQGTTHNLGFMGGDMFEEIPPADAILLKWIMHDWNDEESVTILKKCREAISLSKNEGGNKKIIIIDIVVGYVDNKKKMMDKKSIETQLMFDMLMMSILPGKERSKSEWEKIFFAAGFTHYNITHTLGFRSLIEVYP

>MD01G1089600

MALQLEEEENFCYAMQLVFSSVLSMSMQSAIELGIFDIIAKAGPGAKLSSSEIAAHIGSGTRNSEAPMMLDRILRLLASHSILSCSAVANEEDGSDSQRLYSLGPVSNYFVTNEDGVSLGPLMALIQDKVFLDSWSQLKDAVVEGGIPFNKVHGTHAFEYPGLDARFNQVFNTAMFNHTTIVMKKILHLYKGFEKLTQLVDVGGGLGVTLSLITSKHPHIKGINYDLPHVVKHAPSYPGVEHVGGDMFASVPSGDAIFMKWILHDWSDQHCLKLLKICYNAIPEDGKVIIVEAVLPVMSETSTAVKSTSQIDVLMMTQNPGGKERSREEFMALATGAGFSGIKYECFVCNFWVMEIFK

>MD01G1089800

MFNHSTIVMKRILKLYKGFEHVTQLVDIGGNLGGAISLITSKYPHIKGINFDLPHVIKHASSYPGVENVGGDMFESIPNGDAIFLKFILHDWLDKDCIKLLKNCYNAIPDNGKVIVVEALLPIKPDSNLSVRTNGQLDLHMMTQTPGGMERSQEEFMALATASGFSGIRYECFTANLWIMEFYK

>MD01G1090400

MALQLEEEENFGYAMQIAFSSVLSMSMQSAIELGVFDIIAKAGPGAKLSSSEIVAHIGSGTRNSEAPMMLDRILRLLASHSILICSFVANEEDGSDSQRLYSLGPVSNYFVTNEDGVSLGPLMSFIQDKILLDNWPQLKEAVTEGGTPFKRVHGIHSFEYLDLDPRFNQVFSTGMFNHTAIVMKKIVHVYKGFEKLTQLVDVGGNVGVNLSVITSKYPHIKGINFDLPHVVKHAPSYPGVEHVGGDMLASVPSGDAIFMKWILHDWNDQDCIKLLKNCYNAIPDNGKVIIVEALLPAMPETSTAVKSTSQIDVLMLTLNRGAKERSREEFMALATGAGFRGIKYECFVCNCWVMEIFK

>MD01G1090500

MDSPMALQLEEEENFGCAMQLVFSSVLSMSMQSAIELGVFDIIAKAGPGANLSSSEIAAHIDSGTRNSEAPMMLDRILRLLASHSILSCSVVANEEDGSDSQRLYSLGPVSNYFVTNEDGVSLGPMIALAQDKVVLDSWSQLKDAVVEGGIPFHRVHGKQSHEYTGLDPRFNQVFNTAMFNYTTIVMKKILHLYKGFEKITQLVDVGGGLGVTISLITSKHPHIKGINYDLPHVIKHAASYPGVEHVGGDMLASVPSGDAILLKAILHNWSDQHCLKVLENCYNAIPDDGKVIIVEALLPVMPETSTAGKTTSLFDVLMMTQNKGGKERSREEFMTLATGAGFSGIKYECLVCNVWVMEIFK

>MD01G1149300

MGLSDGEVGSTSHELLEAQAQIWNHMFQFINSMALKCAAQLGIPDVIHNHGQPISLSELIAALNVHPSRAHFVSRLMRILVHSDFFAQHHHVHHDCDDVEEEEAVVLYSLTPTSRLLLKDGPSNTTPLILMILDPVLTTPFHLMGAWLQMNGGDDPATICTPFEMENGMPFWDLAAQEPRFGNLFDEAMEADSKLLGREVVKECGGVFEGLKSLVDVGGGTGTMAKAIANAFPSINCIVFDQPHVVADLQGTTHNLGFVGGDMFVEIPPANAILLKWILHDWSDEESVKILKKCREAILLSKNDEGNKKIIIIDIVVGHVDNKEKMVDKKSIETQLMFDVLMMSAFTGKERSESEWKTIFLAAGFTHYNMTHMFGFRSLIELYP

>MD01G1218900

MGSTPETQMTPTQVSDEEANLFAMQLATGSILHMVLKAAIELDLLEIMAKAGPGAFVSPADLSSQLPTKNPDSPVMLDRMLRLLASYSILTYSLRTLPDGKVERLYGLGPVCKFLTKNEDGASIGSLCLLIQDKVLMESWYHLKDAVLEGGIPFNKAYGMTAFEYHGTEPRFNKVFNKGMADNSTITMKKLLENYNGFEGLTSIVDVGGGTGAVLNMIVSKYPSIKGINFDLPHVIEDAPQYPGVEHVGGDMFVSVPKGDAIFMKWICHDWTDEHCLKFLKNCYAALPDNGKVIVAEYILPVAPDGSLATKEVVHSDAIMLAHNPGGKERTEKEFEALAKGSGFQGFRVVCSAFNTYAIEFLKKN

>MD01G1219000

MLVLGLCKYNLKDAVLEGGIPFNKAYGMTAFEYHGTDPRFNKGMANHSTITMKNLLENYNGFEGLTSIVDVGGGTGIVLNMIVFKYPSIKGINFDLPYVIEDAPQYPGVEHVGGDMFVSVPKGDAIFMKWICHDWSDEHCLKFLKNCYSALPDNGKVIVAECILPVAPDSSLATKGVVHIDAIMLAHNPGGKERTEKEFEALAKGSGFQGFRVVCSAFNTYAIEFLKKN

>MD01G1228900

MDSTRETQMTPTQVSDEEASLFAMQLASASVLPMVLKATIELDLLEIMAKAGPGAFVSPADLASQLPTKNPDAPVMLDRMLRLLASYSILAYSLRTLQDGKVERLYGLGPVCKFLTTNEDGVSIASLCLMNLDKVPVESWYHLKDAVLEGGIPFNKAYGMTAFEYHSTDPRFNKVFNRAMADISTITMKKILETYKGFEGLTSVVDVGGGIGAVINMIVSKYPSIKGINFDLPHVIKDAPQYPGVEHIGGDMFGTVPKGDAIFMKCVCHDWSDEHCLKILKNCYAALPDNGKVILAECILQVAPTSNLATKVVAHHDLIMLTNSPSGRERTEEEFEVLAKGSGFQGFRVMCPAFNTYVIELLKKN

>MD01G1229100

MGGGHLAFTAMAVGEQGNTYILPLSTHHLFHPQSSSSSSSSSSLSLSLSLSLSLSPHQHQHHQQHQIQKMGSTPETQMTPTQVSDEEANLFAMQLASASVLPMVLKAAIELDLLEIMAKAGPGAFVSPADLSSQLPTKNPDASVMLDRMLRVLASYSILTYSLRTLPDGKVERLYGLGPVCKFLTKSEDGASIGSLCLMNQDKVLMESWYNLKDAVLEGGIPFNKAYGMTAFEYHGTDPRFNKVFNKGMADHSTITMKKLLENYNGFEGLTSIVDVGGGTGAVLHMIVSKYPSIKGINFDLPHVIEDAPQYPGVEHVGGDMFVSVPKGDAIFMKWICHDWSDEHCLKFLKNCYAALPDNGKVIVAECILPVAPDSSLATKGVVHIDAIMLAHNPGGKERTEKEFEALAKGSGFQGFRVVCSAFNTYAIEFLKKN

>MD04G1141100

MASLEEPKGLPDIPLDDEARKEEESYCHALQLVVSSVLSFSMQSAIELGVFDIIAKEGQNAKLSSSKIAAHIGTKTPDGPMMLDRLLAVLASNSVLDCTVVDGKLDKCFRRLYGLTPVSKHFVTNEDGVSLAPVLTLVQDEAFLKGWRQVKDAVIEGEIAFDRAHGMHHFQYPSVDHRFNEIFNKAMFNHSTLVMKRILKLYKGFEHVTQLVDVGGNLGGAINLITSKYPHIKGINFDLPHVIKHAYSYPGVENVGGDMFESIPNGDAIFLKNILHDWLDKDCIKLLKNCYNAIPDNGKVIVVEALLPIKPDSNLSVRTNGQLDLHMMTQSPGGMERSQEEFMALATAAGFSGIRYECFTANLWIMEFYK

>MD05G1127400

MTLLELSSALSCDPSRLYRVMRVLVHLKIFKEKPTELGPKGYAQKPLSKRLLKSGENSVAAFILLESSPVMLASWHGLNLWSFAAANPDHSKLINDAMACDARVDVPTVIESCLEVFKGLETIVGVGGGNGTTLCLLVETCPWIRGINFDLPYVVYVAQECDRIENVGVGSSQLGRRRLYSYPKKCREAILNDRGKVIIIEAVIEDGKEENKLADVRLMLGMVMLAHTNTGKERTLKEWGYVLGEAGFSRYTITPIHAVQSMIQAFP

>MD05G1127900

MEEKQRELTWEEEEEGATAEMWKYVFGFTEIAVVRCAIDLGIAEAIENHGSPMTLLELSTALSCDPSHLYRIMRVLVHRKIFKEKPTQSGSNGYAQTPLSRRLLKSAENSMVDLLLMGSNPVMMAPYLRLSTQVQRNTSDAVFDEVHGSDLWNYGAENPDHGKAFNDSMACDARLAVPAALESCPEAFKGIETIVDAGGGNGTTARLLVEACPWITKGIIFDLPHVVSTIPECDRIEKIGGDFFKCIPKADAVVMMWILHDWTDEECISILKNCREAIPNDKGKVIIIDAVVEDNEKDYNKLTRVKLMLDIAMMNGTKGKERTAKEWEYVIREAGFSRHTITPTRTVQSVIQVFP

>MD05G1128000

MEDTQRVLTWEEEEEHAKVDVWKYVFGFVEIAVVKCAIELGIAEAIESHGSPMTLLELSSALSCDPSHLYRVMRVLVHLKLFKEITTNQLGSKGYAQTPLSHRLLKSGENSMVALILLESSPVMLAPWHGLSARIRGNISNQLFEEVHGEDLWSFGAANPDHSKLFNDAMTCDVKAAVPAVIKSCIEVFKGLETIVDVGGGNGTMLRLLVEGCPWIRGINFDLPHVVSAAQDCDRVENVGGDMFDRVPKADAVIIKGVLHNWGDDDCIRILKKCREAVPKDTGKVIIIDAVIDEKYEKEDKKLANLKLMLDMVMIAHTNKGKERSLKEWEYVLGGAGFSGHTITPVPAIQYSVIQAFP

>MD05G1308800

MEGDEARDLFGAQSHLYKHVFSFITSMSLKCVVQLGIPDIIDRHGQPITLPDLVTALQIHPAKTGNVHRLMRLMVHSGFFARKQVPKNHVEADEGEEEAYDLTPSSRLLLKDKVPSLSPFVVAMLDPAFAAPWQFLGNWFRGSEVTPFESAHGMGIWEYGEGNPEFNSLFNKAMASDSGMMNLVIRDCKPIFDGLSSLVDVGGGTGKVARILCDAFPQLKCTVLELPHVVADLPDSENLKFVGGDMFQVIPPADAVFLKLTLHALSDEECLKVLKKCREAIASNGQGKVIIIDIVINEEKDEHEITEAKLLFDLLMMVVVTGRERSEKDWKKLFLEAGFSGYKVTPIFGLRSLIEVFL

>MD07G1161000

MQSAIELGVFDIIAKEGPNAKLSASEIATHIGTMTPDGPMMLDRLLAVLASNSVLDCTVVKGKVEKCFRRLYSLTPVSKQFVTTEDGVSLGPVLALFQDKYAGVDPRFNKMFNKGMFNWTTILMKRILDLYKGFEHVKQIVDVGGSLGVAISLITSKYPHIKGINFDLPHVRKHAPSYPDDGKVIVVEALLPIKPETDLSVRVNALFDVHMMTQTRGGMERSPEEFTALATGAGFSGIRFECFAANFGVMEFYK

>MD07G1161100

MASLEEPKGLPKIPLDDEARREEESYCHALQLVVSSVLSFSMQSAIELGVFDIIAKEGPNAKLSASEIATHIGTKTPDGPMMLDRLLAVLASNSVLDCTVVNGKVDKYFRRLYSLTPVAKHFVTDEDGVSLAPVLTMVQDEVFLKSWPRVKDAVIEGGIAFDKAHGMHHFQYPGVDLRFNKMFNKAMFNHSTIVMKRILHLYKGFEHVKQLVDVGGNLGGAISLITSKYPHIKGINFDLPHVIKHATSYPGVENVGGDMFESIPNGDAIFLKFILHDWLDEDCIKLLKNCYNAIPDDGKVIVVEALLPIKPDNNISVRTNAQLDLHMMTQTPGGMERSQEEFMALATGAGFSGIRYECFTANLWVMEFYK

>MD07G1300000

MTNFEYHGTDPRFNKVFNRAMADHSIIAMKKLLVTYKGFEGLTSVVDVGGGTGSVIHMIVSKYPSFKGINFDLPHVIKDAPQYPGVEHVGGDMFNCYATLPDNGKVILVDIIIPVSPNSSLATKLANHVDMIMLAHNPGGKERTEKEFEALVKGSGFQGFRVMCSAF

>MD07G1300200

MCSTGENQMTPTQVSDEETNLFAMQLASASVPPMVLKAALELDLLEIMAKAGPGAFVSPADLASQLPTKNPDAPVILDRMLRLLASYSIFTYSLRTLPDGKVERLYGLGPVCKLLTKNEDGVSIAPHLLLTQDKVLVESWYHLKDAVLEGGIAFNKAYGMTTFEYHGTDPRFNKVFNKAMADQSSIAMKKLLETYKGFEGLTSVVDVGGGIGSVIHMIVSKYPSIKGINFDLPHVIKDAPQYPGVEHVGGDMFVSVPKGDAIVMKHICHDWSDEHCLKLLKNCHAALPDNGKVILVEIIIPVSPDSSLATKLANHIDVLMLANNPGGKERTEEEFEALAKESGFQGFRVMCSAFHSNVIEFLKKNLI

>MD07G1300500

MGSTVETQMTPTQVSDEEANLFAMQLASASVLPMVLKAALELDLLEIMARAGPGAFVSPADLASQLPTKNPDATVMLDRMLRLLASYSILTYSLRTLRDGNVEQLYGLGPVCKFLTKNEDGVSIAPLCLMNQDKVLMESWYHLKDAVLEGGIPFNKAYGMTAFEYHGTDPRFNKVFNRGMAAHSTITMKKLLETYKGFEGLTSVVDVGGGTGAITNMIVSKYPSIKGINFDLPHVIEDAPQYPGVEHVGGDMFVSVPKGDAIFMKFICHDWSDDHCLKFLKNCYTALPDNGKVILAECILPVAPDSSLATKGVVHIDVIMLAHNPGGKERTEKEFEALAKGSGFKGFRVICSAFNTYVIEFLKKI

>MD07G1300700

MGSTGETQMTPTQVSDQETNLFAMQLANAPVLPMVLKTALELDLLEIMAKVGPGAFVSPADLASQLPTKNPDAPVMLDRMLRLLASYSILTHSLRTLPDGNVERLYGLGPVCKFLTKNEDGVSIAPLCLMSQDKVLMESWYHLKDAVLEGGIPFNKAYGMTAFEYHGTDPRFNKVFNRGMAAQSTIIMKKLLETYKGFEGLTSVVDVGGGTGAVVNMIVSKYPSIKGINFDLPHVIEDAPQFLGVEHVGGDMFVSVPKGDAIFMKSICHDWSDDHCLKFLKNCYTALPDNGKVILVESILPVAPDSSLATKVVVNLDVIMLGYNPGGKERTEKEFEALAMGSGFKGFRVICSAFHIYAIEFLKKI

>MD07G1300900

MGSTGETQMPPTQVTDKETNLFAMQLANAPVLPMVLKTALELDLLEIMVKAGPGAFVSSADLSSQLPTKNPDAPVMLDRMLRLLASYSILTYSLRTLPDGNVERLYGLGPVCKFLTKNEDGVSIAPLCLMSQDKVLMESWYHLKDAVLEGGIPFNKAYGMSAFEYHGTDPRFNKVFNRGMAAQSTITMKKLLETYKGFEGLTSVVDVGGGTGAVVNMIVSKYPSIKGINFDLPHVIEDAPQFLGVEHVGGDMFASVPKGDAIFMKSICHDWSDDHCLKFLKNCYTALPDNGKVILAECILPVAPDSSLATQVVVNLDVIMLGYNPGGKERTEKEFEALAKGSGFKGFRVICSAFHIYAIEFLKKI

>MD09G1089200

MEMQEATEVEASLRGQADIWKYMLGFADSMALKCAVELRIADIIHSHSPTDGASNSKPMITLSQVASSIAPSPDMTYLTRIMRLLVRRNIFAIHHPSDGGEPLYGLTHSSRWLLHNAELSLAPMLMMENHPCLMAPWHYFGQCVKEGGPYAFKMAHGLEIWDYASQNPEFNKLFNDGMACTTRVVMKAILTGYEHGFDGVGSLVDVGGGTGSAVAEIVKSYPNIKGFNFDLPHVVATAPVYHGVSHVSGDMFEGSIPNADAVFMKRIMHNWSDNDCIKILKNCRKAIPEKSGKIIIVDIVLEPNGEGILDDTRLVFDLLMIAHASGGRERTETEWKNILEEGGFPRYKVIKIPALASIVEAYPM

>MD09G1089800

MQEEPEIEASLRGQADIWKYMFGFADSMALKCAVELRIADIIHSHRPSDGASNSNPMIALSQLASCIAPSPDITSLTRIMRLLVRRNIFAVHHPSDGGEPLYGLTHSSRWLLHDAELSLAPILMMNNHPCLMAPWHYFSRRVKEGGPCAFEMAHGLDIWDYASQNPEINKLFNDGMACTSRFELKEILTGYEHGFDGVGSLVDVGGGTGSAVAEIVKAYPNIKGFNFDLPHVVATAPVYHGVSHVGGDMFEGNIPNADAVFMKRIMHDWSDSDCIKILKNCRKAIPEKSGKIIIADIVLEPNGEGTLNDTRLVIDLVMIAHTSGRERTENEWKKILEEGGFPRHKVIKIPALVSIVEAYPM

>MD09G1090200

MEMQEATEVEASLRGQADIWKYMFGFADSMALKCAVELRIADIIHSHSPTDGASNSRPMITLSQLASSIAPSPDMTYLTRIMRLLVRRNIFAIHHSSDGEEPLYGLTHSSRWLLHNTELSLAPMLVMENHPCLMAPWHYFSQCVKEGGPYAFKMAHGLEIWDYASQNPEFNKLFNDGMACTARVVMKAILTGYEHGFDGVGSLVDVGGGTGSAVAEIVKSYPNIKGFNFDLPHVVATAPVYHGVSHVGGDMFEGSIPNADAVFMKWIMHDWSDSDCIKILKNCRKAIPEKSGKIIIVDIVLEPNGEGILDDTRLVFDLLMIAHTSGGRERTETEWKNILEEGGFPRYKVIKIPALASIVEAYPM

>MD10G1030000

MALQLEEEENFGCAMQLVFSSVLSMSMKSAIDLGVFDIIAKAGPGAKLSSSEIAAHIGSGTRNSEAPMMLDRILRLLASYSILSCSVVANEEDGSDSQRVYSIGPVSNYFVTDEDGISLGPMMALSQDKVVLDSWSQLKDAVVEGGIPFHRVHGKQSYEYLGLDSRFSQVFNTAMFNLTTIVTKKILHVYEGFEKITQLVDVGGGLGVTISLITSKHPHIKGINYDLPHVIKDATSYPGVEHVGGDMFTSVPSGDAILLKWVLHNWSDQHCLKLLENCYNAIPDDGKVIIVEALLPVMPETSTAVKSTSLLDVLMLSQKRGGKERSREEFMTLATGAGFSGIKYECFVASLCVMEIFK

>MD10G1030200

MLSLPNDMSQLKDAVVEGGIPFNRVHGKHSFEYLGLDPRFNQVFNTTIFNHTNIVTKKILHIYKGFEKITLLVDVGGGLGVTIRVEHVGGDMFASVPSGDAIFKKLILHDWRDQHCLKLLENCYNAIPDDGKVIIVDALIPVMPETSTAVKSTSQIDVLMMTQNQGGKEQSREEFMALATGARFSGIKYECFVCNFWVMEIFK

>MD10G1257600

MEENQRSIGNIVSARSLMNFIPLQMTLEAAIELNVFSIIAKSGPAGSHLTAKEIASQIPTSNPNSARNLDRILRMLAVHSLLSTSLKPSADDEALQDRAYGLTKETLCLVPDENGVSLAPLISFNCELDIVKSLSMLKDSVLEPEIVPFYKAHGITIFEYMSTKAELSQLFNKSMAESSNLNFDEVLKVYKGFEEVKELMDVGGGIGKTIWKVVSKYPHIHGINFDLPNVVAQAPPYQGVNHIGGNMFETIPNAQSIMLKWVLHNWEDDRCKKLLKSCFGALPKNGKVIVVEFTIPEVLENTKAVLNIVTLDISMMALPGGRERTTAEFDNLAKSVGFVETKIFPIAQEICVMEFLKREEA

>MD12G1103500

MEGNNQVSNVTAGPSSSNNEARLAILELANMISVPMSLNAVVRLNVPDAIWQGGCNTPLSASDVLARVIPNGGGDAENLQRILRMLTSYGVFEEHLSNLDDGSDDRKFSLTPIGQTLVTDQNGLSYGPYVLQHHQDVLMGAWPMVHEAVVDSTVEPFVKANGELAYEYYGKKPEMNGLMQKAMSGVSVPFMKAILDGYDGFEGVERLVDVGGSAGDCLRMILQKHPNVREGINFDLPEVVAKAPTIAGVSHVGGDMFKSIPRGDAIFLKWILSTWTDSECKVILENCYKALPEGGKLIACEPVLPKKSDESHRTRALLENDIFVMTIYRAKGKNRSEDELRQLGLSAGFSHFKPFYIDYFYTVIEFQK

>MD14G1140100

MNVVDYIGRDDRLGGLFTDSMKEFNLIFKKEILEIYTGFEGLHTLVDVGGGDGTILNMIISKHPAIKGINYDLPSVVEKSPSHPGIEHIAGDMFVRIPKGDAIFMKWILHGWDDDRCLMILKNCYEALPDNGKVIVVDMVIQEAPGTSFSAKSLFLFDVYLMNTNVMGKERTERELQSMAKAAGFSDI

>MD14G1140900

MSTINREKEVDSQYAMQLANVSALPMVLKAATGLGVLDIIHRAGAGALFLPSQIASQLLTLHNLDATSVLDGMLRLLSAYSVLTCSTIQANGKVIRVYGLAPVSKYHLKDAVLEGGLPFSKAYGMNVVDYIGRDDRLGGVFKDSMKVFNLIFKKEILEIYTGFEGLRTLVDVGGGDGTILNRIIPKHPAIKGINYDLPLVVKKSPFYPAHCRRYVYKDSALLDNGKVIVVDMVIQEAPKTSLSAKSLSLFDVYRMNTNVSGNERIERELQSLAKAARFSEIRVACISFTFSVVKLLKNM

>MD15G1409200

MALQLEEEENFGCAMQLVFSSVLSMSMQSAIELGVLDIIAKAGPGANLSSSEIAAHIGSGTRNSEAPMMLDRILRLLASYSILSCSVVANEEDGSDSQRLYSLGPVSNYFVTNEDGISFGPMMALIQDKVVLDSWSQLKDAVVEGGIPFNRVYGKGCFEYLGLDPRFNQVFNTAMFNHTTIVTNKILHLYKGFEKITQLVDVGGGLGVTISLITSKHPHIKGINYDLPHVIKHAASYPGVEHVGGDMFASVPSGDAILLKWILHDWSDQHCLKLLENCYNAIPDYGKVIIVEALLPVMPETSTAVKSTSLLDVLMLSQNRGGKERSREEFMTLATGAGFSGIKYECFFSSFCVMEIFK

>MD15G1409700

MALQLEEEENFGCAMQLVFSSVLSMSMQSAIELGVFDIIVKAGPGANLSSSEIAAHIGSGTRNSEAPMMLDRILRLLASYSILSCSVVANEDGSDSQRLYSLGPVSNYFVTNEDGVSFGPMMALMQDKVVLDSWSQLKDAVVEGGIPFNRVYGKGCFEYLGLDPRFNQVFNTAMFNHTTIVTNKILHLYKGFEKITQLVDVGGGLGVTISLITSKHPHIKGINYDLPHVIKHAASYPGVEHVGGDMFASVPSGDAILLKWILHNWSDQHCLKLLENCYNAIPDDGKVIIVEALLPVMPETSTAVKSTSLLDVLMLSQNRGGKERSREEFMTLATGAGFSGIKYECFFSSFCVMEMFK

>Mapoly0136s0036|Mapoly0136s0036.1

MELDSMTKGKLSNKEMPDIRTIYKAFDFATSFALKSTMTMGIPDILARSKEPMTADNIVEKLPCKNPAAAAGYLDRIMDILIVPGFYSKTPVSNADGKSHKAAYGLTPVSRMLVKDDVQFTLLPIALLHVHRVFTDSFQHLHKSVLEDRAPMEVALGKPFYEYLAENQDVRSTFQEALSCHSNYWIKIIAQEYDGLEHTKTLVDVGGCEGESLKELVAVHPHIHGINFDMPVVIQNAPQIPGVDHVGGDYFASVPAGDTIFLKMVLHNNGDEECLKILKNCYQALPERGGKVIIVESVYDHDSKEDEAQFVKYLDVMMLSVFLNGRERSFDAYKALLTSCGFGDCKLIKLSGGVTLIEAYKY*

>Mapoly0337s0001|Mapoly0337s0001.1

MSPQQNFGDDEAGMLQALQMSVMVTLPFTLKAAINLGVPKILVDAGPGAELTAEEIATSIAKVSNCCADPKNLDRILRILASHNVVTENVSKDANDSAQRSYGPTSTLKYFTDNEDGVSLAPLLLVTSEPVLLASYQNLHLPVMDVNVEPHGLDHGMNFFEYVATDAKLEKLFNKAMNDHSNIKMSALLKNYRGFENLTSLVDVGGGLGSTLAIILSKYPNLRGINFDQPQVVANGLQVPNLEHVGGDFFASVPEADAVFMKWILHDWDDERCVKILKNVWKALPPHGKVINLDALLSDNSDPSPATKISLYIDMIMMACSPSGKERTLSQFKKLAADAGFKRVELVAKTSHMSLLEFYKN*

>Mapoly0129s0006|Mapoly0129s0006.1

MSLQQNIRDDEAGLLQALQLSVMITLPFSLKAAMNLGVPKILVDAGPGAALTVEEIATSIDKVSDCCADRKNLDRILRVLASHNVVTEIASKDANDSDSGQRSYGPTSTLKYFTDNEDGVSLAPLLFLHCEPVYLASFQNLHLPVLDVNVEPHVLLHGMNFFEFAATDASIEKLFNKAMHDHTNIKISALLEKYRGFETLTSIVDVGGGLGATLAMILSKYPTLRGINFDLPHVVANGLQVPNLEHVGGDFFVSVPEAGAAFMKWILHDWDDERCVKILKNIWRALPPHGKLINLDSLLPDITDPSPATKISLYTDMIMMAMCPSGRERTLSQFKKLAADAGFKRVELVGQTCNMSLLEFYKN*

>Mapoly0129s0005|Mapoly0129s0005.1

MSPHQNLGDDEAGRVKALQLTAMLALPFSLKAAVNLGVPKILVDAGSGAELTAEDIAKSITKLSDRSADARNLDRILRVLAAHNIVTEIVSKNANDSDSPQRCYGPTSTLKYFTDNEDGVSLAPFLLMTTDPVFLPTFQYLHLPVLDVKVEPHVLVHGMKSFEYAATDARFDKLFNKAMHDHTHLELSALLKKYRGFETLTSVVDVGGGLGATLAMILSKYPNLRGINFDQPHVVADGLQMPNLEHVGGDFFASVPEADAVFMKWILHDWDDERCVKILKNVWRALPPHGKVINLDYVLSDNTDPSIATKVSLYTDIYMMACNSNGRERTLAQFKRLAADAGFKRVEFVAKTDNLSVLEFYKD*

>Mapoly0046s0004|Mapoly0046s0004.1

MAAQTGLGKEPGKVQIYPDKTREISNYWRDKYEKDAKKYWDIFYKRNENRFFKDRHYLDKEWGKYFNSMDIDAGSAIADSGSVSVQHRRTVLEVGCGTGNTVFPLTAQYPDLFVYACDFSPRAVDLVKAHEDFTESRVKAFVCDATSDSLVETISPASVDVVTLVFMLSAVGPDKMPAVVRNVKSVLKPGGHILLRDYAVGDLAQERLMTKDQMISENFYVRGDGTRAYYFSEEGLKKLFADEGLECKEMVVHNRQLENRARKIKMDRRWIQSVFYLPEERMPTAGIESQVARVSAPQKSPTISESGHVRSSAESASACPENGTSHEATEALVSKDPHEEELIVDLSEGAAADLFGGPPSPEPFEVVVGKHTVHALCLNAENQHTFAATGFMLWESALALATLLAVNPSFLRGKTILEVGCGSIGLCSLIASLAAEKVFATDGDSGTMDLLRENLHLNEENFPVQKIAPHKLYWGHADETAALKASNGNRGFDLIIGSDVTYVEAAVPLLFETARSLLRETSPGAQQPQFLLCHRIRGVSESEILSAAAAHGFRVEGLWTSDGYVAGEVDRRRRSNIELLFKEGLEDIATKHNSLRLLLLTPSS*

>Mapoly0003s0058|Mapoly0003s0058.1

MASAVVAFHGQQLAGLKANRTARVESMRSAALSCAAAPVGLPSLKSGVRMSSEWMSGGAALNLLSTSRGAALRMSDKRRVGGKTLGVRCSASGSSTARPASQPRFIQHKQEAFWFYRFLSIVYDHVINPGHWTEDMREDALEPADLNDPNLVVVDVGGGTGFTTLGIVKTVDAKNVTILDQSPHQLAKAKEKEPLKECTIVEGDAEDLPFATDYADRYVSAGSIEYWPDPQRGIKEAYRVIKPGGKACLIGPVHPTHWLSRFFADVWMLFPTEEEYMDWFTKAGFSDVKIKRIGPKWYRGVRRHGLIMGCSVTGVKTEAGDSPLQMGPKAEDTRAPSNPFVFAYRFLIGTIASMYYVLVPIYMWLKDLVTPKGQPI*

>Mapoly0055s0090|Mapoly0055s0090.1

MSPVLKMSPQHNLGDDEAGRLKALQIAMMLALPFSLKAAVNLGVPKILVDAGSGAELTAEDIATSITKLSDRCADATNLDRILRVLASHNVVTEIVSKNGNDSDCSHRSYGPTSTLKYFTDNEDGVSLAPFLLMATDPVTLPAFQYLHLPVLDVNVEPHVLVHGMKLFEYAATDARFSKLFNKAMHDHTHIEMSALLKKYRGFETLTSLVDVGGGLGATLAMILPKYPKIRGINFDQPHVVADGLQVPNLEHVGGDFFASVPEADAVFMKWILHDWDDERCGKILKNVWRALPPHGKLINLDYVLSDTSDPSPATKISLCTDISMMAVNSNGRERTLAQFKTLAADAGFKRVECVAQTDNLSLLEFYKN*

>Mapoly0055s0089|Mapoly0055s0089.1

MATDPVILPTFQYLHLPVLDVNVEPHVLVHGMKSFEYAATDARFDKLFNKAMHDHTHIEMSALLKKYRSFETLTSLVDVGGGLGATLAMILSKYPNLRGINFDQPHVVADGLQVPNLEHVGRDFFASVPEADAVFMKWILHDWDDEKCVKILKIVWRALPPHGKVINLDYVLSDNSDPSPATKISLCTDISMMAVNSNGRERTLAQFKMLAADAGFKRIECVAQIDDLSLLEFYKN*

>Mapoly0013s0033|Mapoly0013s0033.1

MSPQQNIGEDEAGMLQALQMSVMVTLPFSLKAAINLGVPKILVDAGPGAELTAEEIATSIAKVSNCCADPKNLDRILRILASHNVVTEIVSKDAKDSDSAQRSYGPTSTLKYFTDNEDGVSLAPFLSVAIDPVFLASYQNLHLPVMDVNVEPHTLDHGMNLFEYIATDAKLDKLFNKAMHDHSNIKMSALLKNYRGFETLTSLVDVGGGLGATLAMILPKYPNLRGINFDQPHVVANGLQVPNLEHVGGDFFASVPEADAVFMKWILHDWDDERCVKILKNVWRALPPHGKVINLDALLSDNSDPSPATKISLYMDMILMACTPSGRERTLSQFKRLAADAGFKRVELVAKTCHMSLLEFYKN*

>Mapoly0044s0122|Mapoly0044s0122.1

MSSVWQKEDSSWADPSLLVGLPMAVRAALMLNVPKIIDSAYPKLSLSAEEICKHVPTTREHTPKSENLEKILDWLCCNGIFSLIIEEGDDGSKAKRYAHSPKSRCLSRDHTADMLLMYTSPELTRVWPHAHEIVLSPDVSPFEIVTGKNMYTYLAVDAPQVQKLYVRGLNATAIPSLSLLEKYDETFKALKGRVVDVGGQEGTMCAGLVAKYPHLQFVNFDQPEVVRGAPQIPGVEHVGGSFKESVPEGNLLIMKFCLLNWDDETCIGILTNCRKALPVAEGGKMLILERLDRSTEATDAHNEVARTGSLWSNFHGTVLLSGARTRTVEQFRRLALAAGFSQLEFVNHCPGFDLLEASLSPSGHSQ*

>Mapoly0044s0123|Mapoly0044s0123.1

MSSVSQKEDASWADPSLLVGLPMAVRAALMLNVPKIIDSAHPKLSLSAEEICKHVPTTREQIPKAENLEKILDWLCCNGIFSLIIEEGDDGSKVKRYAHSPKSRCLSRDHTADMLLMYTSPELTRVWPHAHEIVLSPDVSPFELVTGKNIYTYFAVDAPQVQELYVRGLNGTAGPSLSLLEMYDETFKALKGRVVDVGGQEGTICAGLVAKYPHLQFVNFDLLEVVRGAPQIPGVEHVGGSFKESVPDGNLLIMKFCLLNWDDETCIGILTNCRKALPVAEGGKMLILERLDRSTEATDAHNEVARTGSLWSNFQSTVMLSGARTRTVEQLRSLALAAGFSQLEFVNHCPGFDLLEASLSPSGHSQ*

>Mapoly0044s0121|Mapoly0044s0121.1

MSSVSQKEDASWADPSLLVGLPMAVRAALMLNVPKIIDSAHPKLSLSAEEICKHVSTTREHTPKSENLEKILDWLCCNGIFSLIIEEGDDGSKVKRYAHSPKSRCLSRDQTADMLLLYTCPELTRVWPHAHEIVLSPDVSPFELVTGKNIYTYLAVDAPHVQELFVRGLNRTAGPALGLLEKYDETFKALKGRVVDVGGQEGTMCAGLVAKYPHLQFVNFDLPEVVKGAPQIPGVEHVGGSFKESVPDGNLLIMKFCLPNWDDETCIGILTNCRKALPVAEGGKMLIMEPLDRSTEATDAHNEVARRGSLCTNFQSTVMFSGARTRTVEQLRSLALAAGFSQLEFVNNFTGFDLLEASLSPSGHSQ*

>Mapoly0681s0001|Mapoly0681s0001.1

MELDSMTKGKLSNKEMPEIRTIYKAFDFATSFALKSTMTMGIPDVLARSKEPMTADNIVEKLPCKNPAAAAGYLDRIMDILIVPGFYSKTPVSNADGKSHKAAYGLTPVSRMLVKDDVEFTLLPIALLHVHRVFTDSFQHLHKSVLEDRAPMEVALGKPFYEYFAENQELRSTFQEALSCHSNYWIKIIAQEYDGFDHTKTLVDVGGCDGESLKELVAVHPHIHGINFDLPAVIQNAPQIPGVDHVGGDYFASVPAGDTIFLKLVLHNNGDEECLKILKNCYQALPERGGKVVIGESVYDHDSKEDEAQFVKHLDVMMLSVFLNGRERSFDAYKALLTSCGFGDCKLIKLSGSVALIEAYKY*

>Mapoly1374s0001|Mapoly1374s0001.1

MIEPTPQSLIDLDWVATSDLNHIKVIASSQNPKENYSWPDYKRSPLILGIPDILPMSKEPMTAENIVKKLPCKNPAAAAGYMKRIMDILIIPSKPLDSTEDENLMKLAPVSRLLVKDDHRFTALPLALMHLYLFFIVGLVASRICIRLCWKIDRRCHELPLKVLKKVIAHEYNGFKITKTLVDVGGDEGEAIRELVAAHPHIHGINFDLPDVIKNAPPIPGVDHVGSNFFQSIPSEKPSFECWRFPHSSPIC*

>Mapoly0015s0026|Mapoly0015s0026.1

MSPQQNFGDDEAGMLQALQMSVMVTLPFTLKAAINLGVPKILMDAGPGAELTAEEIATSIAKVSDCRADSKNLDRILRILASHNVVTEIVSKDANDSDSAQRSCGPTSTLKYFTDNEDGVSLVPFVFMHIDPVFLASYQNLHLPVLDVNVEPHTLDHGMNLFEYIATDAKLDKLFNKAMHDHSNIKMSALLKTYRGFETLTSLVDVGGGLGSTLAMILSKYPNLRGINFDQPHVVANGLQVPNLEHVGGDFFASVPEADAVFMKWILHDWDDERCVKILKNVWRALPPHGKVINLDALMSDISDPSPATKISLYMDMIMMACTPSGRERTLSQFKKLAADAGFKRVELVAKTSHMSILEFYKN*

>Mapoly0015s0025|Mapoly0015s0025.1

MSPQQNLGDDEAGRLKALQIAMMLALPFSLKAAVNLGVPKILVDAGSGADLTAEDIATSIAKLLDRCADATNLDRILRVLASHNVLTEIVSKNGNDSDCPQRSYGPTSTLKYFTDNEDGVSLAPFLLMATDTVILPTFQYLHLPVLDYAATDARFDKLFNKAMHDHTHIEMSALLKKYRGFETLTSLVDVGGGLGATLAMILSKYPKIRGINFDQPHVVADGLQVPNLELVGGDFFASVPEADAVFMKWILHDWDDEQCVKILKNVWRALPPHGKVINLDYVLSDNSDPSPATKISLCTDISMMAVNSNGRERTLAQFKRLAADAGFRRIECVAQIDDLSLLEFYKN*

>Mapoly0015s0023|Mapoly0015s0023.1

MSQQQNLGDDEAERLKALELVMMLALPFSLKAAVNLGVPKILLDAGSGAELTAEDIATKLSDRCPDATNLDRILRVLASNNIVTEIVSKNGNDSDCPQRSYGSTSTLKYFTDNEDGVSIAPFLLLCTDPVFVPTFQYLHMPVLDENVEPHVLVHGMKSFEYAATDTRFDKLFNKAMHDHTHIEISALLKKYRGFETLTSLVDVGGGLGATLAMILSKYPKIRGINFDQPHVVAEGLQVPNLEHVGGDFFASVPEADAVFMKWILHDWDDERCVKILKNVWRALPPHGKVINLDYVLSDNSDPSPVTKISLCMDISMMAINSNGRERTLSQFKRLAADAGFKRVELVAQTDNLSLLEFYKN*

>Mapoly0002s0063|Mapoly0002s0063.1

MTLQQNIGDDEAGRLQALQLAEIVALPLSLKAAVTLGVPKILVDTGPGAGLTVEEIAASISKLSDRCADPANLDRILRVLASHNVVTEIVSKIPNDSDSPQRRYGPTSTLKYFTDNEDGVSLAPLLLLLTDPIFLAPCQYLHLPVLDVKLEPHVVVHGMNCFDYCPTDARFQSVFDKAMHDHSHIEMTALLEKYRGFETLTSLVDVGGGLGASLAMILSKYPNLRGINFDQPHVVTDGLKVPNLEHVGGDFFASVPEADAAFMKWILHDWDDERCVKILQNIWRALPPHGKLLNLDSLLSDVSDPSPATKTSLYLDMFMLAVSPRGRERTLSQFKKLASDAGFKRVEVVAQVDNLSLLEFWKN*

>Mapoly0002s0334|Mapoly0002s0334.1

MNCFEYCPTDARFQTVFDKAMHDHSHILMSALLEKYRGFETLTSLVDVGGGLGASLAMILSKYPNLRGINFDQPHVVADGLKVPNLEHVGGDFFASVPEADAAFMKWILHDWDDERCVKILENIWRALPPHGKLLNLDSLLSDISDPSPATKISLYADMIMLAVTPRGRERTLSQFKKLAAGAGFQKVEVVAQVDNLSLLEFCKN*

>Mapoly0015s0024|Mapoly0015s0024.1

MSPQQNFGDDEAGMLQALQMSVMVTLPFTLKAAINLGVPKILVDAGPGAELTAEEIATSIAKVSNCCADPKNLDRILRVLASNNIVTEIVSKNGNDSDCTQRSYGPTSTLKYFTDNEDGVSLAPFLLLSTDPVFVPTFQNLHLPVLDVNAEPHTLDHGMNLFEYIATDAKLDKLFNKAMHDHSNIKISALLKNYRGFETLTSLVDVGGGLGATLAMILPKYPNLRGINFDQPHVVADGLQVPSTVVSPFLAHYILQTYSMHCYLQLYFHRNCGNTLFCFFAEFKQF*

>Mapoly0040s0105|Mapoly0040s0105.1

MTAEECSGDEREGRLAALQLAGMCAVPLSLKAAILLGVPEILNEAGPDAKLTSEEISKLIVSPGGTSADAENLDRLLRVLACHNIVTETCVAACPSNPQATERRYGVTPVLKYLIASNETGMSLSPLFLLRTDSVYTSAFQYLHAPVLDRNAEPFVIANGKKIIDVLETDSRLSELFDTSMAHHTHMWVSMLLETYRGFEGLTSLVDVGGGVGANLAMILSRYPDLRGINFDLPHVVAKGIQSPRLEHVAGDFFQSVPRGDAVWMKWILHCWSDESCVKILKSVNRALPPRGKLINMDSVLPETSDSSVETQINVCADMLMMAANKGGKQRTLTQFRKLAQDAGFSSVHLVATVDTLSVLEFHKG*

>Mapoly0002s0065|Mapoly0002s0065.1

MTLPENIGDDAAGRLQALQLTDIVALPFCVKAAVLLGVPKILVDAGPGTGLTVEEIAAAISKLSNCCADPANLDRILRVLASFNVVTETLSKNVNDSDSPQRRYGSTSTLKYFTDNKDGVSMAPLLLMVTDPIFLAPYQCLHLPVLDAKSEPHVVVHGKNCFDYYATDARFQTVFDKAMHDHSHIAMSALLEKYRGFETLTSLVDVGGGLGASLAMILSKYPNLRGINFDQPHVVAEGLKVPNLEHVGGSFFESVPEADAVFMKWILHDWDDERCLKILENIYRALPPHGKLLNLDALLSDVSDPSPATKVSLCADIAMLACTPKGRERTLSEFKQLAADAGFKRVEVVAQVDHLSLLECYKN*

>Sobic.001G354200.1 (SbASMT1)

MALVQESSQDLLQSYVELWHQSLCFAKSMALAVALDLRIPDAIHRHGDDGATLAQILAETALHPNKLRALRSLMRALTVFGTFSVQQPPTSVVVDASSGEAAVYRLTAASRFLVGDDEVSSSSTTLAPFVSLALHPIAVAPHTQGMCKWFRQEQNEPTAYALAFRQPTPTIWEHADDVNALLNKGMVADSRFLMPVVIRECGEVFRGIDSLVDVGGGHGGATATIAAAFPHIKCSVLDLAHVVAGAPSDVNVQFIAGNMFQSIPPATAVFLKTTLHDWSDDNCVKILKKCKQAISPRDAGGKVIILDMVVGYGEPNIKLLKTQVMFDLYIMTVNGAERDEQEWKKIFIEAGFKDYKILPVVGALSVIEVYP

>Sobic.001G354400.1 (SbASMT2)

MAPAEESSQDLLQAYVELWHQSLCFAKSMALAVALDLRIPDAIHRHGGGGATLTQILAETKLHPNKLRALRSLMRALTVLGTFSVQQPPPTIADASGEAVYRLTAASRFLVSDEVSSSTLAPFASLALHPIAVAPSTVGMCKWFRQEQNEPSAYALAFCQPTPTIWEHADDVNALLNKGMVADSRFLMPIMLRECGEVFRGIDSLVDVAGGHGGATATIAAAFPHIKCSVLDLPHVVAGAPSDVNVQFVAGNMFESIPPATAVFLKTTLHDWSDDNCVKILKKCRQAISPRIAGGKVIILDMVVGYGELNTKHLETQVMFDLYIMTVNGAERDEQEWKKIFTEAGFKDYKILPVLGALSVIEVYP

>Sobic.002G077700.1 (SbASMT3)

MAQSKSVPVPPTSTTTTTGAELLQQAEAELWCHNFGYLKSMALRCAIKLGIPNAIHRRGGAASLSHCRSCMPPSPSPKRPCLSRIITFLAATGIFREEEISADDGEAAAGPRYHLTAASRLLIVVDDDDDARGGGRGCVAQLFILCSSPSYFTASQHLPEWLQQEDGTTDDDGAPAAARTTPFTMAHGAGFYDVVRRDAAFGVVFDEAMGSDSRFVADIVVRECGEVFAGLTSLVDVGGNNGTTARAIAKAFPHVRCSVLELPRVVDAMPADATVEFVAGDMMKFIPPADAVLLKFTLHNWSDEDCVQILKRSKEAISTREPKGKVIIIDIVSGSPSNKRTLEAQLLMDLCMMVVLEGKERTEETWHKIFLDAGFTRYKVTPILGTTRSLIEVYP

>Sobic.002G079300.1 (SbASMT4)

MDVGGGDGTMAAAIAKAFPQIRCSVLELPHVVDAAPADCGVQFIAGDMMEFIPPADVLLLKWILHNWSDEDCVRILKRCKEVVSTREPKGKVVIIEVVVGSQSKQMLEAQFVSDLCMMLLTTGEERDRDKWQRIFQDAGFTQYKISPVLGFRSLIELYP

>Sobic.003G298500.1 (SbASMT5)

MDTSRDRGLTTGDELMQAQAELWNHVFAYTRSMSLRCAVELGIPDAVNRLGGAASVPELVAALSLPLPRAPYLRRLMRLLAHAGFFVFFFEIGDIAGFFVFDAAAATYGLTPLSRLLVSTATPGAGGGQGGLSPFALAMLHPVIVSPSMSLASWFRAADAANAAARVPFESAHGRDLWAVAKDDREFRAAFHDAMACDGRFVMDVLVRGHSDVFRGLASLVDVGGGSGGAARAIANAFPHIRCSVLELPHVVAAVPTGELGGVEFVAGDMFEHVPKADAVLLKWILHGWDDENCVRLLRRCREAIPSREDGGRVIVMDLVVGSSPADEKATETQLLWDVMMMGVVGSPERDEREWRRIFQDAGFSGYKIVALLGIRSVIEVYP

>Sobic.004G128400.1 (SbASMT6)

MALSKEQSTGSDEQAVLDAQLQLWHHTVGYVKSMALKAALDLNIPNAIHLHGGSATLPQIVTKVTLHPSKIPCLRRLMRVLTFTGVFSVHDGDGGDEPVYGLTPASRLLIGSGMNLTPLLTLMLGAVFVSSFLDIGEWFRHEMPGPSPFEMANGRDIWDLCNHDAGFGKLFDDGMVADSDFIMDVVVKECSDVFQGISSLVDVAGGHGGAAQTIAKAFPHVECSVLDLSHVIASAPTNTNVKYIAGDMFESIPSANAVFLKWIMHDWGDAECVKILKNCKKTIASQGGGKVIILDMVVGTGSSSDEKHVETQILFDLLVMCIYTKGAERDELEWKKIIFEAGFSHYKIIPVLGTRSIIEVYP

>Sobic.004G341500.1 (SbASMT7)

MAFLGEYSSQELLQGQLLLWHQSLGFFKSLALAVAMDLRIADAIHRLGGAATLPQIIAEAGIKIDPRNNKLRDLRRVMRALTVSGIFTVQRPASAVVAAAADGGAEAEGPVYKLTAASRLLVVGEKKSSTTTTMPTPSLTVQVQLFLETCRGSAFSRGMRAWFRPQDEQHQHQEQPAGLSPFAMACGGQTIWERAERDADAFPFDDAMASDTAFLMPIVLRECGDEVFRGLTSLVDVAGGLGGAAANIAAAFPDLKCTVLDLPQVVAKAAAGSNEKVQYVGGDMFESIPPADAVLLKWILHDWSDDECVKILKKCKQAIPPRAAGGKVIIIDMVVGSADEPSPESDVRHVETQVLFDLLIMCINGVERDELEWKKIFSEAGFHDYRIIPVLGVRSIIELYPN

>Sobic.004G341600.1 (SbASMT8)

MAFLGEYSSQELLQGQLLLWHQSLSFFKPVALAVAKDLRIADAIHRLGGAATLPQIIAEAGINPCKLRALRRVMRVLTVSGIFTAVQQPATATAAAASGGGGTEGPVYKLTAASSLLVVGEKSSTAAAAATTKSQLPPSLSVQVQLFLDPCRGSAFSSGIRAWFRQDEHQQPAGLSPFALACDGQTIWERAERDADVFPFDDAMASDNAFLMPIVLKECGDVFRGLTSLVDVAGGLGGAASTIAAAFPDLKCTVLDLPQVIAKAPSAAGTSVQYVAGDMFQSIPPADAVFLKWILHDWNDDDCVKILKNCKQAIPPRDVGGKVIIIDMVVGSESSDNRHVETQVLFDLLVMTIDGAERDEQEWKKIFLEAGFEDYKIIPVLGVRSIIELYP

>Sobic.005G045600.1 (SbASMT9)

MGSITEQQQCTDQQGLLDAQLELWHSTFAFIKSMAFKSALQLGIADAIHCHGGTATLTQIATKAALHPSKTPCLRRLMRVLTVAGIFSIAAKTSSDDDDDDDGGDHVYGLTPASRLLVGSSQNLTPTLSLILDNVFVSPFLDLGTWFEHELPAADLPLFELSHGKNVWDVVGHDPSMSQLFNAGMVADTSFLMDIAIRECGGVVFQGISSLVDVGGGHGAAAQAISVAFPGIQCTVMDLAHVVATAPACAGLSFVAGDMFEAIPPANAVFLKWIMHDWSDTECVTILRNCKKAIPPRDAGGKVIIVDTVVGAGPPNLKNRETQVMSDIFFMIVNGTERDEQEWRKIIFEAGFSDYKIIPVLGVRSIIELYP

>Sobic.005G086600.1 (SbASMT10)

MALSSEDTRELLQAHVELWNQTYSFMKSVALAVALDLCIADAIHRCGGAATLSQILGEIGVRPCKLPALHRLMRVLTVSGTFTIVQPSAATMSLESDGNELVYKLTTASRLLVSSESSATASLSPMLNHVLSPFRDSPLSMGLTAWFRHDEDEQAPSPCPFTLMYGTTLWEVCSRDDAINALFNNAMAADSNFLMQIVLREFGKVFHGIDSLVDVGGGVGGATMAIATAFPSLKCTVLDLPHVVAKAPSSSIGNVQFVGGDMFESIPPANVVFLKWILHDWSNDECIKILKNCKQAIPSRDAGGKIIIIDVVVGSESSDTKLLETQVMYDLHLMKIGGVERDEQEWKKIFLEAGFKDYNIMPVLGLRSIIELYP

>Sobic.005G129100.1 (SbASMT11)

MAGAEDSTQDLLQAHLQLLHQSLGYISSTALAVALDHGIPDAIQHHGGSATLSQILAKIDVSPTKRRGLHRLMRMLTVTGAFSVDQRPAVAVAAAPHDGGRRGDPVYQLTAASRLLLSDDDDGIRHPASLTPLLTMLLGPLLASPLAAVVSALVREDRQPDLSAFGIAHGQTVWDDVADHEAAFNVSLHDAIAADTRFLMPIVLKECGEVFRGIDSLVDVGGGPYGSAAASIAAAFPRLKCSVLDLPHVVAQAPPDSSVQFVAGNMFECIPRANAVFLKWILHDWGDDECIKLLKRCKQAIPPRDAGGKVIIIDMVLGSGPADDVKHKETQVLFDLLMMALNGVERDEQEWKKIFLEAGFKDYKIIPVLGVRSIVELYP

>Sobic.005G216100.1 (SbASMT12)

MAFVHGEQEGEDMVGAFALVYQHVFSYIKTMALKCAVELAIPDAIHGRGGAATLAEIAADTGVHESRLADLRCLMKLLTTSGLFRATAADGGEEVASSYALTAASSLVVGPRGLSNMVRFDCGPVSLTPFFDMPTWLRTAPGPEAPKSLFELTHGRTRWDPANADNDTMIVSAFIESQLLIEAVLGGHADVFRGLTSLVDVGGGQGSFAKAIAAAFPGIKCAVMDLPHVVADAPVAAGGGDDVQFVAGDMFESIPPADAVLLKYVLHCWDDDNCVKILKNCKEAIPARDAGGKLVITEMVLGSGPRRDRNVAETEEMHSLFLTCITGVGREEHEWKKIFVDAGFSDYKITPVMGPLSVIEVYP

>Sobic.005G216200.1 (SbASMT13)

MAFVHGEQEGEDMVGAFALVYQHVFSYIKTMALKCAVELAIPDAIHGRGGAATLAEIAADTGVHESRLADLRCLMKLLTTSGLFRATAADGGEEVASSYALTAASSLVVGPRGLSNMVRFDCGPVSLTPFFDMPTWLRTAPGPEAPKSLFELTHGRTRWDPANADNDTMIVSAFIESQLLIEAVLGGHADVFRGLTSLVDVGGGQGSFAKAIAAAFPGIKCAVMDLPHVVADAPVAAGGGDDVQFVAGDMFESIPPADAVLLKYVLHCWDDDNCVKILKNCKEAIPARDAGGKLVITEMVLGSGPRRDRNVAETEEMHSLFLTCITGVGREEHEWKKIFVDAGFSDYKITPIMGPLSVIEVYP

>Sobic.006G007900.1 (SbASMT14)

MVLISEDSRELLQAHVELWNQTYSFMKSVALAVALDLHIADAIHRRGGAATLSQILGEIGVRPCKLPGLHRIMRVLTVSGTFTIVQPSAETMSSESDGREPVYKLTTASSLLVSSESSATASLSPMLNHVLSPFRDSPLSMGLTAWFRHDEDEQAPGMCPFTLMYGTTLWEVCRRDDAINALFNNAMAADSNFLMQILLKEFSEVFLGIDSLVDVAGGVGGATMAIAAAFPCLKCTVLDLPHVVAKAPSSSIGNVQFVGGDMFESIPPANVVLLKWILHDWSNDECIKILKNCKQAIPSRDAGGKIIIIDVVVGSDSSDTKLLETQVIYDLHLMKIGGVERDEQEWKKIFLEAGFKDYKIMPILGLRSIIELYP

>Sobic.006G008000.1 (SbASMT15)

MVHISEDSRELLQAHVELWNQTYSFMKSVALAVALDLHIADAIHRRGGAATLSQILGEIGVRPCKLPGLHRIMRVLTVSGTFTIIQPSAETMSSESDGREPVYKLTTASSLLVSSESSAAASLSPMLNHVLSPFRDSPLSMGLAAWFRHDEDEQAPGMCPFTLMYGTTLWEVCRRDDAINALFNNAMAADSNFLMQILLKEFSEVFHGIDSLVDVAGGVGGATMAIAAAFPCLKCTVLDLPHVVAKAPSSSIGNVQFVGGDMFESIPPANVVLLKWILHDWSNDECIKILMNCKQAIPSRDAGGKIIIIDVVVGSDSSDTKLLETQVIYDLHLMKIGGVERDEQEWKKIFLEAGFNDYKIIPILGLRSIIELYP

>Sobic.007G074800.1 (SbASMT16)

MALISEESKDLLQAQAELYNQVFSYMKSVALAVALDLRIADAIHHHGGAATIFQIAGEIGVNPCKIPRLRRLMRALTVAGIFTIQPSDEQTVKASSAGHEPAAVYKLTTASRILISKSSPSLFPTLSQQLNSFRDSVLSMGLSTWFRHDEQPGPCPFTLKQGITFWEKSERDHAANVSFNNSMAAHSRFLMQIVLKELSNIFHGMGSLVDVGGGAGGASISIAAAFPCMKKCSVLDLPHVVAKAPSAASVSNNVQFVAGDMFRSIPPANAVFLKWILHDWGDDECIKILKNCKQAIPSRDAGGKAIIIDIVVGSKPSDTKLLETQVLCDLNMMKIGGAERDEQEWKKLFLEAGFKDYNIMPVLGLWSIIEVYP

>Sobic.007G170500.1 (SbASMT17)

MTLRLLAEVSPQDLLVALSELQTHVLSYIKSMALKCAVDLSIHDTIHRHGGAATLADIAADAKIHPAKVPDLQRVMELLAATGIFTATASKKDDGSAETVYGLTTACRFLVGHRNLSPMVPFLVSPLVVSSFFSLSDWLRKEPAAAGAGGAGSLFELAHGCSHREMAKQDAAFSSVVNDSMAADSQLFLEVVIMDKGRIFRGLSSLVDVGGGHGAAAQVIARAFPRIKCMVLDLPHVVNEATASDGNMHFIAGDMFESIPPADAVLLKNILHEWGDENCVKILQRCKQAIPSRTAGGKVIIIEMVRGSSQGDSKINEMEVIRNMFMLCINGVERDINEWKKIFSDAGFSDDYKIMPVLGPFSVIEIYP

>Sobic.008G013900.1 (SbASMT18)

MHQAAPPPRRRLTCRRCSASWRALARPSPPSSAWKSGSDFRDGGATTLFEMALGVPPWTLTKNDDAYNRAMNEACVVDTTMSMDIMLKDTSSSIFSGLTSLVDVGGGHGAAAMAIATAFPHIKCSVLDLEQVIIKVPQQAGTDHIHSTVQFIAGDMFESIPPTDAVFLKHVLDCWDDDHCVKILQQCKRAIPARDAGGKVIIMNVVIGYGSLDKIVKEAQVLFDMYMMRYGGSEREEHEWRKIFSKAGFSDYKITPILGFHSIIEVFP

>Sobic.008G014000.1 (SbASMT19)

MSPPSAHAEQQEPDAHNHLLHQTYVELYHHGLHHIKSSALLCAVGLGIPGAIRGGAATISDLVTDTGVRPAKRSHLRRLMRMLTCFGIFGAASEQREGSAATDDGESETVIYTLTPVSSVLVGDKDASGAAAAASPSLDMSALLRLVARPSTSVSTFFSLEEWFRDGGGATTLFEMALGVPPWTLTKNDAAYNRAMNEGCVADTSLAMDVMLKDTCRGASSIFSGLTSLVDVGGGHGAAAMAIATAFPHIKCSVLDLEQVIIKVPTTDLIHSTVKFIAGDMFESIPPADAVFLKHVLHCWDDDHCVKILRQCKRAIPSRDAGGKVIIMSIVVGYGTLDKVVKETQVLFDMYMMRYGGSEREEHEWRKIFSKAGFSDYKITPILGFHSIIEVFP

>Sobic.009G197000.1 (SbASMT20)

MALTTSTNQALLDAQLELWNTTFSHIKSMALKSALDLRIADAIHNHAGAATVPDIVATVKLHPSKIPCFRRLMRVLAATGVLSAGNPSGSSTELVYALTPLSRLLVGSHNLVPITAMILHPSFVSPFLELGTWFQQELPGPCVFKQTHGQTVWEQAARDASFDALVNDGMVSDSHFIMDIVFEECADAFQGISSLVDVGGGLGAAAQAISKAFPDVKCSVLDLDHVVAKAPSGTDVQYIAGDMFESVPPANAMFFKWVLHDWSHEECVKILKNCKKAIPPKEEGGKVIIIDIVIGEESSNLKHKETQALFDLYIMLVNGIERDEQEWKKIFFEAGFSDYKILPVLGARSIISVYP

>Sobic.009G197400.1 (SbASMT21)

MALTTSTNNQALLDAQLELWHTTFAYMKSMALKSALDLGIADAIHSHGGNATLPQIVSRATPTLHPSKIPCLRRLMRVLTATGIFSAAHHDDAGGGGELVYGLTPASQLLVGGSSSLTPFMSLALHGIFVSPFLGLGTWFQQEHSDPSLFEMTHGQTAWDLNDHNPAFGKLFNQGMVCDSSFIMDIVVKDCGDVFRGLSSVVDVAGGLGGAAMAISIAFPDVQCSVLELPHVAANAPTSTDVKYVAGDMFESIPPANAVFLKWVLHDWGDADCIKILKNCKNAIPSRDAGGKVIILDMVMGGQSSNIKHKETQVLFDLFIMFVNGVERDEQEWKKIIFEAGFSDYKIIPVLGVRSIIEVYP

>Sobic.009G197600.4 (SbASMT22.1)

MAPVKAQDASTDQQTMLDAQLQLWHHTFGYIKSMALKAALDLRIPDAIHQHGGSATLPQIATKATLHPSKIPCLRRLMRVLTLTGVFQHSTTTDDDGGELVYELTPASRLLVGSVTTNVSPFLNMVLGTVFVSSFLDLGEWFQHELPDPSPFKLTHGRHVWDLAIHDASFAKLCDNGMVADSGFIMDVMVKECGDVFQGISGSLVDVAGGLGGATQAIAKAFPHIECSVLDLPNVVAVAPTDTDVKYIAGDMFESVPSANVVFLKWVLHDWGDAECVKILKNCKKAIPSEGGKVIIMDIVVGAGSSDQKNVETQVLFDLFIMTINGAERDEKEWKKIIFEAGFRSYKIMPVLGVRSIIEVYP

>Sobic.009G197600.5 (SbASMT22.2)

MAPVKAQDASTDQQTMLDAQLQLWHHTFGYIKSMALKAALDLRIPDAIHQHGGSATLPQIATKATLHPSKIPCLRRLMRVLTLTGVFQHSTTTDDDGGELVYELTPASRLLVGSVTTNVSPFLNMVLGTVFVSSFLDLGEWFQHELPDPSPFKLTHGRHVWDLAIHDASFAKLCDNGMVADSGFIMDVMVKECGDVFQGISGSLVDVAGGLGGATQAIAKAFPHIECSVLDLPNVVAVAPTDTDVKYIAGDMFESVPSANVVFLKWVLHDWGDAECVKILKNCKKAIPSEGGKVIIMDIVVGAGSSDQKNVETQVLFDLFIMTINGAERDEKEWKKIIFEAGFRSYKIMPVLGVRSIIEVYP

>Sobic.009G197800.1 (SbASMT23)

MLGTVFVSSFFDLGEWFGHELPDPSPFKLAHGRHIWDLADHDASFGKLFDDGMVADSSFIMDVVIKECSDVFQGISSLVDVAGGLGGATQTIAKAFPHVECSVLDLPHVVTNAPTDTNVKYIAGDMFESIPSANAVFLKWILHDWGDDECVKILKNCKKAIASQGGKVVILDMVVGAGSSDEKYVEMQILFDLFMMFINGAERDELEWKKIIFEAGFSSYKIIPVLGVRSIIEIYP

>Sobic.009G198000.1 (SbASMT24)

MALSKKQHTSTDQQAVLDAQLLLWHHTIGYVKSMALKAALDLRIPDAVHQHGGSATLPQIVTKVTLHPSKIPCLRRLMRVLTLTGVFSVVHDGAGDEPVYGLTPASRLLVSSGRLNVTPFLTLMLGTVFVSSFFDLGEWFGHELPDPSPFKLAHGRHIWDLADHDASFGKLFDDGMVADSSFIMDVVVKECSDVFQGISSLVDVAGGLGGATQTIAKAFPHVECSVLDLPHVVANAPTDTNVKYIAGDMFESIPSANAVFLKWILHDWGDAECVKILKNCKKAIASQGGKVVILDMVVGAGSSDEKHVETQILFDLFMMFINGAERDELEWKKIIFEAGFSSYKIIPVLGVRSIIEVYP

>Sobic.010G230800.1 (SbASMT25)

MALPAEYSSEELFKAQLQLWHQALCFFKPVALSVVLDLRIPDAIHRLGGAATLPQIMAEAGINLCKLRDLRRIMRVLILSGIFSVKQVATTVASDGEGPIYKLTTASSLLVSDESLTSQQQLLRPFLYPQLMLTPCRECPVSRGLHAWFRQDQPQPAGLSPFALAYSGLTIWERAERDATTTFPFDDAMASDTAFLMPIVLKDCGEVFHGLTSLVDVAGGLGGAAATIKAAFPDMKCTVLDRPEVVAKAPTDNGVQYVVGDIFQSIPPANAVFLKWILHDWNDDECVKILKNCKKAIPPRDAGGKIIIIDMVVGSEPSDIKHLETQVLHDIIMMNLNGIERDEQEWKKIFFEAGFKDYKIIQLLGVRSIIELYP

>Sobic.010G231000.2 (SbASMT26)

MHFQELVSNSTKTGLADFDLSTFVVKTCYICNHLQTPQHRTTSHQKFKIDPSICTIMALLDEYSSDELLQAQLQLWHQALGFFKSVALAVALDLGIPDAIHRLGGAATLPQILAEAGVNPRKLRNLRRIMRVLTLSGIFSIKQAATAASDGEGPVYKLTTASRLLVKDESLTRSQQLPPVLYTHLMLTPCRESTAGMGINAWFRQEQDQPQPAAGPFALEYNGQTVWERAEHDATTFPFDDAMASDTAFLMPIVLKECSEVFHGLTSLVDVAGGLGGAAATIVAAFPDLKCTVLDLPHVVAKAPTDTNVHYVAGDIFQSIPPANAVFLKWILHDWHDDECVKILKNCKQAIPPRDAGGKIIIIDMVVGSEPSDLKHIETQVLFDLMMMNVNGVERDENEWKNIFFEAGFKDYKIIPLLGVRSIIELYP

>Sobic.010G234400.1 (SbASMT27)

MALLDEYNSQELLQAQLQLWHQALGIFKPVALALALDLHIPDAIHRLGGAATLPQILLEAGINPCKLHDLCRIMRVLTFSGIFSVKQAATVTSDDDDRHDGEGPVYKLTTASRLLVRDESLTTTQQLPALMYVQLMLSPSRESPLCKGLHAWFRQDHQDQPQPAGLSPFALSYSGQTIWERAERDATAFPFDDAMASDTAFLMPIVLKECGEVFHGLTSLVDVAGGLGGAAATIAAAFPDLKCTVLDLPQVVAKAPTDTNVHYVAGDMFQSIPPANAVFLKLVLHDWNDDECVKILKNCKKAIPPRDAGGKIIIIDMVVGSEPSEPSDIKHIETQILKDLMMMNINGLERDEQEWKKISFKAGFKDYKIIPLLGVRSIIELYP

>Sobic.010G234500.1 (SbASMT28)

MALLDEYSSQELLQAQLQLWHQALGIFKPVALALALDLQIPDTIHRLGGAATLPQILLEAGINPCKLHDLRRIMRVLTFSGIFSVKQAATVASDDNDRHDGEGPVYKLTTASRLLVRDKSLTTTQQLPALMYVQLMLSPSRESPLGKGLYAWFRQDQDQPQPAGLSPFALSYSGQTIWERAERDATTFPFDDAMASDTAFLMPIVLKECGEVFHGLTSLVDVAGGLGGAATTIAAAFPDLKCTVLDLPQVVAKAPTDTNVQYIAGDMFQSIPPANAVFLKLVLHDWNDDECVKILKNCKKAIPSRDAGGKIIIIDMVVGSESSDPSDIKHIETQILKDLMMMNINGVERDEQEWKKISFEAGFKDYKIIPLLGVRSIIELYP

>VIT_203s0038g03090|VIT_203s0038g03090.1|Vvinifera_v2.1

MEKVVKIMEKEEAEAEVEMWKYIFGFVEMAVVKCGIELGIADVIESHAGPITLSSLSSSLGCSPSGLYRIMRFLVNRRIFKEVATSQGDTGYQQTPLSRRLMTRSENGMAALLLLESSPVMLAPWHGLSARLLGKGNATFDAAHGQDVWGYAASHPAHSKLINDAMACDARMAVSAIVNGCPEVFDGVSTLVDVGGGDGTALRTLIKARPLIRGINFDLPHVVSSAPKCNGVEYASGDMFDTVPKADAAFLMWVLHDWGDEECIQILEKCRQAIPGDKGKVIIVEAVIQENEKEGDNNLKDVGLMLDMVMMAHTTTGKERTLKEWDYVLKKAGFNRYTVKPIRAVKSVIEAYP*

>VIT_203s0038g03080|VIT_203s0038g03080.1|Vvinifera_v2.1

MNILTKDLNKATRDMKTLEEEEEAEVGIEMWKYIYGFAEMAAVKCGIELGIADVMESHGGPITLSALSSSLGCPPSGLNRIMRFLVSRRIFKEVATSQGDTGYQQTPMSRCLMTSGGDGMAAFVLLESSPVMLAPWHGLSARVLGKGNSTFEAAHGDDIWGYAAKNPDHSKLINDAMASNARRVVPAMVNDCPEVFDGVSTLVDVGGGNGTALRTLVKACPWIRGINFDLPHVVATAPKCNDVEHVGGNMFDSVPKADAVFIMKVLHDWGDEKCIKILEKCREAIPEDKGKVVIVEAVIEDGDPQGDDQKLKDVKLMVDMVMMAHTTTGKERTFKEWDYVLLNAGFSRYTIKPIRNIVQSIIEAYP*

>VIT_215s0045g01490|VIT_215s0045g01490.1|Vvinifera_v2.1

MDLGSAERADELLQAQAQVVNHVFNFMNSMALKCAIDLAIPDVIHSHGQPMLLSQLVTALSVQPSKAPCLHRLMRLLVHSGFFSQQQVIHNNEQEEGYSLTSASRFLLKDEPLTGLPLSLLHLNPVLTAPWHFLSGWFRNGDPTPFYTAHGKPYWDYTAQEPDFNDLFNEAMASDSRIIASVLITKCKEQFKGLFSLVDVGAGTGTMTKAIAKAFPHLKCIVFDQPHVVADLQGGGNLEVVGGDMFETIPSANAVILKWILHNWSDEECVKILKKCKEAIPTKDKGGKLMIIDMVMENNKGDDQAVETQLFWDMLMMTVLTGKQRNENEWKKLFVTAGFTHYKISAVLGFRSLIEVYP*

>VIT_212s0028g02860|VIT_212s0028g02860.1|Vvinifera_v2.1

MDVGSGKRASLLLQAQAHVWNHTMNFVNSMALKCAVRLGIPDAINNHGQPITLSELVTALSINPIKAPCLHRLMRVLVHSGFFAQQQADHNEQEQLYSLTYASRFLLKDEPTSGAPLLLVQVDPHLTNPCHFLSDWFRNSDPTPFVTAYGKPFWDYAAHEPKFNNFFNEAMASDSQLIASVVVGECKEVFRGLSSLIDVGGGTGTMAKVIAKAFPHLKCTVFDQPHVVANLQGGENLEFVGGDIFEAIPPADAILLKSILHNWSDGECVKILKKCKEAIHPRKDKGGKVIIIDIVMENNKGDEAVEAQLFYDILMMVVVAGKERNEREWENLFLAAGFAHYKITSTLGPRSLIEVYP*

>VIT_212s0028g02870|VIT_212s0028g02870.1|Vvinifera_v2.1

MECLGSRERATELLQAQVHTWKHTTNFVNSMVLKCAIQLGIPDVIHSHGQPMALSQLITALSLEPTKAPCLYRLMRVLVHSGFFAQQKLLHNSEEEEGYSLTFASQFLLKDEPVSGVPFLLLQLDPILTAPWHFLGDWFQNEDPTPFHTAHRKSFWDYAVHEPKLNDIFNETMVSDSRLIANMIVRQYKEVFEGLASLVDVGGGTGTMVKAIAKAFPQLKCIVFDQPHVVANLEVGENLEIVGGNIFEAIPPADAILLKWILHDWSDEECVKILKKCKGAIPNKGGKVIIIDMVVESNKGDNKAVETQLFIDMLMMVVVAGKERNEKEWEKLFLAAGFTHYKITPALGLRSLIEVYP*

>VIT_212s0028g02920|VIT_212s0028g02920.1|Vvinifera_v2.1

MDPANGEISSELLQAQAHVWNHIFNFVNSMSLKCAIQLGIPDIIHNHGKPMTLPELVAKLPVHPKKTQYVYRLMRVLVQSDFFAAQRAQQSEEEEGYVLTHASRLLLKDDSLSVRPFLLAMLDPTLTKPWHYVSAWFQNDDPTPFDTAHERTLWDYAGHEPQLNYFFNEAMASDARLVTSVLVKEGKGVFEGLNSLVDVGGGTGTVAKAIANAFPHLNCTVLDLPHVVAGLQGSKNLNYFAGDMFEAIPPADAILLKWILHDWSDEECVKILKRCREAIPSKERGGKVIIIDMMVKNQKGDCKSRETQLFFDMLMMVLVTGKEREEKEWEKLFLEAGFSHYKITPILGLRSLIEVYP*

>VIT_212s0028g02810|VIT_212s0028g02810.1|Vvinifera_v2.1

MDLANGEISAELLHAQAHVWNHIFNFINSMSLKCAIQLGIPDIIHNHGKPMTFPELVAKLPVHPKRSQCVYRLMRILVHSGFLAAQRVQQGEQEEGYVLTDASRLLLMDDSLSIRPFLLAMLDPVLTKPWHYLSAWFQNDDPTPFHTAHEQPFWDYAGHEPQLNNFFNEAMASDSRLVISVLLKEGKGLFEGLNSLVDVGGGTGKVAKAIANAFPHLNCTVLDLPHVVAGLQESKNLNYFAGDMFEAIPPADAILLKWILHDWSDEECVKILKRCREAIPSKENGGKVIIIDMIMMKNQGDYKSVETQLYFDMVMMILVTGRERDENEWEKLFLDAGFSHYKITPILGLRSLIEVYP*

>VIT_212s0028g02950|VIT_212s0028g02950.1|Vvinifera_v2.1

MDPANSEMSSELLQAQAHVWNHIFNFVNSMSLKCAIQLGIPDIIHNHGKPMTLPQLVAKLPVHPKKTQYVYRLMRVLVQSDFFAAQRAQQSEEEEGYVLTHASRLLLKDDSLSVRPFLLAMLDPTLTKPWHYVSAWFQNDDPTPFDTAHERTLWDYAGHEPQLNYFFNEAMASDARLVTSVLVKEGKGVFEGLNSLVDVGGGTGTVAKAIANAFPHLKCTVLDLPHVVAGLQGSKNLNYFAGDMFEAIPPADAILLKWILHDWSDEECVKILKRCREAIPSKERGGKVIIIDMMVKNQKGDCKSRETQLFFDMLMMVLVTGKEREEKEWEKLFLEAGFSHYKITPILGLRSLIEVYP*

>VIT_212s0028g02700|VIT_212s0028g02700.1|Vvinifera_v2.1

MDLANGGRSSELLQAQTHVWNHIFNFINSMSLKCAIQLGIPDIIHNHCQPMTLHELVAKLPVRPNKTLCVHRLMRILVHSGFFTMQRVQESADEEGYVLANASRLLLKDHPLSVTPFLLAMLDPDLTEPWHYVSAWFQNDDPTPFFTAHGRTIWDYGCHEPRFNNFFNEAMASDARLVTSVLVKECKGAFEGLNSFVDVGGGTGTVAKTIVEAFPHLHSTVLDLPHVVADLQGGKSLTYLAGDMFEAIPPADAILLKWILHDWSDEECLKILKQCREAIPSKEKGGKVMIIDMAIQNKKGDDDESNVETQLFFDMLMMVLLPGREREEKEWKKLFLDSGFSGYKITPILGLRSLIEVYP*

>VIT_212s0028g02740|VIT_212s0028g02740.1|Vvinifera_v2.1

MDLANGERSSELLQAQAHVWNHIFNFINSMSLKCAIQLGIPDIIHNHCKPMTLHELVAKLPVRPNKTLCVHRLMRILVHSGFFTKQRVQESADEEGYVLANASRLLLKDHPLSITPFLLLLDPDLTEPWDYASAWFQNDDPTPFFTAHGRTLWDYGCHEPRFNNFFNEGMASDARLVTSVLVKECKGAFEGLNSFVDVGGGTGTVAKTIVEAFPHLHGTVLDLPHVVADLRGSKNLTYLAGDLFEAIPPADAILLKWILHDWSDEECLKILKQCREAIPSKEKGGKVMIIDMAIQNKKGDDDESNVETQLFFDMLMMVLLPGREREEKEWKKLFLDSGFSGYKITPILGLRSLIEVYP*

>VIT_212s0028g01880|VIT_212s0028g01880.1|Vvinifera_v2.1

MDLANGVISAELLHAQAHVWNHIFNFIKSMSLKCAIQLGIPDIIHNHGKPMTLPELVAKLPVHPKRSQCVYRLMRILVHSGFLAAQRVQQGKEEEGYVLTDASRLLLMDDSLSIRPLVLAMLDPILTKPWHYLSAWFQNDDPTPFHTAYERSFWDYAGHEPQLNNSFNEAMASDARLLTSVLLKEGQGVFAGLNSLVDVGGGTGKVAKAIANAFPHLNCTVLDLSHVVAGLQGSKNLNYFAGDMFEAIPPADAILLKWILHDWSNEECVKILKRCREAIPSKENGGKVIIIDMIMMKNQGDYKSTETQLFFDMTMMIFAPGRERDENEWEKLFLDAGFSHYKITPILGLRSLIEVYP*

>VIT_212s0028g02830|VIT_212s0028g02830.4|Vvinifera_v2.1

MDLANGGRSSELLQAQTHVWNHIFNFINSMSLKCAIQLGIPDIIHNHCQPMTLHELVAKLPVRPNKTLCVHRLMRILVHSGFFTMQRVQESADEEGYALANASRLLLKDHPLSATPFLLAMLDPDLTEPWHYVSAWFQNDDPTPFFTAHGRTIWDYGCHEPRFNNFFNEAMASDARLVTSVLIKECKGAFEGLNSFVDVGGGTGTVAKTIVDAFPHLHSTVLDLPHVVADLQGSKNLTYLAGDMFEAIPPADAILLKQWILHDWSDEECLKILEQCRGAIPSKEKGGKVMIIDMAILNKKGDEDESKVETQLFMDMLMMVLYPGREREEKEWKKLFLDSGFSGYKITPILGLRSLIEVYP*

>VIT_212s0028g02850|VIT_212s0028g02850.1|Vvinifera_v2.1

MALAVGETSTELLHAHAHVWNHIFNFINSMSLKCAIELGIPDIIHNHGKPMTLSELVAELPVNPEKTKCVYRLMRLLVQSGFFTRKRVQESGQEEGYVLTHASRLLLKDDPLSARPFLLAMLDPVLITPWQYVSAWFQNDDPTPFDTAHGRTFWDYAGHEPKLNNFFNEAMASDARLVTSVLIKDCKGIFVGLNSLVDVGGGTGTVARAIANAFPHLNCTVLDLPHVVAGLEGSKNLNYLAGDMFEAIPPADAILLKQWILHDWNHDECVKILKRCRDAIPSKEKGGKVIIIDMMMENQKADDESIETQLFWDMLMMIVLTGQERNIKDWEKLFFDAGFSGYKITPMLGLRSLIEVYP*

>VIT_212s0028g02880|VIT_212s0028g02880.1|Vvinifera_v2.1

MDPAKGEISSELLQAQAHVWNHIFNFVNSMSLKCAIQLGIPDIIHNHGKPMTLPELVAKLPVHPKKTQYVYRLMRVLVQSDFFAAQRAQQSEEEEGYVLTHASQLLLKDDSLSVRPFLLAMLDPTLTKPWHYVSAWFQNDDPTPFNTAHERTLWDYAGHEPQLNYFINEAMASDARLVTSVLVKEGKGVFEGLNSLVDVGGGTGTVAKAIANAFPHLNCTVLDLPHVVAGLQGSKNLNYFAGDMFEPIPPADAILLKWILHDWSDEECVKILKRCREAIPSKEKGGKVIIIDMMMKNQKGDCKSRETQLFFDMLMMVLVTGKEREEKEWEKLFLDAGFSHYKITPILGLRSLIEVYP*

>VIT_212s0028g01940|VIT_212s0028g01940.1|Vvinifera_v2.1

MDLANGEISAELLHAQAHVWNHIFNFIKSMSLKCATQLGIPDIIHNHGKPMTLPELVAKLPVHPKRSQCVYRLMRILVHSGFLAAQRVQQGEEEEGYVLTDASRLLLMDDSLSIRPLVLAMLDPILTKPWHYLSAWFQNDDPTPFHTAYERPFWDYAGHEPQLNNSFNEAMASDARLLTSVLLKEGKGVFAGLNSLVDVGGGTGKVAKAIANAFPHLNCTVLDLPHVVAGLQGSKNLNYFAGDMFEAIPPADAILLKWILHDWSDEECVKILKRCREAIPSKENGGKVIIIDMIMMKNQGDYKSTETQLFFDMTMMIFAAGRERDENEWEKLFLDAGFSHYKITPILGLRSLIEVYP*

>VIT_212s0028g02710|VIT_212s0028g02710.1|Vvinifera_v2.1

MDFANGEISAELLHAQAHVWNHILNFIKSVSLKCAIQLGIPDIIHNHGKPMTLPELVAKLPVHPKRSQCVYRLMRILVHSGFLAAQRVQQGEEEEGYVLTDASRLLLMDDSLSIRPLLLAMLDPILTKPWHYLSAWFQNDDPTPFHTAHERPFWDYAGHEPQLNNFFNEAMASDARLVTSVLLKEGKGVFEGLNSLVDVGGGTGQVAKAIANAFPHLNCTVLDLPHVVAGLQGSKNLNYFAGDIFEAIPPADAILLKWILHDWSDEECVKILKRCREAIPSKENGGKVIIIDMIMMKNQGDYKSIETQLFFDMTMMIFAAGRERDENEWEKLFLDAGFSHYKITPILGLRSLIEVYP*

>VIT_212s0059g01790|VIT_212s0059g01790.1|Vvinifera_v2.1

MDRKQKMVSRSENDDVLKISREADEAELMLQGQANIWRHMFAFADSMVLKCALELRIADIIHSHARPITLSQIATCIDSPSPDITCLARIMRFLVCAKIFTAAPPPQSDGGETLYGLTPSSKWLLHDAELSLAPMVLMENHPFLMAPWHCLGTCVKEGGIAFEKAHGRQIWDFASENPEFNKLFNDGMACTAKVVMGEVVAAYKDGFGSIRTLVDVGGGTGGAVAEVVKAYPHIKGINFDLPHVVASAPAYEGVSHVGGDMFESIPNADAIFMKWIMHDWSDEDCIKILKNCRKAVPEKTGKIIIVDGVIREDSDDPFDKTRLVFDLLMIAHSSNGKERSEVEWKKVLEEGGFPRYRILEISISTLPMIIEAYPE*

>VIT_212s0059g01750|VIT_212s0059g01750.1|Vvinifera_v2.1

MVGTSENGDVLKVSSEADETELMLQGQANIWRHMFAFADSMALKCAVELRIADIIHSHARPITLSQIATCIDSPSPDITCLARIMRFLVRAKIFTAAPPPQSDGGETLYGLTPSSKWLLHDAELSLAPMVLMENHPSLMAPWHCFGTCVKEGGIAFEKAHGHQIWDLASEKPEFNKLFNDGMACTAKISIKAVIAAYKDGFGSIGTLVDVGGGTGGAVAEVVKAYPHIKGINFDLPHVVATAPAYEGVSHVGGDMFESIPDADAIFMKWILHDWNDEDCVKILKNCRKAIPEKTGKVIIVDGVIREDGYEPFDETRLVLDLVMMAHTSHGQERTEVEWKKLLEEGGFPRYRILKIPTLQMIIEAYPV*

>VIT_210s0003g00480|VIT_210s0003g00480.1|Vvinifera_v2.1

MDLIHGEGGSELFQAQSHMYKHIFNFINSMALKCAVELGIPDIIHTHNHPISLPQLVSALHLPPTKTRYVNRLMRVLVHSGFFATAKVHENQEEEDEGYVLTTSSRLLLSKDNNNVPNLSAFVLAMLDPVLVTPWHFLGDWFRGNDLTAFDTAHGKSFWDYGSHDHKFFNLFNEAMASDSRMMSLVIKDCRPVFEGLGSLVDVGGGKGLIARIISEAFPQLKCTVFDLPHVVSNFPECGNLKYVGGDMFQSVPTADAILLKLVLHSLSDEECVKILKKCREAIPSKEKGGKVIVIDIVINEKKEEHDITEAKLLFDLLMMTVVTGRERNEKEWEKLFLEAGFSHYKINPIFGLRSLIEVFP*

>VIT_210s0003g00460|VIT_210s0003g00460.1|Vvinifera_v2.1

MDLIHGEGAGELFQAQSHMYKHICSFMNSMALKCAVQLGIPDIIHNHAHPTTLPQLVSALHIPPTKASCVHRLMRLLVHSGFFAIAKVHEHEEEEGYILTPSSRLLFKDNPTSNLSPFVLTVLHPALVTPWHFFGDWLRGDDLTAFETAHGVSFWDHGSHNPEIFNLFNEGMASDSQMMSVVNFRELKPVFEGLSSLVDLGGGTGLLARIISEAFPQLKCTVFDLPHVVANLPESRNLEYVGGDMFQSVPSADAILLKCVLHDWSDEDCLKILKKCREAIRSEEEGGKVIIIDVVINEKKDEDDITETKLLMDMMMMTLVNGRERNEKEWERLFLEAGFRHYKITPIFGLRSLIEVFP*

>VIT_210s0003g00470|VIT_210s0003g00470.1|Vvinifera_v2.1

MDHINGQGRSELFEAQSFIYKHVFSFMDSMSLKCAVQLGIPDAIHNHNQPITLPELASAIQVPPEKTSRLHQLMRLLVHSGFFAMQKVDENQEGYVLTPPSRLLVKGNATSLAPIVLGMLDPVLVTPWHFLGSWLQGSSLTAFEAAHGMDLWNYGNQNPEFFSLIGEIMATDSRMMSLAIRECKEIFEGLSSLVDVGGGTGTMARGICEAFPHLKCTVLDLPQVVANLPKSENLDYVGGDMFQSIPSADAIFIKSVLHNWGDEDCVKILKRCREAIPSSAEGGKVIIIDLVLSNKKDEHELAKTKLFNDMMMMVLVAGKERCEEEWEKLFLEAGFSHYKITPRFGVLSLIEVYP*

>VIT_210s0003g00440|VIT_210s0003g00440.1|Vvinifera_v2.1

MALRCAVQLGIPDIIHNHAHPTTLPQLVSALHIPPTKASCVHRLMRLLVHSGFFAMAKVHEHEEEEGYILTPSSRLLLKDNPTSNLSPLVLTMLHPVLVTPWHFFGDWLRGDDLTAFETAHGVSFWDHGSHNPEIFNLFNEGMASDSQMMSVVNFRELKPVFEGLSSLVDLGGGTGLLARIISEAFPQLKCTVFDLPHVVANLPESRNLEYVGGDMFQSVPSADAILLKCVLHDWSDEDCLKILKKCREAIRSEEEGGKVIIIDVVINEKKDEDDITETKLLMDMMMMTLVNGRERNEKEWEKLFLEAGFRHHKISPIFGLRSLIEVFP*

>Zm00001d049020_T001

MELSPNNSTDQSLLDAQLELWHTTFAFMKSMALKSAIHLRIADAIHLHGGAASLSQILSKVHLHPSRVSSLRRLMRVLTTTNVFGTQQPAGGSDDDSEPVYTLTPVSRLLIASQSSQLAQTPLAAMVLDPTIVSPFFELAAWFQHELPDPCIFKHTHGRGIWELTKDDATFDALVNDGLASDSQLIVDVAIKQSAEVFQGISSLVDVGGGIGAAAQAISKAFPHVKCSVLDLAHVVAKAPTHTDVQFIAGDMFESIPPADAVLLKSVLHDWDHDDCVKILKNCKKAIPPREAGGKVIIINMVVGAGPSDMKHKEMQAIFDVYIMFINGMERDEQEWSKIFSEAGYSDYRIIPVLGVRSIIEVYP*

>Zm00001d038702_T001

MAPANVQHTSTNQQTMLDAQLQLWHHTIGYVKSMALKAAVDLRIADAIHQHGGSATLPQIVTKVTLHPSKIPCLRRLMRVLTLTGVFSVHAGGDEPVYGLTPASRLMVSPGPNLTPFLTLLLSTFFVSSFLDLNEWFQHETGPSPFELANGRDIWALSGHDASFGKLFDDGMVADSGFIMEVVVKECGDVFRGVGSLVDVAGGLGGATQTIAKAFPDMECSVLDLSHVVANAPTDTTVKYIAGDMFESIPSANVVFLKWILHDWGDAECVKILKNCKKAIASQEEGKVVILDMVVGAGSSDEKHVEMQIVFDLFMMFINGTERDETEWKKIIFEAGFSRYKIIPVLGVRSIIEVYP*

>Zm00001d038703_T001

MTLSKKQGAGTDQQALLDAQLQLWHHTIAFVKSMALKAAVDLRIADAIHLHGGSATLSQIVTKVTLHPSKIPCLRRLMRVLTLTGVFSVNSGAVVDEPVYGLTPASRLLVGPGLNQTPFLTLMLSTFFVSSFLGLDEWFQHETGPSPFELANGRDIWTLSGHDASFGKLFDDGMVADSGFIMDVVVKECGDVFRGVGSLVDVAGGLGGATQTIAKAFPDVACSVLDLSHVVANAPTDTTVKYIAGDMFESIPSANVVFLKWILHDWGDAECVKILKNCKKAIASQEEGKVVILDMVVGAGSSDEKHVETQIVFDLFMMFINGTERDETEWKKIIFEAGFSRYKIIPVLGVRSIIEVYP*

>Zm00001d038699_T001

MALTTSTNNQALLDAQLELWHTSFAFIKSMALKSAVDLGIADAIHSHGGNATLPQIVSRAALHPSKIPCLRRLMRVLTAAGIFSAAHRSPDDGGGELVYGLTPASQLLVGGSSSLAPFMSLVLHQVYVSPFLGLGTWLQHERSDPTLFEMTHGLTAWDLNDHKPAFGELFNQGMVCDSSFVMDIVVKECGDVFRGLSSLVDVAGGLGAAALAISTAFPHVQCSVLDLPHVAANAPANTSVKYIAGDMFESIPPADAVFLKWVLHDWGDADCIKILKNCKKAIPSRDAGGKVIIVDMVVGGQSSNIKHKETQVLFDLFIMTINGAERDEHEWKKIISEAGFSDYKIIPVLGVRSIIEVYP*

>Zm00001d032083_T001

MSSVQEELNNTQDMLQGYVELYNYSLSYVKTMAIGCAIQLGIPSAIHRRGGAATISDIITETGVDPSKLPYLRRLMRVLTVSSILATTGTDETETESDDSTVYKLTPASRLLVSGAGAPTSCDISPMLDLLMRPTTSVATYFSLEEWFKDAGATATLFEVAHGMSPWSLTKNDALYNKTLNDGCAADSNFAMDTLLREPRAAGIFRGLGSLVDVGGGHGAAAMAIARAFPHIRCSVLDLEQVVSGAPDDGTVKFIAGDMFESIPAADCVLLKYVLHCWDDESSVKILRQCKRAIPARDAGGKVVIMNMVVGYGSSDRFVKETQVMCDMWMMRYVGVEREEHEWKRIFLEAGFSDYRITPTALGFQSVIEVFP*

>Zm00001d023257_T001

MSPCAHAEQQEPDAPPLHNDHHRHQTYVELYHHGLHHVKSSALLCAVGLGIPGAIHRRGGAATVSDLVSGTGVHPAKRAQLRRLMRMLACFGIFGAAGGDEGESETVYTLTPVSSVLVGDKDKEASATSSASSPDMSALLRLLARPSTSVSTFFDMEEWFRDGGTTTLFETALGVPPWSLTKSDAAYNRAMNEACVADTSLSMDIMLKDTSGGASSVFGGGRLTSLVDVGGGHGAAAIAIANAFPHIKCTVLDLKQVIDNKVPPAAAAGTDRIINSTVQFVAGDMFESIPPADAVLLRHVLDCWDDEHCVKILGQCKRAIPGREAGGKVIIINVVVGYGSPDKLVKETQVLFDMYMMRYDGSEREEHEWREIFSRAGFSDYKITPTIGFHSIIEVFP*

>Zm00001d010736_T001

MALAGITNQDLLDAQVELWHSTFAYIKSMALKSALDLGLADAIYHHGGSATLPQIVDRVTLHPSKTLHLRRLMRVLATTGVFSVQHPSPLGDDSSSASDSEPVYKLTAVSALLVGPRRSHVPLAAFVVDPALVTPFFELGKWLQRELPGPCIFEHAHGQTIWEHANGDAAFNALLNDGMLSDSHFIMDIASKECAHVFQGISSLVDVGGGLGAAAQAISLAFPGVKCSVLDLDHVVAKAPSDTQVSYIGGDMFESVPPADAMFLKWVLHDWGHEECVKILRNCRKAIPPREGGGKVIIIDMVVGAGPADPRHREMQALFDLYIMVVNGMERDEQEWKRIFVEAGFTDYRVTPVLGVRSIIEVYP*

>Zm00001d049303_T001

MRRHMAPIKEQKHTTSAEQQVMLDAELQLWNHTFGYVKSMALKAAIDLGIPEAIHQHGGTATLPQIVTRVKLHPSKTPCLRRLMRVLTLTGVFGAQEPHDDDGGCDDELVYTLTPASRLLVGSPGQNVGPFLTLMLGPIFVSSFLDLRGWFQHETPDPSPFKMTHGRDIWELAAHDAAFGRLFDAGMVADSSFIMDVVVRECGGVFEGISSLVDVAGGLGGATQTIAKAFPNLECSVLDLPNVVASAPADTAVKYVPGDMFESVPAADAVFLKWIMHDWGDADCVKILKNCKKAIPAQGGKVIILDIVVGAGSSCDRKNVETQCLFDLFIMFINGAERDERQWKKIIFEAGFTSYKIIPVLGIRSIIEICL*

>Zm00001d053156_T001

MGSIMEQRCNNDQQALLDAQLELWHSTFAFIKSMAFKSALELGIADAIHCHGGTATLTQIATKAALHPSKTPCLRRLMRALTVAGIFSVAKNSSDDDGDQHVYGLTPASRLLVGSSQNLAPTLSLILNNVFVAPFLDLGTWFESALPATDLPLFELSHRKNVWDVVGHDPSVSQLFNAGMVADTRFLMDIAVRECGDVFQGIRSLVDVGGGHGAAAQAISAAFPAIQCTVLDLAHVVATAPACAGLSFVAGDMFEAIPPANAVFLKWIMHDWGDTECVTILKNCKKAIPPRDAGGKVIIVDTVVGAGPPNLKNKETQVMSDLFFMIVNGLERDEQEWRNVIFEAGFSDYIIIPVLGVRSIIELYP*

>Zm00001d047192_T001

MALSTQDLLEAHVELWHQSLCYAKSLALAVALDLRIPDAIHHHGGSATLPQILAETAAHPSKLRALRRLMRVLTVSGTFSVEQQPPAGGDDSTVDAPDEAVYRLTAASRFLVSDEVSSSTLAPFVSLALHPIAVSPHTMGICAWFRQEQREPSPYGLAFQQTFPTIWEHADDVNALLNKGMVADSRFLMPIMLRECGEVFRGIESLVDVGGGHGGATAAIAAAFPHLKCSVLDLPHVVAGAPSDVNVQFVAGNMFQSIPPATAVFLKTTLHDWGDDECVKILKNCRQAISPRDAGGKVIILDMVVGYGQPNITHLETQVMFDLYIMTVNGAERDEQEWKKIFIEAGFKDYKILPILGALSVIEVYP*

>Zm00001d038700_T001

MAPAKAQHTSTNQQTLLDAQLQLWHHAFGYVKSMALKAALDLGIPDAIHQHGGSATLPQIVTELAALHPSKTPCLRRLMRVLTLTGVFGVVVQHSTTDGGSDLVYELTPASRLLVGSAPSNGPNVSPFLNMILGTAFVSSFLDLGEWFQHELPDPSPFKLAHGQHVWDMARHDASFAKLCDSGMVADSGFIMDVMVEECADGVFRGISSLVDVAGGLGGAAQAIAKAFPHIDCSVLDLPNVVAAAPTSTDVKYIAGDMFQSIPAADVVFLKWVLHDWGDAECVKILQNCKKAIPSEGGKVIIMDIVVGAGSSDRKHVETQVLFDLFIMAINGAERDEEEWKKIIFEAGFSSYNIIPVLGVRSIIEVYP*

>Zm00001d029359_T001

MALMQESSSQDLLQAHDELLHHSLCFAKSLALAVALDLRIPDAIHHHGAGGATLLQILAETALHPSKLRALRRLMRVLTVTGIFSVVEQPPAGGGDDSTVHTSDDEAVVVYRLTAASRFLVSDDVSTATLAPFVSLALQPIAACPHALGISAWFRQEQHEPSPYGLAFRQTPTIWEHADDVNALLNKGMAADSRFLMPIVLRECGETFRGIDSLVDVGGGHGGAAATIAAAFPHLKCSVLDLPHVVAGAPSDGNVQFVAGNMFESIPPATAVFLKKTLHDWGDDECVKILKNCKQAISPRDAGGKVIILDVVVGYKQSNIKHQETQVMFDLYMMAVNGVERDEQEWKKIFTEAGFKDYKILPVIGDVSVIIEVYP*

>Zm00001d029356_T001

MALIMQESSSHDLLQAHDELLHHSLCFAKSLALAVALDLRIPDAIHHHGAGGATLLQILAETALHPSKLRALRRLMRVLTVTGIFSVEQPPAGGGDNSTVHTSDDEAVVVYRLTAASRFLVSDEVSTATLAPFVSLALQPIAACPHALGISAWFRQEQHEPSPYGLAFRQTPTIWEHADDVNALLNKGMAADSRFLMPIVLRECGETFRGIDSLVDVGGGHGGAAAAIAAAFPHLKCSVLDLPHVVAGAPSDGNVHFVAGNMFESIPPATAVFLKKTLHDWGDDECVKILKNCKQAISPRDAGGKVIILDVVVGYKQSNIKHQETQVMFDLYMMAVNGVERDEQEWKKIFIEAGFKDYKILPVIGDVSVIIEVYP*

>Zm00001d052683_T001

MALSKEQKLTISEQQHTASSEQQVALDAELQLWNHTFGYVKSMALKAALDLGIPDAIHQHGGSATIPQIVTRITLHPSKTPCLRRLMRVLTVTGVFGTQEPHDDGGGCDDELVYTLTPASRLLVGPPGQNVSPLLNVMLCPIFVSSFLDLRGWFQHEMPDPSPFKVTHGRDIWELAAHDAGFSRLFDAGMVADSGFIMNVVVRECGSGTVFQGISSLVDVGGGFGGATQAIAKAFPHLECSVLDLPNVVAGAPADTAVKYVAGDMFESVSSADAVFLKSIIHDWGDADCVKILKNCKKAIPAQGGKVIILDIVVGAGSSSCDRKNVETQCLFDLYIMTINGVERDEREWKKIIFEAGFTSYKIIPVLGTRSIIEVCP*

>Zm00001d004921_T001

MALMQESSQDLLEAHDELFHHCLCFAKSLALAVAQDLRIPDAIHHHGGGATLHQILAEAALHPSKLRALRRLMRVLTVSGVFTVQYSSTVDASDGADVVYRLTAASRFLVSDSDEAGTASLAPFANLALHPIAISPHAVGICAWFRQEQHDPSPYGLAFRQIPTIWEHADNVNALLNKGLLAESRFLMPIVLRECGDEVFRGIDSLVDVGGGHGGAAATIAAAFPHVKCSVLDLPHVVAGAPSDACVQFVAGNMFHSIPPATAVFFKTTLCDWGDDECIKILKNCKQAISPRDEGGKVIIMDVVVGYGQSNMKRLETQVMFDLVMMAVNGVERDEQEWKEMFIEAGFKDYKIRPVAGLMSVIEVYP*

>Zm00001d047194_T001 MAFTEESSQDLLQAHDELWHQSVSYLKSLALTVALDLRIPDAIHHHGGGATLLQILDKTALHQSKLRALRRLMRVLTVSGTFSVVQQPPCGDDDSTVYRLTAASRFLVSEEVSSATLAPFMSVVLHPISQSSHARGICAWFRQEHHDPSAFGLAFGQAPTIWEHADDTNAILNKGLAAQSRFLVPVMLRECGEAVFRGIDSLVDVGGGHGGAATAIAAAFPHLKCSVLDLPHVVAGAPSDGNVQFVAGDMFQSIPPATAVFLKTALHDWGDDECVKILKNCRQAISPCDEGGKVIIMDMVVGYDESNTKRLEVQILFDLFIMMVNGAERDEQEWKKIFIQAGFKDYKILPVVGSLSVIEVYP*

>Zm00001d023254_T001 MSPCAHAEQQEPDAPPLHNDHHRHQTYVELYHHGLHHVKSSALLCAVGLGIPGAIHRRGGAATVSDLVSGTGVHPAKRAQLRRLMRMLACFGIFGAAGGDEGESETVYTLTPVSSVLVGDKDKEASATSSASSPDMSALLRLLARPSTSVSTFFDMEEWFRDGGTTTLFETALGVASWSLTKSDAAYNRAMNEACVADTSLSMDIMLKDTGGCCGGGGASSVFGGGRLTSLVDVGGGHGAAAIAIANAFPHIKCTVLDLKQVIDKVPPAAATGTDRIINSTAATAVQFVAGDMFESIPPADAVLLRHVLDCWDDEHCVKILGQCKRAIPGREAGGKVIIINVVVGYGSPDKLVKETQVLFDMYMMRYDGSEREEHEWREIFSRAGFSDYKITPTLGFHSIIEVFP*

>Zm00001d049181_T001 MGHQAQHATDDTEELLAAHRELWCHALGYVKSMALKCALDLRIPDIIQRCGGSATLGQLLAASEVPASNLGYLRRVMRTLTAMRIFAVVGHGHGPDKADDPADDDATAVSYRLTPASRLLATSDDDDDASKNNLSIHPNISSHVRPKNVSLLFSMAEWMKDEQALSVSLYETVNRKCMWACVEDDAATRACFYESMDADTRLVMQAVIRKCPAVFDDGLTSLVDVGGARGTAAAAVVAAFPHIQKCTVMDLPHIVAEAPAGTGLCFHGGDMFEHIPSADAVMLKVYIYTNWYQMDWILHDWDDDKCVKIMERCKEAISGKEGRGGKVIIIETVLGSRPDDDATCKETYVLDLQILSFVNGAEREEHEWRRIFLAAGFRDYKITHTRGIPSIIEVFP*

> LOC_Os01g54969.1 (OsASMT1)

MDASRNSDLAADELLRAQAELWNHIFAYTKSMSLRCAVELGIPDAVHRRGGAVTVPELVAELALPRSREPFLRRLMRLLAHGGIFDAAAGAEDAYGLTAVSRLLVSAPGGAGQGLSPFARAMLHPIIVSPSISLASWFRAAAADDDDEGADAPRVPFAAVHGGRELWAVAKDDPGFGAAFNDAMACDGRFVMDVLLHGHHHGGAQLFRGITSLVDVGGGSGGAARASAAAFPHVRCTVLELPQVVATVPPGDGGVEFVAGDMFDHVPKADAVLLKWILHGWGDEECVRILRRCREAVPAREDGRRVIVMDLVVGSSSSLGDGARDTETQLLWDVMMMGVVGSPERDEREFCKIFHDAGFSGYKILHVLGIRSVIEVYP

> LOC_Os04g11970.1 (OsASMT2)

MSCTEQELSTQDMLQGHIDLHHHLYGYHKSMALLCATDLGIPGAIHRRGGAATISDIVADTMIPPAKLPHLRRLMRVLSVSGIFAVEEDVYKLTPASRILVGDKASCNFSPLVHLVVSPAMLTTFSSLSPWFRDGRNASPTALFEMAHGMPPWEMMKRDDTMNSALNDACVADSSFLMEIALRERGDVVFRGLRSLVDVGGGHGGAAMAIAKAFPDIKCSVLDLPHVISQAPDDGTVCFIAGDMFEDIPPADAVLLKHVLHCWDADDCVKIVGQCKKAIPVRGDGGKVILINPVIGYGVKQDSTLKETSAG

> LOC_Os05g43930.1 (OsASMT3)

MELTESKCDGGQEVSLLDAQLELYWNTFAVIKSMALKSALDLRIADAVHLHGGAATLAEIASEVALHPSKIPCLRRLMRALTVSGVFAAAVKPGDGGGGEPVYELTPSSRLLVGSSNLSGIMSMILHPTLVVPFLGVGEWLRRDREPPEEDPYCIFKQAHGRSLWELAGRDAAFDALINDGMVSDSRVIMDYVVREHGEVFRGIASLVDLAGGLGAAAQVISKAFPEVRCSVMDLGHVVAKAPAGTDVEYIAGDMFESVPPADAVFLKWVLHDWGDDDCIKILKNCKKAIPPRDKGGKVIIMDIVVGAGPSDQKHREVQALFDMYIMFVNGIERDEQEWKKVFMGAGFSGYKIMPVLGFRSMIEVYP

>LOC_Os05g43940.1 (OsASMT4)

MGSLELSKNKCNGGQEVSLLDAQLELYSNTFAVIKSMALKSALDLGIADAVHRHGGAATLAEIASEVALHPSKIPCLRRLMRALTVSGVFAAAVKPGDGGGGEPVYELTPSSRLLVGSSNLSGIMSMILHPTLVVPFLGVGEWLRRDREPPEEDPYCIFKQAHGRSLWELAGRDAAFDALINDGMVSDSRVIMDYVVREHGEVFRGIASLVDLAGGLGAAAQVISKAFPEVRCSVMDLGHVVAKAPAGTDVEYIAGDMFESVPPADAVFLKWVLHDWGDNDCIKILKNCKKSITPRDKGGKVIIMDIVVGAGPSDQKHREVQALFDMYIMLVNGIERDEQEWKKVFVEAGFSGYKIMPILGFRSMIEVYP

> LOC_Os06g13280.1 (OsASMT5)

MSCHAEQELSTPDMLQGHIELHHHLFSYLKSKALRCAADLGVPSAIHRRGGAATISDIAADTGVHPAKLPHLRRIMRVLTVAGIFAANDEPSSSADQDGDAAGETAYTLTPPSRLLVGDRATCNMDPMMRFLAAHGGVPAWEMTKRDNSYSRALNEACAGDTSFVMDIAVREGGDVFRGLSSLVDVGGGHGAAAMAVARAFPHIKCSVLDLPQAISEAPADGTVNFVAGNMFEYIPPANAVFLKYVLHCWGEEDCIKILQQCKKAIPARGDGGKVIIINTVVGSGEPQDNALKETQVLFDVYMMGIGGGEREEHEWKKIFFEAGFSDYKIKPILGFISVIEVYP

>LOC_Os06g16960.1 (OsASMT6)

MALKCAAELGIPAAIHRRGGAATLRDIVADVALRQAKVPHLRRLMRVLTVSGIFAMKQQQPASSGEAVYTLTPASRLLVAGAGGGHDMSPMLRFLVHPTALTPFFSLHAWFRVDDEEEEEEPVAAGGGAAMSLFEMAHGFPRWEMTGRDAAYGAVLNDAMAADSRFVMEVVFREGGGDVFRGIGSLVDVGGGHGAAAAAVAAAFPHVKCSVLDLPQVVRKAPPDAGDVRFVAGDMFEYVPPADAVLLKYVLHCFGDDDCVKILRRCKEAIPARDAGGKVIIINMVIGSGSQRDIFKETQVLFDLYMMYIDGVEREEKEWENIFSKAGFSAYKIMPILGFLSIIEVYP

> LOC_Os07g27880.1 (OsASMT7)

MEKEQQAGHGKLPHEQLLLQASTELMNLSLGYARSMALGCAAKLGVADAIHRAGGRATLHDLHAALSLHPTKLPFLRRVMRVLVASGVFAQVEEEEEEEDHYRLTPVSSLLVTAGDGRGRSLLPLVLFQLSPLCVTPATSMAEWLRSGGEEETAFEMVHGVGLWGACSRAPDLGEHFNDAMAADSRFIMDMAINGSGRQVFDKITSMVDVAGGTGAAARAVAAAFPHIKCTVLDLPHVIDSIPADHGDVVQFVAGDMMDFIPKADALLLKFVLHDWSDEDCIKILKRCKEAIIPSRAAGGKIIILDVVVGSSSEAICQGTQQLFDLIISVTRKSGARYSRRQDSLNTRLAQC

> LOC_Os07g27970.1 (OsASMT8)

MEHEQLLQASTELMNHSLGYIRSMALGCAAKLGVADAIHHAGGRATMDDLRAALSLHPSKLPYFLRRVMRVLVASGVFAHDEEEDDDDIYRVTPVSSLLVTATGGNGGRSLLPFVLLQLSPPIYVTPATSMAEWLTSGEEETPFEMTHGAGLWTVCSRDPELGELFNDAMAADSAFIMDVAIRGAGRQVFDKITSLVDVAGGTGTAARVVAAAFPHIKCTVLDLPHVIDSIPADHRGRDVVKFVAGDMMDFIPRADALLLKFVLHDWSDEDCMKILKRCKEAIPSREAGGKVIVIDVVVGSSTQAMCHGTQLLFDLLISTTLPGMQRGEKEWCKVFKEAGFTDYKISPVLGIRSIIEVFP

> LOC_Os07g28040.1 (OsASMT9)

MEHEQLVQASTELMHHSLGYVRSMALGCAAKLGVADAIHRAGGRATLHDLHAALSLHPTKLPFLRRVMRVLVASGVFAQVKEEEDHYRLTPVSSLLVTAGRTLLPFVLLQHSPLCVTPATSMAEWLKTGEEETAFEMAHGAGLWGACRRAPELGDFFNDAMAADSAFIMDAAIRGARQVFDKITSLVDVAGGTGAAARAVAAAFPHIKCTVLDLPHVIDSIPVDHGDVVQFVAGDMMDFIPQADALLLKFVLHDWSDEDCVKILKRCKEAIPSKDKGGKVIIIDVVVGSSSQAMCYGTQLLFDLTISMLTPGMERDEKEWFKIFNEAGFTEYKISPVLGIRSIIEVFP

> LOC_Os08g07260.1 (OsASMT10)

MALKCAIDLGIPSAIHRNGGSASLPDLLATLPIAENKRPFLHRLMRFLTVSGIFTSADDGVYQLTRVSRLLVDSIPLNLLPSFLCLGEWFRDGGDTTPFAMAHGIDVWGAMSLDRALAAGFSASMAADSKFLAEIAIRRHVEAFMNVSSLVDVGGGDGSMARAIVKAFPHIKCLVLDLPHVVRGIPADGFVEYVAGDMMDFVPPANVVLLKLVLHDWSDEDCVRILSRCREAISNREGGKVIIIDTVIGSQSQQIYEAQLFLDLCMMTVTTGKEREEKEWHMIFLKAGFTQYKILPILGIKSLIEVYP

> LOC_Os08g19420.1 (OsASMT11)

MEKIQVTTPMADSSFNPEFLQAHAELWNLTFSYLKAMALECAIKLGIPNAIHRCGGSASLSELVISIPVPETRKPHLPRLMRFLAAVGVFSLDNPTIDEEVTEKGMGIYRLTPLSRLLVDGSIGGHGSLSPFVLSQTTKYHVSAAMNLSDWFMTEDKEVAIEMPFRAAHGTDLWGVMSRDANMNEVFNAGMGSDSRLAINFIISKCGEVFEGISSLVDVGGGTGTTARDIAKAFPHIKCSVLDLPNVIDTITVDGIIEYIAGDMMEQIPPTDAVLLKYILHDWNDEDCVKILKQCRNAIHAQKPGGKVIIIDIVVGSPSKDMFEAQVSFDLLMMVITSGKERDQHEWHKIFMDAGFSHYKTRPVLGFLAITELYP

> LOC_Os08g35310.1 (OsASMT12)

MDQKQDEILKKDRAELLQAQAELWCHTFGYLKSIALRCAVELGIPNAIHRNGGSASLPELLGTLPLAANKRSCLPRLMRFLVSFGIFKEDISREGTTTTTSVYQLTPVSRLLVDASSRGIFVLGNWLTSSDENTPFGMAHGMDFWDFTGHDAEYSMLFNKGMASDSHFVVNIVIHECAEVFVGVRSLVDVGGRNGAMAKAIADAFPHIKCYVLDLPHVIHGTPTDGIVEFVAGDMMHFVPSADVVLLKFVLHDWSDEDCVRILTRCKQAITNKEEGGKVIIIDTVIGSPSQQILEAQLSMDICMMTLTTGKEREERDWHKIFLEAGFTRYKIMPILGVRALIEVYP

>LOC_Os09g17560.1 (OsASMT13)

MAQNVQENEQVMSTEDLLQAQIELYHHCLAFIKSMALRAATDLRIPDAIHCNGGAATLTDLAAHVGLHPTKLSHLRRLMRVLTLSGIFTVHDGDGEATYTLTRVSRLLLSDGVERTHGLSQMVRVFVNPVAVASQFSLHEWFTVEKAAAVSLFEVAHGCTRWEMIANDSKDGSMFNAGMVEDSSVAMDIILRKSSNVFRGINSLVDVGGGYGAVAAAVVRAFPDIKCTVLDLPHIVAKAPSNNNIQFVGGDLFEFIPAADVVLLKCILHCWQHDDCVKIMRRCKEAISARDAGGKVILIEVVVGIGSNETVPKEMQLLFDVFMMYTDGIEREEHEWKKIFLEAGFSDYKIIPVLGVRSIIEVYP

>LOC_Os10g02840.1 (OsASMT14)

MTPFEVAHGCTRWEIIANDAKDGSVFNTAMVEDSRVAMDIILKESCGVFQGISSLVDVGGGHGAAAAAIATAFPNIKCTVLDLPHIVAEAPTTHSNIQFVGGDFFEFIPAADVVLLKYILHAWQDDDCVKILRRCKEAILARDAGGKVIIIEVVVGIGPKEIVPKEMQILFDVFMMYVDGIEREEHEWKKIFLEAGFSDYKITPVLGARSIIEVYP

> LOC_Os10g02880.1 (OsASMT15)

MAQRVQEEDEQMMSTDDLIQAQIKLYHHCFAFIKSTALWAAIDLRIADVIHRNGGAATLSDLALNVGLHPTKLSHLRRLMRVLTVTGIFAVEDRNGEAMYTLTRVSRLLLNSDGEGTHALSQMARVLANPLAVISHFSIHEWFTTEKATTMTPFEVAHGCTRWEMIANDAKDGSVFNAGMVEDSRVAMDIILKESCGIFQGISSLIDVGGGHGAAAAAIATAFPNIKCTVLDLPHIVAEAPATHSNIQFIGGDLFKFIPAADVVLLKCLLHCWQDDDCVKILRLCKEAIPARDAGGKVIIIEVVVGIGSEEIVPKEMQLLFDVFMMYIDGIEREEYEWKKIFLEAGFSDYKITPVLGARSIIEVYP

> LOC_Os11g12760.1 (OsASMT16)

MAGQANTIQVPTDAELLQAQADLWRHSFSYLTAMALRCAAKLGIPTAIHRLGGEEAAASLPDLMAALSLPASKQPFVRRLMRLLVAVGVFAADGVADERYRLTPLSRILVDGVAAADDHHHDVLQTPFVLTATSRQYVEAALGVDEWLRKDAPPAPAPVPSPFEDAHGAPLFDEATAAAIDPEFAAAADDALAAHDSLGIGAVLRECGDLLRGVASLTDCAGGDGATARAIAAAFPHIKCTVLDLPKVIDKAPVDDGVVNYVAGDMFHAVPPAQAVLLKLVLHFWSDDDCVKILSQCKKAIPSRKEGGKVIVIDILIEPSLGPAMLETQLLMDMAMMVNTRGRQRDESEWRDLFFRAGFSDYKIAKKLGARAVFEVYP

> LOC_Os11g33300.1 (OsASMT17)

MALIHGSEEVISHEEDMVGAFALLYHHVFSYIKPMALKCAVELGIPDAIHRRGGAATLANIAADTGVHASRLTDLRCLMKLLTTSGMFAATAATTDGGEGEPAVATSTMYTLTAASGLVVGPRGLSTVVRFAAGPVAVSPFFDMHAWLRAAPAAAPPAARSLFELAHGRSRWDAANADNDTMNAHSFVESQLLIEAVLRDHAGVFRGLRSLVDVGGGHGAVAKAIAAAFPDIKCTVMDLPHVVADAPVSDDGNLHLVAGDMFQSIPPADAVLLKYVLHCWGDDDCVRILRNCREAIPAREAGGKVIITELVLGSSAASRDANVAEAEDMHSLFLMCISGVGREEREWRAIFSDAGFGDYKITPVLGPISVIEVYP

>LOC_Os12g25450.1 (OsASMT18)

MATTQSNDHGAGLLDAQLELYANTLAVVKSMALKTAMDLGIADAIHHHGGAATLPQILTRVTLHPSKIPCLRRLMRVLTLTGVFAVEKPTAADEPPVYALTPVSRLLVSSGNLQQAPIMSLLLHPSCITPFLRIGDWLQRELPGSSIFEHTHGRSLWEVADGDAAFSKVFNDAMVSDSRLVMDVVVREHGDVFRRISSLVDVAGGHGTAAQAIARAFPEVKCSVMDLAHVVAKAPGGTGVEYIAGDMFESIPPANAVFLKWIMHDWGDDECVKVLKNAKKAIPSKDAGGKVIIIDVVVRAGSPDQKHIELQALFGAYMMLINGVERDEKEWKKVFIEAGFSGYKIIPVLGFRSIIEVYP

> LOC_Os12g25490.1 (OsASMT19)

MAPIQSNGQHATSSLDALSELYGNTFSVIKSMALKAALDLGIADAIHHHGGAATMAQIATRVTLHPSKIPCLRRLMRVLTLSGIFAVQKPAPGDAAAEADEAPMYTLTPVSRLLIGAGNQRHMMSMLLHPCFIAPFFRISDWLQLELPEPCMFKHTHGQSFWEMTNEDAAFNTVVNDGMASDSAFMMDILVREHGEVFQGISSLVDVAGGNGAAARAIAKAFPEVKCSVMDLAHVVADAPRGTGVEFIAGDMFDSIPAANAVFLKWIMHDWSDNDCVKILRNCKKAIPSRDAGGKVIIMDIVVGVGPSDQKHRDVQILFDALIMFVNGVERDEQEWKKLFVEAGFSSYKIMPVMGFRSIIEVYP

>ESW14071 pep chromosome:PhaVulg1_0:8:56528565:56529849:-1 gene:PHAVU_008G250700g transcript:ESW14071 gene_biotype:protein_coding transcript_biotype:protein_coding description:hypothetical protein

MGSHEEDQSAKLLKAQTHVWNHILSFINSMSLKCIVDLGIPDIIHNHGQPMSLSNLISSLPIHSSKTHFIHRLMRIMVHSGFFSQHNHTENELEVKYALTDSSLLLLKSNAMSVTPFLQAMLDPVLTTPWNQFANWFKNGNPTTFEMAQGKPFWEYAGSDPRINSLFNDAMASDAQLVTSVVIEKCKRVFMGLESLVDVGGGTGTMGKAIAKSFPQLECTVFDLPHVVSGLQGSENLKYVGGDMFEAIPPADAILLKWILHDWSDEDSVNILKKCKEALSRKGKEGKVIIIDMVVDKEMKDDESFETQLFFDMLMMVFFTGKERNKKEWVKLFSSAGFNNYKITPVVGLRSLIEIYP

>ESW14037 pep chromosome:PhaVulg1_0:8:56237670:56239124:1 gene:PHAVU_008G247600g transcript:ESW14037 gene_biotype:protein_coding transcript_biotype:protein_coding description:hypothetical protein

MESREEDQSAKLLRAQTHVWNHIFSFINSMSLKCIVDLGIPDIIHNHGQPMSLSNLISSLPIHSSKTHFIHRLMRIMVHSGFFSQHNPTENELELKYALTDSSLLLLKSNAMSVTPFLQAMLDPVMTNPWNQFSNWFKNGNPTTFEMENGKPFWEYASCDPRFNILFNDAMASDAQLVTSLVTEKCKGVFMGLESLVDVGGGTGIMGKAIAKSFPQLECTVFDLPHVVSGLQGSENLKYVGGDMFEAIPPADAILLKWILHDWSDEECVNILKKCKEALSRKGKEGKVIIIDMVVDKEMKDDESFETQLFFDILMMVLLTGKERNKKEWVKLFSSAGFNNYKITPFVGSRSLIEIYP

>ESW13098 pep chromosome:PhaVulg1_0:8:43437752:43438483:1 gene:PHAVU_008G1678001g transcript:ESW13098 gene_biotype:protein_coding transcript_biotype:protein_coding description:hypothetical protein

METQGEEHASKLFRAQTHIWNHMFKFINSLSLRCAIDLSIPNIIHNYGQPMPLSKLISSLPLHPSKTCFISRLMRILTHSGFFSEHHATQNEPEVMYVLTDASKLLLKDQPSSMASLLQLIVDPVYINTWYQLSTWFTNEDPTPFHAENGMTFWDFARCKREFNNLFNDAMASDSHWVSSVVIEKCEGVFNGSKSFVDVGGGTGTMAKAIAKSFPQLNCIVLDLPHVVADLQETENIKICWRGY

>ESW13097 pep chromosome:PhaVulg1_0:8:43437741:43438523:1 gene:PHAVU_008G1678001g transcript:ESW13097 gene_biotype:protein_coding transcript_biotype:protein_coding description:hypothetical protein

METQGEEHASKLFRAQTHIWNHMFKFINSLSLRCAIDLSIPNIIHNYGQPMPLSKLISSLPLHPSKTCFISRLMRILTHSGFFSEHHATQNEPEVMYVLTDASKLLLKDQPSSMASLLQLIVDPVYINTWYQLSTWFTNEDPTPFHAENGMTFWDFARCKREFNNLFNDAMASDSHWVSSVVIEKCEGVFNGSKSFVDVGGGTGTMAKAIAKSFPQLNCIVLDLPHVVADLQETENIKICWRGYV

>ESW11471 pep chromosome:PhaVulg1_0:8:2707004:2717150:1 gene:PHAVU_008G032800g transcript:ESW11471 gene_biotype:protein_coding transcript_biotype:protein_coding description:hypothetical protein

MVSNDGSKASEIFRGQVHLYKHLFAHTIDAMSLKWMVELSIPDIIHNHGQPITFQKLVSILQVSPTKVRGVQSLIHYLAHTGFFEIVSVHENMEEKEAYALTAASQLLVKDSDLCLAPTVEGFVDPGLTGVWSNLKKWTYEDDLTLFGVSVGSNLWEFLDKNPASDEAFNETLAADSKMMNMALKGCNWVFEGVESIVDVGGGTGITAKILCEAFPNMKCIVLERPGVIENLSGTNNLTYVGGDMFKSIPKADAVLLKSQVAFAHSLYFVAKLFLLFASFCTSHHILTVSSSSQSHPSPRPPHSQMALQTATPPTTPSAQVVGNAFVEQYYHILHHSPDLVYRFYQDSSVISRPDSNGVMTSVTTMKGINEKIVSLNFKEFKAEIKTADAQKSYQEGVTVLVTGCLTGKDNMRRKFAQSFFLAPQDNGYFVLNDVFRYVEDDEPSELPPVNGDGDAAAVKIIPEPESSHVADSPAPDSTNSIVNKGQIVVEKAYAPSNHHERQIPAENEDNVESHFQSNGNDDSQATEVDSLAQEDAPKQSYASIVKVQKGSSVPTKVYVPTNTLKSGSNKTESLVAEPVESSEVPEAASDSVNDPESSDAHEEVEGHSIYIRNLPLNVTVGQLEVEFKKFGSIKPDGIQVRNNKQQGYCFGFVEFLSLNSMNSAIQASPIPIGGRQAVVEIKRTTTRVGSGISNTGRPRIPPGRGGLRNDSFRGRGNYGGGRGYGRNDYGNRGGEFSGRGRSHGDGEFSGRGRSHGDGEFSGRGRSHGESYHQGRGRGGRSSGPKQNAVVSN

>ESW11472 pep chromosome:PhaVulg1_0:8:2707004:2717206:1 gene:PHAVU_008G032800g transcript:ESW11472 gene_biotype:protein_coding transcript_biotype:protein_coding description:hypothetical protein

MVSNDGSKASEIFRGQVHLYKHLFAHTIDAMSLKWMVELSIPDIIHNHGQPITFQKLVSILQVSPTKVRGVQSLIHYLAHTGFFEIVSVHENMEEKEAYALTAASQLLVKDSDLCLAPTVEGFVDPGLTGVWSNLKKWTYEDDLTLFGVSVGSNLWEFLDKNPASDEAFNETLAADSKMMNMALKGCNWVFEGVESIVDVGGGTGITAKILCEAFPNMKCIVLERPGVIENLSGTNNLTYVGGDMFKSIPKADAVLLKSQVAFAHSLYFVAKLFLLFASFCTSHHILTVSSSSQSHPSPRPPHSQMALQTATPPTTPSAQVVGNAFVEQYYHILHHSPDLVYRFYQDSSVISRPDSNGVMTSVTTMKGINEKIVSLNFKEFKAEIKTADAQKSYQEGVTVLVTGCLTGKDNMRRKFAQSFFLAPQDNGYFVLNDVFRYVEDDEPSELPPVNGDGDAAAVKIIPEPESSHVADSPAPDSTNSIVNKGQIVVEKAYAPSNHHERQIPAENEDNVESHFQSNGNDDSQATEVDSLAQEDAPKQSYASIVKVQKGSSVPTKVYVPTNTLKSGSNKTESLVAEPVESSEVPEAASDSVNDPESSDAHEEVEGHSIYIRNLPLNVTVGQLEVEFKKFGSIKPDGIQVRNNKQQGYCFGFVEFLSLNSMNSAIQASPIPIGGRQAVVEIKRTTTRVGSGISNTGRPRIPPGRGGLRNDSFRGRGNYGGGRGYGRNDYGNRGGEFSGRGRSHGDGEFSGRGRSHGESYHQGRGRGGRSSGPKQNAVVSN

>ESW14070 pep chromosome:PhaVulg1_0:8:56521938:56523290:-1 gene:PHAVU_008G250600g transcript:ESW14070 gene_biotype:protein_coding transcript_biotype:protein_coding description:hypothetical protein

MESCGEDQSAKLLRAQTHVWNHILSFINSMSLKCIVDLGIPDIIHNHGQPMSLSNLISSLPIHSSKTHFIHRLMRIMVQSGFFSQHNPTENELEVKYALTDSSLLLLKSHAMSMTPYLQAKLDPAFTNPWNQFSNWFKNDSPTTFEMAHGKPFWEYAGSDPRFNILFNDAMASDAQLVTSVVIEKCKGVFMGLESLVDVGGGTGTMGKAISKSFPQLECTVFDLPHVVSGLQGSENLKYVGGDMFEAIPPADAILLKWILHDWSDEECVKILKKCKEAISRKGKEGKVIIIDMVVDKEMKDDESFETQLFFDLMMMVLFNGKERNKKEWVKLISSAGFNNYKITPVMGIRSLIEIYP

>ESW11469 pep chromosome:PhaVulg1_0:8:2695482:2698133:1 gene:PHAVU_008G032600g transcript:ESW11469 gene_biotype:protein_coding transcript_biotype:protein_coding description:hypothetical protein

MASNDGSKASEIFRGQVHLYKHLFAHTIDAMSLKWMVELSIPDIIHNHGQPITFQKLVSILQVSPTKVRGVQSLIHYLAHTGFFEIVSVHENMEEKEAYALTAASQLLVKDSDLCLAPTVEGFVDPGLTGVWSNLKKWTYEDDLTLFGVSVGSNLWEFLDKNPASDEAFNETLAADSKMMNMALKGCNWVFEGVESIVDVGGGTGITANTICEAFPNMKCIVLERPHMFKSIPKADAVLLKMVLHNWNDKDCRKILENCKEAISDKGKRGKVIVVDIVMNESEDEEELNELKLLYDVRMTCLINGKERKEEEWKKLFVEAGFQSYKISPFTGYLSLIQIYP

>ESW13099 pep chromosome:PhaVulg1_0:8:43439020:43439316:1 gene:PHAVU_008G1678000g transcript:ESW13099 gene_biotype:protein_coding transcript_biotype:protein_coding description:hypothetical protein

WIMHNWKDEECVKILKNCKEAITNQGRVIIIDMIMENKKEEDELTETQFFFDMQMMMLFGGKERNEKEWANLIFSAGFTDYKIITNTPGILSIIEIYP

>ESW11470 pep chromosome:PhaVulg1_0:8:2701370:2702913:1 gene:PHAVU_008G032700g transcript:ESW11470 gene_biotype:protein_coding transcript_biotype:protein_coding description:hypothetical protein

MASNDGSKASEIFQGQVHLYKHLFAHTIDAMSLKWMIELGIPDIIHNHGQPITLPKLVSILQVSPTKVRGVQSLIHYLTHTGFFERVSILENMEEKEAYALTAASQLLVKDNNLCLVPMVEGFVDPCLAGVWSHLKKWTYEDDLTLFDVSVGSNMWEFLDKNPASNRLFNEIFAADSKMMNMALRGCNWVFEGVESIVDVGGGTGITAKTICEAFPNMKCIVLDRPSVVENLSGTNNLTYVGGDMLKSIPKADAILLKLVLHNWNDKDCRKILENCKEAISDKGSRGKVIVVDVVMNASEDEEELTGLKLLIDVRMRCFINGKERKEEEWKKLFVEAGFQSYKISPFTGYLSLIQIYP

>ESW11557 pep chromosome:PhaVulg1_0:8:3377029:3379244:-1 gene:PHAVU_008G040200g transcript:ESW11557 gene_biotype:protein_coding transcript_biotype:protein_coding description:hypothetical protein

MGSCFSEKENHVVVEREDTETTLSAMVLGSNVVFPAALNAAIELNVFDIIAKEGSAESGGFMSPSEIASKLLIPTQQQHRCELANRLERLLRLLASYSLLAVSSRSDGDGGSVRVYGVSPSGKYFVRDVSGEGYLASFTSFLCHPALSGVWVNFKEAIIDPEIDLFKKVHGMSKFEYFGKYPEINHGISTLVDVGGGTGQCLKMIISKHPSIKGINFDLPHVIENSPPVPGVEHVGGNMFEGVPQGDAIMLKAICHNWSDEKAVELLSNCHKALPPKGKVIVGDFILPEDPEPTNEHKMISILDNIMFITPGGRERTEKEFESLGKSSGFSRFQVVCRAFSTMALMEFYK

>ESW20997 pep chromosome:PhaVulg1_0:5:2973815:2975036:1 gene:PHAVU_005G0323000g transcript:ESW20997 gene_biotype:protein_coding transcript_biotype:protein_coding description:hypothetical protein

SEIASQLPFQHKKLPQRLDRMLSLLASHSLLTCSTRANQDGKLQRLFQLSPSGKYFLNAEATASLAFFSKFMSHPKLVQALFNFKEVLLDCDNGLYMKVHGMPIYEGIQSDPPWNHIFNQAMANICTAEMTKILQMYTGFEGISLLIDVGGGVGQSLNMIISKYPSIKGVNFDLPQVIQQAPPYPGIEHVEGDMFESVPKGDAILLKGILHNWSDENCLRVLNNCHKALPENGKVVVVDFIMPEEIGCTEADKMVTSFDNLMFLDGGSERTEKEFMNLCQSSQFSSFKVVSRAFTVLGVMEFYK

>ESW21431 pep chromosome:PhaVulg1_0:5:11532987:11536578:1 gene:PHAVU_005G070300g transcript:ESW21431 gene_biotype:protein_coding transcript_biotype:protein_coding description:hypothetical protein

MEEESTDSRKQSRLAIMELANMISVPMALNAVVRLNVADAIWNGGANAPLSAAQILPRILPVGDGDAENLQRLLRILTSYGVFHEHLAAGERKYSLTDVGKALVTDEQGLSYGAYVLQHHQDALMRAWTLVHEAVVDPTKEPFERANGEGAYAYYLKQAEMNELMVKAMSGVSVPFMRAMLEGYDGFQGVERLVDVGGSGGDCLRMILLKYPNIKEGINFDLPEVVAKAPPTPCVTHVGGDMFKSIPQGDAIFMKWVLTTWTDEEIKQIMQNCHKALPEGGKLIACEPVLPEHSDESHRTRALLEGDIFVMTIYRAKGKHRTEEQFRQLANDAGFPRFRAFNVDHFYTVLEFQK

>Pp3c21_990|Pp3c21_990V3.1

MPGKVIDSSYLGGMLRLASSVKVLREVATMSEDGSTELRYGLEPIGKFLVGDAEKGSLVHLLLMYQVPVLLSTWNHLPESVVDDSVQPFAIFNKAMAGHSKLYMRAFLDVYEGFEGVRVLIDIGGGFGSAITTITCETTGLLSGVEHTSGDMFESIPTGGDAISLKIRKLGLDAGFMRVDVVCKVDQLSVTEFVKAWLGSGDRSPMFACDWRFLSIPKVSIPLSSTSTGFKARNHYILHLNKAGIA*

>Pp3c5_16200|Pp3c5_16200V3.1

MIARLSIVHLVRFVSSWIVWQTVILDYNVKVFTEFVAFFNLLMAEATARSSDVDHKDCASAAQNGMRVLSPLLFGHAAAMTLKTAVVLNIPDILARAEPERGALSVHEISKELPSDSVDEQVLHRVMRTLVHLKVFSAERVSESGTTVARYGLTPASRRLVQENNSRSLAPLLMYLNYISSHVPWQHLHESVLYGKDAWGTAYGMTSWEYSDVDPEYGALWNAFNKVQGAPTIEALKRYDGFKDVNVLVDVGGYQGATVAAIVAAHPHIRGINFDLPHVIAEAPEFPGVEHVSGHLLEGGVPSGDAMLMKNIVHLFKDEEAVKVLQNCKKALTANGKLLIYDPVIPSEFDEGGSPYDGVPPLMDLGLLVYGKVDRTEEQWRALLASGGFPNASFVCLQPQH*

>Pp3c11_12200|Pp3c11_12200V3.1

MDLEGRAIEELAKAQLEVLGLAVSVVFPAALNAVVKMQVAEVLATAAEEGHRSLSADEIVARMPNRPSQPNSKNLERLLRVLSFKGVFKEEARHDKRRTFRLTPMSSALIRNLPEGTMANYVLLTSLGQEFIESCKHLTAAVLESKVPFAMAHGGKHQFQYCAANPDYSNVFQAAMTDHSHQLVDLMLAKFEGFKDVQRMADVGGGVGTTIGRIVEQYPHIQGINFDLPHVIAHAPQREGVEHIAGDMFESVPPDCDAFFLKNIIHDWDDELNIQILMNCHKALPSRGRVIMVDAVLPATTLLRESSLDDMCAFEADITMMAVSAHGRERDAEEWENLATTSGFTNITFVKLDYMYVIEAMKP*

>Pp3c12_5860|Pp3c12_5860V3.1

MACGEGHDHVGEDVYSTNQQKATTHIDKVRLAAIELVGQAAIPGTLASLARLNVFEALARAGDGVELTPQELGNQAMPGKVINLSYLGRMLRLASSVKVLREVATMSEDGSTEHRYGLEPIGKFLVDDAEKGSLVHLLLMYQDPVFLSTWNHLPESVLDDSVQPFARAHGGLHAWEYGMQNPEFDEKFNKAMAGHSKLYMRAFLDVYQGFEGVRVLIDVGGGFGSAISTITARYPHIKGINFDQPHVIKACPELPGVEHMSGDMFESIPSGGDAIFLKYILHDWDDESCIKLLKNCHKVLPANGKVIAVDSVLTDTINFEGGDRMAFMVDMNMMAFNHSGARERNEGEMRKLGLYAGFLRVDVVCKVDQLSVTEFIKA*

>ONH92375.1 hypothetical protein PRUPE_8G171000 [Prunus persica]

MEDKQRELGGEEKEEEHAKLEIWKYVFGFVEIAVVKCAIQLGIADAIESHGSPMTLLELSSALRCDPSPLYRIMRVLVHLKIFKEKPATQLGPKVYAQTPLSKWLLKSGKNSMAALILLESSPVMLAPWHGLSARIQGVNNSAFEAVHGEDVWSYAAANPNHSKLINEAMACDARVAVPAVLESCLEVFKGLETIVDVGGGDGTTLRLLVEACPWIRGINFDLPHVVSVAQECDRIENVGGDMFDCVPKADAAIIMWVLHDWGDDECIRILKKCREAVPEDKGQVIIVEAVIEEDNEKQDKKLTNVRLMLDMVMMAHTNTGKERTMKEWGYVLGEAGFSRHTITPIHAVQSVIQAFP

>ONH92376.1 hypothetical protein PRUPE_8G171100 [Prunus persica]

MLSTFDSNLFLQITYTPKTLGQTHTHKDMEDKQSRELRSEEEEEEEHAKVDVWKYVFGFVEIAVVKCAIELGIADTIESHGSPMTLLELSSALRCDPSPLYRIMRVLVHLKIFKEKPATQLGPKVFAQTPLSKWLLKSGETSMSALILLESSPVMLAPWHGLSARIQGISNPAFEAVHGEDVWSYAKANPDHSKLINEAMACDARVAVPAVIESCLEVFKGIATIVDVGGGDGTTLRLLVEACPWVQGINFDLPHVVSVAQECDHIKNVGGDMFDSVPKADAVIMKWVLHDWGDDECIRILKKCREAIPENKGKVIIVEAVIDEKDEKEDIKLTNVRLMLDMVMMAHTNTGKERTLKEWGYVLGEAGFSRHTITPIHAVQSVIQAFP

>ONH92379.1 hypothetical protein PRUPE_8G171400 [Prunus persica]

MEDKQREIGGEEKEEEHAKVQIWKYVFGFAEIAVVKCAIQLGIADAIESHGSPMTLLELSSALRCDPSPLYRIMRVLVHLKIFKEKPATQLGPEVYTQTPLSKRLLKSGQNSMAALILLESSPVMLAPWHGLSARIQGNIRNPVFEEVHGEDLWSFGAANPDHNKLFNEAMACDARVHVPAVIESRLEVFKGIETIVDVGGGDGSTMRLLVEACPWIQGINFDLPHVVSVAQECDRIENVGGDMFDCVPKADAVIIKGVLHDWGDDECIRILKKCREAIPEDKGKVIIVEAVIDEKDEKADIKLTNVRLMLDMVMMAHTNTGKERTLKEWGYVLGEAGFSRHTITPIHAVPSVIQAFP

>ONH92381.1 hypothetical protein PRUPE_8G171600 [Prunus persica]

MEVKQRELGGEKEEEEHSKVEIWKYMFGFVELASGRKVYAQTPLCKRLLKSGQNNMAAFILPESSPVMLAPWHGLSARIQGNIRNPVFEEVYGEDLWSFGAANPDHSKLFNEAMACDARVVVPAVIESCINSDLPHVVSVAQEYDRIENVGGDMFDYVPKADVVIIKWVLHDWEDDECIRILKKCREAIPEDKGKVIIIEAVIDEEDEKEDSKLTNVRLMLDMVMMAHTNTGKERTLKEWVYVLDDGP

>ONH92385.1 hypothetical protein PRUPE_8G171900 [Prunus persica]

MTLLELSSALRCDPSPPYRIMRVLVHLKIFKNKPATQLGPKVYAQTPLSKRLLKSGQNSMAAFILLENSPVMLAPWHVLSTRIKGNIRNSVFEEVHGEDIWSFGAANPDHNKLFNEAMACDARVVVSAMTESCIEVFEGIETIVDVGGGDGTTLRLLVEACPWIQGINFDLPHVVPVAQECDRIENVGGDMFDCVPKADAVIIKSVLQDWGDDECIRILKKCREAIAEDKGKVIIVEAVIDEKDEKEDIKLTNVRLMLDMVMMAYTNTGKERTLKEWEYVLEEAGFSRHTITPIHAVCSVIQAFP

>ONH92386.1 hypothetical protein PRUPE_8G172000 [Prunus persica]

MEDKQRELGGEEKEEEHAKVEIWKYVFGFVEIGVVKCAIELGIADAIESHGSPMTLLELSSALRCDPSPLYRIMRVLVHLKIFKEKPATQLGPKVYAQTPLSKQLLKSGENSMAALILLGNSPVVLAPWHGLSARIQGISNPAFEAERGEDIWSYAAANPDHSKLFNEAMACDARVAVPAVIESCSEVFKGIEKIVDVGGGDGTTLRLLIEACPWIRGINFDLPHVVSVAQECDRIENVGGDMFDFVPKADAVIMKWVLHNWGDDECIRILKKCREAIPADKGKVIIVEAVIDEKDEKEDIKLTNVRLMLDMAMMAATQIGKERTLKEWGYVLADAGFSQHTITPIHAVQSVIQAFP

>ONH96362.1 hypothetical protein PRUPE_7G123600 [Prunus persica]

MENHVSHVTTTAEQPSNEARLGILELANMISVPMSLNAVVRLNVPDAIWQGGSNSPLSASAILSHVLPDGGGDAENLQRILRMLTSYGVFAEHLVSADDDGGSHQRKFSLTEIGKTLVTDQNGLSYGPYILQHHQDVLMGAWPMVHEAVVDPTIEPFVKVNGEPAYEYYGKKPEMNGLMQRAMSGVSVPFMKAILDGYDGFEGVGRLVDVGGSAGDCLRMILQKHPSVREGINFDLPEVVAKAPTIAGVSHVGGDMFKSIPSGDAIFMKWVLSTWTDSECKLIMENCYKALPVGGKLIACEPVLPTKSDDSPRTRALLENDIFVMTIYRAKGKNRTEDELRQLGLSAGFSHFKPIYIDYFYTVLEFQK

>ONI07667.1 hypothetical protein PRUPE_5G134400 [Prunus persica]

MKEKQVTKMATNEEEEIHSQYAMQLASASLVPMVLKAAIELGVFDILHRAGPGALLSATQIASLLPSNNNPGADLVLDRMLRLLSAYSVLACSVTSHQTDGEALRLYGLSPVSKYFIKNQDGVSLAPLLYLCNDKVTIDSWFHLKDAVLEGGLPFSQAYGMNVVEYVGKDERFRGVFKDSMKEFNPIFMKKILETYKGFVGLKTLVDVGGGDGTILNMIISMYPAIKGFNYDLPSVVEKSPSHPGIEHIAGDMFVRIPKGDAVFMKWMLHGWDDKHCLMILKNCYEALPDHGKVILVDMVVPEAPETSLSASSLFQFDVYLMNTNAMGKERTEKELESLAKEAGFSNIRVACSAFTFSVVELFKNV

>ONI07668.1 hypothetical protein PRUPE_5G134400 [Prunus persica]

MKEKQVTKMATNEEEEIHSQYAMQLASASLVPMVLKAAIELGVFDILHRAGPGALLSATQIASLLPSNNNPGADLVLDRMLRLLSAYSVLACSVTSHQTDGEALRLYGLSPVSKYFIKNQDGVSLAPLLYLCNDKVTIDSWFHLKDAVLEGGLPFSQAYGMNVVEYVGKDERFRGVFKDSMKEFNPIFMKKILETYKGFVGLKTLVDVGGGDGTILNMIISMYPAIKGFNYDLPSVVEKSPSHPGIEHIAGDMFVRIPKGDAVFMKVSGCYMAGMTNIA

>ONI10568.1 hypothetical protein PRUPE_4G054200 [Prunus persica]

MDGDEARDLFQAQSHLYKHIFNFISSMSLKCAVQLGIPDIINSHGQPITLPDLVTALQIHPARTGHVHRLMRLMVRSGFFAIKQVRNNQEEEEEEEEEAYDLTPSSRLLLKDKVPSLSPFVLAMLDPALATPWQFLGNWFRGNELTPFESAHGMGFWEYGDQNPEFNSLFNEAMTSDSGMMNLVIKDCKPIFEGLSSLVDVGGGTGKVARILCEAFPHLKCTVLELPQVVANLTDTENLKFIGGDMFQAIPPADAILLKLTLHALSDEECLKVLKKCREAIPGNGQGKVIIIDIVIDDTKDEHEITEAKLFFDLLMMVVVTGRERSEKDWKNLFLEAGFSNYKLTPIFGLRYLHLPHTTVIGFENNDKRAWVERIMQVDKRDIGTALNVISSNISAATFLCSISLTLSSLIGAWLGSSSSNEVFTSELIYGNVSPSILTIKYITLLTCFLLAFACFVQSARHFVHANYLISTPDSNIPAWYVELAVIRGGDFWSLGLRALYFALTLLLWFFGPIPMFLSSIVMVILLHYMDKNTRPLHDHQLPGRQLVKNVGQRITEVAVNIHQHTEAVEATV

>ONI11083.1 hypothetical protein PRUPE_4G086200 [Prunus persica]

MENTRSLENIVSARGLINFIPIQMTLKAAIELNVFSIIAKSGPASHLTAKEIASQIPTSNPNAAGNLERILRLLAAHSLLSTTLNPCPNDETLQERAYGLTNETLCLVPDENGVSLAPFIILNSELEIVKSLYMLKHTVLEPDFLPFCKAHGITIYEYMSKKPEMSQLFNKSMAETSNLNFSEVLKVYKGFEEVKELMDVGGGIGTSMSEVVSMYPHIHGINFDLPNVVAQAPTYQGVNHVGGNMFETIPNAQSIMLKWVLHNWGDDQCKKVLRNCWEALPKSGKVIVVEFAIPEELEKTKAVLNIVTLDITMMACPGGKERTTTEFANLAKHVGFVETKFFPISYGIYVLEFLKIEEA

>ONI13891.1 hypothetical protein PRUPE_4G252900 [Prunus persica]

MDLSNEMSSANLLQAQAHIWNCIFSFINPLSLKCAVQLGIPDIIKKHGNPMSLSELISALPIHPTKSNCVYRLMRILVHSGFFGRQKLSELDEEEGYVLTDASRLLLKDDPLSARPFLLGQLDPFMTKPWHYFSTWFQNNDPTACFTAHGTTFWDFGYLEPSLSHIFNDSMASDARLISKVVSNEYKGVFEGLESLVDVGGGIGTMAKAIADVFPHVECIVFDLPHVVADLKGSKNLKFFGGDMFEAIPHTDAILMKWILHDWSDEECIKILQRSKEAITRKEKKGKVIIIDMVMKQKGDDQSIETQLFFDMLMMVLVTGKERTEKEWAKLFSDAGFSDYKITPICGLRYLIEVYP

>ONI13894.1 hypothetical protein PRUPE_4G253200 [Prunus persica]

MDLSNEMSSANLLQAQAHIWNCIFSFINPLSLKCAVQLGIPDIIKKHGNPMSLSELISALPIHPTKSNCVYRLMRILVHSGFFGRQKLSELDEEEGYVLTDASRLLLKDDPLSARPFLLGQLDPFMTKPWHYFSTWFQNNDPTACFTAHGTTFWDLGYLEPSLSHIFNDSMASDARLISKVVSNEYKGVFEGLESLVDVGGGIGTMAKAIADVFPHVECIVFDLPHVVADLKGSKNLKFFGGDMFEAIPHTDAILMKWILHDWSDEECIKILQRSKEAITRKEKKGKVIIIDMVMKQKGDDQSIETQLFFDMLMMVLVTGKERTEKEWAKLFSDAGFSDYKITPICGLRYLIEVYP

>ONI13895.1 hypothetical protein PRUPE_4G253300 [Prunus persica]

MDLSNEMSSANLLQAQAHIWNCIFSFINPLSLKCAVQLGIPDIIKKHGNPMSLSELISALPIHPTKSNCVYRLMRILVHSGFFGRQKLSELDEEEGYVLTDASRLLLKDDPLSARPFLLGQLDPFMTKPWHYFSTWFQNNDPTACFTAHGTTFWDLGYLEPSLSHIFNDSMASDARLISKVVSNEYKGVFEGLESLVDVGGGIGTMAKAIADVFPHVECIVFDLPHVVADLKGSKNLKFFGGDMFEAIPHTDAILMKWILHDWSDEECIKILQRSKEAITRKEKKGKVIIIDMVMKQKGDDQSIETQLFFDMLMMVLVTGKERTEKEWAKLFSDAGFSDYKITPICGLRYLIEVYP

>ONI16744.1 hypothetical protein PRUPE_3G118900 [Prunus persica]

MSLKCEVQLGIPDVIHNHGQPISLSNLIFGHNVHPSKAHFIACLMRILVHSNFFAQDQQVQLPLRPNNNNSNENIVHQDLDDEAEEKTVVAYSLTPASGLLLKEGTLSTTQFLLMILDPVVTDPFHLMGTWCQMNNHGNLDHPASPFEMAHGRPFWGLAAQQPKFARAVVEECEGVFEGLNSLVDVGGGTGTTAKAIAKAFPNINCTVFDQPHVVANLQGTHNLDFVGGDMFEKIPPANAIFLKWIMHDWSDEERVKILKKSREAILSKNEVGKVIILDINVSADNTKMDKKSIETQLINIMMMVDLSGKKRSEAEWEKLFLTAGFSHYKITHTLSLRSLIEVYPRKKESLF

>ONI16746.1 hypothetical protein PRUPE_3G119100 [Prunus persica]

MGSEVRATHELLQAQAHIWNHIFSFINSMSLKCAVQLDIPDVIQKHGQPMTLSELVSALPISPTKAHFIPRLMRILVHSGFFARESLNGGEQGYVLTEASALLLKDNPISARPFLLAMLNPILTDPWQYLTPWFQNDNPTPFHVVHGMTFWDYGSQDPTLSHFFNDAMPSDARLISSLMIDDCKGVFQGVESLVDVGGGTGTVAKSIADAFPYMKCTVLDLPHVVADQKGSKNLEYVAGDMFEAVPAGGAIFLKWILHDWSDEECVKILERCKEAITREGKKGKVIIVDMTVENKKTDKESGETQLFFDMQMMVMTTGKERNEKEWAKLFSDAGFSHYKITPCLGLRSLNEVYP

>ONI16752.1 hypothetical protein PRUPE_3G119700 [Prunus persica]

MRATHELLQAQAHIWNHIFSFINSMSLKCAVQLDIPDVIQKHGQPMTLSELVSALPISPTKAHFIPRLMRILVHSGFFARESLNGGEQGYVLTDASALLLKDNPMSARPFLLDMLNPILTDPWQYLTTWFQNDNPTPFHVGVFQGVDSLVDVGGGTGTVAKSIADAFPHMKCTALDLPHVVADLKGSKNLEYVAGNMFEAVPAADAIFLKWILHDWSDEECVKILKRCKEAVTREGKKGKVIIVDMKVENKNTNKESGETQLFFDMLMMVMATGKERNEKEWAKLFSDAGFSHYKITPCLSLRSLIEVYP

>ONI16753.1 hypothetical protein PRUPE_3G119800 [Prunus persica]

MNNHGNHDHPASPFEMAHGRPFWGLAAQQPKFGSLFNEAMEADSQLIARAVVEECEGVFEGLNSLVDVGGGTGNMAKAIAKAFPNINCTVLDQPHVVANLQGTHNLDFVGGDMFDKIPPANAIFLKWILHDWSDEESVKILKKSKEAILSKNEGGKVIILEINVSPDNKKMDKKSIETQLMWDMLMMVNLNGKERSEAEWEKLFLTVGFSLYKITHTLGLRSLIEVYP

>ONI16758.1 hypothetical protein PRUPE_3G120200 [Prunus persica]

MGGGTGTVAKSIADAFPHMKCTVLDLPHVVADLKGSKNLEYVAGNMFEAVPAADAIFLKWILHDWSDEECVKILERCKEAITREGKKSKVIIVDMTVENKNTDKESGETQLFFDMLMMVMATGKERNDKEWAKLFSDAGFSHYKITPCLGLRSLIEVYP

>ONI16760.1 hypothetical protein PRUPE_3G120400 [Prunus persica]

MSLSNDKVSATSHELLQAQAHVWNHIFQFINSMSLKCAVQLGIPDVIHNHGQPISLSNLISGLNVHPSKAHFIARLMRILVHSNFFAQDQQVQLPLRPNNNNNNENIVHQDLDDEAEEKTVVVYSLTPASRLLLKEGTLSTRQFLLMILDPVMTDPFHLMGTWCQMNNHGNLDHPASPFEMAHGTPFWGLVAQQPKFGSLFNEAMEADSQLIARAVVEECEGVFEGLNSLVDVGGGTGTMAKAIAKAFPNINCTVFDQPHVVANLQGTHNLDFVGGDMFEKIPPANAIFLKWILHDWSDEESVKILKKSREAILSKNEGGKVIILDINVSADNTKMDKKSIETQLMWDMLMMVELSGKERSEAEWEKLFLTAGFSHYKITHTLGLRSLIEVYP

>ONI16892.1 hypothetical protein PRUPE_3G127600 [Prunus persica]

MDSEVRASHELLQAQAHIWNHIFSFINSMSLKCAVQLGIPDVIQKHGQPMTLSELVSALPISPTKAHFIPRLMRILVHSGFFARESLSGGGEQGYILTDASALLLKDNPMSARPFLLAMLSPILTDPWQYLTTWFQNDNPTPFHVVHGMTCWEYGNQDPTLAHFFNDAMASDARLISSLVIDDCKEVFQGVNSLVDVGGGTGTMAKSIADAFPHMKCTVLDLPHVVADLKGSKNLEYVAGDMFEAVPAADAIFLKWILHDWSDEECVKILEQCKEAITREGKKGKVIIVDMTVENKNTDKESGETQLFFDMHMMVMTTGKERNEKEWAKLFSDTGFNHYKITPCLGLRSLIEVYP

>ONI16909.1 hypothetical protein PRUPE_3G129000 [Prunus persica]

MGLSNDKVSATSHELLQAQAHVWNHIFQFINSMSLKCAVQLGIPDVIHSHGQPISLSNLISGLNVHPSKAHFIERLMRILVHSNFFAQDQQVQLPLLPNNNNNENIVHQDLDDGEEEKAVVVYSLTPASRLLLKEGPLSTTQFLLMILDPVVTDPFHLMGTWCQMNNHGNHDHPASPFEMAHGRPFWGLAAQQPKFGSLFNEAMEADSQLLARAVVEECEGVFEGLNSLVDVGGGTGTMAKAIAKAFPNINCTVFDQPHVVANLQGTHNLDFVGGDMFEKIPPANAIFLKWILHDWSDEESVKILKKSREAILSKNEGGKVIILDINMSADNKKMDKKSIETQLMFDMLMMVDLNGKERSEAEWEKLFLTAGFSHYKITHTFGLRSLIEVYF

>ONI16910.1 hypothetical protein PRUPE_3G129100 [Prunus persica]

MGSEVRASHELLQAQAHIWNHIFSFINSMSLKCAVQLGIPDVIQKHGQPITLSELVSALPISPTKAHFIPRLMRILVHSGFFAQESLSGGEQGYVLTDASALLLKDNPMSARPFLLAMLNPILTNPWQYLTTWFQNDNPTPFHVDPTLAHFFNDAMASDARLISSLMIDDCKGVFHGVDSLVDVGGGTGTMAKSIADAFPHMKCTVLDLPHVVADLKGSQKLEYVAGDMFEVVPEADAIFLKWILHDFSDEECVKILERCKEAITKEGKKGKESGETQLFFDMLMMVLATGKERNEKEWAKLFSDLGFSHYKITPCLGLRSLIEVYP

>ONI16911.1 hypothetical protein PRUPE_3G129200 [Prunus persica]

MGLSNDKVSATSHDLLQAQAHVWNHIFQFINSMSLKCAVQLGIPDVIHSHGRPISLSNLISGLNVHPSKAHFISRLMRILVHSNFFAQDQQVQLPLLPNNNNNNENIVHQDLDDEEEEKAVVVYSLTPASRLLLEESPLSTTQFLLMILDPVVTDPLHLMGTWCQMNNHGNHDHPASPFEMAHGRPFWGLAAQQPKFGSLFNEAMEADSQLLARAVVEECEGVFEGLNSLVDVGGGTGTMAKAIAKAIAKAFPNINCTVFDQPHVVANLQGTHNLDFVGGDMFEKIPPANAIFLKWILHDWSDEESVKMLKKSREAILSKNEGGKVIILDINVSADNKKMDKKSIETQLMWDMLMMVDLNGKERSEAEWEKLFLTAGFSHYKITHTLA

>ONI16913.1 hypothetical protein PRUPE_3G129400 [Prunus persica]

MGSEVRASHELLQAQAHIWNHIFSFINSMSLKCAVQLDIPDVIQKHGQPMILSELVSALPISPTKAHFIPRLMRILVHSGFFAKESLSGGSEQGYVLTDASALLLKDNPMSTRPFLLAMLNPILTDPWQYLTTWFQNDNPTPFHVVHGMTFWDYGNQDPTLAHFFNDAMASDARLISSLMIDDFKGVFQGVDSLVDVGGGTGTVAKSIANAFPHMKCTVFDLPHVVADLKGSKNMEYVAGDMFEAVPAADAIFLKWILHDWSDEECVKILERCKKAITREGKKGKVIIVDMTVENKNKDKESGETQLFFDMLMMVLVKGKERNDKEWAKLFSDAGFSHYKITPYLGLRSLIEVYP

>ONI16915.1 hypothetical protein PRUPE_3G129600 [Prunus persica]

MCINLNSHHILSQQQGMGSEVRASHELLQAQAHIWNHIFSFINSMSLKCAAQLGIPDVIQKHGQPMTLSELVSALPISPTKAHFIPRLMRILVHSGFFARESLSGGEQGYVLTDASALLLKDNPMSARAFLLAVLNPILTDPWQYLTTWFQNDNPTLFHVVHGMTFWDYGNQDPALAHFFNDAMASDARLISSLVIDEYKGVFQGVDSLVDVGGGTGTMAKSIADAFPHMKCTVLDLPHVVANLKGSKNLEYVAGDMFEAVPAADAIFLKWILHDWSDEECVKILEKCKEAITREGKKGKVIIVDMTVENKNTDKESGETQLFFDMEMMVLATGKERNENEWAKLFFDAGFSHYKITPCLGLRSLIEVYP

>ONI16919.1 hypothetical protein PRUPE_3G129800 [Prunus persica]

MGSEVRASHELLQAQAHIWNHIFSFINSMSLKCAVELDIPDVIQKHGQPMILSELVSALPISPTKAHFIPRLMRILVHSGFFARESLSGGSEQGYVLTDASALLLKDNSMSTRPFLLAMLNPILTDPWQYLTTWFQNDNPTPFHVVHGMTFWDYGNQDPTLAHFFNDAMASDAQLISSLMIDDFKGVFQGVDSLVDVGGGTGTVAKSIADAFPHMKCTVFDLPHVVADLKGSKNLEYVAGDMFEAVPAADAIFLKWILHDWSDEECVKILERCKVAITREGKKGKVIIVDMTVENKNKDKESGETQLFFDMLMMVLVKGKERNEKEWAKLFSDAGFTHYKITPCLGLRSLIEVYP

>ONI18734.1 hypothetical protein PRUPE_3G235300 [Prunus persica]

MAMLEVKEEEAILQGQVDILRYTYNFVESMALKCAVELGIADIINSHGQGQPITLSQIAPQIASPSTDLDLLSRLLRFLVHMNLFEATIDPKTGDALYGLTSSSKWLLSNEEQSLAPLVLMANQPHHLASWHYLSQCIKEGGCGFQKANGFSLYDIPSEKSELGNYFKEGMACTSRIVMKAILSTYKQGFDGVGLIVDVGGGLGTAVAEIVKAHPHIRGINFDLADVVATAPKYPGVIHVGGDMFDVIPKADAIFMKWILHNWNDEKCTQILKNCRKAIPEKIGKLVIVDGVLLPDNGLWDSFVQRYDLTMMVQTINGKERSEVEWKKLLREGGFGRYKIIKIPSFLSIIEAYPE

>ONI18736.1 hypothetical protein PRUPE_3G235500 [Prunus persica]

MEATEAEAMLRGQADIWKYMFGFADSMALKCAVELRIADIIHSHAPTDHMITLSQIASHLIAPSPDITCLTRIMRLLVRRNIFAVHHPSEGGEPVYGLTYSSRWLLHDSEMSLAPMLVMENHPSLMAPWHYFSQCVREGGPWAFKKAHGLEIWDFASENPGFNKLFNDGMACTARIVMKAILTEYGRWFDGVGSLVDVGGGTGSAVADIVQAYPNIKGFNFDLPHVVATAPAYHGVSHVGGDMFEDHIPNADAVFMKWIMHDWNDSDCIKILKNCRKAIPERGGKIMIADIVLEPSGDGVLDDTRLVFDLVMIAHASGGKERTENEWEKILKEGGFPRYKIIKIPALLSIIEAYPV

>ONI23621.1 hypothetical protein PRUPE_2G199000 [Prunus persica]

MAFPLERKIHPKINHAEPEDEITKEEEEESFCYAIQLPERVQAPSSLRLILQPRLAPMMMDRILRLLTSHSVLHSSLAAANEDENGGSDFQRWILHDWTDEHCLKLLKNRYRAIPDDGKQCCGSTSQLDVIMMTEIPGGKERSEQEFMALATGAGFSGIRYECFVCNFWVMEFFK

>ONI23622.1 hypothetical protein PRUPE_2G199100 [Prunus persica]

MASSLERKIHPKINHAEPEDEITKEEEDESFCYAIQLVGSSVLSMSLQSAIELGVFDIIGKEGPGAKLSSSEIAAKIGAKNPEAPMMIDRILRLLTSHSVLHCSLVSANEDENGGGSDFQRVYSLGPVSKYFVNDEEGGSLGPVLTFTQDKVYMESWSQLKDAVVEGGIPFNRVHGTHAFEYPGLDPRFNQAFNTAMFNHTTIVIKKLLHIYKGLEDKNLTQLVDVGGGLGMTLNLITSRYQHIKGINFDLPHVVNHAPSYPGVEHVGGDMFASVPNGDAIFMKWILHDWSDEHCLKLLKNCYKAIPDNGKVIVVEALLPAMPDTSTAVKSTSQLDVMMMIQYPGGKERSEEEFMALATGAGFNGIRYECFVCNFWVMEFYK

>ONI23623.1 hypothetical protein PRUPE_2G199100 [Prunus persica]

MASSLERKIHPKINHAEPEDEITKEEEDESFCYAIQLVGSSVLSMSLQSAIELGVFDIIGKEGPGAKLSSSEIAAKIGAKNPEAPMMIDRILRLLTSHSVLHCSLVSANEDENGGGSDFQRVYSLGPVSKYFVNDEEGGSLGPVLTFTQDKVYMESWSQLKDAVVEGGIPFNRVHGTHAFEYPGLDPRFNQAFNTAMFNHTTIVIKKLLHIYKGLEDKNLTQLVDVGGGLGMTLNLITSRYQHIKGINFDLPHVVNHAPSYPGVEHVGGDMFASVPNGDAIFMKVIFEFLKH

>ONI23625.1 hypothetical protein PRUPE_2G199300 [Prunus persica]

MASSLERKSHPKINHVEPGDEITREEEEESFYYAMQLVGSSVLSISLQSAIELGVFDIIDKEGLGAKLSSSGIAAKIGTKNSEAPMMMDRILRLLTSHSVLHCSLVAANEDENEGGSLGPLMALDQDKVFMKSRSQLKAAVVEGGIPFNKVHGMHAFEYPCLDPRFNQVFNTAMFNHTTIVTKKLLHIYKGLEDKNLTQLVDVGGGLGVWNMFASVPSGDAIFMKWILHDWSDEHCLKLLKNCYKAIPDNGKVIVVEALLPAMPETS

>ONI23626.1 hypothetical protein PRUPE_2G199400 [Prunus persica]

MASSLERKSHPKINHAEPEDEITKEEEDESFCYAIQLVGSSVLSMSLQSAIELGVFDIIAREGPGAKLSSSEIAAKIGTKNPEAPMMVDRILRLLTSHSVLNCSAVAANGGSDFQRVYSLGPVSKYFVNDEEGGSLGPVLTLIQDKVFMESWSQLKDAVVEGGIPFNRVHGTHAFEYPGLDPRFNQAFNTAMFNLTTIVIKKLLRIYKGLEDKNLTQLVDVGGGLGVTLNLITSRYQHIKGVEHVGGDMFASVPSGDAIFMKWILHDWSDEHCLKLLKNCYKAIPENGKVIVVEGLLPAMPDTSTAVKSTSQLDVMMLTQNPGGKERSEQEFMALATGAGFSGIRYECFVCNFWVMEFFK

>ONI23627.1 hypothetical protein PRUPE_2G199400 [Prunus persica]

MASSLERKSHPKINHAEPEDEITKEEEDESFCYAIQLVGSSVLSMSLQSAIELGVFDIIAREGPGAKLSSSEIAAKIGTKNPEAPMMVDRILRLLTSHSVLNCSAVAANGGSDFQRVYSLGPVSKYFVNDEEGGSLGPVLTLIQDKVFMESWSQLKDAVVEGGIPFNRVHGTHAFEYPGLDPRFNQAFNTAMFNLTTIVIKKLLRIYKGLEDKNLTQLVDVGGGLGVTLNLITSRYQHIKGINFDLPHVVNHAPSYPGVEHVGGDMFASVPSGDAIFMKWILHDWSDEHCLKLLKNCYKAIPENGKVIVVEGLLPAMPDTSTAVKSTSQLDVMMLTQNPGGKERSEQEFMALATGAGFSGIRYECFVCNFWVMEFFK

>ONI23628.1 hypothetical protein PRUPE_2G199500 [Prunus persica]

MASSLERKSHPKINHVEPGDEITKEEEEESFCYAMQLVGSSVLSISLQSAIELGVFDIIAKEGPGAKLSLSEIAAKIGTRNSEAPMMMDRILRLLASHSVLHCSLVAANEDENGGSDFQRVYSLGPVSKYFVNDVEGGSLGPLMALDQDKFVLIIILIGSQLKDAVVEGGIPFNRVHGMQTFEFLGLDPRFNQVFNTAMFNHTTIVIKKLLHIYKGFQDKNLTQLIDVGGGFGVTLNLVTSRYPHIRGIYYDLPHVVNHPLPILVWNMFASVPIPSGDAIFLKWILHNRSDEHCLKLLKNCCKAIPDNGKVIVVEELLPAMPDTRDTSTAVKSTSQLDVIMMTQIPGAKERRTRNHGPGNWC

>ONI23629.1 hypothetical protein PRUPE_2G199600 [Prunus persica]

MASSLERKSHPKINHAEPEDEITKEEEDESFCYAMQLVGSSVLSMSLQSAIKLGIFDIIARKGPGAKLSSSEIATKIGTENPEAPVMVDRILRLLTSHSVLNCSAVAANGGSDFQRVYSLGPVSKYFVNDEEGGSLGPLLTLIQDRVFLESWSQLKDAVVEGGIPFNRVHGMHAFEYPGLDPRFNQVFNTAMFNHTTIVIKKLLHIYKGLEDKNLTQLVDVGGGLGVTLNLITSRYQHIKGVEHVGGDMFASVPSGDAIFMKWILHDWSDEHCLKLLKNCYKAIPDNGKVIVVEALLPAMPETSTATKTTSQLDVLMMTQNPGGKERSEQEFMALATGAGFSGIRYECFVCNFWVMEFFK

>ONI23630.1 hypothetical protein PRUPE_2G199600 [Prunus persica]

MASSLERKSHPKINHAEPEDEITKEEEDESFCYAMQLVGSSVLSMSLQSAIKLGIFDIIARKGPGAKLSSSEIATKIGTENPEAPVMVDRILRLLTSHSVLNCSAVAANGGSDFQRVYSLGPVSKYFVNDEEGGSLGPLLTLIQDRVFLESWSQLKDAVVEGGIPFNRVHGMHAFEYPGLDPRFNQVFNTAMFNHTTIVIKKLLHIYKGLEDKNLTQLVDVGGGLGVTLNLITSRYQHIKGINFDLPHVVNHAPSYPGVEHVGGDMFASVPSGDAIFMKWILHDWSDEHCLKLLKNCYKAIPDNGKVIVVEALLPAMPETSTATKTTSQLDVLMMTQNPGGKERSEQEFMALATGAGFSGIRYECFVCNFWVMEFFK

>ONI23632.1 hypothetical protein PRUPE_2G199800 [Prunus persica]

MASSLERKSHPKINHVETEDEITKEEEEEILCYALQLVGSFALSISLQSAIELGVFDIIAREGPGAKLSSSEIAAKIGTKNSEAPMMMDRILRLLTSHSVLHCSLVAANEDENGASDFQRVYSLGPVSKYFVNDEEGGSLGPMMALTQDKVFTESWSQLKDAVVEGGIPFNRVHGMQTFEFLGLDPRFNQVFNTAMFNHTTIVIKKLLHIYKGFEDKNLTQLVDVGGGFGVTLNLVTSRYPHIRGINYDLPHVVNHAPSYPGVEHVGGDMFASVPSGDAIFVKWILHNWSDEHCLKLLKNCYKAIPDNGKVIVVEELLPAMPDTSTAVKATSQLDMIMMTQIPGAKERSEEEFMALATGAGFSGIRYECFVCNLWVMELFK

>ONI23635.1 hypothetical protein PRUPE_2G200100 [Prunus persica]

MASSLERKSHPKINHAEPEDEITKEEEDESFCYAIQLVGSSVLSMSLQSAIELGVFDIIAREGPGAKLSSSEIAAKIGTKNPEAPMMVDRILRLLTSHSVLNCSAVAANGGSDFQRVYSLGPVSKYFVNEEEGGSLGPVLTLIQDKVFMESWSQLKDAVVEGGIPFNRVHGTHAFEYPGLDPRFNQAFNTAMFNLTTIVIKKLLHIYKGLEDKNVTQLVNLITSRWYQFQLAPCRKSCPFLSW

>ONI23636.1 hypothetical protein PRUPE_2G200100 [Prunus persica]

MASSLERKSHPKINHAEPEDEITKEEEDESFCYAIQLVGSSVLSMSLQSAIELGVFDIIAREGPGAKLSSSEIAAKIGTKNPEAPMMVDRILRLLTSHSVLNCSAVAANGGSDFQRVYSLGPVSKYFVNEEEGGSLGPVLTLIQDKVFMESWSQLKDAVVEGGIPFNRVHGTHAFEYPGLDPRFNQAFNTAMFNLTTIVIKKLLHIYKGLEDKNVTQLVNLITSRWYQFQLAPCRKSCPFLSW

>ONI25770.1 hypothetical protein PRUPE_2G319700 [Prunus persica]

MGSTGETQMTPTQVSDEEANLFAMQLASASVLPMVLKAAIELDLLEIMAKAGPGVFLSPTDIASQLPTKNPDAPVMLDRMLRLLASYSILTYSLRTLADGKVERLYGLGPVCKFLTKNEEGVSIAPLCLMNQDKVLVESWYHLKDAVLEGGIPFNKAYGMTAFEYHGTDPRFNKVFNRGMADHSTITMKKILETYKGFEGLTSVVDVGGGTGAVLNMIVSKYPSIKGINFDLPHVIEDAPQYPGVEHVGGDMFVSVPKGDAIFMKWICHDWSDEHCLKFLKNCYAALPDNGKVILGECILPVAPDSSLATKGVVHIDVIMLAHNPGGKERTEQEFQALAKGAGFQGFNVACSAFNTYVIEFLKKN

>ONI25771.1 hypothetical protein PRUPE_2G319700 [Prunus persica]

MGSTGETQMTPTQVSDEEANLFAMQLASASVLPMVLKAAIELDLLEIMAKAGPGVFLSPTDIASQLPTKNPDAPVMLDRMLRLLASYSILTYSLRTLADGKVERLYGLGPVCKFLTKNEEGVSIAPLCLMNQDKVLVESWYHLKDAVLEGGIPFNKAYGMTAFEYHGTDPRFNKVFNRGMADHSTITMKKILETYKGFEGLTSVVDVGGGTGAVLNMIVSKYPSIKGINFDLPHVIEDAPQYPGINHVGGDMFVSVPKGDAIFMKWICHDWSDEHCLKFLKNCYAALPDNGKVILGECILPVAPDSSLATKGVVHIDVIMLAHNPGGKERTEQEFQALAKGAGFQGFNVACSAFNTYVIEFLKKN

>ONI34719.1 hypothetical protein PRUPE_1G494500 [Prunus persica]

MSSRISEKTNANPMSQQQEEEKEEEEVGKLAIRLANVVILPMVLKSAIELNIIDIISAAGDGKFLSPSEIAASLPTKNPDASVLLDRMLRLLTSHSILKCSVRTGQDGKVERLYGAGALCKFLVKDQHDGVGSVGPLLLLHHDKVFMESWCHFNDAVLEGGIPFNRAYGMTAFEYPETDERFNRVFNQAMSNHTTLILKKIFDVYKGFEGLNVLVDVGGGIGVTLNLITTKYPRIKGINFDLPHVLADAPSYPGVEHVGGDMFVSVPQGDAIFMKWILHDWSDEHCLTLLKNCCKALPSSGKVIIVESILPDIPDSSVTSNIVCEQDLLMLTVNPGGKERTKHEYEDLAVKSGFSGCEVICNAYNSWIMEFHKNANP

>Solyc01g068550.2.1 (SlASMT1)

MSSSESTSSELLHAQAQIWNYIFNFISSSAVRCAFQLGIPDVLYKHDKPMCLSDISAELSVVNSSKVSFLPILMQFLVQSGFLNQHEDHYSLTPASCLLAKDDPFNVRSLLLLNHGQAFSKAWPELSDWFQNDSPTPFHTAHGKSLWDFIGEEQPSVLGDIFNDALASDSRLNTNVLIAECKHVFEGLTSLVDVGGGTGTVSIAIAKAFPNIKCTVLDLPQVVGDLKGSGNLDFVGGDMFDMIPHTNAILLKCVLHDWNDEDCVKVLKKCKESIPSREKGGKVIIIDTVLEDPKQSNEFVRAQHNMGMLMMVLFAAKERTEKEWEKLFSEAGFTEYKIFPALGLRSLIEIYP

>Solyc02g077510.2.1 (SlASMT2)

MYFHCQTCGVPTLATSLCFYPVSFSLCGCVSGTVEALTELDAIHRLMGLLVYSGFFATTNTILLDESSENQQGYILTPSSKLLLKSEIPNLSPFVRAIVAPVSVNPWQSVGDWFLGNETTPFETPHGAPMWEFFHQNPGFNNVFNEDCKEVFQDMDSLVDVGGDTGIIANTILAAFPHLKCTVLDLPHVVANMPDTHNLKYVGGDMFHSIPSADSILFKHVLHNWSDEDCVKILKRCRGAIKDKNEGRKGKFLIIDMVLDGDDEEANMTEV

>Solyc02g077520.2.1 (SlASMT3)

MDVPMSSEMFEAQAHIYKHAFSFANSMVLGCAIQLGIPDVIHSHKQPMTLSQLVSHLKLPLEKSDAIHRLMRLLVYSGFFATTDFLDENSESQQGYVLTPSSKLLLKSEIPNLSPFARAMIDPVMVNPWQSLGDWFLGNETTPFETAHGAPMWKFCDQNPRFSNVFNEAMASDSQMMCLVVKDCKQVFQEIDSLVDVGGGTGIIANTILAAFPHLKCTVLDLPHVVANMPDTENLKYVGGDMFHSIPSADAILFMHVMHNWSDENCVKILKRCGEAIKDKNEGRKGKVLIIDMVLDRDKEEANMTEVKLIFDVLMMVLVTGRQRTEKEWEKLFLEAGFMSYKITPLFGLRSLIEVFP

>Solyc02g077530.1.1 (SlASMT4)

METNNNVERANELFKAQAHIYKHAFAYANSMALNCAIQLGIPDIIHNHKKPITLPDLLSGLKLPSSKSNAIHRLMRLLVHAQFFDIIKLEENSETEGYVLTTSSRLLLKSEIPNLLPCVRLMVDPVLVTPWQLLGEWFHKNEEATPFETAHGMPMWDFCAQNPIFDTAFNEAMASDSQMMKLVVKDCREVFEGLNSLVDVGGGTGVIAKTILEAIPHLKCTVLDLPHVVANMPQTENLIYVGGNMFQCIPHADAILLKHVMHDWSDEDCVKILKRCREAIEDKDEGRKGKVLIIDMVLGRDEEEANMTEVKLIFDVLMMVVTTGRQRTEKEWEKLFTEAGFMSYKITPLLGLRSLIQVFP

>Solyc03g097700.2.1 (SlASMT5)

MALPNNIGDETNEVLAAQAHIANHVFNYINSMSLKCAIQLGIPDIIHNHGRAMTQSDLVNALPINKSKGHDCIYRLMRILIHAGFFTQGEEGYLLTPTSRLLLKNEPLSLAPFVQAQLDPVLMDPWHSLGEWFANEDSTPFATAHGKPLFEYAGDEQRLNNLFNEAMGCDARLIMSVLIKNGKGVFEGLKSLVDVGGGIGTVAKAISNAFPELKCSVFDLPHVVEGLEGGKNLTYIAGDMFKFVPSADAILLKWILHDWSDKDCVKILKKCKEAIPSKENGGKVIVIDIVIDNQKRDNKSFETQLFSDVLMMVHVSGKERNEQEWAKLFSNAGFSDYKISPILGLRSVIEVYP

>Solyc06g060200.1.1 (SlASMT6)

MMDHTYSGVFNKAMFNHTTLMMKKILDKYKGFENIKTLVDVGGGLASNLKMITTKYPTIKGTNFDVPHVVQHAPTYKGVGHVGGDMFESVPEGHAIFMKKWILYDWSDSQCVKLLKNCYKATPAEN

>Solyc06g064500.2.1 (SlASMT7)

MASNNNICAYELIEAEAQSWDYILSYLRPSCIKCAIQLGIPDILHKNADPIMSLSDLIAALPNLNPSKTTFIPILMRVLVDFGLFNYHQQQGDGYSLTTVGRLLVENHHFGNRSFFLFAQHPVVLNTAASVGDWLKDDLRTAFETADGKSHWDYCGADPEFNGVFNDAMAGDSRLMSNLLISDCCAGVFEGLTSLVDIGGGTGAVAMAIAGAFPSLKCIVLDLPHVIADRKGSGNLEFVAGSMFDKIPHANAILLKWILHNWDDEDCVKLLKKCKESISSRENGGKVIIIDMIMEDNYNNKQLVQSQHLMDLIMRITYASKERTEKEWEKLFLEAGFSGYKIITSLGLRSLIEIYP

>Solyc06g064510.2.1 (SlASMT8)

MANKKNNMYANELIEAEALSWDYILTYLRPPCIRCAIQLGIPDILHKSAHPIMSLSDLIAALPNVNPSKTTFIPILMRVLVDLGLFNYHPQQGDGYSLTSVGRLFVESDPSNKRSIFTFLIQFEIVDSMSDWLRNDLPTAFETAHGKSIWDYCSGESEYSGVFNDAMASDSKLISNLLISDCCSGVFEGLTSLVDVGGGTGTVAMAIAGAFPSLKCTVLDLPHAIGDRKGTQNLEFVAGNMFDKIPHANAILLKWILHDWNDEDCVKILKKCKESIPSKENGGKVIIIDIVMEDNCSNNEQLVQSQHLMDLLVRITYDSKERSNKEWEKLFLDAGFSGYKIITSLGLRSLMEVYP

>Solyc10g008120.2.1 (SlASMT9)

MVVPIVGEHETEILHAQTHIWNHIFSFINSMSLKSAIQLNIPDIIHKHGKPMTLDELANALSINHSKITHLRRLMRILVHSGFFLKSVSGSGSESGSGEGGYVLAPPALLLLKDEPLTVTPFLLAMLDPILVKPWHHVSEWFTSDEPTAFEVAHGRTFWDYAGHEPRLNHFFNDAMASDAKLVMSVVMKYSKDVFEGLNSIVDVGGGTGTVAKTIAKTFPNLQCTVFDLPHVVEGLEGSDNLTYVGGDFFVSIPHAEALLLKWILHDWSDEESVKILKKCKEAIPSKEKGGKVIIIDMMVDNKIGDDESIETQIFFDMLMMVLVTGRERSERGWAKIFSEAGFSDYKVTPILGLRSLIEVYP

>Solyc10g079540.1.1 (SlASMT10)

MADHNENLTPSELLQAETQSWNQLYFFIEHVTLKCALQLDIPNVITKHDKPMTISELMSSLPISPSKYPYFHRLTRILVHYGFLILQKHDDNNVDDDKGCYSLAPADCYVVKDGPWNSMEDQDTFFFKAWSCLGDWFKNDDPSAFYTAYGDLFWSKLSSDSSTSNWFNENMSRDSRSFMNVLIGNEYKDVFKGLTSLVDVGGGTGTVAMSIAKSFPDMKCIVLDLPPVVANLQGSENLEFVAGDMFQKIPPANAVLLKSILHDWNDEECVKILKNCKEALRGSGKVIIIDMVMENTELDDESVQAQLFIDMLMMVFVGSKERNEKEWEKLFSISGFTSYKIVLTLGLRSVIELYP

>Solyc12g041960.1.1 (SlASMT11)

MALPNDNTREILAAQAHIWNHTFSYINSMSLKCAIQLGIPDIIHSHGRAMTLSDLVNALPINNNVKTLDYIFRLMRILIHGGFFNKIKVNDKEEGYLLTPASCLLLKDEPLSQVAFVQTELDQSFMDPWHSLIKWIRSDHDNSSTPFAISHGKPLFEYDETQPNINRQFNEIMASDSRLVISVLIKNCKGVFEGLKSLVDVGGGIGIVGKVLADAFREMNCIVFDLPHVIEGCEGSKNLCYVGGDMFKFIPSANAILLKWVLHDWSDEECIKILKKCKKAIPSKEKGGKVIIIDMVLMDRKIEKGDGKSYETQLFFDMLMMVHVSGKERNQQDWAKLFCSAGFSDYNIIPMLGLRSIIEVFP

>Sphmag15G072100|Sphmag15G072100.1

MAADGSSDHRAQAVEEDHHETPLRNGIVVGEQDTAAARHAAMRLATMMVVPAALKAAIELGVFEILAKARGAAPRAKSLTAKEIASQVIQPDSDGLSVINDRYLERILRLLASENVVSESAVAVASGNSSCSTSGMERCYALQPAGTYFVRSDEDAASLAPLLVQLQDRDFLEPWHHLSATVMDDSTEPFTKAHGEPVFQYTSHNPRLDKIFNTSMAHHSRLYTQAVLQVYHGFQDVKCLVDVGGGTGSSLALIAAKYPHIRGINYDLPHVTAASPAHPGVEHVGGDMFKSVPSGDAIFMKWILHDWNDSECVTILRNCFNALATSGGKVIAVESVLPDLVLQSELGAGPTVALRADLLMLACSAVGARERTLQEFRELGKTAGFASVSVVVSIDFLSVLEFRKA*

>Sphmag02G186300|Sphmag02G186300.1

MATNSYRVVHANGTTENLGKQSSSVHQNDVARHAALEFTLYSVVSGTLKAAIELGVFEIAKAGASAGQKSLTAKEIAEQLVRPTANGTAVNSGYLQRILRLLASVNIVSESVAVVAAGPGNFTSYNTHHQRSYALTPIGKYFVQGEDGVSLAPLILMSEDWVFKKAWDHLSAAVLDDVDPFVRAHGKSEFQLNNEDPRVDKLFNTAMSAHSRICMEAILGAYHGFQDVNCLVDVGGTGASLASITAKYPHIRGINFDLPHVVATSPAYPRVEFVGGNMFESIPSGDAIFMKSILHWNDEDCMTILKNCFKALPSRGGKVIVMESVLPDSINLQSEDDGVKSLLALRVDVVMLAYSGGAKERTLHEFQQLADATGFASLALITTIDFLSVLEFTRVAV*

>Sphmag02G187200|Sphmag02G187200.1

MAVNSYTDAQAYGTTVDLHKESSSIHQFDAVRYAAMELTSYCTVPGAMKAAIELGVFEIAKAGAAGKKSLTAKEIAEQVVRPAGNVTVVNHGYLQRVLRLLASVNVVSEHVVVVTATEGNLTSYKTHHRHSYEITPIGKYFVRGEDGISLAAFILMTEDWVFKKPWEHLSAAILDDSDPFVRAHGKNQFQLYNENPRVDKLFNAAMSSHSRLYTEAVLGAYHGFQDVNCLVDVGGGGSSLALITAKYPHIRGINFDLPHVIATSPAYPRVEFLGGNMFESVPSGDAIFMKYILHDNDEECVTILRNCFKALPTSGGKVIIVESVLPNSINLQSEYDGSLLGLRMDLAMLALNSGAKERTLHEFQELADIVGFKSLTLVVTIDFLSVLEFTKATI*

>Sphmag02G179800|Sphmag02G179800.1

MATNSYRVAHANGTTENLGKQSSSFHQNYVARHAALDFALYSVVSGTLKAAIELGVFEIAKAGASASQKSLTAKEIAEQLVRPTANGTAVNSGYLQRLLRVLASVNIVSESVAVVAAGPGNFTSYNTHHQRSYALTPIGKYFVQGEDGFSWAPLVLMSEDWVFKKAWDHLSAAVLDDVDPFVRAHGKSEFQLNNEDPRVNKLFNTAMSSHARIYMEAMLEAYHGFQDVNCLVDVGGTGASLASITAKYPHIRGINFDLPHVVATSPAYPRVEFVGGNMFESIPSGDAIFMKSILHWNDEDCVTILKNCFKALPSSGGKVIVMESVLPDSINLQDEDGGVKSSLALRVDVVMLAYSGGAKERTLHEYQQLADATGFASLALITTVDFLSVLEFTRVAV*

>Sphmag02G014100|Sphmag02G014100.1

MAANSYRVAHSNGTTRNFGKQSSSVHQNDVARHAAMEFTLYSVVSGTLKAAIELGVFEIAKAGASASQKSLTATEIAEQLVRPTANGTAVNSGYLQRFLRLLASVNIVSESVAVVAAGPGNFTSYNTHHQRSYALTPIGKYFVQGEDGVSLAPFILMSEDWVFKKAWDHLSAAVLDDVDPFVRAHGKSEFQLNNEDPRVDKLFNTAMSSHSRIYMEAMLGAYHGFQDVNCLVDVGGTGASLASITAKYPHIRGINFDLPHVVATSPTYPMCMIGVEFVGGNMFESIPSGDAIFMKVLHDWNDEDCMTILKNCFKALPSSGGKVIVMESVLPYSINLQSENNGVKSLLGLRIDLVLAFNSGGAKERTLHEFQQLADATGFASLALITTIDFLSVLEFTRVAT*

>Sphmag05G004500|Sphmag05G004500.1

MAVNGRSDHHAQAADEDHHETPLRNALVDEQDAAARDAAMRLATIMVVPAALKAAVELGFEILAKARGAGKSLTAKEIVSQVLPPDSGSVINYAYLERILRLLASENVVRESAVTVPSNSNSSTERCYALQPVGTYFVRSDEDAGSLAPLLVHLQDPVSLEPWQHLSATVLDDSTDPRRAHGESIFQYINHNPRSEKIFHAAMGHHSRLYMKAVLRAYHGFQDVKCLVDVGGGTGSLALITAKYPHIRGINYDLPHVAAASPAYPGVEHVGGDMFESVPCGDAIFTKWVLQDWNDECLMILRNCFNALPASGGKVIAVNSVLPDLLLQSELGAGSAVALRIDLIMLAHASPGAKRTLQEFRQIAKAAGFASVSVVVAIDFLSVLEFNKA*

>Sphmag05G005000|Sphmag05G005000.1

MAVNSSTDHAQATEEDRHEIPLRNGIHGEQDAAARHAAMRLATTMAVPAAVKAAIELGVEILAKACGPRKSLTAKEIASQVLQPDSAGLSGLNDRYLERILRLLASENVVSESVVTVAGNSSCSNSSSTERCYALQPVGTYFVRGDEDAASLAPLLVMLQDGDFLKPWHHLSATVLDSTDSFRKAHGESIFEYMSHNPRPEKIFNAAMGHHSRLYMQAVLRAYHGFQDVKCLVDVGGTASSLVIITAKYPHIRGINYDLPHVAAASPAYPGVEHVGGDMFESVPSGDAIFMKWILDWNDSECVTILRNCFNALPASGGKVIVVESVLPDLVLQSELGAGPTVALCMDLMMLSYAVGAKERTLHEFGQIAKAAGFTSVSVVATIDFLSVLEFNKA*

>Sphmag05G009900|Sphmag05G009900.1

SVINDRYLERILRLLASENVVRESAVTVASANSSNSTERFYALQPVGTYFVRNDEDAASAPFLNLLQDRDFIEPWHHLSATVLDDSTDAFRRAHGESVFQYTSHNPRIDKIFNAGMAQSRLYMQAVLRAYHGFQDGKCLVDVGGGTGSSFALIAAKYPHIRGINYDLPHVVAASPAYGVEHVGGNMFESVPSGDTIFMKWILHGWDDSECVTISRNCFNALPASGGKVIAVESVLPLLLQSELGAGQTVALRMDLMMLAFAWRGGEFQQLAKTAGFASVSVVASIDFLSVLEFNK*

>Sphmag05G004700|Sphmag05G004700.1

MAVNGRSDHHAQAADEDHHETPLRNALVDEQDAAARDAAMRLATIMVVPAALKAAVELGFEILAKARGAGKSLTAKEIVSQVLPPDSGSVINYAYLERILRLLASENVVRESAVTVPSNSNSSTERCYALQPVGTYFVRSDEDAGSLAPLLVHLQDRVSLEPFQHLSATVLDDSTDPRRAHGESIFQYINHNPRSEKIFHAAMGHHSRLYMKAVLRAYHGFQDVKCLVDVGGGTGSLALITAKYPHIRGINYDLPHVAAASPAYPGVEHVGGDMFESVPSGDAIFTKWVLHDWNDECLMILRNCFNALPASGGKVIAVESVLPDLLRQSELGAGSEVALRLDLIMLAHASPGAKRTLQEFRQIAKAAGFASVSVVVAIDFLSVLEFNKA*

>Sphmag05G010500|Sphmag05G010500.1

MRLATTMAVPAALKAAVDLGVFEILAKARGAGKSLTAKDIASQVLRPDSGGLSVINDRYERILRLLATENVVRESAVTVASANSSSSTERFYALQPVGTYFVRNDEDAASLAPFIILSDRDFIEPWHHLSATVLDDSTDTFRKAHGESLFQHTSHNPRSDKIFNAGMAQHSRLYMQALRAYHDFQDGKCLVDVGGGTGSSLALIAAKYPHIRGINYDLPHVVAASPPYPGVEHVGGMFESVPSGDTIFMQWILHNWDDSECVTILRNCFNALPTSGGKVIALESVLPDLLLQSELAGPTVRLRMELIMLAFNSVGARERTLQEFQELAKTAGLASVSVVVSIGFLSVLEFNKA*

>Sphmag05G004900|Sphmag05G004900.1

MAVNGRSDHHAQAADEDHHETPLRNGLVDEQDAAARDAAMRLATIMVVPAALKAAVELGFEILAKARGAGKSLTAKEIVSQVLPPDSGSVINYAYLERILRLLASENVVRESAVTVPSYFVRSDEDAGSLAPLLVHLQDRVSLEPWQHLSATVLDDSTDPFRRAHGESVFQYINHNPSEKIFHAAMGHHSRLYMKAVLRAYHGFQDVKCLVDVGGGTGSSVALITAKYPHIRGINYLPHVAAASPAYPRVEHVGGDMFESVPCGDAIFTKWVLHDWNDSECLMILRNCFNALPASGKVIAAESVLPDLLLQSELGAGSEVALRLDLIMLAHASPGAKERTLQEFRQIAKAAGFAVSVVVAIDFLSVLEFNKA*

>Sphmag05G011000|Sphmag05G011000.1

MASGRADCWGQGERKENLTMAWERDGSSDHHAQAAQEDHHETPFGNGIVGEQDPAARDAVRLATTIAVPAALKAAVDLGVFEILAKARGAGKSLTTKDIASQVLRPDSGGLSVIIDRYERILRLLASENVVRESAVTVTSANSSSSTERFYALQPVGTYFVRNDEDAASLAPLMILSDRDFIEPWHHLSATFLDDSTDAFRRAHGESVFQWSSLALIANHVLASRHRVDRQLVCDRIRPELQPLITANFQMFHRASGGKVIAVESVLPDLLLQSELGEGPTVALRMDLMMLTFASGARERTLQEFQELAKTAGFASVSVVASIDFLSVLEFNKA*

>Sphmag05G004800|Sphmag05G004800.1

MAVNSSTDYAQATEEDHHEIPLRNGIHGEQDAAARHAAMRLATTMAVPAALKAAIELGVEILAKECGPRKSLTAKEIASQVLQPDSGGLSVLNDRYLERILRLLASENVVSESVVTVAGNSSCSNSSTERCYALQPVGAYFVRGDEDAASLAPLLVMLQDRDFLKPWHHLSATVLDDTESFRKAHGESVFEYMSHNPRLERIFNASMAHHSRLYMQAVLRAYHGFQDVKCLVDVGGKGSSLAIITAKYPHIRGINYDLIHVAAASPAYPGVEHVGGDMFESVPSGDAIFMKWILHWNDSECVTILRNCFNALPASGGKVIVVESVLPDLVLQSELGAGPTVALCMDLMMLSCTSGAKERTLHEFGQIAKAAGFTSVSVAATIDFLSVLEFNKA*

>Sphmag05G010900|Sphmag05G010900.1

MGTDGSSDHHAQAAQEDHHETPFWNGIVGEQDAAARDAAMRLATAIAVPAALKAAVDLGIEILAKARGAGKSLTAKDIASQVLRPDSGGLSVINDRYLERILRLLATENVVRESAVTVSANSSNSTERFYALQPVGTYFVRNDEDAASLAPFLILLQDRDFIEPWHHLSATVLDDSTAFRRAHGESVFQYSSHNPRFDKIFNAGMAQHSRLYMQAVLRAYHGFQDGKCLVDVGGGTSSLALIAAKYPHIRGINYDLPHVVAASPAYPGVEHVAGNMLKSVPSGDTIFMKWILHGWDSECVTILRNCFNALPASGGKVIAVESVLPDLLLQSELGAGQTVALRMDLMMLAFAWRGRERTLQEFQHLAKTAGFASVSVVASIDFLSVLEFNKA*

>Sphmag05G010600|Sphmag05G010600.1

MAVPAALKAAVDLGVFEILAKARGAGKSLTAKDIASQVLRPDSGGLSVINDRYLERILRLATENVVRESAVTVASANSSNSTERFYALQPVGTYFVRNDEDAASLAPFLILLQDRDFIPWHHLSATVLDDSTDAFRRAHGETVLRAYHGFQDGKCLVDVGGGTGSSLALIAAKYPHIGINYDLPHVVAASPAYPGVEHVAGNMLESVPSGDTIFMKWILYGWDDSECTVALRMDLMLAFAWLGGRERTLQEFQELAKTAGFASVSVVASIDFL>Sphmag05G010400|Sphmag05G010400.MRLATIMAVPAALKAAIELGVFEILSKARGAGKISLTAKERLRRNENVVRESAVTVPSGSNSSTERCYALQPVGTYFVRSDVDSASLAPLLVHLHDRDFLEPWHHLSATVLDDSTEPFRAHGESAFQYISHNPRSDKIFNAAMAHHSRLYMQAVLRAYHGFQDVKRLVDVGGGVEHVGDMFDSVPSGDAIFMKWILHDWNDSECVTILRNCFNALPASGGKVNCSGVVAVRIDLIMAHTSAGAKERTLHEFRQIAKAAGFASVSVVVAIDFLSVLEFDKA*

>Sphmag05G016100|Sphmag05G016100.1

MGTDGSSDHHAQAAQEDHHETPSRNGIVGEQDAAARDAAMRLATAMAVPAALKAAIDLGFEILAKARGAGTSLTAKDIASQVLRPDSGGLSVINDRYLERILRLLASENVVRESAVTVSANSSSSTERFYALQPVGTYFVRNDEDAASLAPLIIAWQDRDFIEPWHHLSATVLDDSTPFRKLHGESFFQYTSHNPRLDKIFNAGMAQHSRLYMQAVLRAYHGFQDGKRLVDVGGGTSSLALIAAKYPHIRGINYDLPHVVAASPAYPGVEHVGGNMFESVPSGDTIFMKSILHAWDSECVTILRNCFNALPASGGKVIALESVLPDFLLQSELGAGLTVALRMDLVMLAFASVGRERTLQEFQELAKTAGFANVSVVVSIGFLSVLEFNKA*

>Sphmag05G011100|Sphmag05G011100.1

MGTDGSSDHHAQAAQEDHHETPSRNGIVGEQDAAARDAAMRLATAMAVPAALKAAIDLGFEILAKARGAGTSLTAKDIASQVLRPDSGGLSVINDRYLERILRLLASENVVRESAVTVSANSSSSTERFYALQPVGTYFVRNDEDAASLAPLIIAWQDRDFIEPWHHLSATVLDDSTPFRKLHGESFFQYTSHNPRLDKIFNAGMAQHSRLYMQAVLRAYHGFQDGKRLVDVGGGTSSLALIAAKYPHIRGINYDLPHVVAASPAYPGVEHVGGNMFESVPSGDTIFMKSILHAWDSECVTILRNCFNALPASGGKVIALESVLPDFLLQSELGAGLTVALRMDLVMLAFASVGRERTLQEFQELAKTAGFANVSVVVSIGFLSVLEFNKA*

>Sphmag05G009800|Sphmag05G009800.1

MRTDGSSDHHAQAAQEDHHETPFRSGIVGDQDAAARDAAMRLATTIAVPAALKAAIDLGFEILAKARGVGKSLTAKDIASQVLRPDSGGLPVINDRYLERILRLLASENVVRESAVTVSANSSNSTERFYALQPVGTYFVRNDEDAASLAPFLILSQDRDFIEPWHHLSATVLDDSTTFRKAHGESVFQHTSHNPRSDKIFNAGMAQHSRLYMQAVLRAYHGFQDGKCLVDVGGGTSSLALIAAKYPHIRGINYDLPHVVAASPPYPVDFA*

>Sphmag05G005100|Sphmag05G005100.1

MYAAMRLTMTSPVPAALKAAIELGVFEILAKARGVGKSLTAKEIASQVVQPVSGLSVINGCLERILRLLASENVVSESAVTVASGNSSSYSNRSTERCYALLPVGTYFVRSDEGGASLPLLFLEQDRDFQKGWHYLSATSRLYMQAVLRTYHGFQDVKCLVDVGGGTGSSLALITAKPHIRGINYDLPHVAAASPAYPGVEHIGGNMFESVPSGDAIFMKWILHGWNDSECVTILRCFNALPASGGKVIAVESVLPDFVLHSELDAGMRAALREDLIVMMLNTVGAKERTLQEFRLAMAAGFASVCVVVTVDFLSVLEFNKA*

>Sphmag05G011200|Sphmag05G011200.1

MGTDGSSDHHAQAAQEDHHETPSRNGIVGEQDAAARDAAMRLATTVAVPAALKAAIDLGFEILAKARGAGKSLTAKDIASQVLRPDSGGLSVINDRYLERILRLLATENVVRESAVTPSANSSSTTERFYALQPVGTYFVMNDEDAGSLAPLLIMSQDRDFIEPLHHLSATVLDDSTNFRKAHGESLFQYASHNPGFDKLFNAGMAHQSRLYMQAVLRAYHGFQDGKCLVDVGGGTSSLALIAAKYPHIRGINYDLPHVVAASPPYPGVEHVGGNMFESVPSGDTIFMKIILHNWDSKCVTILRNCFNALPTSGGKVIAVESVLPDFLLQSELGAGPTAKFRMNLIMLAFTSVGRERTLQEFQELAKTAGFASVSVAVSIGFLSVLEFNKA*

>Sphmag09G101500|Sphmag09G101500.1

MAVAAKGSMPPTSESMAPAVSANGGSIWEQQLSEEEMITAQTVALPMMLGFVTPFVMRSVLLGLPDIIAGAGPGKSLTLKQIAAELNTTRSNNNVDGSAAGSTPVNKSNLQRLLNYMVSGILSCSKKVQDDDPQSELVIGAAAADHDSDHLQYGLTAVSNLLVTKNNPSSQAPMLLLTSGASQASWAQLHCSVFGEHPWRTAHGKNVWEYSRDNPEFNRTVNAAMATSAAMLSYGLKYEGFKHIKTLVDVGGGVGKALGILVSSYPQMHGINFDQPHAVADAPNIPGVEHVGGNIESIPSGDAIFIKFILHNWGDDDCIKVLKNCKKALPENGKVLIYELVMHPNGRWAQMMDLMLTQFESGMERTEQQWRTLIAAAGFSSINFIELNHEHWLIEVSN*

>Sphmag14G045600|Sphmag14G045600.1

MATNSYRVVHANGTTENLGKQSSSVHQNDVARHAALEFTLYSVVSGTLKAAIELGVFEIAKAGASAGQKSLTAKEIAEQLVRPTANGTAVNSGYLQRILRLLASVNIVSESVAVVAAGPGNFTSYNTHHQRSYALTPIGKYFVQGEDGVSLAPFILMSEDWVFKKAWDHLSAAVLDDVDPFVRAHGKSEFQLNNEDPRVDKLFNTAMSAHSRIYMEAILGAYHGFQDVNCLVDVGGTGASLASITAKYPHIRGINFDLPHVVATSPAYPRVEFVGGNMFESIPSGDAIFMKSILHWNDEDCMTILKNCFKALPSRGGKVIVMESVLPDSINLQSEDDSVKSLLALRVDLVMLAYSGGAKERTLREFQQLADATGFASLALITTIDFLSVLEFTRVAI*

>Sphmag04G137300|Sphmag04G137300.1

MATDGSSDHHAQAAQEDHHETPSRNGIVGEQDAAARDAAMRLATTMAVPAALKAAIDLGFEILAKARGAGTSLTAKDIASQVLRPDSGGLSVINDRYLERILRLLASENVVRESAVTVSANSSSSTERFYALQPVGTYFVRNDEDAASLAPLVILQQHRDFIEPWHHLSATVLDDSTTFRKAHGESFFQYLSHNPRFDKMFNAGMGQYSRLYMQAVLRAYHGFQDGKRLVDVGGGTSSLALIAAKYPHIRGINYDLPHVVAASPPYPGVEHVGGNMFESVPSGDTIFMKSILHGWDSECVTILRNCFNALPASGGKVIALESVLPDFLLQSELGAGTTAQLRLDLMMLTFASVGRERTLQEFQELAKTAGFANVSVVVSMGVLSVLEFNKA*

>Sphmag04G006900|Sphmag04G006900.1

MAEEVNWSKAQQPVADEEELMANRAVLHPLILGFESSFVLRTAVMLELPDIIARAGPDELTVKQIAAQLKSESVNEFGLQRVLTALVNWKLFRCTKPDLMSEMQYGLTPISKLLVTENPYNQAPAVLFHTDPAILALWQQLHRYVLYGETPFNLHMERIFCGMCSGNLQPFVAGSGCSVPLWIQFLMGILTRHRDIWQCFKDNPELSKCFNACMASATPIDLTVVRKYKGLKDIKTVDVGGGIGKALKTIISSYPHIHGINYDLPHVIADAPTMPGIEHVGGSMFESVPSGDAIFKRVMHDWNDENCLKILNNCQKALPEKGKVLICDLVLQPSGGLPQLMDMVMMVITDGGMETEEQWRKLLTTAGFSTINFIELLQQVWLIEAVK*

>Sphmag12G011200|Sphmag12G011200.1

MAEANGSKTQAQPVADVEELKASRAVLYRLLFGFAGAFVLRTAVVLGLPDIIARAGPDELTVKQIAAQLKSESVNESGLQRVLTALVNFQLLRCTQAKMSERQYGLTPVSKLLVTENNHNQAPAVLFPTDPAALAPWQQLHHYVLYGEDAWQSAHGKDLWTSLNDNPELNKCFNACMSVTAMDFGVERKYEGFKDIKTLVDVGGGTGKTLETIISSYPHIHGINYDLPHVIADAPTPGIEHVAGSMFESVPSGDAIFMKRILHDWSDENCLKILNNCQKALPEKGKVLIRDYVLQSGGVAQMADLFMMAHTNGGMERTEEQWRKLLTTAGFSTVNFIELFEQEWLIEASK*

>Sphmag12G001200|Sphmag12G001200.1

MAANGNGYHAHVGKENHPETPLRNGIVGEQDAARDAAMRLSVAGAVPAALKASIELGVFILAKAGEVGKSLTAKEIASQVVQPVNGLSIINHGYLERILRLLASENVVSESTVTVANGLSNSSNTERCYALQPVGTYFVSGKEDGDSLAPLLFLEQDRDVQEAWRHLSATVLDDTTEFRRAHGMSCFQYVGHNPRFYEVFNSSMAHHSRLYMKAVLRAYHGFQDVKCLVDVGGGTGTLALITAKYPHIRGINFDLPNVALASPAYPGVEHVGGNMFESVPSGDAVFMKWVLDNWNSECVTILRNCFNALPASGGKVIAAESVLPELVQSEVGVGTQAALRIDVSAMMFNAVGAKRTLHEFQKLAKAAGFASVSVVATIDFLSVLEFKKV*

>Sphmag12G011300|Sphmag12G011300.1

MSERQYGLTPVSKLLVTENNPHNQTPVELFPTDPAALAPWQQLHHYVLYGEVAWQSAHGDLWTSLNDNPELNKRFNACMASATTIDLSVVRKYEGFKDIKTLVDVGGGTGKTLETIISYPHIHGINYDLPHVIADAPTIPGIEHVAGSMFESVPSGDAIIVKRIMHDWSDENCLKILNCHKALPEKGKVLIHDFVLQPSGGLPQIVDMLTMAHTIGGMERTEEQWRKLLTTAGFSTNFIELFEQEWLIAASK*

>Sphmag12G011800|Sphmag12G011800.1

MAEANGSKTQAHPVADVEEFTARRAVLYRLLFGFAGSFVLRTAVVLGLPDIIARAGPDELTVKQIAAQLKSESVNESGLQRVLTALVNFQLLRCTEGEMSERQYGLTPVSKLLVTENNHNQTPVVLFPTDPAALAPWHQLHHYVLYGEDAWQSAHGKDLWTSLNDNPELNKCFNDSMSATTMDFGVERKYEGFKDIKTLVDVGGGTGKTLENIISSYPHIHGINYDLPRVIADAPTPGIEHVAGSMFESVPSGDAIFMKRIMHDWSDEHCLKILNNCQKALPEKGKVLIHDFVLQSGGLPQIVDMMMMAHTGGMERTEEQWRKLLTTAGFSTINFIELFEQKWLIEASK*

>Sphmag12G011500|Sphmag12G011500.1

MAEANGSKTQAQPVADVEELRANRAVLYGLVFGFAGSFVLRTAVVLGLPDIIARAGPDELTVKQIAAQLKSESVNEFGLQRVLTALVNFQLLRCTEAEMSERQYGLTPVSKLLVTENNHNQAPVVLFMTDPAALAPWHQLHHYVLYGEDAWQSAHGKDLWTSLNDNPELNKCYNACMSATTVDLGVVRKYEGFKDIKTLVDVGGGTGKTLETIISSYPHIHGINYDLPRVIADAPTPGIEHVAGSMFESVPSGDAIFMKRIMHDWSDEHCLKILNNCQKALPEKGKVLIHDFVLQSGGLSQIVDMLMMAHTNGGMERTEEQWRKLLTTAGFSTINFIELLEQEWLIEASK*

>Sphmag12G011100|Sphmag12G011100.1

MAEANGSKTQAHPVADVEELTARRAVLYRLLFGFAGSFVLRTAVVLGLPDIIARAGPDELTVKQIAAQLKSESVNESGLQRVLTALVNFQLLRCTEAEMSERQYGLTPVSKLLITENNHNQTPVVLFETDPAALAPWGQLHHYVLYGEDAWQSAHGKDLWTSLNDNPELNKCFNACMSATTMDFGVERKYEGFKDIKTLVDVGGGTGKTLENIISSYPHIHGINYDLPHVIADAPTPGIEHVAGSMFESVPSGDAIFMKRIMHDWSDENCLKILNNCRKALPEKGKVLIHDFVLQSGGLPQIVDMLMMAHTNGMERTEEQWRKLLTTAGFSTINFIELFQQKWLIEASK*

>Sphmag12G011700|Sphmag12G011700.1

MAEANGSKTQAQPVADVEELTANRAVLYRLLFGFAGSFVLRTAVVLGLPDIIARAGPDELTVKQIAAQLKSESVNESGLQRVLTALVNFQLLRCTEGEMSERQYGLTPVSKLLVTENNHNQTPVVLFPTDPAALAPWHQLHHYVLYGEDAWQSAHGKDLWTSLNDNPELNKCFNDSMSATTMDFGVERKYEGFKDIKTLVDVGGGTGKTLENIISSYPHIHGINYDLPRVIADAPTPGIEHVAGSMFESVPSGDAIFMKRIMHDWSDEHCLKILNNCEKALPEKGKVLIHDFVLQGGGLSQIVDMMMMAHTNGGMERTEEQWRKLLTTVGFSTINFIELFEQEWLIEASK*

>Sphmag12G011400|Sphmag12G011400.1

MAEAHGSKAQQPVADEEEFMADRAVLYPLILGFVCSFVLRTAVVLGLPDIIARAGPDETTVKQIAAQLKSESVNEFGLQRPLTALVKWKLFRCTKAETSKMQYGLTPVSKLLEFWKYCDNPEFAKCFNDGMTAMTNIQSIALRKYEGFKDMKTLVDVGGGIEYVAGDFFESVPSGDAFMKRALPEKGKMSIYDFVLESIGGLPQIFDTMMMTLTNTGMERTEEQWRNLLTTAGFSANFIQLLAEQWLIEAVK*

>Sphmag12G012100|Sphmag12G012100.1

MGEVNVSKVELPVADEEELVVGNDVLYSLLFGFVGSFVLRTAVVLGLPDIIARAGPDGTTLKQIAAQLKSKSVNEFELQRVLSALVTSKIFRCSKVGTTLEMQYGLTPASKLLVTENPNQAPMVLFQTDPAAQAPWQQLHRCVLYGEQPWKSAHGKDAWKYFNDNPELSKCFNDSMAATTVELIAVRKYEGFKDIKTLVDVGGGIGRALETIMSSYPHIHGINYDLPHVIADAPTIGIEHVSGSMFESVPSGDAIFMKHIMHDWNDEDCLKILNNCQRALPEKGKVLIFDFVVPPGGIPQTFDMVMMAHTDGGTERTEEQWRKLLTTAGFSTINYIELLQEQWLIEAVK*

>Sphmag12G012000|Sphmag12G012000.1

MAEANGSKTQAQPVADVEELTARRAVLYRLLFGFAGSFVLRTAVVLGLPDIIARAGPDELTVKQIAAQLKSESVNEFGLQRVLTALVNFQLLRCTEAEMSERQYGLTPVSKLLVTENNHNQTPVVLFMTDPAALAPWGQLHHYVLYGEDAWQYAHGKDIWTSLNDNPELNKCYNASMSATTVELIAVRKYEGFKDIKTLVDVGGGTGKTLETIISSYPHIHGINYDLPRVIADAPTPGIEHVAGSMFESVPSGDAIFMKRIMHDWSDEHCLKILNNCQKALPEKGKVLIHDFVLQGGGLSQIVDMMMMAHTNGGMERTEEQWRKLLTTAGFSTINFIELFEQEWLIEASK*

>Sphmag12G011600|Sphmag12G011600.1

MAEAHGSKAQQPVADEEEFMADRAVLYPLILGFVGSSVLRTAVVLGLPNIIVRAGPDETTVKQIGAQLKSESVNEFGLQRVLTALVKWKLFRCTKAETSKMQYGLTPVSQLLEFWKYCDNPEFAKCFNDGMTAMINIESIALRKYEGFKDIKTLVDVGGGVGKTLETIISTYPHIHGTYDHHFSSTHHACVHIMRCWNCENCLKILNNCQTALPEKGKMFIYDFVLESIGGLPQIFTMMMTLTNTGMERTEQWRNLLTTAGFSAINFIQLLAEQWLIEAVK*

>Sphmag01G164100|Sphmag01G164100.1

MAPNSYSDAQIDLNKQSSSVYYYDAARNAAMELTGYCSVSGCMKAAVELGVFEVLAKAASGRKSLTAKEIAEQLVRPGANGAVVNQGYLQRVLRLLASVNVLSESVEVVAAEPGNLTSNTHHRRSYALTQVGKHFVRGEDGVSLAPLMLMNEDWVFKKTWDHLSAAILDDSVDPFVRHGTSQFQLNNEDSRVDKVFHTAMASHSRLYTQAVLGAYQGFQDVNCLVDVGGGLGSSLAITAKYPHIRGINFDLPHVVAAAPPYPGVECVGGDMFESVPTGDAIFMKWILHDWSDEDCRILRNCLKALPASGGKVIVVDTVLPESISLESENAGARSFVGLRTDVAMLAYNSGGAKETLHEFQQLADAVGFARMELVVTVDFMSVLEFTKTAA*

>Sphmag18G023800|Sphmag18G023800.1

MATNSYRVAHSNGTTGNFGKQSSSVHQNDVARHAAMEFTLYSVVSGTLKAAIELGVFEIAKAGASASQKSLTAKEIAEQLVRPTANGTAVNSGYLQRFLRLLASVNIVSESVAVVAAGPGNFTSYNTHHQRSYALTPIGKYFVQGEDGVSLAPFILMSEDWVFKKAWDHLSAAVLDDVDPFVRAHGKSEFQLNNEDPRVDKLFNTAMSSHSRIYMEAMLGAYDGFQDVNCLVDVGGTGASLASITAKYPHIRGINFDLPHVVATSPTYPMCMIGVEFVGGNMFESIPSGDAIFMKVLHDWNDEDCMTILKNCFKALPSSGGKVIVMESVLPDSINLQFENSGVKSLLGLRIDLVLAYNSGGAKERTLHEFQQLADATGFASLALITTIDFLSVLEFTRVAT*

>Sphmag16G083200|Sphmag16G083200.1

MAEANYDGSKMEQSAAADQEAELMAGHDVLCSLLFGFVGSFLLRAAVVLGLPDIIARAAPDGTLTVKQIAAQMIQKSGVETVNEFELQRVLTALANYKIFRSCTQDVGSDQEMQYGLTASKLLVTENNPHNQAPGVLFQTDPVAQAPYRQLHRCVLYGEQAFQSAHGKDVWKYVNDDEYSKCFNAAMASTTTIDLIAVRKYQGFKDIKTLVDVGGGVGKALETIISTFPHIHGINYLPHVVADAPTMPGIEHVAGSMFESVPSGDAIFMKHIMHDWNDNNCIKILNNCQRALPEKKVLIYDFVVQPSGGLPQIFDVVMMAHTNGGMERTQDQWRKLLTTAGFSAINFIQLMPQQLIEAVK*

>evm.model.chr4.2085

MGSVENGLVIANEDEWLLGMEFGNFSCLPMAMKTAIELDVLQIIANAGDGAQISPREIVHIPTTNPDAAITLDRILRVLASHSVLSSSVTTDKNGKAQRVYGLTALCKYLVQNKDGVSAPLVLMNQDKVLMECWYHLKDAVLEGIEPFTKAHGVNAFQYHAKDPRFNNVFNRGMAEHTMLMEKILDTYQGFENFHELVDVGGGIGSTLNLIVSKYPHIKGICFDMPHVVAEAPHYPLRHIGGDMFDTIPSGQAIFMKWILHDWSDEHCVKILKNCYKALPEGGKVIVVDSILPVAETSPYARQAFHCDLLMLAYNPGGKERTEQEFRDLAKAAGFAGGIKPVCCVNGMWVIEFYQP*

>evm.model.chr4.2087

MGSMEKGLIIANEEEWILGMELGNFSCLPMAMKAAVELDVLQIIANAGPGAQISPREIVHIPTENPDAAITLDRILRVLASHSVLSSSVTTDENGKAERVYGLTPLCKYLVQNKDGVSAPLVLMNQDKVFMESWYYLKHAVLDGSQPFTKAHGVNAFEYPAKDPRFNNVFNRAMAEHTMLMQKILDTYPGFKDVQEIVDVGGGIGSTLNLIVSKYPHIKGVNFDMPHVVAEAPQYPLRHVGGDMFDSVPSGQAIFMKWILHDWSDEHCLRLLKNCHKALPEGGKVIVVDSILPVAETSPYARQAFHCDLLMLAYNPGGKERTEQEFRDLAKATGFAGGIKPICCINGMWVMEFHQD*

>evm.model.chr4.2086

MGSMENGLIIANEDEWLLGMELGNFSCLPMAMKAAVELDVLQLIANAGPGAQISPREIVHIPTTNPDAAITLDRILRVLASHSVLSSSVTTDENGKAERVYGLTPLCKYLVQNKDGVSAPLVLMNQDKVFMESWYYLKDAVLDGSQPFTKAHGVNAFEYPAKDPRFNNVFNRAMAEHAMLMQKILDTYPGFKDVQEIVDVGGGIGSTLNLIVSKHPHIKGVNFDMPHVVAEAPQYPLRHVGGDMFDSVPSGQAIFMKWILHDWSDEHCLRLLKNCHKALPEGGKVIVVDSILPVAETSPYARQAFHFDLLMLAYNPGGKERTEQEFRDLAKATGFAGGIKPVCCINGIWVMEFHQA*

>evm.model.chr4.1806

MGSMEKGLIIANEEEWVLGMELGNFSCLPMAMKAAVELDVLQIIANAGHGAQISPREIVHIPTTNPDAAITLDRILRVLASHSVLSSSVTTDENGKTGRVYGLTPLCKYLVQNKDGVSAPLPFTKAHGVNAFEYPAKDPRFNNVFNRAMAEHSTMLMQKILDTYPGFKDVQEIVDVGGIGSTLNLIVSKYPHIKGVNFDMPHVVAEAPQSPGLRHVGGDMFDSVPSGQAIFMKWILDWSDEHCLRLLKNCHKALPEGGKVIVVDSILPVAAETSPYARQAFHCDLLMLAYNPGGKRTEQEFRDLAKATGFAGGVKPVCCINGMWVMEFHKQA*

>evm.model.chr7.281

MDVLSQHIDPKLQAQALKFIVPFVPGLVLKSAVLLKVPDIIAKAGPDAYLSVHQIAAQLTQNPHLNYLSRILRYLAAVGVFSTTIDTSDEVRYGLTDMTKVFYVTENNPWSLVPMLLMNHEVFMAPWQHLHECVLEGGDAFQKANGKDMWAYGRDDPQVNNIVNSAMASPSKVVMKQVASYDGFSHVKTLVDVGGGLGAALAEISGVYPHIHGINFDLPHVVATAPVLPGIEHVGGMFESVPSGDAIFMKSVLHDWDDERCLKILQNCHKALPENGKIILAERVLPRHSDPNGTMFVVDLAMMAHTNGGRERTEKQWRALLENAGFSQMKILGRADRLPIKIVEGVKITKM*

>evm.model.chr11.1112

MNGPLETNHATMDGLRLFESEEEELEGQAQAWGHIFAFVESLAVKCAALLGIPDIIARHPRATLSLPQIAESLPSKAPDVGCLLRIMRFLVSKNVFTSEVAVTQNGVPETRYGLTPSSWLLKHNQYLSMRPMLLMQNDKRSVAPWHHFNECVLEGGIAFERANGADIWSYASKDPEYHLFNDAMACNAKMVMKAILSKYDGFTALNSLVDVGGGIGTGIAEIVSVYPHIRGINYDLHVVATAPPFPGVEHIGGDMFASVPSADAVFMKWIMHDWNDEDCIKILKECRKAIPEDRGVIIADVVLNEEGRKKRALDPVGLVFDLVMVAHSSGGKERTEEEWRKLLSRAGFNRYNIIIPALQSIIEAFPS*

>evm.model.chr7.280

MDAHSQHIDPKLQAKALEFIVPFVPGLVLKSAVLLRIPDIIARAGPDAYLSVHQIAAQLTQNPHLDNLSRILRYLAAVGIFSAAVDSSNRVRYGLTDMAKVFYVTENNHLSLVPVLLMNHEVFMAPWQHLHECVLEGGEAFQKANGKDMWAYGRDDPHVNNIVNSAMASSSKVVMKQVASYDGFKDAKMVVDVGGGVGAALAEIIGAYPHIHGINFDLPHVVATAPVLPGIEHVGGMFESVPSGDAIFMKSVLHDWDDEECLKILENCHKALPENGKIILAEIVLPQLSNPNGARFVVDLAMIAHTSGGRERSEEQWRALLENAGFSQIEILGRADRLPIKIVEGVKIAKM*

>evm.model.chr6.245

MAARASSTEPPSSVDAQHKLYEMILCFAKPMALKSAVLLNIPDIIASEGHGDSLSLQQIAHISPPPQSMEYLLRILRFLASYDVFTERQWKRNGADKFFCESYHHLNESVLEGGYAFNAFGKSPWDYVSEHPNYNKTFNDGMVSHTRALMASALKIYDGFKNFKSVVDVGGGVGTAIMIAKHHTHLKVINFDLPHVIDTAPAIPGVEHVGGNMFEQIPPADAVFIKSILHDWDDEHVRILNKCREAIPENGKVIIVDVIVERNEGSMRRLGLLWDMIMMAFTTGGKERTEEEFKVFEKAGFKSYTLLKLPAVQSIVELSKA*

>evm.model.chr10.1216

MDCSGDQVREIIEEKITGHEEDMESLIELCKRMFGFMDSLGLRWAVEMGIPIRTLFPNPPNPPSRLLLICPLSTEREIVGKEGKEVEYGLTPISKWLLTENGSCMNSMVMMQTHEAVFPWYHFGECVLRGGFPFEVVHGKSMFSFAKNNPDFGKLFNDAMASHSGMLMKHILSVYGDDRFKDLKSLVDVGGGDGTTVSQIAHSFPHIKCYNYDLPQVIHQAPPYPGVQHIAGDMFMVPHADAVFMKMILHDWGDEECMQILRNCRQAIPVDGKVIIVDVVLTKDGVRRRGFEEIGMMDMVMLAHQRGKERSEEQWKELLQASGFDCYKITPLPDHLSIIEAVPADRD*

>evm.model.chr6.356

MAANASSEPHTLDAKHKLYEMILSFAKPMTLKAAVLLNIPDIIASEGHGASLSLQQIAAISPPSQSMEYLLRILRFLASYDVFTESVHVVRGEEKQWSFGLTAISELLVQKGNQHSLAFLLLAADKFFCESYHHLDESVLEGCYAFNKVYSKSPWEYVSEHPNFNKTFNDGMVSHTRLMASALKIYDGFRDVKTVVDVGGGVGTAISIIVKHHTHLKGINFDLPHVIATAPAIPGVHVGGNMFEQIPPADAVFIKWILHDWDDEDCVRILKKCREAIPDNGKVIIVDAVVERNEGLRRLGLLFDITMMTFTTRGKERTEDEFKLLFEKAGFKSYAFLKLPAVQSIIELYKV*

>evm.model.chr6.355

MAGNASSEPHTLDAQHKLYEMILCFAKPMALKAAVLLNIPNIIASEGHGASLSLQQIAAISPPPQSMEYLLRILRFLASYDVFTESVHVVRGEEKQWSFGLTAISELLVQKGNQHSLAFLLLVADKFFCESYHHLDESVLEGCYAFNKVYSKSPWEYVSEHPNYNKTFNDGMVSHTRLMASALKIYDGFRDVKTVVDVGGGVGTAISIIVKHHTHLKGINFDLPHVIATAPAIPGVHVGGNMFEQIPPADAVFIKWIIHDWDDEDCVKILKKCREAIPENGKVIIVDAVVERNEGLRRLGLLFDIAMMTFTTGGKERTEEEFKLLFEKAGFKSYAFLKLTAVQSIIELYKV*

>evm.model.chr8.207

MADKYSNRDSLFENEEEELRGHAQVWKYVLAFVDSMAVRCAVLLGIPDMISREGHHATLLSEISEKLPTKSPDVGCLFRLLRFLVSKNVFSAKSVVRADNGICETRYGLTPVSKWLVTSRDVNLVPMFLMINQTNLAPWYCFNECILNGGITAFERANGAEPWSFAASHPEYSKIFNNMACNTKISMKALLSNYDGFQSLNSLVDVGGGTGTAAEEIVRAYPHIKAISYDLPHVVAAPSRPGVEYVGGDMFAGVPSADAVFLKWIMHDWNDEDCIRILNQCRNAIPETGKVIIVDVIAEESDEKENGILRETKLVLDLVMVTNTIGGKERTEAEWKRVLCGAGFGRYNIISIQAHSVIEAFPSESTIY*

>evm.model.chr6.246

MATSSSSIEPPSSVDAQHKLYEMILCLAKPMALKAAVLLNIPDIIASEGHGDSLSLQQIAHISPPPQSMEYLLRILRFLASYDVFTESLQVVGEETQWRFRLTAISELLVQKGNQQSLPFLLMAADNFFSESFHHLHESVLEGGSAFNKAYGKSPWEYVSEHPKYNKIFNDGMVCHTTFMASALKIYDGFKNFKTVVDVGGGVGTAISLIVKHHTHLRGINFDLPHVIATAPAIPGENVGGNMFEQIPPADAVFIKWILHDWDEEQCVRILNKCREAIPENGKIIIVDAVVERNKSMRRLGLSFDIAMITFTSGGKERTEEEFKALFEKAGFKSYTLLNLPAVQSIIELSKD*

>evm.model.chr10.1211

MDYKRGCSGHEVQEITEDKITAHAEDMEGFIELWKRMFGFMDSLGLRWAVEMGIPDIISAGPQSAQQIASHLPCESPQVDFISGILRFLAMRGVFTEIVDKEGKEEVEYGLTAISKWLTENGSCMNSMVMMQTHVAVFAPWYHFGESVLRGGFPFEVVHGKSLFPFAKDNPDFGKLFDAMASYSRMLLKHVLSVYGDEDRFKDLKSLVDVGGGDGTTVSQIAHSFPHIKCYNYDLPVIQDAPPYPGVQHIAGDMFMTVPHADAVFMKLILHDWGDEECMQILRNCRQAIPVDGKVIVDVVLTKDGVRRRGFEEIGVMMDMVMLAHQRGKERSEEQWKELLQASGFDCYKITPLPHLSIIEAVPADRN*

>evm.model.chr10.1222

MDHSKSGSSNEHGEEEEQNKIHVRDQEDMDGFIELWKRMFGFMDSLGLRWAVEMGIPDISKSGPQSAQQIASHLPSESPHIDFISRILRFLAMRGVFTETVGEEEKEVRYGLTPISKWLTENSRCMNSVILVQTHEKDIAPWYFFGQCVLRGGFPYEIAHGKQRFSFAKDDPDFNKLNDSMASCSRTLIGNILTTYSDGFKRLKSLVDVGGGDGTTISNIAQSFPHIKCYNYDLPHIQEAPPYPGVEHIAGDMFISVPHADAIFMKTVLHDWEDEECKKILKNCRQAIGQEDGHVIVDAVLREAGKKRSGFDEIGVMMDMVMLSHQRGKERSESQWKELLEASGFDRYSFIPLPSEMSIIEAFPSI*

>evm.model.chr10.672

MASSAEEFSSASFHPCDDGNDHENLWRSQASFWNMTFSILPAMVVKSALRLRLPDIIARGPDAALSLQDIAAHLPTQTPNLDALSRILCYLSRVGIFTQSIANDRVGDTSSETRYGLTLGKSCFVVENNPSSLAPFLLLLTHQALTTAWNHFDACVLEGPNAFEKCHGKDLWPYAESSDFNQTFNASMEALTRAVMKGILSTYDGFRDVKTLVDVGGGVGQALRDIVAAYPHIRGIFDLPHVIATAPTVPGIHHVSGNFFEDSLPCADAFFMKHVLHAWDDELCCKLLNNCHKALENGKLIVAEEVVKVSPGHADPVESGEAMDMIMLGVTRGGRERSERQWRELLAGSGFRLTIVGRAGNIVKIIEAVKF*

>evm.model.chr10.1212

MAMDRSKSGSSNQHEEEEQNKIHVRDQEDMEGFIELWKRMFGFMDSLGLRWAVEMGIPDISKSGPQSAQQIASHLPSESPQIDFISRILRFLAMRGVFTETVGEEEKEIRYGLTPISKLLTENSDCMNSMILVQTHEKDIAPWYFFGQCVLRGGFPFEMAHGKRRFSFTKDDPDFNKFNDSMASYSRMLINNILTTYGDGFKRLKSLVDVGGGDGTTISKIAQSFPHIKCYNYDLPVIQDAPPYPGVEHIAGDMFISVPHADTIFMKTVLHDWGDEECKKILKNCRQAIGQEDGHIIVDAVLREAGKKRSGFDEIGVMMDMVMLSHQRGKERSESRWKELLEASGFDRHNIIPLNSQMSIIEAFPSISFS*

>evm.model.ctg25062.1

MRMLGFNGLLGRRWQVEWVLRDIISKSGPQSAQQIASHLPSESPQVAFISRILRFLAMRVFTEAVGEEDQKEIRYGLTPISKWLVTDNTENGSMNLMVLMETHEALVAPWYRFGECVLGGFPFDVMHGKPFFSFSKDNPGFNKLFNDAMASYSGMLMKHILAAYSDSGFKGLKSIVDGGGNGTAISKVAEAFPHIKCYNYDLPQAVQDAPPYPGVEHIAGDMFETVPHADAVFMKLLQDWGDEECMQILKNCGKAIPEDGKLIIVDAVLTEAGKKRSDLEEIALILDMVMLAHQRKERTEQQWGDLLRASGFDRYNIIPLPSQISIIEAFPALAH*

>evm.model.chr6.247

MAARASSTEPPSSVDAHYKLYEMILCFAKPMALKAAVLLNIPDIIASEGHGDSLSLQQIAHISPPPQNMEYLLRILRFLASYDVFTESMQVVGEEKQWRFGLTAISELLVQRGNQQSLPFLLLIEDKFFCESYHHLNESVLEGCYAFNKAYSKSPWEYLSEHHKHNKTFNDAMVSDTAFMPLALKIYDGFKDVKTVVDVGGGVGTAISIIVKHHAHLKGINFDLPHVIDTAPAITGEHVGGNMFEQIPPADAVFMKSILHDWTDEHCVRILNKCREAIPENGKVIIMDVIVERNESLRRLGLLWDMTMMAFTSGEKERTEEEFKVLFEKAGFKSYTVLKLPAVQSIIELSKG*

>evm.model.chr10.1253

MGDVNGSKMEEMDQVRLDGSTQTHEDMQGFVDLRKRMFGFMDSLGLRWAVEMGIPDIIAFGSQTVQDIASRLPSQSPQIEFISRTLNFLAMRGVFTKKICDDKKVRYGLTPISKWLVTNAKFCVNPVVLMNTHEAMVTSWYRFGECGLRDGVPFQVAHGKPLFSFAKDNPEFGKLFRGIATLSAPRMKPIILAYGEGFKRLKSLVDVGGGDGDAVATIQQSFPHIKCYNYDLPEVIDAPAYPGVEHIGGDMFVSVPRADALLLKLVLHNWGDKECINILRNCRHAIPEDGKLLIVGVVDKNSDGSDIAVTMDMYMLAYFGGKERTEEQWRELLHASGFDRYRFIPLSNPQFFIIAFF*

>evm.model.chr4.2088

MGSMEKGLVIANEEKWVLGMELGNFSCLPMAMKAAVELDVLQIIANAGHGAQISPREIVHIPTTNPDAAITLARIFRVLASHSVLSSSVTTDENGKTERVYGLTPLYKYLEQNKDGVSAPLLLMSQDKVFMESWYYLKDAVLDGSQSFTKAHGVNAFEYPAKDPRFNDVINRAMAEHTMLMQKILDTYPGFNDVQEIVDVGGGIGSTLNLIVSKYPHIKGVNFDMPHVVAEAPQSPLRHVGGDMFDSVPSGYEVSNGILQWILHDWSDEHCLRLLKNCHKALPEGGKSRGKERTEEFRDLAKATGFAGGIKPVCRINGMWVMEFHKQA*

>evm.model.chr1.876

MDATRSQQIDPKAQAQAWDFIFSFVPALVLKSALLLEIPDIIARAGPDAHLSFQQIAAQPTQNPSLDYLSRILRYLAVKGIFTHSTATDTSEVRYGLTDMAKLFVVSESNPLSLVPLLLVNHEVFMAPWHHFHDCVLEGGDAFEKDHAKDLWAYGRSDPQVNDIFNSAMASFTKVSMQIVGSYQGFNDVKTVVDVGGGVGMALAEIIGVYPHIHGINFDLPHVVANAPDLPGIQHVGNMFESVPSGDAIFMKSVLHDWDDKRCLKILENCHKALPENGKIMLAESVLPEHLNLNQ>evm.model.chr1.87MDASRSQQIDPKAQAQAWDFIFSFVPALVLKSALLLKIPDIIARAGPDAHLSLQQIAAQPTQNPSLDYLSRILRYLAVKGIFTHSTATDTSEVRYGLTDMAKLFFVSESNPLSLVPMLMQNHEVFMAPWHHFHECVLEGGDAFEKAHGKDLWAYGRSDPQDVKTVVDVGGGVGMALAIIGVYPHIHGINFDLPHVVANAPDLPGIQHVGGNMFESVPSGDAIFMKSVLHDWDDERCEILENCHKALPENGKIMLAETVLPEQSDPNQGIEILSDLVMIAHANGGRERSEGQWRGLEKAGFSGIKIVGRAEVFSTIVEAVKFTYDVKE*

>evm.model.chr6.1244

KQEEVESHGRLAIMELANMISVPMALNAVVTLNIADIIWQKGANNPLSALQISLQIPTGSSKADAGNLERIMRMLASYNVFKETILTQSNGVSERLYSLTEIGKTLVKDEMGLSFGSYLQHHQKSLLEAWPLLHEAVLDPKHEPFRKAHGKPAYEFYGDDDAANDLMKTAMASVSVPMRTILKSYGGFDGVHRVVDVGGSSGFCLHMIIETHNHIQGINFDLPNVVAGAPSYAGMTVGGDMFESIPSGDAIFMKWILSTWSDEKCIQILRNCYRALPDGGKVIACEPVLPKYTDDQRTRALLEGDIFIMTIYSLGGKERTEEEFSQLGLAAGFSKLVALYLDPFYTVLEFHKCRPKDYIVLFKNIEGDDTHEFFEQFQLFTISGFSLCAMNITFIATGLVEVGE*

>evm.model.chr10.1254

MDDVNGSKMEAMDQVRLDGSTQTHEDMQGFVDLRKRMFGFMDSLGLRWAVEMGIPDIIAFGSQTVQDIASRLPSQSPQIEFISRTLNFLAMRGIFTKKICDDKKVRYGLTPISKWLVTNAKFCVNPMVLMSTHEAMVAAWYRFGECGLRDGVPFQMKPIMLAYGEGFKHLQSLVDVGGDGNAVSTIRQSFPHIKCYNYDLPEVIQDAPAYPGVEHIGGDMFVSVPRADALLLKLVLNWGDKECINILRNCRHAIPEDGKLIIVDGVVDENNDGSEIELTMDMCMLAYLGGKERTEQWSELLHASGFDRYRFIPMSNSKWSIIEAFP*

>evm.model.chr4.869

MALRAASEEISASSALPLEFDNGDQLMSRAWKIIFSMMPPMILQSAVRLKVPDIISRAGGAALSAQDIAAQLTAATQSPCNIDSLSRILTYLSSIGIFAQKIIDHVDDAASGSTREIRALTDMTKAYFVTENNPTSLAPYLLLHTHPTTAAVWNNFAECALGQDRPSAFDKRYGQDFSYTQSHADTNDMFNASMASLTKAVMSDVLPAYDGFKNVNTLVDVSAGVGQALTEIFSAYHIHATNFDRPHVIANAPAVEGIQHVSGNIFEGIPSGDEFFMKHVLHLWDDKECHDILLNYKALPANGKLILAEAVVKVCEDELDPVGCGQVMDMIMLGSTHGGRERTEQQWKDLLGASFRITKIVGRKQSLVKVIEAVKLI*

>evm.model.chr6.1245

MDDNNNNNYNVLEKQEEVESHGRLAIMELANMISVPMALNAVVTLNIADIIWQKGANNPSALQISLQIPTGTSSKADAGNLERIMRMLASYNVFKETILTQSNGVSERLYSLTEIGQTVKDEMGLSFGSYVLQHHQKSLLEAWPLLHEAVLDPKHEPFRKAHGKPAYQFYGDDAAANLMKTAMASVSLPFMRTILKSYGGFDGVHRVVDVGGSSGFCLHMIIETHNHIQGINFDLPVVAGAPSYAGIVFALEILTQVVLAICNVFPYGSLYTGRTCPTAKFSLMKHVLVACVRCGPIPPYDPSVTHVGGDMFESIPSGDAIFMKWILSTWSDEKCIQILQNCYKALPNGGKVIAEPLLPKYTDDSQRTRALLEGDIFIMTIYSLGGKERTEEEFSQLGLAADFSKLVALYLDPYTVLEFHNTTASTRLSTEQG*

>evm.model.chr10.1204

MDCSGDQVREIIEEKITGHEEDMEGLIELCKRMFGFMDSLGLRWAVEMGIPDIISKSGPSVQQIASHLPSESPQREIVGKEVKEVEYGLTPISKWLLTENGSCMNSMVMMETHEALFAWYYFGECVLRGGFPFKEFMGMKTDSKTWKSLVDVGGGHGTTVSQIAHSFPHIKCYNYDLQVIHQSPHYPGVQHIAGDMFMTVPHADAVFMKLILHDWGDEECMQILRNCRQAIPADGKIIVDAVWTKDGNRRSGFEEIAVMLDMLVLANHSGKERSEEQWVELLQASGFDCYNITPLDHLSIIEAVPADRE*

>evm.model.chr4.870

MALVAASEEISASSTLPLEFDNGELISRAWKIIFSMMPPMILQSAVRLKVPDIISRAGPAALSAQDIAAQLTAATQSPCNIDILSRILAYLSSIGIFAQKIIDHVDDTASGRTAGTRERYALTDMTKAYFVTENNPTSLSHADTNDIFNASMASLTKAVMSDVLAAYDGFKDVNTLVVAGGVGQALIEIVSAYPHIHAINFDQPHVIANAPAVEGIQHVSGNIFEGIPSGDAFFMKVLHLWDDKECHDILLNCYNALSANGKLILAEAVIKVCEDELDPVGCGQAMDMIMLGFMHGRERTEQQWKDLLGDAGFRITKIVGRKQSLVKVIEAVKLI*

>evm.model.chr6.862

MAASPSSTEPQSQDAQHKLYEMILSFAKPMALKAAVLLNIPDIIAREGHGDSLSLQQIAHISPPPQSMEYLLRILRFLASFDVFTESLQIVGEDEKLWKFGLTTISELLVQNGNQQSLPFLLLIADKFFCESYHHLNESVLEGCYAFNKVYSKSLWEYVSEHPNYNKTFNDGVVSHTNLMASALKIYDGFKNVKTVVDVGGGVGTAISIIVKHHTHLKRINFDLPHVIATAPAMPGEHVEGNMFEQIPPADAVFLKSILHDWDDEHCVRILKKCREAISGNGENGGRIQSAVREAFKSYTLLKVSAVEAIIELSKA*

>evm.model.chr6.861

MAANDSPTEPPSSVDAQHKLYEMILSFAKPMALKAAVLLNIPDIIAREGHGDSLSLQQIAHISPPPQSMEYLLRILRFLASFDVFTESLQIVGEDEKQWKFGLTTISELLVQNGNQQSAPFLLLIADKFFCESYHHLNESVLEGCYAFNKVYSKSLWEYVSEHPNYNKTFNDGVVSHRNLMASALKIYDGFKNVKTVVDVGGGVGTAISIIVKHHTHLKRINFDLPHVIATAPAMPVEHVEGICSNRYLQQTQYFSRILKKCREAISGNGKVIIVDTLVERDEGTTRRLGLLWDMMMAFTTGGKERTEEEFKVLFEKAGFKSYTLLKVSAVEAIIELSKA*

>evm.model.chr7.385

MSGNSASALAIGHDQKNEGLQALMEMSYLCVFPMAAKAAILLQVPEILAAASGPLSAKQADKIGGNATSEAKLDRLLQALATHGLFSIHQAPPPSLKTYSLNAMSRLLVKDENGSSLAMFLLNTDPSHALPLQYLHEAILDDAAVPFEKAYGQPLFKFVAQSPQMGEAFYTAMSNLSIVTRSILQSYDGFKDVKVLVDVGGGPGLNSSLIKAAHPHISVINFDMPFVVAKASPLPGEHRGGNMLESVPSGGDAILLKVLCSRLVASFDLAMMSVFRGGKERSSKQFEGLVHSARFNFKVVLFGDMLSVMEAFKSEDA*

>evm.model.chr2.691

MAASASSTEPFSSVDAQHKLCEMILCFAKPMALKAAVLLNIPDIIASEGHGDSLSLQEVAHISPPPRSMEYLLRILRFLASYDVFTESLQVVGEEKHWRFGLTAISELLGCYAFNKVYKSPWEYVSEHPKYNEILNDGMVSHTRALMDSALRLQGFRMLRQCGRWRRCWPHFHDRQTPSERHQFRSTHVIDTGCHNGVEHVGGNMFVQIPPADAVFIKCREAIQENGKVIIVDVVVNNARRLRRLELLWDMTMMAFTTGGKERTEEEFKVLFEKAGFKSYTLLKLPAVQSIIELPA*

>evm.model.ctg25063.1

MDNKGGCRGNDQRKEEEIVDAHHQEDMEGFIELWKRMFGFMDSLGLRWAVEMGIPDIISSGPQSAQQIASHLPSESPQVAFISRILRFLAMRGVFTEAVGEEDQKEIRYGLTPISKWLTDNTENGSMNLMVLMETHEALVAPCKDNPGFNKLFNDAMASYSGMLMKHILAAYSDSGFGLKSIVDVGGGNGTAISKVAEAFPHIKCYNYDLPQAVQDAPPYP>evm.model.chr1.87MDASRSQQIDPKAQAQAWDFIFSFVPALVLKSALLLKIPDIIARAGPDAHLSLQQIAAQPTQNPSLDYLSRILRYLAVKGIFTHSTATDTSEVRYGLTDMAKLFFVSESNPLSLVPLLMQNHEVFMAPWHHFHECVLEGGEAFEKAHGKDVWAYGRSDPQVNEIFNSAMAWYPTQSAGILLPGSFWRWNTAPPENGKIMLAETVLPEHSDPNQGIEILSDLVMIAHANGGRERSEGWGGRLEKGRLLWNQNRRKS*
