# Supplementary figures and images for: Walnut N-Acetylserotonin Methyltransferase Gene Family Genome-Wide Identification and Diverse Functions Characterization During Flower Bud Development
Source: Front Plant Sci. 2022 Apr 15;13:861043. doi: 10.3389/fpls.2022.861043 (PMC9051526; doi:10.3389/fpls.2022.861043)

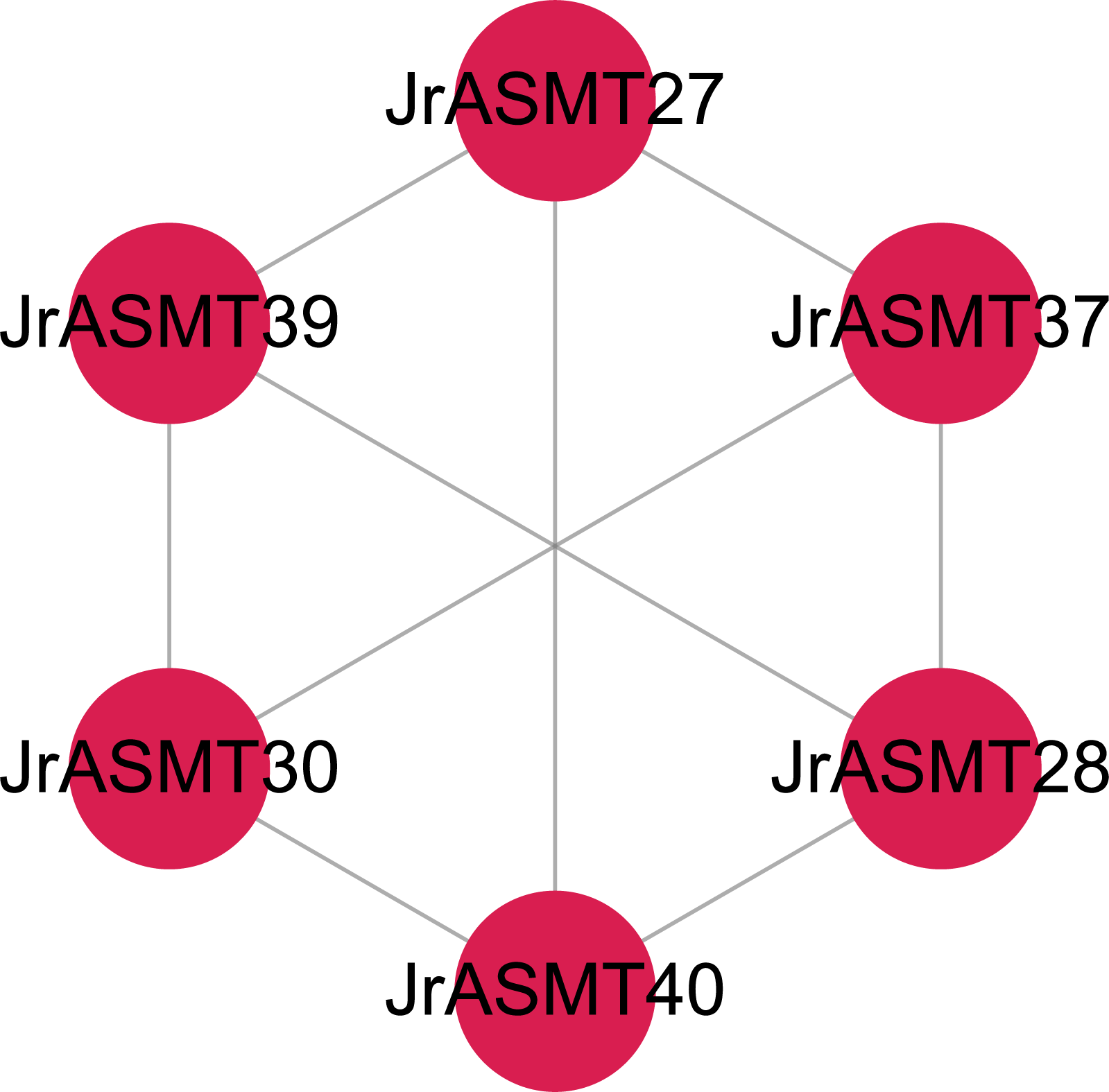

Supplement: Supplementary Figure 1 — Sample clustering tree and trait correlation heat map. [file Image_1.TIF]

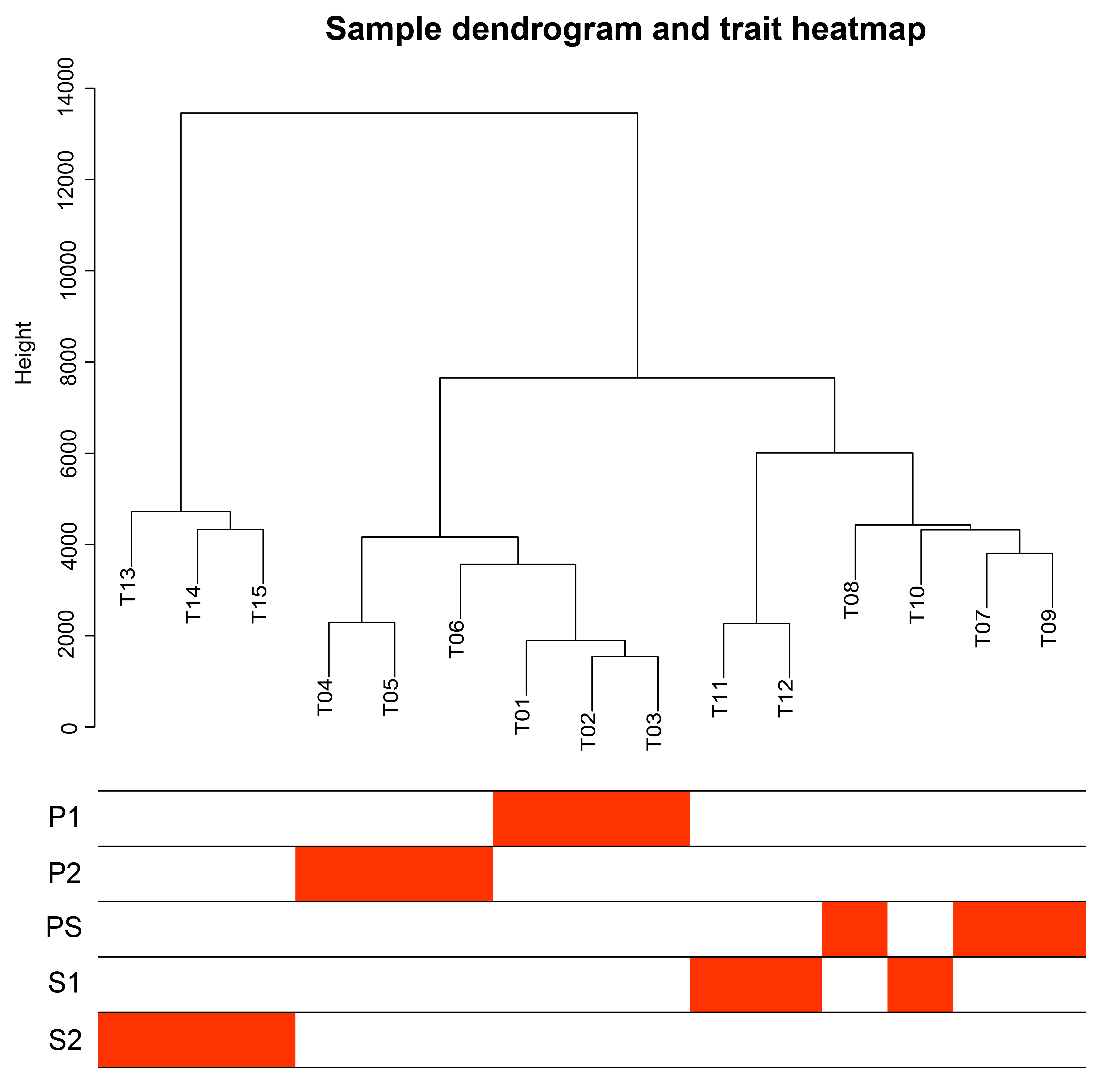

Supplement: Supplementary Figure 2 — Direct interaction network diagram of JrASMT. [file Image_2.TIF]

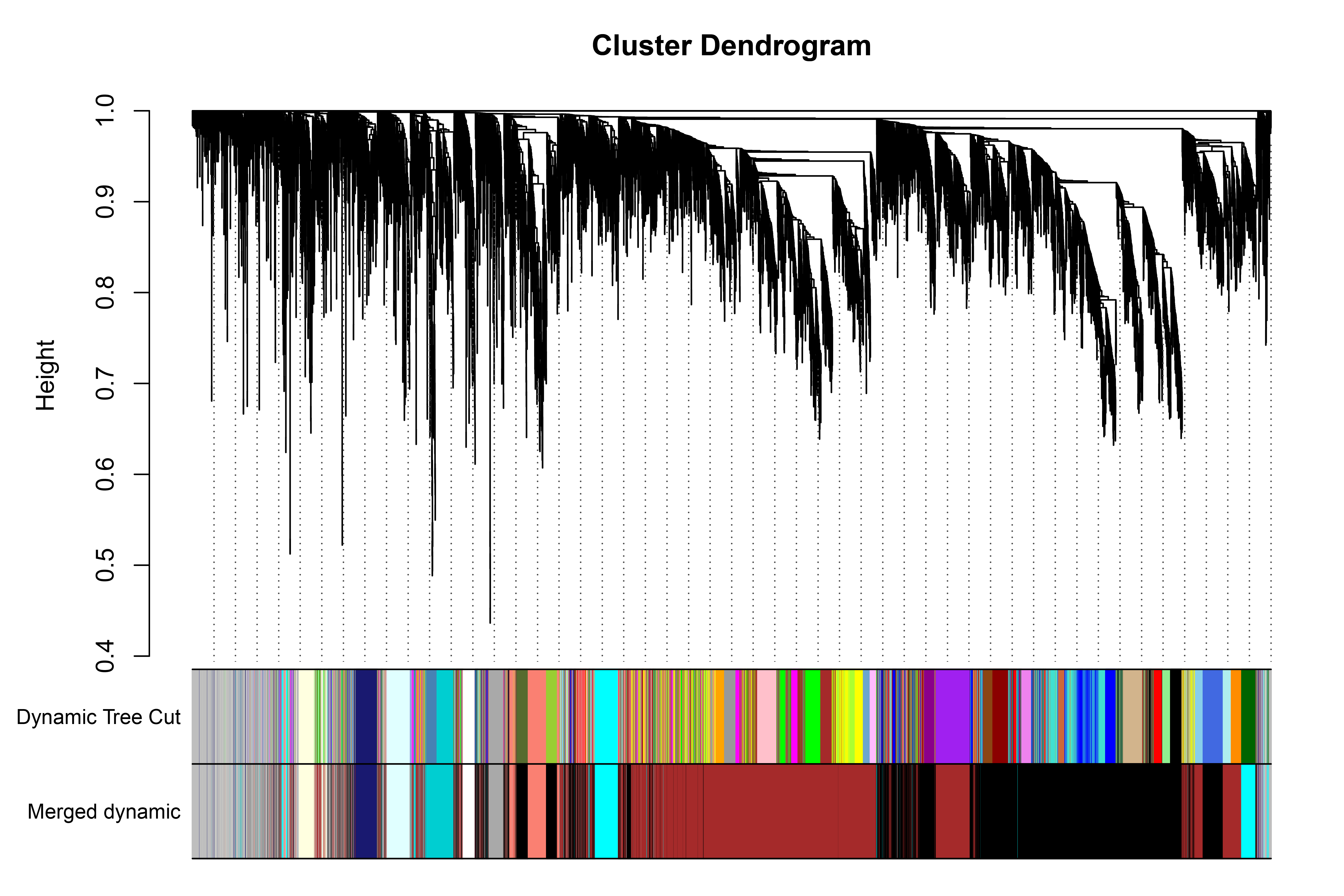

Supplement: Supplementary Figure 3 — Merged dynamic gene modules and genes tree. [file Image_3.TIF]

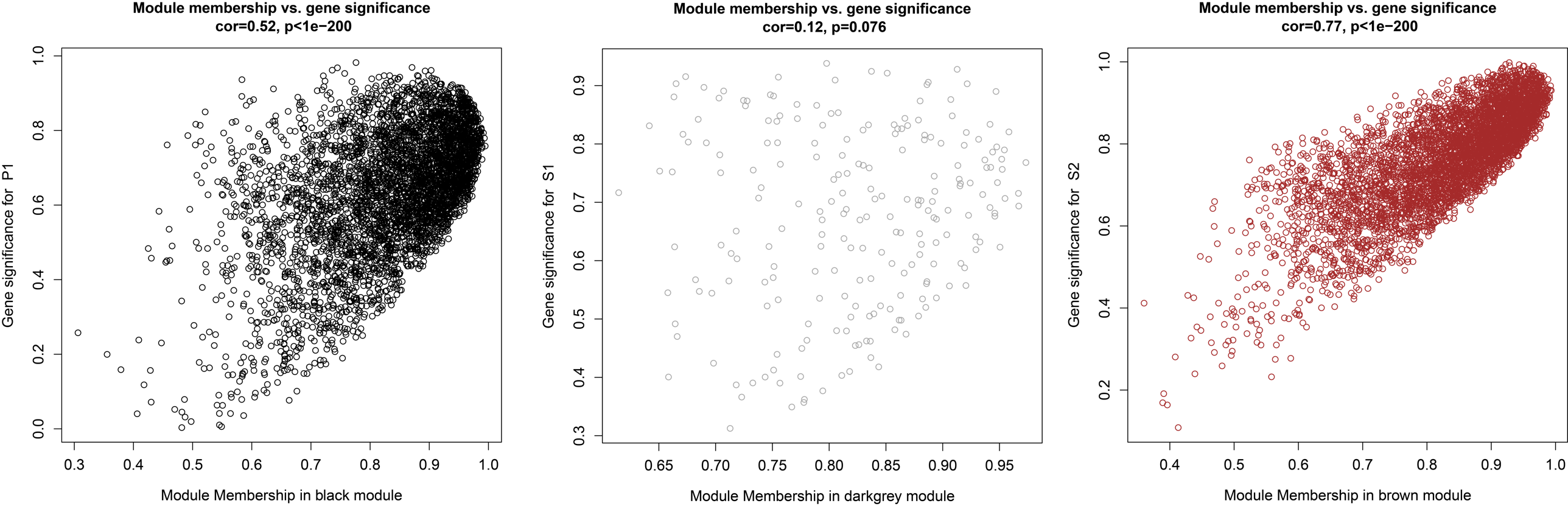

Supplement: Supplementary Figure 4 — Scatter plot of correlation between module membership and gene significance in black, brown and dark grey. [file Image_4.TIF]
